# Bleeding profile of women using a drospirenone-only pill 4 mg over nine cycles in comparison with desogestrel 0.075 mg

**DOI:** 10.1371/journal.pone.0231856

**Published:** 2020-06-29

**Authors:** S. Palacios, E. Colli, P. A. Regidor

**Affiliations:** 1 Salud y Medicina de la Mujer, Director—Instituto Palacios, Madrid, Spain; 2 Exeltis HealthCare Madrid, Madrid, Spain; 3 Exeltis Europe, Ismaning, Germany; Weill Cornell Medical College Qatar, QATAR

## Abstract

**Background:**

Progestin-only pills are associated with irregular bleeding pattern including amenorrhea. Desogestrel 75mcg even being a pill that inhibits ovulation shows a poor cycle control that limits a more common use. A drospirenone (DRSP)-only pill was developed to improve the bleeding profile.

**Methods:**

A phase III study in healthy women aged 18 to 45 years was performed to compare the bleeding profile and safety of women taking a DRSP only pill in a regime of 24 days of 4 mg of DRSP tablets followed by 4 days of placebo versus desogestrel 0.075 mg per day continuously over 9 cycles. A total of 858 women with 6691 drospirenone and 332 women with 2487 desogestrel treatment cycles were analyzed. The primary endpoint was the proportion of women with bleeding/spotting days in each cycle from cycles 2 to 9 and cumulative in cycles 2 to 4 and cycles 7 to 9 including and excluding those with amenorrhea.

**Findings:**

In each cycle, up to cycle 7, the proportion of women with unscheduled bleeding including those which did not bleed was statistically significantly lower in the DRSP group than in the DSG group (p = 0.0001, chi-square test). The mean [SD] number of unscheduled bleeding and spotting days during cycles 2–9 was statistically significantly lower in the DRSP group than in the DSG group (21.5 [22.86] days vs. 34.7 [33.73] days, p = 0.0003, Wilcoxon-rank-sum-test). Excluding amenorrhoeic women following results were obtained: In the cycles 2–6, the proportion of women with unscheduled bleeding was statistically significantly lower in the DRSP group than in the DSG group (p = 0.0001, chi-square test). The mean [SD] number of bleeding days was 8.6 [8.52] days vs. 12.9 [16.47] days, p = 0.0233.

**Conclusions:**

This report describes the improvement in bleeding profile of women using the new DRSP only oral contraceptive in comparison to DSG providing a better quality of live and adherence to the contraceptive method.

EudraCT registration number: 2011-002396-42.

## Introduction

Oral contraceptives are among the most popular forms of contraception. They are divided into combined-oral-contraceptive pills (COCPs), and progestogen-only pills (POPs).

In comparison to COCPs, POPs offer several advantages. The most relevant are: a decreased venous thromboembolism (VTE) risk [1, 2] and fewer metabolic changes [3]. This makes them a suitable option for women who are intolerant to or contraindicated for estrogens (due to migraine or cardiovascular risk factors such as hypertension, hyperlipidemias, obesity, diabetes, smoking habits, etc.) [4, 5].

POPs provide contraceptive efficacy through various mechanisms. Regimens of the first and second generation displayed only incomplete ovulation inhibition. However, due to their additional effects on the cervical mucus and the endometrium, the efficacy is close to COCs. The effectiveness is enhanced by complete ovulation inhibition, but a poor cycle control remains a common side effect [6, 7].

The third generation of POPs introduced the inhibition of ovulation enhancing efficacy with a pearl index like that of COC [7]. Still, problematic bleeding while using POPs is challenging [8].

During a normal menstrual cycle, the endometrium is exposed to circulating sex steroids. It is the sequential exposure of the endometrium to the natural steroids, estradiol, and progesterone, that leads to a characteristic histological feature [8].

Estradiol exposure during the follicular phase is responsible for endometrial proliferation. Exposure to progesterone in the luteal phase results in secretory differentiation. Progesterone is antiestrogenic and inhibits endometrial growth and glandular differentiation. It is the withdrawal of estradiol and progesterone, in the absence of pregnancy, which triggers the onset of menstrual bleeding [9].

Exogenous administration of sex steroids, in the form of hormonal contraception, dramatically influences endometrial histology [9, 10].

The exact mechanisms of problematic bleeding associated with hormonal contraception are largely unexplained. The evidence to date implicates superficial blood vessel fragility within the endometrium and local changes in endometrial steroid response, structural integrity, tissue perfusion, and local angiogenic factors as contributing factors [10].

For many women problematic bleeding will be due to the contraceptive method itself: the pattern and duration of bleeding and the likelihood of this settling will vary. Women may consider that the contraceptive and non-contraceptive benefits of this method outweigh the inconvenience of unpredictable bleeding. Nevertheless, these menstrual disturbances are the most common quoted reasons for discontinuation in up to 25% of users. [11, 12].

In a previous study, the safety and cycle control profile of a novel developed drospirenone (DRSP) only pill was described [2]. The present study aimed to further assess the improvement in the bleeding profile of a drospirenone only pill containing 4 mg over 9 cycles in comparison with desogestrel 0.075 mg.

## Material and methods

This phase III study was a double-blinded, randomized controlled trial including 73 primary and secondary gynaecological health care centres including university hospitals in Austria, Czech Republic, Germany, Hungary, Poland, Romania, Slovakia and Spain. The studies were performed between August 1, 2012, and January 27, 2014. The protocol was designed and conducted according to existing legal regulations, and in accordance with good clinical practice in the conduct of clinical trials and the declaration of Helsinki including recommendations made in the European Medicines Agency (EMA) CHMP Guideline on Clinical Investigation of Steroid Contraceptives in Women. Institutional review board approval was obtained for all study sites.

### Ethical approval

All participants gave their written informed consent for participation in the clinical trial after obtention of the correspondent ethical committee approval.

For each of the investigational centres an ethical approval was obtained (see S4 File with the list of all ethical committees.)

The overall approval for the trial with the leading ethical committee was given the 13.07.2012 by the Landesamt für Gesundheit und Soziales Berlin, Geschäftstelle der Ethik Kommission des Landes Berlin, number 11/0606 EK.

### Study medication

Study medication was DRSP, one tablet of 4mg non-micronised DRSP per day, via oral route, with consecutive administration of 24 active tablets and 4 placebo tablets, and no tablet-free interval between 2 successive cycles.

Desogestrel 0.075 mg (in a regimen of 28 active pills, marketed under trade names such as Cerazette® and Cerazet®) was chosen as the comparator for safety, as it also inhibits ovulation as a POP. It is also the first POP with a missed pill window of 12 hours, instead of the 3 hours allowed by conventional POPs, and is one of the leading POPs on the European market.

Medication compliance was measured using an electronic diary, providing time and hour for each tablet intake, and therefore allowing for calculation of the number of intakes of study medication delayed for more than 12 hours, i.e., more than 36 hours after the previous tablet intake.

### Study populations

**Fig 1:**
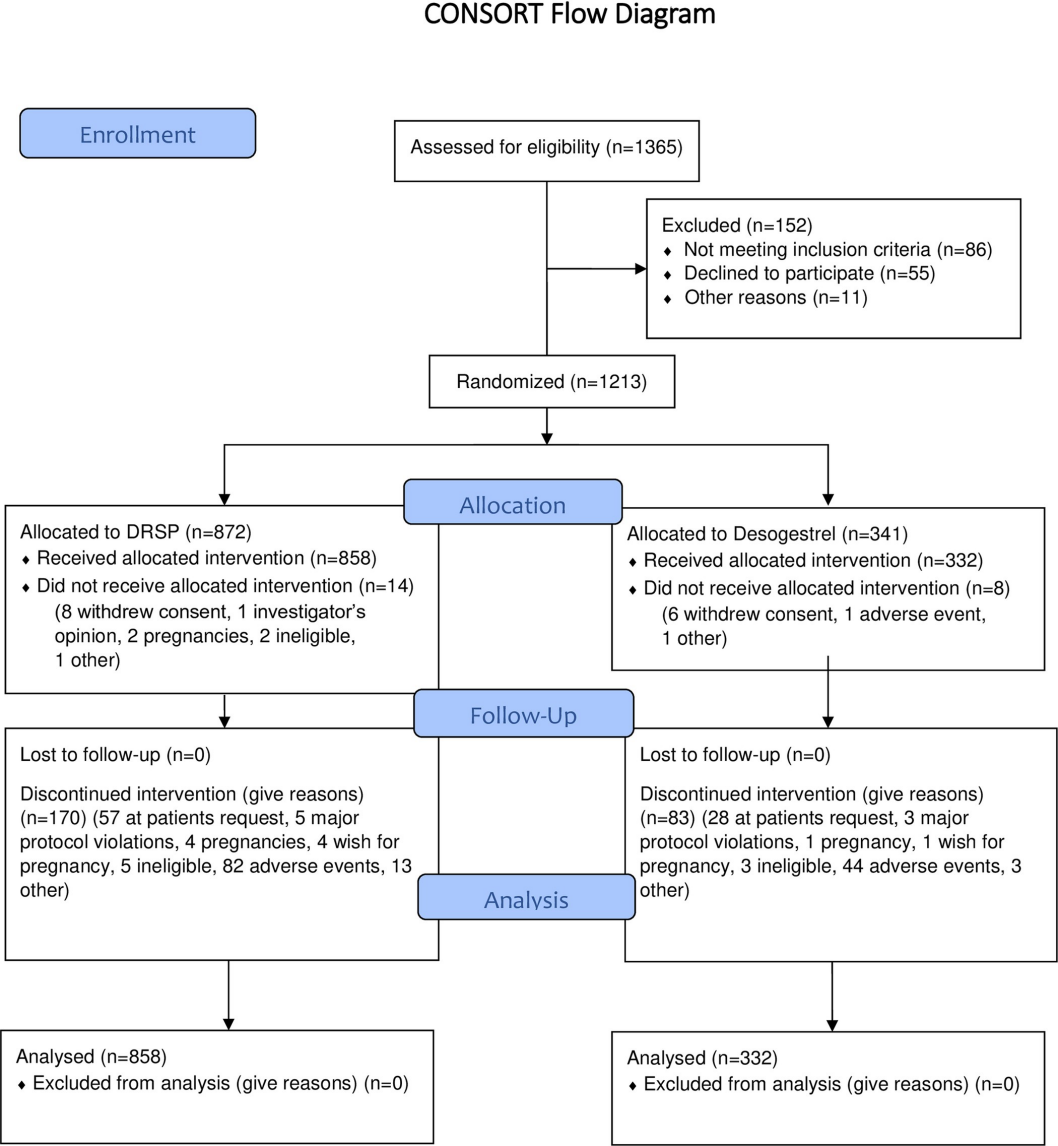
Consort of the study.

A total of 858 women with 6691 drospirenone and 332 women with 2487 desogestrel treatment cycles were analyzed. Fig 1 depicts the randomization and dropout rate. Women included in this study were all of child-bearing potential, at risk of pregnancy, agreeing to use only the study medication for contraception for the duration of the study medication treatment, aged 18 to 45, with systolic blood pressure (SBP) < 140 mmHg, diastolic blood pressure (DBP) < 90 mmHg. They could either start the study medication with a break of at least one day after the administration of another hormonal contraceptive (“starters”) or switch directly from another hormonal contraceptive to the study medication with no break in administration (“switchers”) (Table 1 depicts the clinical data).

**Table 1:** Baseline patients characteristics.

		Study Population	
	Statistic	DRSP 4mg (N = 858)	Desogestrel 0.075mg (N = 332)
Age (years)	Mean (SD)	28.9 (7.1)	28.9 (7.1)
Age group			
≤ 35 years	n (%)	682 (79.5)	259 (78.0)
> 35 years	n (%)	176 (20.5)	73 (22.0)
Ethnicity Caucasian	n (%)	856 (99.8)	331 (99.7)
BMI [kg/m^2^]	Mean (SD)	22.96 (3.537)	22.82 (3.905)
	Min/Max	16.6/41.0	15.9/38.0
BMI group			
< 30 kg/m^2^	n (%)	828 (96.5)	316 (95.2)
≥ 30 kg/m^2^	n (%)	30 (3.5)	16 (4.8)
BP group			
SBP < 130 and DBP < 85 mmHg	n (%)	727 (84.7)	290 (87.3)
SBP ≥ 130 and DBP ≥ 85 mmHg	n (%)	131 (15.3)	42 (12.7)
Subject status			
Switcher	n (%)		
Direct switcher	n (%)	628 (73.2)	259 (78.0)
Indirect Switcher	n (%)	39 (4.5)	14 (4.2)
Starter	n (%)	191 (22.3)	59 (17.8)
Unknown	n (%)	-	-
VTE risk factor			
Presence of at least one risk factor	n (%)	142 (16.5)	59 (17.8)
Previous delivery			
Yes	n (%)	395 (46.0)	150 (45.2)
Regular menstrual bleeding during the last 6 cycles			
Yes	n (%)	786 (91.6)	305 (91.9)
Prior treatment with sex hormones and modulators of the genital system			
Yes	n (%)	469 (54.7)	195 (58.7)

All participants gave their written informed consent for participation in the clinical trial after obtention of the correspondent ethical committee approval.

### Bleeding

Scheduled bleeding or spotting was defined as any bleeding or spotting that occurred during hormone-free intervals (defined as days 25–28 +/- 1). Up to 8 consecutive bleeding/spotting days were considered as scheduled bleeding days. Unscheduled bleeding or spotting days were defined as any bleeding/spotting that occurred while taking active hormones (days 2–23), except days which were classified as scheduled bleeding days. As desogestrel is administered without any free period, no scheduled bleeding is expected. The women recorded any vaginal bleeding or spotting by intensity (slight, moderate, heavy) per each medication cycle in an electronic diary.

### Primary efficacy endpoint

Proportion of women with unscheduled bleeding/spotting in each cycle from cycles 2 to 9 and cumulative in cycles 2 to 4 and cycles 7 to 9 including and excluding amenorrhoeic women.

### Secondary efficacy endpoints

Number of bleeding/spotting days during cycles 2 to 4, 7 to 9 and 2 to 9 and proportion of subjects with no bleeding/spotting including and excluding amenorrhoeic women.

### Safety

Adverse events (AEs), any untoward medical occurrence in a women, reported by the women or observed by the clinical investigator during the study was registered using the case report form (CRF), including duration, causality assessed by investigator, seriousness, severity, frequency, treatment, action taken and outcome. Deviations from the reference ranges of laboratory parameters (thyroid function, haematology, urinalysis, biochemistry, pregnancy test) were evaluated regarding clinical significance by the investigator. Serious adverse events (SAEs) were AEs with any of the following criteria; resulted in death, were life-threatening, required hospitalization, resulted in significant disability or incapacity, congenital abnormalities. Deep vein thrombosis or pulmonary embolism and hyperkalaemia was considered as AEs of special interest and would lead to discontinuation. Vaginal bleeding was considered an AE if it required any additional treatment, led to discontinuation, or fulfilled a seriousness criterion (Table 2 depicts bleeding related AE´s). Abnormal uterine bleeding was present in 27 (3.2%) of the women using drospirenone and in 49 women (6.6%) using desogestrel.

**Table 2:** Number of patients with bleeding or spotting by treatment cycle and period.

Cycle	DRSP 4mg n/m (%)	DSG 0.075mg n/m (%)	Difference (95% CI)	Chi square test p value
Cycle 1	692/765 (90.5)	284/305 (93.1)	-2.66 (-6.18, 0.87)	0.1657
Cycle 2	482/692 (69.7)	211/285 (74.0)	-4.38 (-10.5;1.75)	0.1704
Cycle 3	429/637 (67.3)	160/251 (63.7)	3.60 (-3.37; 10.58)	0.3064
Cycle 4	390/606 (64.4)	161/244 (66.0)	-1.63 (-8.69; 5.44)	0.6531
Cycle 5	351/566 (62.0)	118/219 (53.9)	8.13 (0.41; 15.85)	0.0372
Cycle 6	305/530 (57.5)	110/199 (55.3)	2.27 (-5.82; 10.36)	0.5812
Cycle 7	292/503 (58.1)	91/185 (49.2)	8.86 (0.47; 17.26)	0.0380
Cycle 8	264/468 (56.4)	87/178 (48.9)	7.53 (-1.07; 16.14)	0.0859
Cycle 9	249/442 (56.3)	73/161 (45.3)	10.99 (2.02; 19.97)	0.0167
Cycles 2–4	421/527 (79.9)	192/222 (86.5)	-6.60 (-12.3; -0.95)	0.0324
Cycles 5–7	313/423 (74.0)	106/157 (67.5)	6.48 (-1.95; 14.91)	0.1216
Cycles 7–9	274/374 (73.3)	93/137 (67.9)	5.38 (-3.64; 14.39)	0.2312
Cycles 2–6	346/422 (82.0)	152/172 (88.4)	-6.38 (-12.4; -0.35)	0.0553
Cycles 2–9	256/305 (83.9)	102/116 (87.9)	-4.00 (-11.2; 3.22)	0.3044

n: Number of women with indicated event

m: Number of women in respective cycle

%: Percentage based on m

CI: Confidence interval

### Sample size

To test non-inferiority of the bleeding pattern between the two treatment groups (assuming a 24% proportion of the control group, 9% non-inferiority margin, one sided type I error 2.5%, 80% power, and 2:1 treatment allocation rate) a sample size of 531 in the DRSP group and of 266 in the desogestrel group was required. To prove superiority under the same assumptions a sample size of 443 in the DRSP group and of 222 in the desogestrel group was required. Considering a possible drop-out rate of 20%, 857 DRSP and 333 desogestrel treated women were to be enrolled. A 5:2 ratio was used as the result of this study had to be added to a prior study. For an assumed PI< 1.0 the number of cycles needed to fulfil the precision requirement with 90% power was 12.337. Thus 6.169 cycles were to be collected in the study, requiring 685 evaluable subjects with a treatment duration of 9 cycles.

Randomization was performed by using a validated system that automates the random assignment of treatment groups to randomization numbers. The randomization scheme was completed in a 5:2 ratio using blocking methodology via a center- based randomization method. The randomization data were kept strictly confidential, accessible only until the time of unblinding. The DRSP only group received the test product (DRSP 4.0 mg) in blister a + reference placebo in blister b, and the desogestrel group received the test placebo in blister a + reference product (desogestrel 0.075 mg) in blister b.

### Bleeding records

The tolerability assessments were based on the vaginal bleeding pattern. From Day 1 of Medication cycle 1 (i.e. start of use of the drugs intake) to the end of the clinical trial at day 29 of the last cycle, the women had to record daily any vaginal bleeding or spotting in their electronic diary, which comprised the following details:

- Presence of any vaginal bleeding or spotting (Yes, No)
- Bleeding intensity (slight, moderate, heavy)

### Statistics

The vaginal bleeding pattern statistic was performed on the FAS. Bleeding data were summarized by treatment groups by means of the default summary statistics. The hypothesis that drospirenone is non inferior to desogestrel regarding the proportion of subjects with unscheduled bleeding/spotting during cycles 2 to 6 was tested confirmatory using chi-square test. The number and rate of subjects with different bleeding patterns was presented for each cycle and cumulative in cycles 2 to 4 and cycles 7 to 9. Chi-square test was applied to compare rates in both treatment groups. Numbers of bleeding/spotting days and bleeding/spotting episodes were presented by each cycle and by cycles 2 to 4, 7 to 9 and 2 to 9. The treatment groups were compared using a Wilcoxon-rank-sum-test. Data were also tested for normality. Numbers of missed tablets or entries in the e-diaries for subjects with unscheduled bleeding/spotting were presented by treatment cycle.

## Results

Eight hundred and fifty-eight women were treated with drospirenone only 4mg during the 9-cycles and 332 women were treated with desogestrel 0.075mg.

The proportion of women with bleeding and spotting decreased from 69.7% in cycle 2 to 56.3% in cycle 9 in the DRSP only group and from 74.0% to 45.3% in the desogestrel group; the overall median number of bleeding and spotting days decreased from 10 days (first reference period: cycles 2 to 4) to 6 days (last reference period: cycles 7 to 9) in the DRSP group and from 12 to 7 days in the DSG group. Among these, spotting days prevailed (see Table 2).

The proportion of women with unscheduled bleeding/spotting during cycles 2–6 was lower in the DRSP group than in the DSG group (73.0% vs. 88.4%), with the difference (95% CI) of -15.4% (-21.78%; -8.99%) between the groups. The highest proportion of women with unscheduled bleeding or spotting was observed in cycle 2: 51.4% of the DRSP and 74.0% of the DSG group women. The incidence of unscheduled bleeding decreased over time in both groups, to 43.9% in the DRSP and 45.3% in the DSG group women in cycle 9. In each cycle, up to cycle 7, the proportion of women with unscheduled bleeding was statistically significantly lower in the DRSP group than in the DSG group (p = 0.0001, chi-square test) (Table 3).

**Table 3:** Number of women with unscheduled bleeding or spotting by treatment cycle and period (FAS).

Cycle	DRSP 4mg n/m (%)	DSG 0.075 mg n/m (%)	Difference (%) (95% CI)	Chi square test p value
Cycle 1	375/765 (49.0)	177/305 (58.0)	-9.01 (-15.59; -2.44)	0.0077
Cycle 2	356/692 (51.4)	211/285 (74.0)	-22.59 (-28.90; -16.28)	<0.0001
Cycle 3	319/637 (50.1)	160/251 (63.7)	-13.67 (-20.77; -6.56)	0.0002
Cycle 4	291/606 (48.0)	161/244 (66.0)	-17.96 (-25.12; -10.81)	<0.0001
Cycle 5	252/566 (44.5)	118/219 (53.9)	-9.36 (-17.13; -1.59)	0.0185
Cycle 6	240/530 (45.3)	110/199 (55.3)	-9.99 (-18.10; -1.89)	0.0161
Cycle 7	221/503 (43.9)	91/185 (49.2)	-5.25 (-13.66; 3.16)	0.2198
Cycle 8	202/468 (43.2)	87/178 (48.9)	-5.71 (-14.32; 2.89)	0.1919
Cycle 9	194/442 (43.9)	73/161 (45.3)	-1.45 (-10.42; 7.52)	0.7511
Cycles 2–4	358/527 (67.9)	192/222 (86.5)	-18.55 (-24.56; -12.55)	<0.0001
Cycles 5–7	269/423 (63.6)	106/157 (67.5)	-3.92 (-12.56; 4.72)	0.3799
Cycles 7–9	243/374 (65.0)	93/137 (67.9)	-2.91 (-12.10; 6.28)	0.5392
Cycles 2–6	308/422 (73.0)	152/172 (88.4)	-15.39 (-21.78; -8.99)	<0.0001
Cycles 2–9	243/305 (79.7)	102/116 (87.9)	-8.26 (-15.71; -0.81)	0.0490

n: Number of women with indicated event %: Percentage based on m

m: Number of women in respective cycle CI: Confidence interval

**Fig 2:**
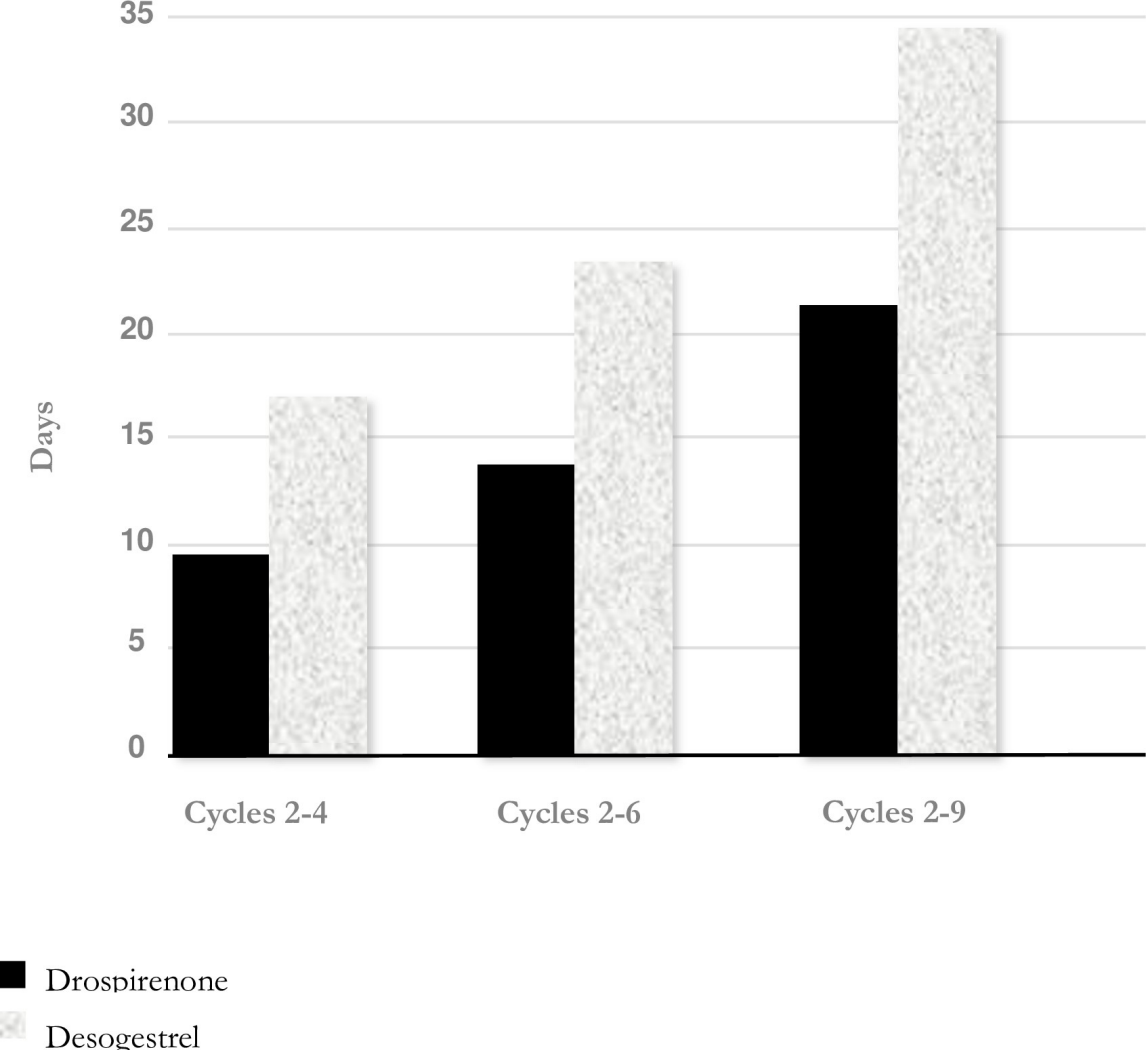
Total unscheduled bleeding/spotting days (mean). P < 0.0003 for all three cycle groups. Drospirenone versus desogestrel.

The mean [SD] number of unscheduled bleeding and spotting days during cycles 2–9 was statistically significantly lower in the DRSP group than in the DSG group (21.5 [22.86] days vs. 34.7 [33.73] days, p = 0.0003, Wilcoxon-rank-sum-test). The mean number of days with unscheduled bleeding and spotting decreased over time and was lower in the DRSP group than in the DSG group in each reference period, and the difference was statistically significant (Table 4 and Fig 2).

**Table 4:** Number of days with unscheduled bleeding and/or spotting by treatment period.

Cycle		DRSP 4mg (N = 858)	DSG 0.075mg (N = 332)	Total (N = 1190)	Wilcoxon-rank-sum-test p value
Cycles 2–4	N	527	222	749	
	Mean (SD)	9.6 (11.58)	16.9 (16.93)	11.7 (13.80)	<0.0001
	Median	5.0	12.0	7.0	
	Min/ Max	0/66	0/79	0/79	
Cycles 5–7	N	423	157	580	
	Mean (SD)	7.4 (9.53)	10.6 (12.69)	8.3 (10.56)	0.0232
	Median	4.0	7.0	4.0	
	Min/ Max	0/67	0/61	0/67	
Cycles 7–9	N	374	137	511	
	Mean (SD)	7.2 (8.85)	10.8 (13.34)	8.2 (10.35)	0.0277
	Median	4.0	7.0	4.0	
	Min/ Max	0/51	0/83	0/83	
Cycles 2–6	N	422	172	594	
	Mean (SD)	13.7 (15.98)	23.7 (24.69)	16.6 (19.44)	<0.0001
	Median	7.0	17.0	9.5	
	Min/ Max	0/89	0/134	0/134	
Cycles 2–9	N	305	116	421	
	Mean (SD)	21.5 (22.86)	34.7 (33.73)	25.1 (26.92)	0.0003
	Median	14.0	26.0	16.0	
	Min/ Max	0/95	0/156	0/156	

N: number of patients in specified treatment group; n: number of patients with data available; SD: Standard Deviation

The mean [SD] number of unscheduled bleeding and spotting days during cycles 2–6, excluding the amenorrhoeic women, was statistically significantly lower in the DRSP group than in the DSG group (18.8 [15.97] days vs. 24.6 [22.68] days, p = 0.0365, Wilcoxon-rank-sum-test). The mean number of days with unscheduled bleeding and spotting decreased over time and was lower in the DRSP group than in the DSG group in each reference period, and the difference was statistically significant (Table 5).

**Table 5:** Mean [SD] number of unscheduled bleeding and spotting days during cycles 2–6, excluding the amenorrhoeic women.

Cycle		DRSP (N = 858)	DSG (N = 332)	Total (N = 1190)	Wilcoxon-rank-sum-test p value
Cycle 1	N	375	137	512	
	Mean (SD)	5.9 (4.53)	5.5 (4.35)	5.8 (4.48)	0.3714
	Median	5.0	4.0	4.0	
	Min/ Max	1/19	1/20	1/20	
Cycle 2	N	355	175	530	
	Mean (SD)	6.5 (4.82)	9.6 (6.63)	7.5 (5.67)	< .0001
	Median	5.0	8.0	6.0	
	Min/ Max	1/27	1/28	1/28	
Cycle 3	N	318	123	441	
	Mean (SD)	6.4 (4.63)	8.1 (5.98)	6.9 (5.10)	0.0062
	Median	5.0	7.0	5.0	
	Min/ Max	1/28	1/28	1/28	
Cycle 4	N	287	123	410	
	Mean (SD)	6.1 (4.78)	8.5 (6.16)	6.8 (5.34)	< .0001
	Median	5.0	7.0	5.5	
	Min/ Max	1/28	1/28	1/28	
Cycle 5	N	251	89	340	
	Mean (SD)	5.9 (4.16)	7.0 (5.44)	6.2 (4.55)	0.1514
	Median	5.0	6.0	5.0	
	Min/ Max	1/24	1/28	1/28	
Cycle 6	N	238	86	324	
	Mean (SD)	5.7 (4.18)	7.8 (5.94)	6.2 (4.79)	0.0082
	Median	5.0	6.0	5.0	
	Min/ Max	1/28	1/27	1/28	

N: Number of women in specified treatment group

n: Number of women with indicated event

SD: Standard deviation

A trend towards less bleeding/spotting days was observed over time. The mean (SD) number of bleeding or spotting days decreased from 13.1 (13.05) days during cycles 2–4 to 9.7 (10.39) days during cycles 7–9 in the drospirenone group and from 16.9 (16.93) to 10.8 (13.34) days in the desogestrel group. The median number of bleeding or spotting days decreased from 10.0 to 6.0 days in the drospirenone group and from 12.0 to 7.0 days in the desogestrel group, respectively.

The number of bleeding/spotting days was lower in the drospirenone than in the desogestrel group at all defined treatment periods. However, the difference between the mean (SD) number of bleeding or spotting days was statistically significant only during the first reference period: 13.1 (13.05) days in the Test vs. 16.9 (16.93) days in the Reference group (p = 0.0149, Wilcoxon-rank-sum-test) (see Table 6).

**Table 6:** Total mean number of bleeding/spotting days.

Cycle		Test (N = 858)	Reference (N = 332)	Total (N = 1190)	Wilcoxon-rank-sum- test p value
Cycles 2–4	N	527	222	749	
	Mean (SD)	13.1 (13.05)	16.9 (16.93)	14.2 (14.40)	0.0149
	Median	10.0	12.0	10.0	
	Min/ Max	0/66	0/79	0/79	
Cycles 5–7	N	423	157	580	
	Mean (SD)	10.2 (11.13)	10.6 (12.69)	10.3 (11.56)	0.6868
	Median	6.0	7.0	6.0	
	Min/ Max	0/67	0/61	0/67	
Cycles 7–9	N	374	137	511	
	Mean (SD)	9.7 (10.39)	10.8 (13.34)	10.0 (11.26)	0.9659
	Median	6.0	7.0	6.0	
	Min/ Max	0/60	0/83	0/83	
Cycles 2–6	N	422	172	594	
	Mean (SD)	19.1 (18.77)	23.7 (24.69)	20.5 (20.74)	0.0894
	Median	14.0	17.0	15.5	
	Min/ Max	0/100	0/134	0/134	
Cycles 2–9	N	305	116	421	
	Mean (SD)	29.4 (27.84)	34.7 (33.73)	30.9 (29.63)	0.2557
	Median	21.0	26.0	22.0	
	Min/ Max	0/109	0/156	0/156	

N: Number of women in specified treatment group. n: Number of women with indicated event. SD: Standard deviation

The percentage of women with frequent bleeding gradually decreased over time from 9.1% during cycles 2–4 to 5.3% during cycles 7–9 in the DRSP group and from 7.2% to 4.4% in the DSG group and was comparable between the treatment groups in each reference period.

Using the definition of prolonged bleeding as an episode lasting more than 10 days the percentage of women treated with drospirenone was 18.1% (cycles 2–4), 11.6% (cycles 5–7) and 9.1% (cycles 7–9) vs 26.1% (cycles 2–4), 20.0% (cycles 5–7) and 16–7% (cycles 7–9) in women treated with desogestrel. The differences were statistically significant in all reference periods as shown in Table 1 below (Table 7).

**Table 7:** Number of women with prolonged bleeding per reference period.

Cycle	DRSP n/m (%)	Desogestrel n/m (%)	P-value*
Cycles 2–4	96/ 530 (18.1)	58/222 (26.1)	0.013
Cycles 5–7	49/ 423 (11.6)	31/155 (20.0)	0.009
Cycles 7–9	34/ 375 (9.1)	23/138 (16.7)	0.015

n: Number of subjects with indicated event.

m: Number of subjects in respective cycle.

%: Percentage based on m.

* P-value was calculated with Pearson's chi-squared test.

### Incidence of TEAEs based on abnormal vaginal (or uterine) bleeding

In total, 46 (5.4%) of the DRSP group and 31 (9.3%) of the DSG group women experienced bleeding- related TEAEs, the majority of which were considered at least possibly related to the investigated products. Most bleeding TEAEs were of mild or moderate severity, whereas TEAEs of severe intensity were reported for four DRSP and three DSG group women.

The number of women who withdrew from the study due to bleeding related adverse events was 27 patients (3.3%) in the drospirenone group and 22 patients (6.6%) in the desogestrel group (p < 0.05).

## Discussion

Decreasing side effects and increasing the satisfaction with contraception is important to help women to believe in the method and continue its use. One the most frequent reasons for stopping the use of contraceptives are problems with the bleeding pattern [13]. These discontinuation rates vary based on the method of birth control, with LARCs having the highest satisfaction and lowest discontinuation rate [13,14,15].

This study proofed the superiority of drospirenone versus desogestrel even though the regimen of both contraceptives used in this trial were different: drospirenone was administered for 24 days followed by a 4-day hormone-free interval, whereas desogestrel was administered for 28 days without any interval. Therefore, subjects who received drospirenone experienced both scheduled and unscheduled bleeding, whereas the users of desogestrel experienced unscheduled bleeding only. Overall the study results confirm the results by Archer et al [2].

In comparison to desogestrel the bleeding pattern with drospirenone showed less bleeding in terms of bleeding/spotting days and episodes, and the contribution of scheduled bleeding days (as opposed to spotting days) to these. Previous studies report comparable differences between ovulation inhibition and hormonal values with drospirenone versus desogestrel [16, 17]. The desogestrel group was characterized by a relatively high proportion of the bleeding pattern variables amenorrhea, infrequent bleeding, frequent bleeding, and prolonged bleeding when compared to the group taking drospirenone. The percentage of women discontinuing treatment because of irregular bleeding was higher in the desogestrel group and even lower or like COC´s irrespectively if used continuously or not [18]. The current study demonstrated that with increased treatment duration, amenorrhea and infrequent bleeding, i.e., less bleeding, became more common. This phenomenon was also observed in the desogestrel collaborative study [12].

The number of bleeding/spotting days decreased, as well as the number of bleeding/spotting episodes. At the same time the proportion of women who had no bleeding or spotting increased from 30.3% to 43.7% women in the drospirenone and from 26.0% to 54.7% in the desogestrel group. Taken together, the bleeding became lighter and shorter in both groups, with an increasing number of subjects reporting absence of bleeding.

The limitation of the study can be seen in the in the rate of patients with data available for the bleeding and spotting analyses during the 9 cycles of treatment. The number decreased from 90.5% in cycle 1 (data available of 692 from 765 patients) to 56.3% in cycle 9 (data available of 249 from 442 patients) for the drospirenone group. A similar reduction was observed in the desogestrel group (93.1% in cycle 1 and 45.3% in cycle 9). Another limitation is that we compared two different dosing regimens (continuous vs 24 +4) and this makes the analyses of some parameters like scheduled bleedings more challenging.

As the clinical contraceptive efficacy of this new DRSP only pill is similar to those COC containing DRSP and/or to the POP containing desogestrel [19] and the bleeding profile is also close to that of COC, this new DRSP only pill will enhance compliance widening the group of women able to use this contraceptive method.

## Conclusion

This is the first comparative trail between two POPs regarding bleeding profile. A decrease was observed in all groups of treatment from the start of treatment to the last period in the number of women with overall bleeding/spotting and in the number of women with unscheduled bleeding/spotting, as well as in the number of days of overall and unscheduled bleeding/spotting. In comparison with desogestrel, the use of DRSP was associated with a significantly lower rate of women with overall and unscheduled bleeding/spotting during cycle 2 to 4, and with a significantly lower number of overall bleeding/spotting days during cycles 2 to 4 and a significantly lower number of unscheduled bleeding/spotting days during the whole 9-cycle comparative study. Early study withdrawals associated with abnormal bleeding was reported for 3.3% DRSP women *vs* 6.6% desogestrel women. Hence the introduction of DRSP as an new estrogen free contraceptive improves quality of life as a better bleeding pattern is observe.

## Supporting information

S1 Checklist (DOCX)

S1 File Original study plan and statistical analyses. (PDF)

S2 File List of clinical trial centres part 1. (PDF)

S3 File List of clinical trial centres part 2. (PDF)

S4 File List of ethical committee centres. (PDF)

## References

[1] Lidegaard Ø, Løkkegaard E, Jensen A, Skovlund CW, Keiding N. Thrombotic stroke and myocardial infarction with hormonal contraception. N Engl J Med. 2012; 366(24):2257–2266. 10.1056/NEJMoa1111840 22693997

[2] Archer DF, Ahrendt HJ, Drouin D. Drospirenone-only oral contraceptive: results from a multicenter noncomparative trial of efficacy, safety and tolerability. Contraception. 2015; 11;92(5):439–44. 10.1016/j.contraception.2015.07.014 26232513

[3] Regidor PA. The clinical relevance of progestogens in hormonal contraception: Present status and future developments. Oncotarget. 2018 10 2;9(77):34628–34638. 10.18632/oncotarget.26015 eCollection 2018 Oct 2. 30349654 PMC6195370

[4] Medical eligibility criteria for contraceptive use, 5th ed 2015 World Health Organization. ISBN 978 92 4 154915 8. 26447268

[5] Kiley JW, Shulman LP. Estradiol valerate and dienogest: a new approach to oral contraception. International journal of women's health. 2011; 3: 281–286. 10.2147/IJWH.S22645 Epub 2011 Aug 18. 21892339 PMC3163658

[6] McCann MF, Potter LS. Progestin-only contraception: a comprehensive review. Contraception, 1994; 50, (Suppl. 1): 9–21. 10.18773/austprescr.1999.007 10226677

[7] Rice C, Killick S, Hickling D, Coelingh Bennink H. Ovarian activity and vaginal bleeding patterns with a desogestrel-only preparation at three different doses. Human Reproduction 1996; vol.11 no.4:.737–740. 10.1093/oxfordjournals.humrep.a019245 8671319

[8] Noyes RW, Hertig AT, Rock J. Dating the endometrial biopsy. Fertil Steril 1950; 1: 3–25. 10.1016/s0002-9378(16)33500-1 31623748

[9] Jabbour HN, Kelly RW, Fraser HM, Critchley HO. Endocrine regulation of menstruation. Endocr Rev 2006; 27: 17–46. 10.1210/er.2004-0021 16160098

[10] Smith OP, Critchley HO. Progestogen only contraception and endometrial breakthrough bleeding. Angiogenesis 2005; 8: 117–126. 10.1007/s10456-005-9003-z 16211361

[11] Kovacs G. Progestogen-only pills and bleeding disturbances. Hum Reprod. 1996 10;11 Suppl 2: 20–3. 10.1093/humrep/11.suppl_2.20 8982741

[12] Collaborative Study Group. A double-blind study comparing the contraceptive efficacy, acceptability and safety of two progestogen only pills containing desogestrel 75 μg/day or levonorgestrel 30 μg/day. Eur J Contracept Reprod Health Care. 1998; 3:169–78. 10.3109/13625189809167250 10036599

[13] Villavicencio J, Allen RH. Unscheduled bleeding and contraceptive choice: increasing satisfaction and continuation rates. Open Access Journal of Contraception 2016:7 43–52. 10.2147/OAJC.S85565 29386936 PMC5683158

[14] Moreau C, Cleland K, Trussell J. Contraceptive discontinuation attributed to method dissatisfaction in the United States. Contraception. 2007;76(4):267–272. 10.1016/j.contraception.2007.06.008 17900435

[15] Trussell J. Contraceptive failure in the United States. Contraception. 2011;83(5):397–404. 10.1016/j.contraception.2011.01.021 21477680 PMC3638209

[16] Duijkers IJM, Heger-Mahn D, Drouin D, Colli E, Skouby S. Maintenance of ovulation inhibition with a new progestogen-only pill containing drospirenone after scheduled 24-h delays in pill intake. Contraception. 2016 4;93(4):303–309. Epub 2015 Dec 17. 10.1016/j.contraception.2015.12.007 26708301

[17] Duijkers IJ, Heger-Mahn D, Drouin D, Skouby S A randomised study comparing the effect on ovarian activity of a progestogen-only pill (POP) containing desogestrel and a new POP containing drospirenone in a 24/4 regimen. Eur J Contracept Reprod Health Care. 2015;20(6):419–27. 10.3109/13625187.2015.1044082 Epub 2015 Jun 15. 26073333

[18] Edelman A, Micks E, Gallo MF, Jensen JT, Grimes DA. Continuous or extended cycle vs. cyclic use of combined hormonal contraceptives for contraception. Cochrane Database Syst Rev. 2014 7 29;(7):CD004695 10.1002/14651858.CD004695.pub3 25072731 PMC6837850

[19] Healthcare Bayer. Yaz Summary of Product Characteristics; 2015.

