# Supplementary material for: Bleeding profile of women using a drospirenone-only pill 4 mg over nine cycles in comparison with desogestrel 0.075 mg
Source: PLoS One. 2020 Jun 29;15(6):e0231856. doi: 10.1371/journal.pone.0231856 (PMC7323950; doi:10.1371/journal.pone.0231856)
Supplement: S1 File — (PDF) [file pone.0231856.s002.pdf]

Prof. Dr. Pedro-Antonio Regidor

This supplement contains the following items:

1. Original protocol and final protocol, summary of changes.
2. Original statistical analysis plan, final statistical analysis plan, summary of changes.

Laboratorios León Farma S.A.

CLINICAL TRIAL REPORT

A Pivotal, Multicenter, Double-Blind, Double-Dummy, Randomised Trial on the Contraceptive Efficacy, Tolerability and Safety of LF111 (Drospirenone) Over 9 Cycles in Comparison with Desogestrel 0.075 mg

**Product Name**	LF111 (Drospirenone)
**Indication**	Oral contraception
**Protocol Number**	CF111/302<br>incl. pooled efficacy results with CF111-301
**EudraCT Number**	2011-002396-42
**Report Version**	Final Version 1.0
**Phase**	III
**Date First Subject Entered**	01-AUG-2012
**Date Last Subject Completed**	27-JAN-2014
**Coordinating Investigator**	Dr. Santiago Palacios<br>Instituto Palacios<br>Calle Antonio Acuña, 9<br>28009 Madrid, Spain<br>Phone: +34 91 578 24 96<br>Fax: +34 91 431 99 51
**Name of Sponsor Legal Representative Signatory**	Dominique Drouin<br>Directeur du Développement Endocrinologie & Gynécologie<br>CHEMO France<br>7 rue Victor Hugo<br>92310 Sèvres, France<br>Phone: +33 1 49 66 22 26<br>Fax: +33 1 41 14 99 17<br>E-mail: dominique.drouin@chemofrance.com
**Report Issue Date**	10-JUL-2014

This trial was performed in compliance with Good Clinical Practices (GCP) and applicable regulatory requirements, including the archiving of essential documents. The information contained in this document is the property of Laboratorios León Farma S.A. and may not be disclosed to parties not associated with the clinical investigation or used for any purpose without prior written consent of Laboratorios León Farma S.A.

1. APPROVAL SIGNATURES / LIST OF AUTHORS

1.1 Sponsor Approval Signatures

TRIAL TITLE:	A Pivotal, Multicenter, Double-Blind, Double-Dummy, Randomised Trial on the Contraceptive Efficacy, Tolerability and Safety of LFI 11 (Drospirenone) Over 9 Cycles in Comparison with Desogestrel 0.075 mg
TRIAL NUMBER:	CFl 11/302

1, the undersigned, have read this report and confirm that to the best of my knowledge it accurately describes the conduct and results of the trial.

SIGNATURE: DATE:

July 10th 2014

Dominique Drouin, Directeur du Développement
Endocrinologie & Gynecologie, CHEMO France
(Legal representative of Laboratorios Le6n Farma S.A.)

1.2 Coordinntin lnvc::sHgntor Appr ovalSignatur<.:

TRIAL TLTLF: A Pivolal. MulLicentel', Doublc·Blind D<.luhJe . l) ummy, RandomiscdTrial ()n thc. Conlr3c1>tivc Enic:,cy, I <1l« ohilityandSafctyof LI'111 (l>m.spirenonc) Ovcr 9 Cyclcs in Compari on wi th Dcsogcstrcl 0.075 ing

TRIAI, NIJi'vIBER: Cf ll 1/302

1, lh undcrsigncd, have read this rc.port and confinn lhat 10 tJ1c best of 01)' knowlcd .gc it occuratcly <lcscrihe thc conduc1 a.nd rcs uhs of the trial.

SIGNATlJRE: DATE:

, CA-/· _ ---.::,; 10/JUL/2014

D w gol'alacios

Ipst l t\11.() Pa la cios
Ca ll c Antooio1\cuiia. 9
28009 Madrid, Spain
l'honc: +3491 578 2496
Fax: 1349143199 51

2. SYNOPSIS

Name of Sponsor:<br>Laboratorios León Farma S.A.	Individual Trial Table Referring to Part of the Dossier	*(For National Authority Use only)*
Name of finished product:	Volume:	
Name of active ingredient:<br>Drospirenone	Page:	

Title of trial:	A Pivotal, Multicentre, Double-Blind, Double-Dummy, Randomised Trial on the Contraceptive Efficacy, Tolerability and Safety of LF111 (Drospirenone) Over 9 Cycles in Comparison with Desogestrel 0.075 mg
Coordinating Investigator:	Dr. Santiago Palacios, Instituto Palacios, Calle Antonio Acuña, 9, 28009 Madrid, Spain
Number of trial centres and countries:	73 centres in Austria, Czech Republic, Germany, Hungary, Poland, Romania, Slovakia and Spain
Sponsor:	Laboratorios León Farma S.A.<br>La Vallina s/n, Polígono Industrial de Navatejera<br>24008 Navatejera (León), Spain<br>Phone: + 34 619 275 590, Fax: +34 197 668 963
Sponsor legal representative:	Dominique Drouin<br>Directeur du Développement "Endocrinologie & Gynécologie"<br>CHEMO France<br>7 rue Victor Hugo, 92310 Sèvres, France<br>Phone: +33 1 49 66 22 26, Fax: +33 1 41 14 99 17<br>E-mail : dominique.drouin@chemofrance.com
Publication (reference):	None.
Studied period (years):	Date of first subject entered: 01-AUG-2012<br>Date of last subject completed: 27-JAN-2014
Phase of development:	Phase III
Objectives:<br>Primary:	• To demonstrate the contraceptive efficacy of LF111
Secondary:	• To demonstrate the safety and tolerability of LF111 in comparison to desogestrel 0.075 mg, especially regarding bleeding pattern
Trial design:	Prospective, multicentre, randomised, active control, double-blind, double-dummy trial.<br>After providing informed consent at Visit 1a (screening) and receiving study medication at Visit 1b, subjects attended Visits 2 to 4 at Day 24±2 of the 1st, 3rd, and 6th cycle, and Visit 5 at Day 29+2 of the 9th cycle. The follow-up (Visit 6) took place 7-10 days after last IMP intake.

Name of Sponsor:<br>Laboratorios León Farma S.A.	Individual Trial Table Referring to Part of the Dossier	*(For National Authority Use only)*
Name of finished product:	Volume:	
Name of active ingredient:<br>Drospirenone	Page:	

Number of subjects (planned and analysed):	Planned: 1200 (857 for LF111 and 343 for desogestrel 0.075 mg)<br>enrolled: 1365; randomised: 1213 (872 Test; 341 Reference);<br>withdrawn: 275; analysed (efficacy, safety and tolerability): 1190 (858 Test; 332 Reference)
Main criteria for inclusion:	Woman without uncontrolled current diseases at risk of pregnancy, at the age of 18-45 years, systolic blood pressure < 140 mmHg, diastolic blood pressure < 90 mmHg
Test drug,<br>dose and mode of administration,<br>batch numbers:	LF111 film-coated tablets (24 tablets containing 4 mg drospirenone followed by 4 placebo tablets), oral administration once daily<br>Manufacturer: León Farma, S.A.<br>LFD0158A, LFD0187A, LFD0217A, LFD0228A
Test placebo,<br>dose and mode of administration,<br>batch numbers:	28 film-coated placebo tablets, oral administration once daily<br>Manufacturer: León Farma, S.A.<br><br>LFD0162A, LFD0182A, LFDO226A
Reference drug,<br>dose and mode of administration,<br>batch number:	Desogestrel 0.075 mg film-coated tablets (28 active tablets),<br>oral administration once daily, manufacturer: N. V. Organon<br><br>LFD0180A, LFD0179A
Reference placebo,<br>dose and mode of administration,<br>batch number:	28 film-coated placebo tablets, oral administration once daily<br>Manufacturer: León Farma, S.A.<br><br>LFD0161A, LFD0183A, LFDO213A, LFDO225A
Duration of treatment:	9 x 28 days
Criteria for evaluation:	
<u>Efficacy variables</u>:	Primary endpoint: Overall Pearl Index (PI)<br>Secondary endpoints:<br>• PI for method failures<br>• PI after correction for back-up contraception and sexual intercourse status<br>• Overall pregnancy ratio<br>• Method failure pregnancy ratio

<table>
<tr><td>Name of Sponsor:
Laboratorios León Farma S.A.</td><td rowspan="3">Individual Trial Table Referring to Part of the Dossier

Volume:

Page:</td><td rowspan="3">(For National Authority Use only)</td></tr>
<tr><td>Name of finished product:</td></tr>
<tr><td>Name of active ingredient:
Drospirenone</td></tr>
<tr><td><u>Safety</u>:

<u>Tolerability</u>:</td><td colspan="2">• Adverse events (AEs)
• Clinical laboratory tests (haematology, biochemistry and urinalysis for all Safety Set subjects; haemostatic variables, carbohydrate metabolism and bone metabolism for a subset of subjects)
• Vital signs (blood pressure, heart rate and body weight)
• Gynaecological examination, intravaginal ultrasound and cervical cytology
• Physical examination
• 12-lead electrocardiogram (ECG) for a subset of subjects
• Clinical laboratory parameters
• Special clinical laboratory parameters (haemostatic variables, carbohydrate metabolism and bone metabolism) for a subset of subjects
• Gynaecological, cervical smear and intravaginal ultrasound examination
• Physical examination
• Proportion of subjects with unscheduled bleeding/spotting in Cycles 2 to 6 (confirmatory)
• Proportion of subjects with unscheduled bleeding/spotting in each cycle from Cycles 2 to 9 and cumulative in Cycles 2 to 4 and Cycles 7 to 9
• Number of bleeding/spotting days during Cycles 2 to 4, 7 to 9 and 2 to 9
• Number of bleeding/spotting episodes during Cycles 2 to 4, 7 to 9 and 2 to 9
• Proportion of subjects with no bleeding/spotting</td></tr>
<tr><td>Statistical methods:</td><td colspan="2">The sample size of subjects taking LF111 (857 subjects were to be enrolled) was based on the primary efficacy endpoint, overall Pearl Index. The sample size of subjects taking desogestrel 0.075 mg (333 subjects were to be enrolled) was based on the secondary tolerability endpoint, the proportion of subjects with unscheduled bleeding/spotting in Cycles 2 to 6. Desogestrel in this study was used for the comparison of the products' vaginal bleeding pattern and safety.</td></tr>
</table>

Name of Sponsor: Laboratorios León Farma S.A.	Individual Trial Table Referring to Part of the Dossier	*(For National Authority Use only)*
Name of finished product:	Volume:	
Name of active ingredient: Drospirenone	Page:	

	An analysis of the primary efficacy variable defined as overall PI was performed for the Full Analysis Set (FAS). The two-sided 95% confidence interval (CI) for the overall PI was calculated assuming that events of pregnancy had a Poisson distribution.<br>The secondary efficacy analysis was based on the FAS. The two-sided 95% CIs were calculated for the method failure PI, PI after correction for additional contraception and sexual intercourse status, overall and method failure pregnancy ratio.<br>The cumulative pregnancy rate was calculated by means of *Proc lifetest* procedure. The cumulative pregnancy probability was calculated using the Kaplan Meier estimator.<br>Safety analyses were performed for the Safety Set (SS).<br>All treatment-emergent adverse events (TEAEs) were summarised by calculating the number and percentage of subjects with AEs by preferred term and system organ class. Also TEAEs were summarised by severity and relationship to treatment. Number and percent of serious adverse events (SAEs) and TEAEs leading to study termination were provided. In addition, summaries of TEAEs for defined subgroups were provided.<br>Clinical laboratory variables were summarised for each treatment group at each visit. Laboratory values were compared using 2-sample t test. Summaries of quantitative parameters (haematology, biochemistry, haemostatic, carbohydrate metabolism and bone metabolism) by treatment group were provided. Shift tables were provided to illustrate changes with respect to the laboratory reference ranges from baseline to endpoint. The number and percent of subjects with values outside the limits of clinical significance were summarised. Vital signs variables were summarised for each treatment group at each visit, including within-subject change from baseline at key time points. Absolute change in body weight from baseline was compared between the treatment groups using ANCOVA with age and body weight at baseline as covariates and treatment group as a fixed factor. Absolute and relative changes of systolic and diastolic blood pressure from baseline to endpoint were tested using ANCOVA with age and the respective blood pressure at baseline as covariates and treatment as a fixed factor. Summary of quantitative vital signs values by treatment group and for each defined subgroup were presented. For gynaecological examination, intravaginal ultrasound, cervical

Name of Sponsor: Laboratorios León Farma S.A.	Individual Trial Table Referring to Part of the Dossier	*(For National Authority Use only)*
Name of finished product:	Volume:	
Name of active ingredient: Drospirenone	Page:	
	cytology and physical examination, n umbers and proportion of subjects with normal and abnormal findings, including assessment of clinical significance, as well as shift tables from screening to Visit 5/EDV were presented. ECG was assessed for a subset of subjects. The number and proportion of subjects (SS) with clinically significant ECG abnormalities were summarised by treatment group and compared using Fisher’s test. Summaries of ECG parameters (heart rate; RR, PR, QRS and QT duration; QTcB and QTcF) and interpretation (normal or abnormal) were provided for a subset of subjects and compared between the groups using 2-sample t test and fisher’s exact test (for interpretation only).<br><br>Tolerability analysis included the vaginal bleeding pattern and was performed on the FAS. Tolerability data were summarised by treatment groups by means of the default summary statistics. The hypothesis that the Test is non inferior to the Reference with regard to the proportion of subjects with unscheduled bleeding/spotting during Cycles 2 to 6 was tested confirmatorily using χ^2 test. The number and rate of subjects with different bleeding patterns was presented for each cycle and cumulative in Cycles 2 to 4 and Cycles 7 to 9. χ^2 test was applied to compare rates in both treatment groups. Numbers of bleeding/spotting days and bleeding/spotting episodes were presented by each cycle and by Cycles 2 to 4, 7 to 9 and 2 to 9. The treatment groups were compared using a Wilcoxon-rank-sum-test. Numbers of missed tablets or entries in the e-diaries for subjects with unscheduled bleeding/spotting were presented by treatment cycle.	

Name of Sponsor: Laboratorios León Farma S.A.	Individual Trial Table Referring to Part of the Dossier	*(For National Authority Use only)*
Name of finished product:	Volume:	
Name of active ingredient: Drospirenone	Page:	

**Summary of results**<br>Subject disposition and baseline characteristics:	Of the 1365 subjects screened, 152 subjects were screening failures and 1213 subjects were randomised in a ratio 5:2 to treatment with either Test (872 subjects) or Reference (341 subjects) medication. Of 1213 subjects randomised, 1191 received IMP and 22 subjects prematurely terminated the trial before the start of treatment.<br>Of 1191 treated subjects, 253 (21.2%) subjects terminated prematurely: 170 subjects (19.8%) in the Test and 83 subjects (24.9%) in the Reference group. The most common reasons for discontinuation in both treatment groups were adverse events and withdrawal of consent. In total, 688 (78.9% of the Randomised Set) Test group and 250 (73.3%) Reference group subjects were completers.<br>The Safety Set and the Full Analysis Set comprised 1190 subjects each: 858 (98.4% of the randomised subjects) in the Test and 332 (97.4%) in the Reference group.<br>All but three FAS subjects were of Caucasian ethnicity. The mean (SD) subjects' age was 28.9 (7.1) years in each treatment group ranging from 18 to 45 years. The majority of women, 682 subjects (79. 5%) in the Test and 259 (78.0%) in the Reference group, were 35 years of age or younger. In total, 176 (20.5%) Test group and 73 (22.0%) Reference group subjects were older than 35 years. Over 70% of the FAS subjects in each group had completed high school or had a university degree. No statistically significant differences between the groups with regard to subjects' age, ethnicity or highest education level completed were observed.<br>Current smokers comprised 27.6% of the Test and 31.0% of Reference group subjects.<br>No statistically significant differences in weight (p = 0.846, 2-sample t test), height (p = 0.439, 2-sample t test) or BMI (p = 0.560, 2-sample t test) were observed between the treatment groups at screening. The proportion of subjects with BMI ≥ 30 kg/m^2 was comparable between the groups (3.5% of the Test and 4.8% of the Reference group subjects). Subjects with systolic blood pressure (SBP) > 140 mmHg, or diastolic blood pressure (DBP) > 90 mmHg were not eligible. At screening, 15.3% of the Test and 12.7% of the Reference group subjects had SBP ≥ 130 mmHg or DBP ≥ 85 mmHg.<br>887 (74.5%) FAS subjects switched directly from another oral contraceptive, whereas 250 (21.0%) were starters.<br>The most common previous medical or surgical history finding in both

Name of Sponsor: Laboratorios León Farma S.A.	Individual Trial Table Referring to Part of the Dossier	*(For National Authority Use only)*
Name of finished product:	Volume:	
Name of active ingredient: Drospirenone	Page:	

groups was Caesarean section, followed by dysmenorrhoea in the Test group and by vaginal infection in the Reference group. The most frequent ongoing medical history findings were dysmenorrhoea and breast pain.

No major differencies between the treatment groups were observed with regard to the VTE risk factors assessed.

In total 395 women (46.0%) in the Test and 150 women (45.2%) in the Reference group reported having had at least one delivery. Prior miscarriages were reported by 7.9% of the Test and 7.2% of the Reference group subjects, and prior abortions by 15.3% of the Test and 17.5% of the Reference group subjects.

No statistically significant differences were observed between the groups with regard to prior bleeding characteristics. The vast majority of subjects (91.6% of the Test and 91.9% of the Reference group subjects) reported having had scheduled/regular bleeding during the last 6 cycles prior to screening, unscheduled bleeding was uncommon (1.0% of the Test versus 2.1% of the Reference group subjects), absence of more than one bleeding was reported by 0.8% of the Test and 1.8% of the Reference group subjects. Moderate intensity of scheduled/regular bleeding prevailed (68.9% subjects in the Test group versus 70.2% in the Reference subjects). The incidence of previous spotting was low (2.3% Test group to 4.0% Reference group subjects).

At screening, 232 FAS subjects (19.6% of the Test and 19.3% of the Reference group subjects) reported that they had suffered from dysmenorrhoea within six cycles prior to screening. At follow-up, 150, i.e. more than a half of these subjects, reported having no dysmenorrhoea. In total, 145 FAS subjects (11.2% of the Test and 14.8% of the Reference group) experienced mastodynia/mastalgia within six cycles prior to the screening and 81 subjects of these had no mastodynia/mastalgia at follow up.

In total, 992 (83.4%) of FAS subjects reported at least one prior medication or contraceptive method (range: 82.1% Test to 86.7% Reference group subjects). The most common of these were sex hormones and modulators of the genital system (range: 54.7% Test to 58.7% Reference subjects). Approximately 30% of the subjects in each treatment group reported intake of at least one concomitant medication. The most common were analgesics (7.5% Test to 8.4%

Name of Sponsor: Laboratorios León Farma S.A.	Individual Trial Table Referring to Part of the Dossier	*(For National Authority Use only)*
Name of finished product:	Volume:	
Name of active ingredient: Drospirenone	Page:	

Reference group subjects), antibacterials for systemic use (6.9% in each treatment group), and antiinflammatory and antirheumatic products (6.8% of the Test and 6.9% of the Reference group subjects).

Based on tablet count, the mean (SD) overall compliance to the IMP (FAS) was very high: 101.7 (14.13)% in the Test and 101.9 (5.93)% in the Reference group. In total, 163 subjects (13.6%) missed at least one pill. Compliance above 100% was achieved not because the subjects took more tablets than prescribed, but due to unreturned tablets or blisters.

Efficacy results:

The primary efficacy variable was the overall Pearl Index. A total of 858 subjects with 6691 drospirenone and 332 subjects with 2487 desogestrel treatment cycles were analysed. During these cycles five Test group and one Reference group subjects became pregnant, all pregnancies occured in the age group ≤35 years and were considered method failure. Secondary efficacy analyses included overal PI after correction for additional contraception and sexual activity status, method failure PI and pregnancy ratio.

The method failure PI was calculated based on sexual activity cycles without additional contraception where the e-diary documented regular pill intake during the cycle, excluding the cycles with four or more days with forgotten tablets (i.e. no records in the diary on tablet intake), or two or more consecutive days with forgotten tablets (i.e. no records in the diary on tablet intake) during the cycle and no protocol deviations having effect on this cycle.

The PI Indices for the FAS and for the age group ≤35 years subjects are presented in the table below:

	**Test**<br>**PI (95% CI)**	**Reference**<br>**PI (95% CI)**
Overall PI	0.9715 (0.3154; 2.2671)	0.5227 (0.0132; 2.9124)
Overall PI for subjects ≤ 35 years	1.2428 (0.4035/2.9004)	0.6767 (0.0171; 3.7705)
Overall PI after correction for additional contraception and sexual activity status	1.0875 (0.3531/2.5379)	0.5845 (0.0148; 3.2568)
Overall PI after correction for additional contraception and sexual activity status for subjects ≤ 35 years	1.4000 (0.4546/3.2670)	0.7598 (0.0192; 4.2333)
Method failure PI	1.4006 (0.4548/3.2684)	0.7159 (0.0181; 3.9885)
Method failure PI for subjects ≤ 35 years	1.8351 (0.5959/4.2826)	0.9319 (0.0236; 5.1922)

Name of Sponsor: Laboratorios León Farma S.A.	Individual Trial Table Referring to Part of the Dossier	*(For National Authority Use only)*
Name of finished product:	Volume:	
Name of active ingredient: Drospirenone	Page:	

The PI point estimate for the Test group was higher than for the Reference group. The PI calculation for the Reference group, however, was less precise, as it was based on a considerably lower number of cycles, resulting in a much wider confidence interval. The upper limit of the PI 95% CI was lower for the Test group than for the Reference group.

The cumulative 9-cycle pregnancy ratio (95% CI) in the Test group was 0.70% (0.09; 1.31), and in the Reference group it was 0.34% (0.00; 1.01). For the age subgroup ≤ 35 years, it was 0.90% (0.11; 1.68) in the Test vs. 0.44% (0; 1.31) in the Reference group.

Pearl Indices for DRSP users in pooled CF111/301 and CF111/302 trials

A total of eight in-treatment pregnancies, assessed as being method failure, were observed in women who used DRSP 4.0 mg up to 13x28 day cycles in CF111/301 and CF111/302 trials. All eight pregnancies were reported for subjects ≤ 35 years. The total number of analysed exposure cycles for the overall PI was 14329. The PI Indices for the FAS and for age group ≤35 years subjects are presented in the table below:

	**Total (N=1571)**	**Subjects ≤ 35 years (N=1251)**
	**PI (95% CI)**	**PI (95% CI)**
Overall PI	0.7258 (0.3133; 1.4301)	0.9332 (0.4029; 1.8387)
Overall PI after correction for additional contraception and sexual activity status	0.7898 (0.3410; 1.5562)	1.0223 (0.4414; 2.0144)
Method failure Pearl Index	0.9682 (0.4180; 1.9077)	1.2702 (0.5484; 2.5027)

The cumulative 13-cycle pregnancy ratio (95% CI) of DRSP users (FAS) in both trials was 0.72 (0.17-1.27), and that of the age subgroup ≤ 35 years it was 0.93 (0.21-1.64).

Safety and tolerability results:

The mean (SD) treatment duration was 222.7 (65.79) days in the Test group and 213.9 (72.14) days in the Reference group. The median duration was 252.0 days in both groups, ranging from 3 to 276 days in the Test and from 1 to 280 days in the Reference group.

The proportion of subjects with TEAEs was lower in the Test than in the Reference group (38.7% vs. 45.2%), and this difference was statistically significant (p = 0.042, Fisher's exact test). The most frequently affected SOCs were infections and infestations, and

Name of Sponsor: Laboratorios León Farma S.A.	Individual Trial Table Referring to Part of the Dossier	*(For National Authority Use only)*
Name of finished product:	Volume:	
Name of active ingredient: Drospirenone	Page:	

reproductive system and breast disorders.

The most common individual TEAEs in both treatment groups were vaginal haemorrhage (3.7% of the Test and 7.2% of the Reference group subjects), headache (4.4% Test and 5.1% Reference group subjects), acne (3.1% Test and 5.7% Reference) and nasopharyngitis (3.4% Test and 3.9% Reference). Vaginal haemorrhage and acne were more frequent in the Reference group than in the Test group. The treatment groups were comparable with regard to the frequencies of the other TEAEs. The treatment groups were comparable with regard to the frequencies of other most frequent TEAEs.

The most common TEAEs considered by the investigators as at least possibly related were vaginal haemorrhage, acne and weight increased.

The vast majority of TEAEs were classified as mild or moderate, severe TEAEs were reported for 2.8% of the Test and 3.3% of the Reference group subjects.

There were no deaths reported. In total 15 (1.7%) Test group and six (1.8%) Reference group subjects experienced treatment emergent SAEs. Of these, two TESAEs, hepatic adenoma reported in the Test group and ectopic pregnancy reported in the Reference group were assessed as possibly related to study treatment and were reported as SUSARs. After the database lock, some doubts have arisen regarding the diagnosis of hepatic adenoma in favour of focal nodular hyperplasia. The diagnosis will be clarified in July 2014, when the results of MRI and ultrasound examination are available.

TEAEs of special interest (hyperkalaemia and blood potassium increased) were reported for two Test group subjects. The subjects did not present clinical signs related to hyperkalaemia. In the Reference group no TEAEs based on increased blood potassium levels were reported. No VTE cases were reported during the trial.

Overall 82 (9.6%) Test group and 44 (13.3%) Reference group subjects experienced TEAEs, leading to premature termination of the trial. The most frequent TEAEs leading to withdrawal were vaginal haemorrhage (2.6% of the Test and 5.4% of the Reference group subjects) and acne (1.0% of the Test and 2.7% of the Reference group subjects).

Overall 12 pregnancies occurring after the start of IMP intake were

Name of Sponsor: Laboratorios León Farma S.A.	Individual Trial Table Referring to Part of the Dossier	*(For National Authority Use only)*
Name of finished product:	Volume:	
Name of active ingredient: Drospirenone	Page:	

reported in this trial (six on-treatment and six post-treatment). All pregnancies occurred in the Test group, except one extrauterine pregnancy in the Reference group. Five (including twins) normal male babies were born.

Haematology, biochemistry, TSH and urinalysis laboratory assessments were performed in all trial subjects, while haemostatic, carbohydrate metabolism and bone metabolism assessments were performed in a subset of 68 subjects.

The changes of haematology, biochemistry, TSH, haemostatic and carbohydrate metabolism parameters over time were not clinically relevant and the between-group differences were small.

The levels of bone remodelling markers (bone alkaline phosphatase and Beta-CTX) were within the range for premenopausal women, not treated with contraceptives and no statistically significant differences between the treatment groups were found. The changes over time in each treatment group were assessed as not clinically significant.

The mean [SD] weight increase from baseline to endpoint was less pronounced in the Test group than in the Reference group (0.1 [3.2] kg vs. 0.5 [3.1] kg), the difference between the groups was statistically significant (p=0.0296, ANCOVA with age and body weight or BMI at baseline as covariates and treatment group as a fixed factor). The mean (SD) BMI in the Test group increased by 0.04 (1.17) kg/m^2, and that of the Reference group by 0.20 (1.11) kg/m^2, with a statistically significant difference (p = 0.0331, ANCOVA).

No relevant changes in blood pressure or heart rate over time or differences between the groups were observed for the Safety Set, as well as for the age and BMI subgroups. In the subgroup of subjects with SBP≥130 mmHg or DBP≥85 mmHg, blood pressure decreased over time: The median change in SBP from baseline at endpoint was -7.0 mmHg in the Test and -8.0 mmHg in the Reference group. The median change of DBP was -5.5 mmHg in the Test and -5.0 mmHg in the Reference group.

The incidence of abnormal gynaecological, cervical cytology, TVUS examination and physical examination findings assessed as clinically significant was low.

The data of this trial did not show a clinically meaningful effect in the

Name of Sponsor: Laboratorios León Farma S.A.	Individual Trial Table Referring to Part of the Dossier	*(For National Authority Use only)*
Name of finished product:	Volume:	
Name of active ingredient: Drospirenone	Page:	

Test group on the QTcF interval as well as the other ECG intervals (heart rate, QRS and PR intervals) alone or compared to the Reference group.

Tolerability

The tolerability analyses were focused on bleeding pattern changes. The proportion of subjects with bleeding and spotting decreased from 69.7 % in Cycle 2 to 56.3 % in Cycle 9 in the Test and from 74.0% to 45.3% in the Reference group; the overall median number of bleeding and spotting days decreased from 10 days (first reference period: Cycles 2 to 4) to 6 days (last reference period: Cycles 7 to 9) in the Test and from 12 to 7 days in the Reference group. Among these spotting days prevailed.

The proportion of subjects with unscheduled bleeding/spotting during Cycles 2-6 was lower in the Test than in the Reference group (73.0% vs. 88.4%), with the difference (95% CI) of -15.39% (-21.78%; -8.99%) between the groups. During Cycles 2-6, the Test treatment was superior to the Reference treatment with regard to the proportion of subjects with unscheduled bleeding.

The highest proportion of subjects with unscheduled bleeding or spotting was observed in Cycle 2: 51.4% of the Test and 74.0% of the Reference group subjects. The incidence of unscheduled bleeding decreased over time in both groups, to 43.9% of the Test and 45.3% of the Reference group subjects in Cycle 9. In each cycle up to Cycle 7, the proportion of subjects with unscheduled bleeding was statistically significantly lower in the Test group than in the Reference group.

The mean [SD] number of unscheduled bleeding and spotting days during Cycles 2-9 was statistically significantly lower in the Test than in the Reference group (21.5 [22.86] days vs. 34.7 [33.73] days, p = 0.0003, Wilcoxon-rank-sum-test). The mean number of days with unscheduled bleeding and spotting decreased over time and was lower in the Test than in the Reference group in each reference period and the difference was statistically significant.

From Cycle 2 to Cycle 9 the proportion of subjects who had no bleeding or spotting increased from 30.3% to 43.7% in the Test and from 26.0% to 54.7% in the Reference group.

The percentage of subjects with frequent bleeding gradually decreased

Name of Sponsor: Laboratorios León Farma S.A.	Individual Trial Table Referring to Part of the Dossier	*(For National Authority Use only)*
Name of finished product:	Volume:	
Name of active ingredient: Drospirenone	Page:	

	over time from 9.1% during Cycles 2-4 to 5.3% during Cycles 7-9 in the Test group and from 7.2% to 4.4% in the Reference group and was comparable between the treatment groups in each reference period. The percentage of subjects who experienced prolonged bleeding decreased from 12.1% during Cycles 2-4 to 2.9% during Cycles 7-9 in the Test group and from 16.7% to 10.9% in the Reference group. The incidence of prolonged bleeding in each reference period was lower in the Test than in the Reference group, with statistically significant differences between the groups in the second and in the third reference period.<br><br>The median number of unscheduled bleeding/spotting episodes in the Test group was 1.0 episode in each reference period, and that in the Reference group was 3.0 episodes in the first and 1.0 episode in the second and the third reference periods. The difference between the mean [SD] numbers of unscheduled episodes was statistically significant only in Cycles 2-4 (1.7 [1.55] episodes in the Test vs. 2.6 [1.92] episodes in the Reference group; $p < 0.0001$, Wilcoxon-rank-sum-test) and not significant in Cycles 5-7 and Cycles 7-9.<br><br>With regard to bleeding, 46 (5.4%) Test group and 31 (9.3%) Reference subjects experienced vaginal (or uterine) bleeding-related TEAEs, 28 (3.3%) Test group subjects and 22 (6.6%) Reference group subjects discontinued prematurely due to TEAEs, and four (0.5 %) Test group and three (0.9%) Reference group subjects had severe TEAEs.
Conclusion:	The results of this trial show that the use of drospirenone 4.0 mg in a regimen 24 verum / 4 placebo over 9 treatment cycles provided effective contraceptive protection with an acceptable bleeding pattern. In particular, the treatment with drospirenone 4.0 mg in a regimen 24 verum / 4 placebo resulted in a lower frequency of unscheduled bleeding (i.e. provided better cycle control) and less prolonged bleeding than the treatment with desogestrel 0.075 mg in a 28/0 regimen. During Cycles 2-6, the treatment with drospirenone 4.0 mg in a regimen 24 verum / 4 placebo was superior to the treatment with desogestrel 0.075 mg in a 28/0 regimen with regard to the proportion of subjects with unscheduled bleeding. No major differences between the treatment groups were observed for safety analyses.<br><br>The data from two 9-cycle to 13-cycle trials showed that LF111 is an

Name of Sponsor: Laboratorios León Farma S.A.	Individual Trial Table Referring to Part of the Dossier	*(For National Authority Use only)*
Name of finished product:	Volume:	
Name of active ingredient: Drospirenone	Page:	

	effective oral contraceptive with an overall Pearl Index (95% CI) of 0.7258 (0.3133; 1.4301).
Date of the report:	10-JUL-2014

3. TABLE OF CONTENTS

1. APPROVAL SIGNATURES / LIST OF AUTHORS 2
1.1 Sponsor Approval Signatures 2
1.2 Coordinating Investigator Approval Signature 3
2. SYNOPSIS 4
3. TABLE OF CONTENTS 18
3.1 LIST OF TABLES 22
3.2 LIST OF FIGURES 25
4. LIST OF ABBREVIATIONS AND DEFINITION OF TERMS 27
4.1 List of Abbreviations 27
4.2 Definition of Terms 28
5. ETHICS 30
5.1 Independent Ethics Committees (IECs) 30
5.2 Ethical Conduct of the Trial 30
5.3 Subject Information and Consent 30
6. INVESTIGATORS AND TRIAL ADMINISTRATIVE STRUCTURE 31
7. INTRODUCTION 33
7.1 Background 33
7.2 Investigational Medicinal Product 33
7.3 Rationale 33
8. TRIAL OBJECTIVES 35
9. INVESTIGATIONAL PLAN 36
9.1 Overall Trial Design and Plan Description 36
9.2 Discussion of Trial Design, Including the Choice of Control Groups 38
9.3 Selection of Trial Population 39
9.3.1 Inclusion criteria 39
9.3.2 Exclusion criteria 39
9.3.3 Removal of subjects from therapy or assessment 41
9.4 Treatments 41
9.4.1 Treatments administered 41
9.4.2 Method of assigning subjects to treatment groups 43
9.4.3 Selection and timing of dose for each subject 43
9.4.4 Blinding 44
9.4.5 Prior and concomitant therapy 45
9.4.6 Treatment compliance 46

9.5 Efficacy, Safety and Tolerability Variables ... 46
9.5.1 Efficacy and safety measurements ... 46
9.5.1.1 Efficacy assessment criteria ... 46
9.5.1.2 Safety and tolerability assessment criteria ... 47
9.5.2 Appropriateness of measurements ... 54
9.5.3 Primary efficacy variable ... 54
9.5.4 Drug concentration measurements ... 54
9.6 Data Quality Assurance ... 54
9.7 Statistical Methods Planned in the Protocol and Determination of Sample Size ... 56
9.7.1 Statistical and analytical plan ... 56
9.7.1.1 Analysis sets ... 56
9.7.1.2 Statistical methods ... 57
9.7.2 Determination of sample size ... 61
9.8 Changes in the Conduct of the Trial or Planned Analyses ... 62
9.8.1 Protocol amendments ... 62
9.8.2 Changes to analyses described in the protocol ... 63
10. TRIAL SUBJECTS ... 65
10.1 Disposition of Subjects ... 65
10.2 Protocol Deviations ... 67
11. EFFICACY EVALUATION ... 69
11.1 Data Sets Analysed ... 69
11.2 Demographic and Other Baseline Characteristics ... 69
11.3 Medical History ... 72
11.4 Gynaecological, Obstetric and Menstrual History ... 75
11.5 Prior medications, including contraceptive methods ... 78
11.6 Concomitant medications ... 79
11.7 Measurements of Treatment Compliance ... 81
11.8 Efficacy Results and Tabulations of Individual Subject Data ... 82
11.8.1 Analysis of efficacy ... 82
11.8.1.1 Overall Pearl Index ... 82
11.8.1.2 Overall Pearl Index for DRSP users in pooled CF111/301 and CF111/302 trials ... 88
11.8.2 Statistical/analytical issues ... 90
11.8.2.1 Adjustment for covariates ... 90
11.8.2.2 Handling of drop-outs or missing data ... 90

11.8.2.3 Interim analyses and data monitoring 90
11.8.2.4 Multicentre studies 90
11.8.2.5 Multiple comparisons/multiplicity 90
11.8.2.6 Use of an “efficacy subset” of subjects 91
11.8.2.7 Active-control studies intended to show equivalence 91
11.8.2.8 Examination of subgroups 91
11.8.3 Tabulation of individual response data 91
11.8.4 Drug dose, drug concentration and relationships to response 91
11.8.5 Drug-drug and drug-disease interactions 91
11.8.6 By-subject displays 91
11.8.7 Efficacy conclusions 92
12. SAFETY EVALUATION 95
12.1 Extent of Exposure 95
12.2 Adverse Events (AEs) 95
12.2.1 Brief summary of adverse events 95
12.2.1.1 Summary of adverse events by subgroups 96
12.2.2 Display of adverse events 98
12.2.3 Analysis of adverse events 100
12.2.4 Listing of adverse events by subject 102
12.3 Deaths, Other Serious Adverse Events and Other Significant Adverse Events 102
12.4 Pregnancies 105
12.5 Clinical Laboratory Evaluation 106
12.5.1 Listing of individual laboratory measurements by subject and each abnormal laboratory value 106
12.5.2 Evaluation of each laboratory parameter 106
12.5.2.1 **Laboratory values over time and individual subject changes** 107
12.5.2.2 Individual clinically significant abnormalities 114
12.6 Vital Signs, Physical Findings and Other Observations Related to Safety 115
12.6.1 Vital signs 115
12.6.1.1 Body weight and body mass index 115
12.6.1.2 Body weight and body mass index by subgroups 117
12.6.1.3 Blood pressure and heart rate 119
12.6.1.4 Blood pressure and heart rate by subgroups 122
12.6.2 Gynaecological examination 124
12.6.3 Cervical smear examination 125
12.6.4 Intravaginal ultrasound examination 125

12.6.5 Physical examination 125
12.6.6 12-lead ECG 126
12.7 Tolerability 129
12.7.1 Number of subjects with bleeding or spotting 129
12.7.2 Number of subjects with unscheduled bleeding or spotting 133
12.7.3 Number of subjects who had no bleeding or spotting 135
12.7.4 Numbers of subjects by bleeding intensity 136
12.7.5 Subjects with infrequent, frequent and prolonged bleeding 137
12.7.6 Number of bleeding or spotting days 138
12.7.7 Number of unscheduled bleeding or spotting days 142
12.7.8 Number of bleeding days by intensity 147
12.7.9 Number of bleeding or spotting episodes 148
12.7.10 Incidence of TEAEs based on abnormal vaginal (or uterine) bleeding 150
12.7.11 Return of fertility 150
12.8 Safety and Tolerability Conclusions 150
13. DISCUSSION AND OVERALL CONCLUSION 154
13.1 Discussion 154
13.2 Overall Conclusion 155
14. REFERENCE LIST 157

3.1 LIST OF TABLES

Table 1: Investigation Schedule 37
Table 2: Details on Test product, LF111 42
Table 3: Details on Test Placebo 42
Table 4: Details on the Reference Product (Desogestrel) 42
Table 5: Details on Reference Placebo 42
Table 6: Safety laboratory parameters 52
Table 7: Subject Disposition (Randomised Set) 66
Table 8: Subjects Disposition by Attended Visit (FAS, SS) 67
Table 9: Deviations Leading to Exclusion from the Method Failure Analysis (FAS) 68
Table 10: Analysis Sets (Randomised Set) 69
Table 11: Demographics (SS, FAS) 70
Table 12: Substance Use at Screening (SS, FAS) 71
Table 13: Baseline Characteristics at Screening (FAS, SS) 72
Table 14: Prior Medical History Findings, Frequency ≥ 2.0% of Subjects in any Treatment Group (FAS, SS) 73
Table 15: Ongoing Medical Findings, Frequency ≥ 2.0% of Subjects in any Treatment Group (FAS, SS) 74
Table 16: VTE Risk Factors (FAS, SS) 74
Table 17: Gynaecological, Obstetric and Menstrual History (FAS, SS) 75
Table 18: Scheduled/Regular Bleeding Characteristics Before Screening (Randomised Set) 76
Table 19: Dysmenorrhoea Characteristics at Screening and at Follow-up (FAS, SS) 77
Table 20: Mastodynia/Mastalgia Characteristics at Screening and at Follow-up (FAS, SS) 78
Table 21: Prior Medications, Therapies, Contraceptives by ATC 2nd Level, Frequency ≥ 2.0% of Subjects in any Treatment Group (FAS, SS) 79
Table 22: Prior Medications, Therapies, Contraceptives by Generic Name WHO DD, Frequency ≥ 2.0% of Subjects in any Group (FAS, SS) 79
Table 23: Concomitant Medications, Therapies, Contraceptives by ATC 2nd Level, Frequency ≥ 2.0% of Subjects in any Treatment Group (FAS, SS) 80
Table 24: Concomitant Medications by Substance Name, Frequency ≥ 2.0% of Subjects (FAS, SS) 80
Table 25: Concomitant Antibiotics by Substance Name, Frequency> 0.5% of Subjects in any Treatment Group (FAS, SS) 81
Table 26: Concomitant Antibiotics Use Duration in Days (FAS, SS) 81
Table 27: Compliance (%) to Investigational Medicinal Product (FAS, SS) 82
Table 28 Exposure Cycles (FAS) 83

Table 29 Overall Pearl Index (FAS) 83
Table 30 Overall Pearl Index for Subjects ≤ 35 Years (FAS)....84
Table 31: Sexual Activity Cycles Without Additional Contraception (FAS)....84
Table 32 Overall Pearl Index After Correction for Additional Contraception and Sexual Activity Status (FAS)....85
Table 33 Overall Pearl Index After Correction for Additional Contraception and Sexual Activity Status, Age Group ≤ 35 Years (FAS) 85
Table 34: Perfect Medication Cycles (FAS) 86
Table 35 Method Failure Pearl Index, (FAS) 86
Table 36 Method Failure Pearl Index, Age Group ≤ 35 Years (FAS) 87
Table 37: Overall Pregnancy Ratio (FAS) 87
Table 38: Overall Pregnancy Ratio, Age Group ≤ 35 Years (FAS)....88
Table 39: Overall Pearl Index for Pooled CF111/301 and CF111/302 Trials (FAS) 88
Table 40: Overall Pearl Index after Correction and Sexual Activity Status in Pooled CF111/301 and CF111/302 Trials (FAS) 89
Table 41: Method Failure Pearl Index in Pooled CF111/301 and CF111/302 Trials (FAS)....89
Table 42: Overall Pregnancy Ratio for Pooled CF111/301 and CF111/302 Trials (FAS) 90
Table 43: Exposure to Investigational Medicinal Product [Days] (SS, FAS)....95
Table 44: Summary of Subjects with Treatment Emergent Adverse Events (SS)....96
Table 45: Summary of Subjects with TEAEs by Age Group (SS) 97
Table 46: Summary of Subjects with TEAEs by BMI Group (SS) 97
Table 47: Summary of Subjects with TEAEs by Blood Pressure Group (SS)....98
Table 48: Incidence of TEAEs by Primary System Organ Class, Frequency of Subjects with TEAEs ≥ 2.0% in any Treatment Group (SS)....98
Table 49: Incidence of TEAEs by Preferred Term, Frequency of Subjects with TEAEs ≥ 2.0% in any Treatment Group (SS) 99
Table 50: Incidence of Most Frequent TEAEs by Age Subgroup (SS) 99
Table 51: Incidence of Most Frequent TEAEs by BMI Subgroup (SS) 100
Table 52: Incidence of Most Frequent TEAEs by Blood Pressure Subgroup (SS)....100
Table 53: Incidence of TEAEs Assessed as Related, Which Occurred in > 1 Subject of any Treatment Group (SS)....101
Table 54: TEAEs by Severity (SS) 102
Table 55: Incidence of Serious Adverse Events (SS) 103
Table 56: Incidence of TEAEs Leading to Premature Discontinuation (SS)....105
Table 57: Pregnancies (SS) 106

Table 58: Summary of Haematology Variables with Statistically Significant Differences Between the Treatment Groups (SS) 107
Table 59: Shifts of Haematology Parameters from Baseline to Endpoint in >5.0% of Subjects of Any Group (SS) 108
Table 60: Summary of Cholesterol and Triglycerides under Fasting Conditions (SS) 109
Table 61: Biochemistry Variables with Individual Subject Changes in More than 5.0% of Subjects (SS) 110
Table 62: Summary of Haemostatic Parameters with Statistically Significant Differences Between the Groups (SS) 111
Table 63: Changes of Haemostatic Parameters from Baseline to Endpoint (SS) 112
Table 64: Shifts of Carbohydrate Metabolism Parameters from Baseline to Endpoint (SS) 112
Table 65: Summary of Abnormal Urinalysis Values (SS) 114
Table 66: TEAEs Based on Individual Clinically Significant Laboratory Abnormalities / MedDRA Coding (SS) 115
Table 67: Summary of Body Weight and BMI (SS) 116
Table 68: Incidence of TEAEs Based on Weight Changes (SS) 117
Table 69: Body Weight and BMI Changes from Baseline to Endpoint by Age Subgroups (SS) 117
Table 70: Body Weight and BMI Changes from Baseline to Endpoint by BMI Subgroups (SS) 118
Table 71: Body Weight and BMI Changes from Baseline to Endpoint by Blood Pressure Subgroups (SS) 119
Table 72: Blood Pressure (SS) 120
Table 73: Summary of Heart Rate (bpm) (SS) 121
Table 74: Blood Pressure and Heart Rate Changes from Baseline to Endpoint by Age Subgroups (SS) 122
Table 75: Blood Pressure and Heart Rate Changes from Baseline to Endpoint by BMI Subgroups (SS) 123
Table 76: Blood Pressure and Heart Rate Changes from Baseline to Endpoint by Blood Pressure Subgroups (SS) 124
Table 77: Summary of Cervical Smear Examination (SS) 125
Table 78: Frequency of Abnormal Physical Examination Findings Assessed as Clinically Significant (SS) 126
Table 79: Summary of ECG Parameters (SS) 127
Table 80: Interpretation of ECG Parameters (SS) 129
Table 81: Number of Subjects with Bleeding or Spotting by Treatment Cycle and Period (FAS) 131
Table 82: Number of Subjects with Bleeding by Treatment Cycle and Period (FAS) 132

Table 83: Number of Subjects with Spotting by Treatment Cycle and Period (FAS) 133
Table 84: Number of Subjects with Unscheduled Bleeding or Spotting by Treatment Cycle and Period (FAS) 133
Table 85: Number of Subjects with Unscheduled Bleeding by Treatment Cycle and Period (FAS) 134
Table 86: Number of Subjects with Unscheduled Spotting by Treatment Cycle and Period (FAS) 135
Table 87: Number of Subjects with no Bleeding or Spotting by Treatment Cycle and Period (FAS) 136
Table 88: Number of Subjects with Slight Bleeding by Treatment Period (FAS) 137
Table 89: Number of Subjects with Moderate Bleeding by Treatment Period (FAS) 137
Table 90: Number of Subjects with Heavy Bleeding by Treatment Period (FAS) 137
Table 91: Number of Subjects with Infrequent Bleeding per Reference Period (FAS) 138
Table 92: Number of Subjects with Frequent Bleeding per Reference Period (FAS) 138
Table 93: Number of Subjects with Prolonged Bleeding per Reference Period (FAS) 138
Table 94: Number of Days with Bleeding and/or Spotting by Treatment Period (FAS) 140
Table 95: Number of Days with Bleeding by Treatment Period (FAS) 141
Table 96: Number of Days with Spotting by Treatment Period (FAS) 142
Table 97: Number of Days with Unscheduled Bleeding and/or Spotting by Treatment Cycle (FAS) 143
Table 98: Number of Days with Unscheduled Bleeding and/or Spotting by Treatment Period (FAS) 145
Table 99: Number of Days with Unscheduled Bleeding by Treatment Period (FAS) 146
Table 100: Number of Days with Unscheduled Spotting by Treatment Period (FAS) 147
Table 101: Number of Days with Slight, Moderate and Heavy Bleeding by Treatment Period (FAS) 148
Table 102: Number of Bleeding and/or Spotting Episodes by Reference Period (FAS) 149
Table 103: Number of Unscheduled Bleeding and/or Spotting Episodes by Reference Period (FAS) 149
Table 104: Summary of Subjects with TEAEs Based on Abnormal Bleeding (SS) 150

3.2 LIST OF FIGURES

Figure 1: Flow Chart of Subjects Disposition 65
Figure 2: Number of Subjects with Bleeding orSpotting by Treatment Day (SS) 130
Figure 3: Number of Subjects with Bleeding/Spotting by Reference Period (FAS) 131
Figure 4: Mean Number of Bleeding or Spotting Days per Cycle 139

Figure 5: Mean Number of Unscheduled Bleeding or Spotting Days per Reference Period (FAS) 144

4. LIST OF ABBREVIATIONS AND DEFINITION OF TERMS

4.1 List of Abbreviations

ABBREVIATION	EXPLANATION
AE	Adverse event
ADR	Adverse drug reaction
ALAT	Alanine aminotransferase
ALP	Alkaline phosphatase
APC	Activated protein C
ASAT	Aspartate aminotransferase
ß-hCG	Beta human chorionic gonadotropin
BMI	Body mass index
bpm	Beats per minute
BUN	Blood urea nitrogen
CI	Confidence interval
COCP	Combined-oral-contraceptive pill
CPK	Creatine phosphokinase
CRA	Clinical Research Associate
CRF	Case report form
CRO	Contract Research Organisation
CS	Clinically significant
CTX	Cross-linked c-terminal telopeptides
DBP	Diastolic blood pressure
DRSP	Drospirenone
ECG	12-lead electrocardiogram
e-diary	Electronic diary
EE	Ethinyl estradiol
E2	17β-estradiol
FAS	Full Analysis Set
Gamma-GT	Gamma-glutamyl transferase
GCP	Good Clinical Practice
HDL cholesterol	High-density lipoproteins cholesterol
ICH	International Conference on Harmonisation
IEC	Independent Ethics Committee
IMP	Investigational medicinal product
IUD	Intrauterine device
LDH	Lactate dehydrogenase
LDL cholesterol	Low-density lipoproteins cholesterol
MCH	Mean corpuscular haemoglobin
MCV	Mean corpuscular volume
MedDRA	Medical Dictionary for Regulatory Activities
PAP smear	Papanicolaou smear
PI	Pearl Index

ABBREVIATION	EXPLANATION
POP	Progestogen-only-pill
PT	Preferred term
SAE	Serious adverse event
SAP	Statistical Analysis Plan
SAS	Statistical Analysis Software
SBP	Systolic blood pressure
SD	Standard deviation
SS	Safety Set
SOC	System Organ Class (MedDRA)
SUSAR	Suspected unexpected serious adverse reaction
TEAE	Treatment emergent adverse event
TESAE	Treatment emergent serious adverse event
TSH	Thyroid-stimulating hormone
VTE	Venous thromboembolism
WHO DDE	World Health Organization Drug Dictionary Enhanced

4.2 Definition of Terms

Subjects

**Starter:**	First administration of a hormonal contraceptive or at least 4 month break after the administration of another hormonal contraceptive
**Switcher:**	Direct switch from another hormonal contraceptive to the IMPs with no break in administration

Vaginal Bleeding

**Bleeding**	Evidence of blood loss that required the use of sanitary protection with a tampon, pad or panty liner.
**Spotting**	Evidence of minimal blood loss that does not require new use of any type of sanitary protection, including panty liners.
**Episode of bleeding/spotting**	Bleeding/spotting days bounded on either end by two days of no bleeding or spotting.
**Scheduled bleeding day**	Any bleeding or spotting that occurs during the hormone-free intervals (defined as Days 25 – 28 +/-1). Up to 8 consecutive bleeding/spotting days are considered as scheduled bleeding days. This definition is applicable only to subjects who received drospirenone tablets.
**Unscheduled bleeding/spotting day**	Any bleeding or spotting that occurs while taking active hormones (Days 2 – 23), except days which are classified as scheduled bleeding days. This definition is applicable

	only to subjects who received drospirenone tablets. In the desogestrel treatment group all bleeding days are classified as unscheduled.
<u>**Contraceptives**</u>	
**Barrier contraception method**	Contraceptive measure that prevents pregnancy by physically preventing sperm from entering the uterus, i.e. condom, female condom, cervical cap, diaphragm or contraceptive sponge.
**Spermicide**	Contraceptive substance that eradicates sperm, inserted vaginally prior to intercourse to prevent pregnancy. (Usually, spermicides are combined with contraceptive barrier methods.)
**Intrauterine device (IUD)**	Device that is placed in the uterus to prevent pregnancy
**Emergency contraception**	Postcoital contraception ("morning-after pill")

5. ETHICS

5.1 Independent Ethics Committees (IECs)

The trial protocol, protocol amendments, including the informed consent form and the subject information sheet in the official languages of each country, were submitted to the relevant IECs/IRBs in the different countries in accordance with local laws and regulations. Written approvals of the IEC's were obtained before the start of the trial. A list of ethics committees is provided in Appendix 16.1.3.

5.2 Ethical Conduct of the Trial

The trial was conducted in accordance with the Declaration of Helsinki (1996), the International Conference on Harmonisation (ICH) Guideline for Good Clinical Practice (GCP), with the Commission Directives 2001/20/EC and 2005/28/ and the local laws of the countries where the trial was performed.

5.3 Subject Information and Consent

The investigators were to explain to each subject the nature of the trial, its purpose, the procedures involved and the potential risks and benefits of participation in the trial. Written informed consent was to be received from all subjects prior to the conduct of any trial-related procedures. Collection of informed consent was to be documented on the case report form (CRF). A blank CRF can be found in Appendix 16.1.2. A sample subject information sheet and informed consent form in English are provided in Appendix 16.1.3.

6. INVESTIGATORS AND TRIAL ADMINISTRATIVE STRUCTURE

Sponsor

Laboratorios León Farma S.A.
La Vallina s/n
Polígono Industrial de Navatejera
24008 Navatejera (León), Spain
Phone: +34 619 275 590
Fax: +34 197 668 963
Email: Leonfarma@chemogroup.com

Sponsor Legal Representative

Project leader of the trial: Dominique Drouin,
Directeur du Développement "Endocrinologie & Gynécologie"

CHEMO France
7 rue Victor Hugo
92310 Sèvres, France
Phone: +33 1 49 66 22 26
Fax: +33 1 41 14 99 17
E-mail: dominique.drouin@chemofrance.com

Contract research organisation (CRO)

Scope International AG
Konrad-Zuse-Ring 18
68163 Mannheim, Germany
Phone: +49 621 429 39 0
Fax: +49 621 429 39 40

Project manager:	Bernd Hassler PhD
Trial biostatistician:	Grazina Binkauskiene MSc
Clinical trial report author:	Ramune Pusliene MSc

Coordinating Investigator:
Dr. Santiago Palacios
Instituto Palacios
Calle Antonio Acuña, 9
28009 Madrid, Spain
Phone: +34 91 578 24 96
Fax: + 34 91 431 99 51

The trial was conducted in 73 centres located in Austria, Czech Republic, Germany, Hungary, Poland, Romania, Slovakia and Spain. A list of all sites, investigators, as well as their addresses, contact information and curricula vitae of the investigators are given in Appendix 16.1.4.

E-Diary Provider

PHT Corporation Sàrl
2, chemin Louis-Hubert
1213 Petit-Lancy, Geneva, Switzerland
Phone: +41 22 879 91 00
Fax: +41 22 879 91 01

Laboratory

Haematological (including differential blood count) and biochemical laboratory assessments as well as serum pregnancy tests were performed by:

Laboratorium für Klinische Forschung GmbH
Lise-Meitner-Str. 25-29
24223 Schwentinental, Germany
Phone: +49 4307 8276 0
Fax: +49 4307 8276 79
E-Mail: project@lkf-kiel.de

The cervical cytology assessments were done centrally by an external pathologist:

Priv.-Doz. Dr. Med. Lutz Riethorf
Labor für Klinishe und Molekulare Pathologie
Grandweg 64
22529 Hamburg, Germany
Phone: +49 54809070
Fax: 49 54809071
E-Mail: riethdorf@uni-hamburg.de

Central ECG Assessment

All ECGs were centrally evaluated by:

eResearchTechnology Limited (ERT)
Pegasus House
Bakewell Road
Orton Southgate
Peterborough PE2 6YS, United Kingdom
Phone: +44 1733 374851
Fax: +44 1733 239789

7. INTRODUCTION

Oral contraceptives are among the most popular forms of contraception. They can be divided into combined-oral-contraceptive pills (COCPs, combination of an estrogen and a progestogen) and progestogen-only pills (POPs).

In comparison to COCPs, POPs offer several advantages: they are associated with a decreased venous thromboembolism (VTE) risk and cause fewer metabolic changes (no effects on blood pressure or cholesterol level, only small effects on clotting mechanism and glucose metabolism). This makes them a suitable option for women who are intolerant to or contraindicated for estrogens (due to migraine or cardiovascular risk factors such as hypertension, hyperlipidaemias, obesity, diabetes, smoking habits etc.). POPs can also reduce the symptoms of premenstrual syndrome and painful periods.

7.1 Background

Drospirenone (DRSP) is a fourth generation progestogen, which is derived from spironolactone. DRSP has a pharmacological profile similar to natural progesterone and possesses anti-mineralocorticoid and anti-androgenic activity.

In combination with ethinyl estradiol (EE) and 17β-estradiol (E2), DRSP has been extensively studied in the preclinical and clinical setting. DRSP 3 mg in combination with EE 30 µg or 20 µg, from 21 days to 24 days, is registered for use in the prevention of pregnancy as an oral contraceptive, (e.g. Yasmin®, Yasminelle®, YAZ®). In addition, DRSP is registered for use in combination with E2 1 mg as Angeliq® [1] (in Europe at 2 mg and in the USA at 0.5 mg) as hormone replacement therapy for estrogen deficiency symptoms in postmenopausal women and the prevention of postmenopausal osteoporosis in women who are intolerant to or contraindicated for other medicinal products approved for the prevention of osteoporosis.

Leon Farma has developed a new formulation with 4 mg of DRSP (LF111). Ovarian suppression with LF111 was demonstrated in two phase II studies (CF111/202 and CF111/203).

The contraceptive efficacy of LF111 was demonstrated in the uncontrolled Phase III study CF111/301 over 13 medication cycles.

It was planned that this controlled, CF111/302 Phase III study, will provide around 6500 cycles to be pooled with the cycles from the CF111/301 study for the calculation of PI to fulfil the requirements of EMA. [5]

7.2 Investigational Medicinal Product

The test product was LF111. One LF111 package includes 24 DRSP 4 mg tablets, followed by 4 placebo tablets. For details, please refer to the Drospirenone POP- LF111 Investigator's Brochure (IB) [4].

The reference product was desogestrel 0.075 mg (Cerazette®) tablets. One package contains 28 active tablets.

7.3 Rationale

Due to the cardiovascular risk associated with EE in COCPs, there is an increasing, unmet need for POPs. In addition, POPs that consistently inhibit ovulation and therefore allow for a time window of at least 12 hours of delay in pill intake, are more convenient than conventional POPs.

Concerning its primary objective (demonstrating contraceptive efficacy), the current study was connected to the Phase III CF111/301 study. The pooled data of both studies were

analysed together to fulfil the precision requirements of the Guideline on Clinical Investigation of Steroid Contraceptives [5] for the calculation of the Pearl Index (PI).

The current study intended to demonstrate that LF111 (i.e. a regimen of 24 DRSP 4 mg tablets followed by 4 placebo tablets) shows reliable contraceptive efficacy and is also well tolerated and safe compared to desogestrel 0.075 mg, especially regarding bleeding pattern.

8. TRIAL OBJECTIVES

Primary objective

- To demonstrate the contraceptive efficacy of LF111.

Secondary objective

- To demonstrate the safety and tolerability of LF111 in comparison to desogestrel 0.075 mg, especially regarding bleeding pattern.

9. INVESTIGATIONAL PLAN

9.1 Overall Trial Design and Plan Description

Copies of the clinical trial protocol and amendments are provided in Appendix 16.1.1. A sample case report form (CRF) is provided in Appendix 16.1.2.

This was a Phase III prospective, multicentre5:2 randomised, double-blind, double-dummy, active control, parallel-group trial conducted in 73 centres.

After providing informed consent at Visit 1a (screening) and receiving study medication at Visit 1b, eligible subjects were to attend Visits 2 to 4 at Day 24±2 of the 1st, 3rd and 6th cycle, and Visit 5 at Day 29+2 of the 9th treatment cycle. The interval between Visit 1a and Visit 1b should not exceed 30 days. The follow-up (Visit 6) was to take place 7 to 10 days after the last IMP intake.

In Germany, the study design was amended to reschedule Visit 6 to 10-28 days after the last IMP intake as requested by the Ethics Committee of the State of Berlin, see Section 9.8.1.

Subjects who met the selection criteria were randomised in 5:2 ratio at Visit 1b to double- blind and double-dummy treatment with either Test: DRSP 4.0 mg for 24 days followed by placebo for 4 days + placebo of desogestrel 0.075mg or Reference: desogestrel 0.075 mg for 28 days + placebo of Test for nine cycles.

The investigation schedule is presented in Table 1.

Table 1: Investigation Schedule

Visits	V1a (Screening)	V1b (Randomisation)[g]	V2	V3	V4	V5 / EDV	V6 (Follow-Up)[i]
**Medication cycle**			**1**	**3**	**6**	**9[a]**	**7-10 days after last IMP intake[b]**
			**Day 24±2 of medication cycle**			**Day 29+2**	
Informed consent	x						
Demography	x						
Medical and gynaecological history[c]	x						
Prior medication/ contraceptive devices	x						
Concomitant medication/ contraceptive devices	x		x	x	x	x	
Physical examination	x					x	
Physical examination (for Germany only)	x[h]	x[h]	x[h]	x[h]	x[h]	x[h]	x[h]
Vital signs[d], body weight	x		x	x	x	x	
ECG for a subset of subjects		x				x	
Gynaecological examination	x					x	
Intravaginal ultrasound	x					x	
Cervical cytology	x					x	
Routine laboratory parameters[e]	x			x		x	
Special laboratory parameters for subset of subjects[e]		x				x	
Laboratory test for electrolytes					x		
Urinalysis[f]	x			x		x	
Serum pregnancy test	x					x	
Urine pregnancy test			x	x	x		x
In-/exclusion criteria	x	x					
Randomisation		x					
Dispensing of IMPs and home pregnancy test kit		x	x	x	x		
Drug accountability			x	x	x	x	
Dispense/collect e-diary		x				x	
Review e-diary			x	x	x	x	
Adverse events		x	x	x	x	x	x

(a) Assessments were to be performed after completion of cycle 9 and also in case of discontinuation.

(b) The post treatment evaluation was to be performed by interviewing the subjects. Inquiries were to be made regarding menstrual cycle, possible return of fertility, and possible use of contraceptive. For Germany Visit 6 (follow-up) was to take place 10 – 28 days after the last IMP intake (Protocol Amendment No.1 Germany).

(c) Including check of venous thromboembolism (VTE) risk

(d) Blood pressure, pulse and (only at V1a) height

(e) Laboratory parameters: haematology, biochemistry, TSH.
Haematology: haemoglobin, red blood cell count, mean corpuscular volume (MCV) and associated parameters, haematocrit, MCH, white blood cell count, differential white blood cell count including

neutrophils, lymphocytes, eosinophils, basophils and monocytes, platelet count.
Biochemistry: sodium, potassium, chloride, creatinine, blood urea nitrogen (BUN), calcium, glucose, total proteins, albumin, total cholesterol (HDL, LDL cholesterol), triglycerides, gamma glutamyl transferase, total and direct bilirubin, alkaline phosphatase (ALP), alanine aminotransferase (ALAT), aspartate aminotransferase (ASAT), creatine phosphokinase (CPK), lactate dehydrogenase (LDH).

Special clinical laboratory parameters (haemostatic variables, carbohydrate metabolism and bone metabolism) for a subset of subjects (at least 40 per treatment group):
Haemostatic variables: factor VIIc, factor VIIIc, protein C activity, antithrombin III activity, D-dimer. Resistance to activated protein C (APC) variable was added by the Protocol Amendment No.1 Austria and Protocol Amendment No.2 Germany.
Carbohydrate metabolism: fasting glucose, serum insulin, C-peptide
Bone metabolism: bone alkaline phosphatase, cross-linked c-terminal telopeptides (CTX)phosphatase, cross-linked c-terminal telopeptides (CTX)

(f) Urinalysis: leukocytes, nitrite, protein, glucose, ketones, blood, pH, urobilinogen, bilirubin, haemoglobin – dipstick. If any of the measured parameters of urine analysis was out of range/pathologic, sample was to be shipped to the central laboratory for microscopic examination.

(g) The interval between visits V1a and V1b should not exceed 30 days.

(h) With particular attention to lower extremities (Protocol Amendment No.1 Germany).

9.2 Discussion of Trial Design, Including the Choice of Control Groups

The trial design and choice of control group were based on the Committee for Proprietary Medicinal Products Guideline on Clinical Investigations of Steroid Contraceptives in Women [5]. According to this Guideline, for a new contraceptive method, a sufficient number of cycles should be studied to obtain the desired precision of the estimate of contraceptive efficacy. In addition, it is stated that data for the calculation of the overall Pearl Index (PI) may emanate from more than one study. The LF111 clinical development programme will allow for calculation of the overall PI by pooling data from two Phase III studies: the completed uncontrolled study CF111/301, investigating contraceptive efficacy over 13 cycles, and the current, double-blind, randomised efficacy study CF111/302 in comparison to Cerazette®.

For an assumed PI<1.0, 12337 of cycles were needed to fulfil this precision requirement with a 90% power.Thus 6169 cycles were to be collected in each of the two studies. It was estimated that this would require 685 evaluable subjects with a treatment duration of nine cycles in the current study.

According to the Guideline, active controlled studies should be performed to assess the adverse events, including vaginal bleeding events, and the comparator should, whenever possible, be chosen among market leading products with a similar mechanism of action and schedule of use. Desogestrel 0.075 mg (in a regimen of 28 active pills, marketed under trade names such as Cerazette® and Cerazet®) was chosen as the comparator in this study, because it is more effective at preventing ovulation than other POPs [6] and has been shown to inhibit ovulation in over 90% of cycles with a Pearl Index (PI) similar to the low-dose COCs [7]. It is also the first POP with a delayed pill intake window of 12 hours, instead of 3 hours allowed by first generation POPs, and is one of the most commonly used POPs in the European market. Desogestrel was choosen for the comparison of the vaginal bleeding patterns and safety of the products. The sample size of subjects taking desogestrel was calculated based on tolerability endpoint, the proportion of subjects with unscheduled bleeding.

9.3 Selection of Trial Population

9.3.1 Inclusion criteria

Subjects had to meet ALL of the following criteria:

1. Woman without uncontrolled current diseases at risk of pregnancy, at the age of 18-45 years
2. For starters: At least four menstrual cycles during the last six months before Visit 1a were regular (i.e. cycle length between 24 and 35 days)
3. Systolic blood pressure < 140 mmHg, diastolic blood pressure < 90 mmHg, in a sitting position, after 5 minutes of rest
4. Subject agrees to use only IMP for contraception for at least 9 cycles
5. Menstruation restarted since last pregnancy (only applicable for women that were pregnant within the last 6 months), i.e. at least one menstrual cycle after delivery
6. Laboratory values with no deviations of any clinical relevance for the course of the study in the opinion of the investigator
7. Written informed consent given freely after the nature of the trial and disclosure of data had been explained to the subject

9.3.2 Exclusion criteria

Subjects were to be excluded for ANY ONE of the following reasons:

**Criterion**	**Rationale**
1. Pregnant subject	Safety
2. Breastfeeding subject	Safety
3. Subject is known to or suspected of not being able to comply with the study protocol and the use of the IMP	Safety, efficacy
4. Abnormal finding on pelvic, breast or ultrasound examination that precludes participation in the trial	Safety
5. Unexplained amenorrhoea, known polycystic ovary syndrome	Safety
6. Subject having ASC-US or more severe finding on Pap smear	Safety
7. Known contraindication or hypersensitivity to ingredients (drospirenone, desogestrel) or excipients of IMPs (cellulose, lactose, silicon dioxide, magnesium stearate, corn starch, polyethylene glycol, polyvinyl pyrrolidone, polyvinyl alcohol, aquariusBT16035 cottage green, talc, titanium dioxide; silica colloidal anhydrous, all-rac-α-tocopherol, lactose monohydrate, maize starch, povidone, stearic acid, hypromellose, macrogol 400)	Safety
8. Significant cardiovascular, hepatic or renal disease, diabetes with vascular involvement, uncontrolled thyroid disorder or current venous thrombosis or embolism	Safety
For Germany: Diabetes mellitus, history or presence of arterial/venous thrombosis or embolism (Protocol Amendment No.1 Germany)	Safety
9. Undiagnosed vaginal bleeding	Safety
10. Known or suspected sex-steroid sensitive malignancies	Safety
11. Presence or history of severe hepatic disease as long as liver function values have not returned to normal	Safety
12. Evidence or history of alcohol, medication or drug abuse (within the last 12 months)	Lack of suitability for the trial

13. Known bleeding disorder or history of unexplained bleeding or bruising within the last 12 months prior to V1a	Safety
14. Prohibited previous medication / contraceptives (injectable hormonal methods of contraception within the last 6 months before V1a, progestin-releasing IUD or contraceptive implant within the last 2 months before V1a, anti-retroviral therapy within the last 6 months before V1a, microsomal enzyme- inducing drugs within the last 28 days before the start of IMP intake)	Safety
15. Dependence on prohibited co-medication (estrogens, progestogens, activated charcoal, microsomal enzyme-inducing drugs, anticonvulsants [e.g. hydantoins, phenytoin, carbamazepine, oxcarbazepine, topiramate, felbamate, primidone], barbiturates, antibiotics [such as, rifabutin or rifampicin], ritonavir, nelfinavir, atorvastatin, bosentan, griseofulvin, phenylbutazon, St. John's wort [hypericum perforatum], medications that may increase serum potassium [ACE inhibitors, angiotensin – II receptor antagonists, potassium-sparing diuretics, potassium supplementation, heparin, aldosterone antagonists and NSAIDs])	Safety
16. Planned surgery during the anticipated time of participation in this trial requiring withdrawal of an oral contraceptive	Lack of suitability for the trial
17. Regular concomitant use of barrier contraceptive methods, spermicides, IUDs or other contraceptive measures (excepting occasional use due to risk of infection)	Lack of suitability for the trial
18. Evidence or history of neurotic personality, psychiatric illness or suicide risk	Safety
19. Participation in another trial of investigational drugs or devices parallel to, or less than 90 days before trial entry, or previous participation in this trial	Safety
20. Employee of the investigator or trial centre or family member of the employees or the investigator	Standard requirement
21. Any condition that, in the opinion of the investigator, may jeopardise the trial conduct according to the protocol	Standard requirement

For Germany the following two additional exclusion criteria were added by Protocol amendment No.1 Germany, requested by EC:

22. Subject concerned has been committed to an institution by virtue of an order issued either by the judicial or the administrative authorities	
23. Adrenal insufficiency, history or presence of cerebral-vascular disease, headaches with focal neurological symptoms, major surgery with prolonged immobilisation, cholestatic jaundice of pregnancy or jaundice with prior pill use	Safety

For Czech Republic the following four additional exclusion criteria were introduced by Protocol Amendment No.1 Czech Republic requested by Regulatory Authority:

22. For women with BMI ≥ 30 kg/m^2: Family history of venous thromboembolism in siblings or parents at a relatively young age	Safety

23. For women with BMI ≥ 30 kg/m^2: History of venous or arterial thrombosis (deep venous thrombosis, pulmonary embolism)	Safety
24. For women with BMI ≥ 30 kg/m^2: Smoking	Safety
25. History of migraine with focal neurological symptoms	Safety

9.3.3 Removal of subjects from therapy or assessment

Every subject had the right to refuse further participation in the trial at any time and without providing reasons and without any personal disadvantage. Subject's participation was to be terminated immediately upon her request. 'Withdrawal of consent' was to be recorded on the CRF as a reason for premature termination of the trial.

The reasons for discontinuation were to be documented in the CRF and in the subject's medical records. The project leader of the trial at León Farma was to be informed of each withdrawal and the reason for it in the monthly status report.

Subjects might withdraw or might be withdrawn from the trial for the following reasons:

- At subject's own request (withdrawal of consent)
- If in the investigator's opinion, for reasons of safety or ethics, continuation in the trial would be detrimental to the subject's well-being
- Major protocol violation
- Pregnancy
- Wish for pregnancy
- Ineligibility
- Adverse event
- At the specific request of the sponsor

For Czech Republic (Protocol Amendment No.1 Czech Republic) an additional reason for withdrawal was added:

- For women with BMI ≥ 30 kg/m^2: prolonged immobilisation.

Withdrawals were not to be replaced.

The time to return of fertility was to be followed up to a year in all subjects discontinuing treatment for wish of pregnancy. Therefore the investigator had to ask these subjects to inform him/her as soon as they would become pregnant within one year following their study participation.

9.4 Treatments

9.4.1 Treatments administered

Details on LF111 are presented in Table 2.

Table 2: Details on Test product, LF111

**Dosage Form:**	28 film-coated tablets
**Route of administration:**	Oral, once daily
**Strength:**	4.0 mg DRSP (only in 24 active tablets ─ missing in the four placebo tablets)
**Excipients:**	Lactose, cellulose, silicon dioxide, magnesium stearate, corn starch, polyethylene glycol, polyvinyl pyrrolidone, polyvinyl alcohol, aquariusBT16035 cottage green, talc, titanium dioxide.
**Presentation:**	24 white tablets and 4 green tablets
**Manufacturer:**	León Farma, S.A.
**Batch numbers:**	LFD0187A, LFD0158A, LFD0217A, LFD228A

Table 3: Details on Test Placebo

**Dosage Form:**	28 film-coated tablets
**Route of administration:**	Oral, once daily
**Strength:**	Not applicable
**Excipients:**	Lactose, cellulose, silicon dioxide, magnesium stearate, corn starch, polyethylene glycol, polyvinyl pyrrolidone, polyvinyl alcohol, aquariusBT16035 cottage green, talc, titanium dioxide.
**Presentation:**	24 white tablets and 4 green tablets
**Manufacturer:**	León Farma, S.A.
**Batch numbers:**	LFD0162A, LFD0182A, LFDO226A

Table 4: Details on the Reference Product (Desogestrel)

**Dosage Form:**	28 film-coated tablets
**Route of administration:**	Oral, once daily
**Strength:**	0.075 mg
**Excipients:**	Silica colloidal anhydrous, all-rac-α-tocopherol, lactose monohydrate, maize starch, povidone, stearic acid, hypromellose, macrogol 400, talc, titanium dioxide (E 171)
**Presentation:**	28 white tablets
**Manufacturer:**	N. V. Organon
**Batch numbers:**	LFD0180A, LFD0179A

Table 5: Details on Reference Placebo

**Dosage Form:**	28 film-coated tablets
**Route of administration:**	Oral, once daily
**Strength:**	Not applicable
**Excipients:**	Cellulose, lactose, silicon dioxide, magnesium stearate, corn starch, polyethylene glycol, polyvinyl pyrrolidone, polyvinyl alcohol, talc, titanium dioxide
**Presentation:**	28 white tablets
**Manufacturer:**	León Farma S.A.
**Batch numbers:**	LFD0161A. LFD0183A, LFD0225A, LFD0213A

A complete record of batch numbers and expiry dates of all IMPs is maintained in the trial master file.

For the IMPs a system of medication numbering in accordance with the requirements of Good Manufacturing Practice was to be used to ensure that for each subject, any dose of the trial drug can be identified and traced back to the original batches of the active ingredients.

A listing of subjects who received each batch (and certificates of the IMPs) is provided in Appendix 16.1.6.

9.4.2 Method of assigning subjects to treatment groups

Randomisation was to be performed by the Scope International AG using a validated system that automates the random assignment of treatment groups to randomisation numbers. The randomisation scheme was completed in a 5:2 ratio using blocking methodology via a centre-based randomisation method. The randomisation scheme was to be reviewed by the Data Management and Statistics Department and locked after approval.

Randomisation data were to be kept strictly confidential, accessible only to authorised persons, until the time of unblinding.

Two randomisation lists were to be prepared:

- Randomisation list for selected sites participating in the special laboratory analyses (in Germany and Austria), allocating randomisation numbers, treatment codes and flag for special laboratory analysis
- Randomisation list for all other sites, allocating randomisation numbers and treatment codes

Subjects were to be randomly assigned to the Test group or the Reference group in the ratio 5:2

- The Test group was to receive test product (DRSP 4.0 mg) in Blister A + reference placebo in Blister B.
- The Reference group was to receive test placebo in Blister A + reference product (desogestrel 0.075 mg) in Blister B.

The randomisation scheme and codes are provided in Section 16.1.7.

9.4.3 Selection and timing of dose for each subject

In this trial the IMP dose for each subject was two tablets per day. During the 28-day medication cycle the subject was to take 28 tablets from Blister A and 28 tablets from Blister B.

Each subject was to receive the medication package for the first two medication cycles at V1b and was to be given detailed instructions on the use of the IMP orally by the investigator and by the information given in the subject information sheet.

- Starters had to take the first tablet from Blister A and the first tablet from Blister B on the first day of their next menstrual bleeding. (For the definitions of starters and switchers refer to Section 4.2.)
- Switchers had to take the first tablet from Blister A and the first tablet from Blister B on the day following the last active pill of the previous hormonal contraceptive.

From Day 1 to Day 28 of the medication cycle, one tablet from Blister A and one tablet from Blister B had to be pushed out of the blister pack and swallowed whole at the same time every day. Tablets had to be taken every day at about the same time so that the interval between two tablets always was 24 hours.

The first tablet from the next Blister A and the first tablet from the next Blister B were to be taken directly on the next day without any tablet-free interval, and regardless of whether the

scheduled bleeding occurred, had stopped or was still continuing. Each medication cycle was to begin on the same day of the week. Administration of the IMP was to be continued in this manner for nine medication cycles in total.

If any bleeding or spotting occurred, the intake of the IMP was to be continued. In case of heavy bleeding, the subject was asked to consult the investigator for diagnostic clarification. The administration of hormonal preparations to treat bleeding was not allowed during the course of the trial as this might have influenced the results.

It was anticipated that contraceptive protection might be reduced if more than 36 hours had elapsed between two tablet intakes.

- If the subject was less than 12 hours late in taking any tablet, the missed tablet was to be taken as soon as it was remembered and the next tablet was to be taken at the usual time.
- If the subject was more than 12 hours late, she had to take the tablets as soon as she remembered and then take the next ones on time, even if that meant taking up to four tablets at the same time. If the subject was more than 12 hours late, she should use an additional method of contraception (barrier method) for the next seven days.

If tablets were missed in the first week and intercourse took place in the week before the tablets were missed, the possibility of a pregnancy was to be considered.

In case of severe gastro-intestinal disturbance, absorption may not be complete and additional contraceptive measures were to be taken. If vomiting occurred within 3-4 hours after tablet-taking, absorption might not be complete and an additional method of contraception (barrier method) for the next seven days was to be used. IMP intake, forgotten intake of IMP and concomitant contraceptive devices had to be recorded in the electronic diary on a daily basis. The subjects had a possibility to fill in the e-diary retrospectively only for one missed day.

In addition to the records in the CRF the investigator was to keep a separate record of the subject's number, subject's name, date of dispensing and amount of IMP dispensed to each subject.

9.4.4 Blinding

The test product and the test placebo, as well as the reference product and the reference placebo were identical in size, colour and appearance. The packaging and labelling did not allow for any distinction between the products and their corresponding placebos.

During the trial, the subjects and all personnel involved in the conduct and interpretation of the trial, including the investigators, site personnel, and the sponsor’s staff, were blinded to the medication codes. The randomisation schedule was to be filed securely by the CRO, in a manner such that blinding was properly maintained throughout the trial. Medication codes were not to be available until the completion of the trial and until after final data review (clinical data base lock), except in the case of emergency.

The unblinding was to be done only in the case of emergency when the knowledge of the treatment was needed to treat the subject. Emergency unblinding was to be done via sealed emergency code envelopes. The CRO Medical Monitor was to be contacted, if possible, in advance. The date and reason for breaking the blind were to be documented on the envelope and in the CRF. In case of decoding, the subject was to be withdrawn from the trial.

Subjects with suspected unexpected serious adverse reactions (SUSARs) were to be unblinded for regulatory reporting by the CRO Drug Safety representative. Other study personnel and the investigators were to receive blind information on the SUSAR until the study was unblinded.

9.4.5 Prior and concomitant therapy

Any medication used within one month prior to screening or during the trial, including over-the-counter medications and herbal remedies was to be documented with the start and stop dates and frequency on the appropriate CRF page. Cosmetics and dietary supplements were not to be recorded.

All contraceptives, including emergency contraception, used within six cycles before Visit 1a were to be documented in the CRF. The subjects were to document any concomitant use of contraceptives, including emergency contraception, in the electronic diary (e-diary) from the start of IMP intake to Visit 5.

In this trial the following previous therapies and contraceptive devices were not permitted:

- Injectable hormonal methods of contraception within the last six months before V1a
- Progestin-releasing IUD or contraceptive implant within the last two months before V1a
- Anti-retroviral therapy within the last six months before V1a
- Microsomal enzyme-inducing drugs within the last 28 days before the start of IMP intake

The concomitant use of the following medications and contraceptive devices was not permitted during the course of the study:

- Estrogens
- Progestogens (also for the treatment of spotting or unscheduled bleeding)
- Barrier contraceptive methods (for definition see Section 4.1), except occasional use due to the risk of an infection, or in case of gastro-intestinal disorders, vomiting or missed tablet as described in Section 9.4.3
- Spermicides, excepting occasional use in case of gastro-intestinal disorders, vomiting or missed tablet as described in Section 9.4.3
- Emergency contraception
- IUDs
- Other contraceptive measures
- Activated charcoal taken within three hours before or after intake of IMP

Women receiving long-term treatment (longer than seven days) with one of the below listed drugs were to be excluded from the study and were to be advised on additional contraceptive measures by the investigator.

If short-term treatment (up to seven days) with one of the below-mentioned drugs was unavoidable during the study, the subject had to inform the investigator and other protective measures like condoms or diaphragms had to be used to guarantee reliable contraception (the "temperature method" and the calendar method by Knaus-Ogino were not recommended). The use of supplemental contraceptive measures had to be continued for 28 days after the discontinuation of the concomitant treatment. The chosen protective measures were to be recorded in the subject's electronic diary.

- Microsomal enzyme-inducing drugs
- Anticonvulsants (e.g. phenytoin, carbamazepine, oxcarbazepine, topiramate, felbamate, primidone)
- Barbiturates
- Antibiotics such as rifabutin or rifampicin

- Ritonavir, nelfinavir
- Atorvastatin
- Bosentan
- Griseofulvin
- Phenylbutazon
- St. John's wort (hypericum perforatum)
- Medications that might increase serum potassium (ACE inhibitors, angiotensin – II receptor antagonists, potassium-sparing diuretics, potassium supplementation, heparin, aldosterone antagonists and nonsteroidal antiinflammtatory drugs, NSAIDs). No additional contraceptive measures were necessary in this case.

9.4.6 Treatment compliance

The trial staff was to dispense the appropriate amount of investigational medicinal products for each subject and for each treatment interval. At each visit, subjects had to bring back the trial medication (including empty and partially empty containers) and product accountability was to be performed by the trial staff.

9.5 Efficacy, Safety and Tolerability Variables

9.5.1 Efficacy and safety measurements

9.5.1.1 Efficacy assessment criteria

The efficacy assessments were based on the pregnancy tests that were to be performed throughout the trial. Blood samples for the serum pregnancy test were to be collected at V1a and V5 (or EDV). The urine pregnancy test by dipstick was to be performed at V2, V3, V4 and V6 and in any case during the study when pregnancy was suspected.

At V1b, V2, V3 and V4, the subjects were to be provided with home pregnancy test kits and instructed to perform pregnancy tests at home at the start of each new medication cycle.

The results of all these pregnancy tests were to be documented in the CRF.

9.5.1.1.1 Overall Pearl Index

The overall Pearl Index was defined as follows:

Overall PI = number of pregnancies*1300/number of medication cycles

Overall PI was to include all pregnancies which occurred during the study. Pregnancies following premature termination of IMP were to be excluded from calculations unless intravaginal ultrasound examination and β–HCG test was not performed to determine whether the date of conception was after the premature discontinuation [5].

Medication cycle was defined as 28 days starting with the administration of the first tablet from the blister containing 28 tablets and ending with the last day of intake.

9.5.1.1.2 Pearl Index for method failures

Method failures PI included all pregnancies when IMPs were used correctly. Pregnancies were to be excluded from the calculation if in the medication cycles of conception or in the previous medication cycles depending on the date of conception:

- not all active tablets were taken
- there was a tablet-free interval between the previous medication cycle and the medication cycle of conception
- vomiting or diarrhoea was documented

- administration of prohibited prior or concomitant therapy as detailed in Section 9.4.5 was documented

9.5.1.1.3 Pearl Index after correction for back-up contraception

For the calculation of the PI after correction for back-up contraception all medication cycles in which back-up contraception was used were to be considered as not evaluable and were not to be included in the analysis.

9.5.1.1.4 Life table analysis

In addition to PI analysis, overall and method failure life table analysis was to be performed in order to have the pregnancy rate at each month and cumulative pregnancy rates. For overall life table analysis as well as for overall PI analysis all pregnancies that occurred during the study were to be included and all pregnancies following premature termination of IMP were not to be included in calculations unless an appropriate test was not performed to determine the date of conception [5]. Method failure pregnancy rates similar to method failure PI were to include only those cycles when IMP was used correctly.

9.5.1.2 Safety and tolerability assessment criteria

9.5.1.2.1 Safety assessments

9.5.1.2.2 Adverse events

<u>Definitions</u>

An **adverse event** (AE) is any untoward medical occurrence in a subject or clinical investigation subject administered a pharmaceutical product and which does not necessarily have to have a causal relationship with this treatment. An AE can therefore be any unfavourable and unintended sign (including an abnormal laboratory finding, for example), symptom, or disease temporally associated with the use of a medicinal product, whether or not considered related to the medicinal product.

Laboratory, ECG or vital signs' abnormalities were to be documented as AEs if they were considered clinically significant, required treatment, fulfilled any SAE criterion, or caused the subject to change the trial schedule.

In the case of laboratory/ECG abnormalities that were a sign of a medical condition, the condition was to be reported as an AE and not the sign.

All noxious and unintended responses to a medicinal product related to any dose were to be considered **adverse drug reactions** (ADRs). The definition covers also errors and uses outside of the protocol, including misuse and abuse of the product.

The phrase "responses to a medicinal product" means that a causal relationship between a medicinal product and an adverse event is at least a reasonable possibility, i.e., the relationship cannot be ruled out.

All AEs for which the judgement of relationship to trial medication was "possibly related" or "related" were to be considered ADRs.

A **serious adverse event** (SAE) is any untoward medical occurrence that at any dose:

- results in death,
- is life-threatening,

  NOTE: The term "life threatening" in the definition of "serious" refers to an event in which the subject was at risk of death at the time of the event; it does not refer to an event which hypothetically might have caused death if it were more severe.
- requires in-patient hospitalisation or prolongation of existing hospitalisation*,

- results in persistent or significant disability or incapacity, or
- is a congenital anomaly or birth defect;
- is an important medical event that requires intervention to prevent one of the above.
- NOTE: Medical and scientific judgement was to be exercised in deciding whether expedited reporting was appropriate in other situations, such as important medical events that might not be immediately life threatening or result in death or hospitalisation but might jeopardise the subject or might require intervention to prevent one of the other outcomes listed in the definition above. These should also usually considered serious.

  * "In patient hospitalisation" is defined as 24 hours in hospital or an overnight stay.

An AE involving hospitalisation due to a planned trial visit and for no other reason and without prolongation, an overnight hospitalisation due to transportation, organisation or accommodation problems without medical background or an elective or preplanned hospitalisation for an existing condition that had not worsened was not to be considered as an SAE.

A **suspected, unexpected serious adverse reaction** (SUSAR) was a serious adverse reaction which fulfilled the precondition of not being previously reported with regard to type, severity or frequency of occurrence in the applicable product information, i.e. current Investigator's Brochure [3].

Classification of the severity of AEs

The maximum severity (intensity) of the AE was to be categorised by the investigator as follows:

- **Mild:** A type of adverse event that is usually transient and may require only minimal treatment or therapeutic intervention. The event does not generally interfere with usual activities of daily living.
- **Moderate:** A type of adverse event that is usually alleviated with additional specific therapeutic intervention. The event interferes with usual activities of daily living, causing discomfort but poses no significant or permanent risk of harm to the research participant.
- **Severe:** A type of adverse event that requires intensive therapeutic intervention. The event interrupts usual activities of daily living, or significantly affects clinical status. The event possesses a significant risk of harm to the research participant and hospitalisation may be required.

AE causality assessment

The assessment of the causal relationship of an AE to the investigational medicinal product was to be a clinical decision made by the investigator based on all available information at the time of the completion of the CRF. The following classification was to be used:

- **Not related:** A clinical event with no evidence of any causal relationship.
- **Unlikely related:** A clinical event, including laboratory test abnormality, with a temporal relationship to drug administration which makes a causal relationship improbable, and in which other drugs, chemicals or underlying disease provide plausible explanations.
- **Possibly related:** A clinical event, including laboratory test abnormality, with a reasonable time sequence to administration of the drug, but which could also be

explained by concurrent disease or other drugs or chemicals. Information on drug withdrawal may be lacking or unclear.

- **Related:** A clinical event, including laboratory test abnormality, occurring in a plausible time relationship to drug administration, and which cannot be explained by concurrent disease or other drugs or chemicals. The response to withdrawal of the drug (dechallenge) should be clinically plausible. The event must be definitive pharmacologically or phenomenologically, using a satisfactory rechallenge procedure if necessary.

Rating of frequency of AEs

The frequency of AEs was to be categorised by the investigators as follows:

- **Single:** Single occurrence without interruption lasting up to 24 hours.
- **Intermittent:** Intermittent events were to be recorded as **one** event if the time period between two manifestations was less than 24 hours.
- **Continuous**: Continuous occurrence without interruption lasting longer than 24 hours.

Recording and documentation of AEs by the Investigator

All AEs, including SAEs, occurring within the period of observation for the clinical trial had to be recorded.

The period of observation for the collection of AEs extended from the time when the subject gave informed consent until the date of Visit 6. Any AE that was still ongoing after Visit 6 was to be left as ongoing in the CRF. However the investigator was to continue to follow up the ongoing AEs until these were finally resolved or it was medically justifiable to stop further follow up (e. g. a chronic condition was reached) and record information in the source documents.

If the investigator detected an SAE/AE of special interest in a trial subject after the end of the period of observation, and considered the event to be at least possibly related to prior trial treatment or procedures, he or she had to contact the CRO to determine how the SAE/AE of special interest should be documented and reported.

At each visit, the investigator was to ask the trial subject in a non-leading manner about the state of his/her health in order to elicit information on AEs which might have occurred since the last visit. Any clinically significant observations made during the visit itself also were to be documented as AEs.

The AEs had to be documented as soon and as completely as possible on the “Adverse Events” pages in the CRF. AE recording included duration (date of occurrence and resolution), causality, seriousness, severity, frequency, treatment, action taken and outcome.

A clinically significant worsening of an AE (e.g., relevant change in severity, seriousness) had to result in a new entry. The original entry was to remain unresolved and was to be given an end date reflecting the date of the worsening and a comment had to be entered stating that the AE was continuing with a changed severity/seriousness (e.g., “continues as event name with onset date and new severity/seriousness”). The onset date of the new entry was also the date of worsening. The onset date of an SAE was the time as of which the event fulfiled a criterion for seriousness.

Adverse events which occurred during the trial were to be treated by established standards of care to protect the life and health of the subject. If such treatment constituted a deviation from

the protocol, the subject was to be withdrawn from the trial and the reason had to be documented in the CRF.

Reporting of SAEs/AEs of special interest detected after final visit

If the investigator detected an SAE/AE of special interest of the trial subject after the end of observation period and considered the event to be at least possibly related to trial treatment or procedures, he/she had to contact the CRO to determine how to document and report the event.

Follow-up of subjects with AEs

All AEs, irrespective of severity and whether serious or not, were to be monitored by the investigator through the entire trial until completion (V6, Follow-up visit) or discontinuation (EDV). Any AE that was still ongoing after V6 was to be documented as “ongoing” in the CRF. However, the investigator was to continue to follow up ongoing AEs until these were finally resolved or it was medically justifiable to stop further follow up (e.g. a chronic condition was reached) and record information in the source documents.

Reporting of SAEs and AEs of Special Interest

Any SAE was to be reported after first knowledge of the event, within 24 hours, to the CRO Drug Safety contact:

Ruta Kvederiene
SCOPE International
Kalvariju 300
LT-08318 Vilnius, Lithuania
E-mail: rkvederiene@scope-international.com
Phone: +370 52360349
Fax: +370 52327903

The CRO Drug Safety representative was to report the respective (S)AEs to León Farma within one working day, to:

Nieves Fernandez
Quintanapalla 2
28050 Madrid, Spain
Phone: +34 619 275 590
Fax: +34 91 766 89 63

Expedited and periodic reporting required by the above mentioned functionaries and institutions were to be fulfilled according to current international regulations, local laws and guidances. The detailed reporting duties and division of responsibilities between the sponsor and the CRO were detailed in a Safety Plan.

Pregnancy reporting and follow-up

The subjects were to be instructed to contact the investigator or trial staff immediately if pregnancy was suspected. This applied also to pregnancies within three months following regular or premature termination of a subject. Pregnancy discovered during the clinical trial had to lead immediately to exclusion (if at screening) or withdrawal of the subject.

Pregnancy had to be documented by completing the Pregnancy Report Form and had to be reported immediately within 24 hours of having gained knowledge of the pregnancy by facsimile or e-mail to the Drug Safety Manager (see contact details for SAE reporting above).

The Drug Safety Manager had to take care of informing the sponsor, the responsible Clinical Research Associate (CRA) and other relevant administrative sites.

Any pregnant subject discovered after IMP administration had to be followed up until completion of pregnancy. Pregnancy outcome had to be documented and reported by completing and faxing the Pregnancy Outcome Form. The follow-up report was to be sent three months after parturition. Individual cases with abnormal outcome (congenital anomalies in the foetus/child, reports of induced, elective abortions, foetus death and spontaneous abortion, and adverse reactions in the neonate that were classified as serious) were to be reported as SAEs.

9.5.1.2.3 Adverse events of special interest: venous thromboembolism and hyperkalaemia

In this trial deep vein thrombosis or pulmonary embolism and hyperkalaemia were considered as AEs of special interest. In case of suspected deep vein thrombosis, pulmonary embolism or hyperkalaemia IMP was to be discontinued, hospitalisation was to be considered and clinically significant cases were to be reported as SAEs (seriousness criterion: medically important condition), including follow-ups.

The clinical significance of the elevated blood potassium levels had to be assessed both by the investigator and sponsor's medical monitor. Potassium level, time and the level of potassium increase from the previous known value, any clinical signs or symptoms that could be associated with elevated potassium and any additional risks for the individual subject were to be considered. Possible reasons for pseudohyperkalaemia were to be checked (haemolysis – blood sampling was to be repeated, thrombocytosis: platelets > 500 x 10^9/L), the subject was to be interviewed about intake of concomitant medication that might have caused hyperkalaemia (such as ACE inhibitors, potassium-sparing diuretics etc.). In case of elevated serum potassium level, the following investigations were to be performed or collected: blood test for electrolytes, renal function, creatine kinase and blood gas analysis (including pH); urine test for potassium, calculation of trans-tubular potassium gradient, ECG and vital signs.

In case of VTE the subject was to be interviewed about immobilisation, complete blood count and coagulation parameters were to be performed, duplex ultrasonography (or other imaging tests) and ECG were to be performed. If necessary, all cases of VTE were to be reviewed by a VTE Committee.

9.5.1.2.4 Clinical laboratory evaluations

Urine dipstick tests, including pregnancy tests, were to be performed by the investigator. Additionally, the subjects were instructed to perform urine pregnancy tests at home at the beginning of each medication cycle. All other laboratory analyses were performed at a central laboratory, LKF - Laboratorium für Klinische Forschung GmbH (see Section 6 for address). Details regarding the collection, shipment of samples, reporting of results, and alerting of abnormal values were to be outlined in a laboratory manual, which was to be supplied to all centres.

Blood samples for haematology, biochemistry, thyroid function assessments were to be collected at V1a, V3, V4 (electrolytes only) and V5 (or EDV). The condition at time point of blood sampling (fasting or non-fasting) was to be documented in the CRF.

Blood samples for the special laboratory parameters, haemostatic variables, carbohydrate metabolism and bone metabolism, for a subset of subjects were to be collected at V1b and V5 (or EDV) under fasting condition. The bone marker levels follow a circadian rhythm, therefore all blood samples for the special laboratory parameters had to be drawn between 8 and 9 a.m. to ensure the comparability of the results.

Serum pregnancy tests were to be performed at V1a and V5 (or EDV).

Samples for urinalysis were to be collected at V1a, V3 and V5 (or EDV). Urine dipstick tests were to be performed by the investigator. If any of the measured parameters of urine analysis was out of range/pathologic, the sample was to be shipped to the central laboratory for microscopic examination.

Urine pregnancy tests by dipstick were to be performed at V2, V3, V4 and V5 and in any case during the trial when pregnancy was suspected.

The laboratory parameters assessed during the trial are listed in Table 6 below.

Table 6: Safety laboratory parameters

Thyroid function	Thyroid-stimulating hormone (TSH)
Haematology	Red blood cell count, haemoglobin, mean corpuscular volume (MCV) and associated parameters, mean corpuscular haemoglobin (MCH), haematocrit, white blood cell count, differential white blood cell count including neutrophils, lymphocytes, eosinophils, basophils and monocytes, and platelet count.
Biochemistry	Sodium, potassium, chloride, creatinine, blood urea nitrogen (BUN), calcium, glucose, total proteins, albumin, total cholesterol (high- and low-density lipoproteins HDL, LDL cholesterol), triglycerides, alanine aminotransferase (ALAT), aspartate aminotransferase (ASAT), gamma-glutamyl transferase (gamma-GT), alkaline phosphatase (ALP), total and direct bilirubin, creatine phosphokinase (CPK) and lactate dehydrogenase (LDH).
Urinalysis	Protein, glucose, ketones, blood, nitrite, urobilinogen, bilirubin, haemoglobin, leucocytes and pH.
Pregnancy tests	Serum and urine β-human chorionic gonadotropin (β-hCG) tests.
**Special tests (planned for a subset of at least 40 subjects per treatment group):**	
Haemostatic parameters	Factor VIIc, factor VIIIc, protein C activity, antithrombin III activity, D-dimer. Activated protein C (APC) resistance was added per Protocol Amendment No. 1 Austria and Protocol Amendment No. 2 Germany.
Carbohydrate metabolism	Plasma fasting glucose, serum insulin, C-peptide.
Bone metabolism	Bone alkaline phosphatase, cross-linked c-terminal telopeptides (CTX)

Deviations from the reference ranges of the laboratory parameters had to be evaluated with regard to clinical significance by the investigator.

All new clinically significant laboratory findings or worsening of clinically relevant conditions had to be reported as AEs and had to be followed with appropriate medical care, even after termination of the study, until normal or baseline values were reached and the condition had stabilised or a non-IMP cause had been identified.

Haemolysed blood samples had not to be analysed for electrolytes. The laboratory had to inform the site about the haemolysis of the sample and another blood sample was to be taken and sent to the laboratory for analysis.

9.5.1.2.5 Vital signs

Vital signs parameters comprised systolic blood pressure (SBP), diastolic blood pressure (DBP), heart rate, body height, weight and body mass index (BMI).

Blood pressure and heart rate were to be measured at screening, V2, V3, V4 and V5 (or EDV).

In order to obtain accurate readings, the following points were to be observed:

- Painful procedures, like drawing blood, should always be performed after vital signs measurements (not before).
- A calibrated sphygmomanometer was to be used for all measurements in this trial.
- A proper cuff size was to be used (the ideal ratio between cuff width and arm circumference is 0.4)
- The right arm was always to be used. (If this was not possible, the left arm was always to be used.)
- The subject had to rest in a chair with a back on it for at least 5 minutes, and should not talk.
- The subject had to keep her arm (the elbow pit) at heart level. She was allowed to rest her arm on a table in a comfortable place, provided the elbow pit was at the level of the heart.
- Three measurements with 1 minute pause between them were to be performed.

Height was to be measured at screening only. Body weight was to be measured at screening, V2, V3, V4 and V5 (or EDV), in underwear and without shoes. The BMI as a measure for the physical constitution of a subject was to be calculated by weight/(height)^2.

9.5.1.2.6 12-Lead electrocardiogram

In a subset of 200 subjects a 12-lead ECG (supine) was to be performed at V1b and V5 (or EDV). ECG recording was to be performed after the subject has been resting for 5 min. In order to ensure data comparability, all participating sites were to be provided with the same ECG device model. All ECG data were to be transferred electronically to the central ECG assessment centre (for the address see Section 6).

9.5.1.2.7 Physical examination

Physical examination was to be performed at screening and V5 (or EDV) for all countries, except Germany. For German subjects physical examination with particular attention to the lower extremities was to be performed at all study visits (Protocol Amendment No.1 Germany). The parameters to be assessed were general appearance, ears, eyes, nose, throat, lungs/chest, heart, abdomen, back, thyroid, lymph nodes, skin and extremities. The results were to be documented in the CRF.

9.5.1.2.8 Gynaecological examination

At screening and V5 (or EDV) all subjects were to undergo a gynaecological examination including inspection of the external genital organs, speculum examination, palpation of the internal genital organs and examination of the breasts. If at screening results from a gynaecological examination within two weeks before were available, covering the details as specified below, the examination had not to be repeated. The palpation was to be performed always after generation of the cytological smear since the palpation might cause minor internal damage, sometimes associated with minor bleedings. Also vaginal interventions or application of medication were to be avoided within at least 24 hours prior to the cytological smear test.

9.5.1.2.9 Cervical smear according to Papanicolaou

Cervical cytology was to be performed at screening and V5 (or EDV). If at V1a the cytological report from a cervical cytology examination within three months before was available covering the details as specified below, examination was not to be repeated, but could be performed optionally. For the cytological smear according to Papanicolaou (PAP smear) three swabs were to be taken: the first from the portio vaginalis by wiping over the complete surface of the portio vaginalis, the second and the third from the cervical channel.

The PAP smear assessments were performed centrally by an external pathologist (for contact details see Section 6). The cytological smear was to be assessed according to the 2001 Bethesda System. Subjects with positive test results (ASC-US or worse) at screening were not to be included in the trial.

9.5.1.2.10 Intravaginal ultrasound examination

Intravaginal ultrasound examination was to be performed twice: at screening and V5 (or EDV) to detect any pathological findings of the uterus, endometrium and ovaries. Ovarian cysts, i.e. any fluid-filled structures larger than 30 mm in diameter that persisted for more than two cycles, were to be documented as pathological findings. Worsening of an existing ovarian cyst at V1a or new occurrence of an ovarian cyst during the study was to be documented as adverse events. Any structure similar to an ovarian cyst that did not persist was to be defined as an enlarged follicle and not to be considered pathological.

All gynaecological, intravaginal ultrasound examination and cervical cytology results were to be documented in the CRF.

9.5.1.2.11 Tolerability assessments

The tolerability assessments were based on the vaginal bleeding pattern. From Day 1 of Medication Cycle 1 (i.e. start of IMP intake) to V5/EDV, subjects had to record daily any vaginal bleeding or spotting in their electronic diary, which comprised the following details:

- Presence of any vaginal bleeding or spotting (Yes, No)
- Bleeding intensity (slight, moderate, heavy)

9.5.1.2.12 Demographic and social data, medical history and other baseline assessments

At V1a subject's year of birth, ethnic group, highest level of education completed, sexual activity, smoking history, alcohol consumption, medical and surgical history for former six months or longer, if relevant, including VTE risk; gynaecological and obstetrical history of the subject were retrieved by a physician. The questioning included details on bleeding, the occurrence of dysmenorrhoea and mastodynia/mastalgia. Prior medications used within one month before V1a and all contraceptives used within six cycles before screening were to be recorded.

9.5.2 Appropriateness of measurements

All efficacy, safety and tolerability measurements described in Section 9.5 are recognised standard methods. Therefore, no further details concerning reliability or relevance will be discussed here.

9.5.3 Primary efficacy variable

The primary efficacy variable was the overall Pearl Index.

9.5.4 Drug concentration measurements

No drug concentration measurements were performed in this trial.

9.6 Data Quality Assurance

All aspects of the protocol were to be complied with during the trial. Should amendments become necessary, these were to be discussed immediately and in detail between the clinical investigator and the sponsor. The agreement reached had to be presented in writing in the form of a protocol amendment, which gave details of the modification and the reasons for the change. This was to be submitted to the ethics committee.

All other persons involved in the trial were to be informed of their responsibilities by the investigator and the tasks for all investigators' staff members were defined in the delegation sheet. The correct execution of all tasks related to the trial was to be supervised by the investigator during the course of the trial.

An Investigators' Meeting was not performed. In order to guarantee that all investigators participating in the study had the same understanding of the study objectives and relevant procedures, the participating investigators were familiarised with the study protocol, CRF and all scheduled assessments during the site initiation visit.

Monitoring

At regular intervals throughout the trial, the trial sites were monitored by authorised CRO monitors who were specially trained for this clinical trial. A CRA meeting took place in Deidesheim, Germany on 6-7 February 2012. Monitoring visits before, during and at the end of the trial were carried out according to ICH GCP guidelines. These were supplemented by telephone and written contacts.

The monitors were responsible for verifying the following:

- Compliance with the protocol
- Subject enrolment and consent procedure
- Completeness, exactness and plausibility of data entered in the CRF by verifying them against the source documents
- Occurrence of AEs and AE procedures
- Storage and accountability of the IMP
- Organisation of the investigator's site file
- Adherence to local regulations on the conduct of clinical trials

Clinical database

The clinical database was set up and maintained by the CRO. All data management procedures were described in a separate Data Management Plan. Data management was performed using Clintrial version 4.5 (Phase Forward Inc., Waltham, USA). Data were entered into the database system using double data entry techniques by two independent data entry typists. A comparison of these entries was performed electronically and emerging discrepancies were checked and corrected. After entry into the database all data were checked and clarified according to a pre-specified data validation plan. Edit and consistency checks were also performed as outlined in the Data Management Plan.

AEs, concomitant diseases and medical history entries were coded according to Medical Dictionary for Regulatory Activities (MedDRA) version 15.1. The World Health Organization Drug Dictionary Enhanced (WHO DDE), March 2013 was used for coding of concomitant medications.

After resolution of all queries, the database was locked and the data were exported to SAS® version 9.3 for Windows (SAS Institute Inc., Cary, NC, USA).

Laboratory

Blood samples were sent to the central laboratory (LKF - Laboratorium für Klinische Forschung GmbH). The analytical work was conducted according to validated methods/Good Laboratory Practice standards. Details regarding the collection, shipment of samples, reporting of results, and alerting of abnormal values were outlined in the laboratory manual,

which was sent to all centres. Central laboratory data were electronically transferred into the clinical database.

The laboratory certificates are provided in Appendix 16.1.10.

Auditing

The following external on-site audits were performed:

- Trial centre 453 in Hungary on 13-FEB-2014
- Trial centre 553 in Poland on 20 and 21-FEB-2014.

An inspection by Austrian Competent Authority was performed at trial centre 151 in Austria on 16 and 17-APR-2013.

The audit certificates are provided in Appendix 16.1.8.

9.7 Statistical Methods Planned in the Protocol and Determination of Sample Size

9.7.1 Statistical and analytical plan

The statistical analyses performed in this trial were initially specified in the protocol (Appendix 16.1.1). Full details of the statistical analyses, data conventions for analysis and presentation of data are provided in the Statistical Analysis Plan (SAP) (Final Version dated 28-APR-2014), which is located in Appendix 16.1.9.

Efficacy target parameters

Primary: Overall Pearl Index, including subgroup of subjects ≤35 years.

Secondary: method failure PI, PI after correction for additional contraception and sexual active cycles, overall and method failure pregnancy ratios, including subgroup of subjects ≤35 years.

Safety target parameters

AEs, clinical laboratory parameters, ECG, vital signs, gynaecological examination assessments, cervical smear, intravaginal ultrasonography results and physical examination findings.

Tolerability parameters

Vaginal bleeding pattern.

Other parameters

Demography, substance usage (smoking, alcohol consumption), scheduled/menstrual bleeding pattern within six cycles before screening, dysmenorrhoea and mastodynia/mastalgia characteristics within three cycles prior to screening, medical and gynaecological history, VTE risk factors (family history of thromboembolic illness, evidence of predisposing conditions for a vascular or metabolic disease, current smokers older than 35 years or non-smokers over 40 years old and subjects with BMI > 30 kg/m^2) at screening, prior and concomitant medication and therapies and IMP compliance.

9.7.1.1 Analysis sets

Analysis sets

According to the protocol, the following analysis sets were defined:

- The **Safety Set (SS)** consisted of all subjects who were randomised, had received at least one dose of IMP and had at least one post-baseline assessment of any safety/tolerability measurement.

- The **Full Analysis Set (FAS)** consisted of all subjects who were included in the SS and had at least one post-baseline assessment of any efficacy measurement.
- The **Per Protocol Set (PPS)** consisted of all subjects who were included in the FAS and did not present any major protocol violation.

During the Blind Data Review Meeting it was decided to expand the definition of the FAS and to include explanation of "at least one post-baseline assessment of any efficacy measurement", by adding "i.e. had at least one exposure cycle or had at least one post- baseline pregnancy test performed". Also a decision was made not to use the PPS for analyses since the Pearl Index was to be calculated using method failure and therefore protocol deviations were to be taken into account. Therefore protocol deviations were not categorised into major and minor rather they were categorised according to their impact on the perfect medication cycles.

Additionally in the SAP the following analysis sets were defined:

- **Enrolled Set** consisted of all subjects who signed informed consent.
- **Randomised Set** consisted of all subjects who were enrolled into the trial and were randomised.

For changes in the conduct of the planned analyses, see Section 9.8.

9.7.1.2 Statistical methods

9.7.1.2.1 Efficacy analysis

All efficacy variables were to be calculated separately for this trial and by pooling the data of the current trial and CF111/301 trial. All efficacy variables were analysed for the FAS.

Analysis of the primary efficacy variable:

Primary efficacy variable was the overall Pearl Index. Overall PI was also to be calculated for the subgroup of subjects ≤ 35 years.

Pregnancies were to be classified as M for method failure, U for user failure, A for post-therapy:

- M (method failure) - pregnancy where the subject was compliant with IMP dosing near the time of conception and estimated date of conception was during treatment period (extended with a maximum of 2 days)
- U (user failure) = pregnancy where the subject failed to comply with IMP dosing near the time of conception and estimated date of conception was during treatment period (extended with a maximum of 2 days)
- A (post-therapy) - pregnancy where estimated date of conception was after the stop date of IMP intake.

All cycles were to be categorised as follows:

- Exposure cycles: 28 days cycles, where at least one e-diary entry of pill intake was available
- Sexual activity cycles: Cycles, where sexual activity was documented in the CRF, in case such entry was not available, e-diary entries were to be taken into account
- Cycles without additional contraception: Cycles where no additional contraception was documented, neither in the CRF nor in e-diary

- Sexual activity cycles without additional contraception: Cycles, where sexual activity was documented in the CRF (in case such entry was not available, e-diary entries were to be taken into account), and no additional contraception was documented neither in the CRF nor in e-diary)
- Perfect medication cycles (sexual activity cycles without additional contraception where the e-diary documented regular pill intake during the cycle, excluding the cycles with four or more days with forgotten tablets, or two or more consecutive days with forgotten tablets during the cycle and no protocol deviations having effect on this cycle.)

Overall Pearl Index was to be calculated as: number of pregnancies (M, U) * 1300/number of exposure cycles.

The two-sided 95% confidence interval (CI) for the overall PI was to be calculated assuming that events of pregnancy have a Poisson distribution. The CI was to be calculated using the following equations from Gerlinger et al. [8]:

$$CI_{lower}(PI) = CI_{lower}(\text{number of pregnancies (M,U)}) * 1300/\text{number of exposure cycles}$$
$$CI_{upper}(PI) = CI_{upper}(\text{number of pregnancies (M,U)}) * 1300/\text{number of exposure cycles}$$

where

$$CI_{lower}(\text{number of pregnancies}) = 0.5\ \chi^2(0.025; 2*\text{number of pregnancies (M,U)})$$
$$CI_{upper}(\text{number of pregnancies}) = 0.5\ \chi^2(0.025; 2*(\text{number of pregnancies (M,U)}+1))$$

with $\chi^2(\alpha; n)$ = α-Quantile of χ^2-distribution with parameter n.

Analysis of the secondary efficacy variables:

Secondary efficacy variables were:

- Method failure PI
- PI after correction for additional contraception and sexual intercourse status
- Overall pregnancy ratio
- Method failure pregnancy ratio

Method failure PI was to be calculated as: Number of pregnancies (M) * 1300/Number of perfect medication cycles.

Method failure was to include all pregnancies categorised as M.

PI after correction for additional contraception and for sexual activity was to be calculated as: Number of pregnancies (M,U) * 1300/Number of medication cycles (excluding those with back-up contraception and without sexual activity).

The two-sided 95% CI was calculated for the method failure PI and for the PI after correction for back-up contraception, as described above for the overall PI.

Overall pregnancy ratio was to be calculated as: Total number of pregnancies (M,U)/Total number of FAS subjects.

Method failure pregnancy ratio was to be calculated as: Total number of pregnancies (M)/Total number of FAS subjects.

Life table analysis was to be performed in order to have the pregnancy rate at each month and cumulative pregnancy rates. For the overall life table analysis all pregnancies categorised as M or U were to be included and all pregnancies following premature termination of IMP

were not to be included in calculations unless an appropriate test was not performed to determine the date of conception.

The cumulative pregnancy rate was to be calculated using the *Proc lifetest* procedure. The Kaplan Meier method was to be used to estimate the cumulative pregnancy probability. The period from the start of treatment until the pregnancy was to be the time variable in this analysis. Subjects who did not become pregnant were to be censored at their time of last administration of IMP. Subjects who became pregnant were to be censored at the estimated date of conception.

The Clopper-Pearson 95% CI was to be calculated for the pregnancy ratio.

9.7.1.2.2 Safety analysis

The safety variables were to be analysed for the SS. Safety assessments were to be summarised by treatment groups by means of the default summary statistics. For continuous laboratory parameters the quartiles (Q1 and Q3) were to be presented.

Adverse events

For the purpose of analyses, all AEs were coded according to Medical Dictionary for Regulatory Activities (MedDRA) version 15.1. The incidence of AEs was to be compared between the treatment groups using Fisher's EXACT test.

All AEs were to be summarised by number and proportion of subjects and number of AEs. Similar summaries were to be provided for each of the subgroup. All TEAEs were to be summarised by calculating the number and percent of subjects with AEs by primary MedDRA system organ class (SOC) and preferred term (PT). Similar summaries were also be provided for TEAEs by severity and relationship to IMP, for serious AEs and TEAEs as well as for TEAEs that led to study discontinuation and TEAEs of special interest. In addition TEAE summaries for defined subgroups were to be provided. All AE data were to be listed, including separate listings for SAEs, TEAEs leading to premature discontinuation and TEAEs of special interest.

Laboratory data

Haematology, biochemistry and urinalysis variables were to be summarised for each treatment group at each visit. Laboratory values were to be compared using 2-sample t test. Blood glucose, total proteins, total cholesterol, HDL and LDL cholesterol and triglycerides were to be summarised additionally for fasting subjects only. Metabolism parameters were to be splitted by fasting status, only blood samples taken before 11:00 a.m. were to be analysed.

Summaries of quantitative laboratory test results by treatment group and for each defined subgroups were to be presented. Absolute changes from baseline at each scheduled visit were to be presented. For all quantitative variables summaries based on their classification according to the reference range (low, normal or high) and clinical significance as per investigator's assessment, were to be provided for each scheduled visit. Shift tables showing changes in number and percent of subjects with low, normal or high laboratory values from baseline to endpoint, based on reference ranges were to be presented.

Vital signs

Vital signs were to be presented by visit for absolute values as well as for absolute and relative changes from baseline at each scheduled visit and at endpoint. In addition, summary of vital signs was to be provided for defined subgroups.

Absolute change in body weight from baseline was to be compared between the treatment groups using ANCOVA with age and body weight at baseline as covariates and treatment group as fixed factor.

Systolic and diastolic blood pressure absolute and relative changes from baseline to endpoint were to be tested using ANCOVA with age and respective blood pressure at baseline as covariates and treatment as fixed factor.

ANCOVA results were not to be interpreted in a formal confirmatory sense.

Summaries of quantitative vital signs parameters' results by treatment group and for each defined subgroup were to be presented as well.

Cervical smear results

Frequency tabulations for cervical smear examination were to be provided.

Gynaecological and intravaginal ultrasound examination findings

Summary of subjects with normal and abnormal (clinically significant or not) gynaecological and intravaginal ultrasound examination findings were to be summarised by visit, shift tables were to be prepared showing changes from screening to V5 (EDV).

Physical examination findings

Number and frequency of subjects with normal and abnormal (clinically significant or not) physical examination findings was to be provided by scheduled visit. Shift tables were to be prepared showing changes from screening to V5 (EDV).

ECG

Number and proportion of subjects with clinically significant abnormalities (ECG interpretation) by treatment group and scheduled visit as well as summary of ECG variables (heart rate; RR, PR, QRS and QT duration; QTcB and QTcF) as well as interpretation (normal, abnormal) for a subset of subjects were to be provided. The ECG parameters were to be compared between the treatment groups using 2-sample t test, whereas for the comparison of ECG interpretation results Fisher's exact test was to be used.

9.7.1.2.3 Tolerability analysis

Analysis of the tolerability endpoints was to be conducted using the FAS. The tolerability data were to be summarised by treatment groups by means of the default summary statistics.

The following tolerability endpoints were defined:

- Proportion of subjects with unscheduled bleeding/spotting in Cycles 2 to 6 (confirmatory)

The hypothesis that the Test is inferior to the Reference with regard to the proportion of subjects with unscheduled bleeding/spotting during Cycles 2 to 6 was to be tested confirmatorily using χ^2 test.

- Proportion of subjects with unscheduled bleeding/spotting in each cycle from Cycle 2 to 9 and cumulative in Cycles 2 to 4, Cycles 5 to 7 and Cycles 7 to 9

- Proportion of subjects with no bleeding/spotting
- Number of bleeding/spotting days during Cycles 2 to 4, 7 to 9 and 2 to 9.
- Number of bleeding/spotting episodes during Cycles 2 to 4, 7 to 9 and 2 to 9

Number and rate of subjects with different bleeding patterns were to be presented for each cycle and cumulative in Cycles 2 to 4 and Cycles 7 to 9. χ^2 test was to be applied to compare rates in both treatment groups.
Number of days of bleeding/spotting and number of bleeding/spotting episodes were to be analysed and reported by each cycle and defined time periods (cycles 2 to 4, cycles 7 to 9, cycles 2 to 9). The treatment groups were to be compared using a Wilcoxon-rank-sum-test.

For subjects who experienced unscheduled bleeding/spotting , numbers of missed pills or e-diary entries were to be presented. The treatment groups were to be compared using a Wilcoxon-rank-sum-test.

Interim analyses

It was not planned to perform an interim analysis.

Blind data review

A Blind Data Review Meeting was to be held after data entry, prior to the locking of the database.

The following aims were defined for this meeting:

- to assign subjects to each of the analysis sets
- to identify protocol deviations which might have an impact on cycles
- to assign pregnancies to their category
- to define TEAEs of special interest, if necessary
- to check that there were no data issues that are outstanding or need resolution;
- to solve any outstanding issues in the SAP.

All decisions made during the data review meeting were documented in the Blind Data Review Report (see Appendix 16.1.9).

9.7.2 Determination of sample size

Calculation of the number of subjects taking LF111 based on primary efficacy endpoint:

According to the CHMP Guideline on Clinical Investigation of Steroid Contraceptives in Women [5], the number of cycles collected should be at least large enough to give the overall Pearl Index (PI) with a 95% confidence interval (CI) such that the difference between the upper limit of the CI and the point estimate does not exceed 1.

According to Gerlinger C et al. (2003) [8] the number of cycles needed to fulfil the EMA precision requirement for the Pearl Index with a 90% power is as follows:

Assumed true Pearl Index	Number of cycles
0.1	5954
0.2	7501
0.3	8125
0.4	8671
0.5	9178
0.6	10101
0.7	10517
0.8	10920
0.9	11661
1.0	12337
1.2	13286
1.4	14443
1.6	15483
1.8	16443
2.0	17576
2.5	20163
3.0	22737

Two studies (CF111/301 and the current study) were planned for the calculation of the overall PI, and this study was to be performed to have at least half of evaluable cycles needed.

For an assumed PI<1.0 the number of cycles needed to fulfil this precision requirement with a 90% power is 12337 (see the table above). Thus 6169 cycles were to be collected in each of the two studies. This would require 685 evaluable subjects with a treatment duration of nine cycles in this study.

Taking into consideration a possible withdrawal rate of an estimated 20%, 857 subjects taking LF111 were to be enrolled into this study.

<u>Calculation of the number of subjects taking desogestrel 0.075 mg based on secondary tolerability endpoint:</u>

In order to test non-inferiority of the bleeding pattern between the two treatment groups, assuming a 24% proportion of the control group [9], 9% non-inferiority margin, one sided type I error 2.5, 80% power and 2:1 treatment allocation rate, a sample size of 531 in the Test group and of 266 in the Reference group was required. To prove superiority under the same assumptions a sample size of 443 in the Test group and of 222 in the Reference group was required.

Taking into consideration a possible drop-out rate of an estimated 20%, 333 subjects taking desogestrel 0.075 mg were to be enrolled into this study.

In order to attain a 5:2 ratio, 857 LF111 subjects and 343 desogestrel subjects were to be enrolled.

9.8 Changes in the Conduct of the Trial or Planned Analyses

9.8.1 Protocol amendments

All protocol amendments came into effect before the first subject entered the trial.

Protocol Amendment No.1 Germany

This protocol amendment (dated 23-FEB-2012) was prepared to comply with requirements of the EC of the State of Berlin (at the Office for Health and Social Affairs, LAGeSo, Berlin). The following changes were made:

- Diabetes mellitus (in the original protocol: diabetes) and a history or presence of arterial thrombosis or embolism were added to exclusion criterion no. 8.
- Subjects committed to an institution by virtue of an order issued either by the judicial or the administrative authorities were added as a new exclusion criterion no. 22.
- Contraindications from the LF111 Investigator's Brochure: adrenal insufficiency, history or presence of cerebral-vascular disease, headaches with focal neurological symptoms, major surgery with prolonged immobilisation, cholestatic jaundice of pregnancy or jaundice with prior pill use were added as a new exclusion criterion no. 23.
- Safety reasons were explicitly added as criteria for the premature termination of the trial.
- Physical examinations with particular attention to the lower extremities were added at each study visit.
- The follow-up visit (Visit 6) was rescheduled to 10-28 day after the last IMP intake (in the original protocol 7 to 10 days).
- The section on informed consent was corrected, and the relatives of the trial participants' were deleted from the list of persons who must have an opportunity to inquire about the details of the trial.

Protocol Amendment No.2 Germany and Protocol Amendment No.1 Austria

These protocol amendments (both dated 25-JJUN-2012) were prepared to comply with the requirement of the BfArM (Federal Institute for Drugs and Medical Devices), Germany. APC resistance was added to the haemostatic laboratory parameters to be performed in a subset of subjects.

Protocol Amendment No.1 Czech Republic

This protocol amendment (dated 22-MAR-2012) was prepared to comply with requirements issued by the State Institute for Drug Control (SÚKL):

- For women with BMI $\geq$ 30 kg/m^2 three additional exclusion criteria no. 22-24 (family history of VTE in siblings or parents at a relatively young age; history of venous or arterial thrombosis [deep venous thrombosis, pulmonary embolism] and smoking) were added.
- History of migraine with focal neurological symptoms, which is contraindicated for desogestrel, was added as an exclusion criterion no. 25.

9.8.2 Changes to analyses described in the protocol

During the preparation of the SAP, the following changes and corrections were made to the statistical analyses originally described in the trial protocol:

- The **Per Protocol Set** was not defined since the PI was calculated using method failure and protocol deviations were to be taken into account. Protocol deviations were categorised according to their impact on the perfect medication cycles, but not into major and minor.
- **Enrolled Set** consisted of all subjects who signed informed consent.
- **Randomised Set** consisted of all subjects who were enrolled into the trial and were randomised.

- According to the protocol, the Full Analysis Set consisted of all subjects who had received at least one dose of the IMP and who had at least one study observation. The definition of FAS was corrected in the SAP. According to the SAP, the FAS consisted of all subjects who were included in the Safety Set and who had at least one post-baseline assessment of any efficacy measurement, i.e. had at least on exposure cycle or had at least one post-baseline pregnancy test performed.
- Four subgroups, based on subjects' age, BMI and blood pressure were defined (for details, see Section 11.8.2.8).
- The following additional or corrected definitions related to bleeding pattern were given:
  - **Scheduled bleeding day** was defined as any bleeding or spotting that occurs during hormone-free intervals (defined as Days 25 – 28 +/-1). Up to 8 consecutive bleeding/spotting days are considered as scheduled bleeding days. This definition is applicable only to subjects who received DRSP tablets during the trial.
  - **Unscheduled bleeding/spotting day** was defined as any bleeding or spotting that occurs while taking active hormones (Days 2 – 23), except days which are classified as scheduled bleeding days. This definition is applicable only to subjects who received DRSP tablets. In the desogestrel treatment group all bleeding days are classified as unscheduled.
  - The following 3-cycle reference periods were defined for bleeding pattern analysis:
    - Cycles 2 – 4
    - Cycles 5 – 7
    - Cycles 7 - 9
  - The term **amenorrhoea** was not used, "no bleeding/spotting" was used instead.
  - **Episode of bleeding/spotting** was defined as bleeding/spotting days bounded on either end by two days of no bleeding or spotting. (In the protocol this had been defined as bleeding/spotting days bounded on either end by **one** day of no bleeding or spotting.)
  - **Infrequent bleeding** was defined as 1 – 2 bleeding/spotting episodes during the reference period.
  - **Frequent bleeding** was defined as 6 or more bleeding/spotting episodes during the reference period.
  - **Prolonged bleeding** was defined as bleeding/spotting episode with a duration of more than 14 days.

10. TRIAL SUBJECTS

10.1 Disposition of Subjects

Figure 1: Flow Chart of Subjects Disposition

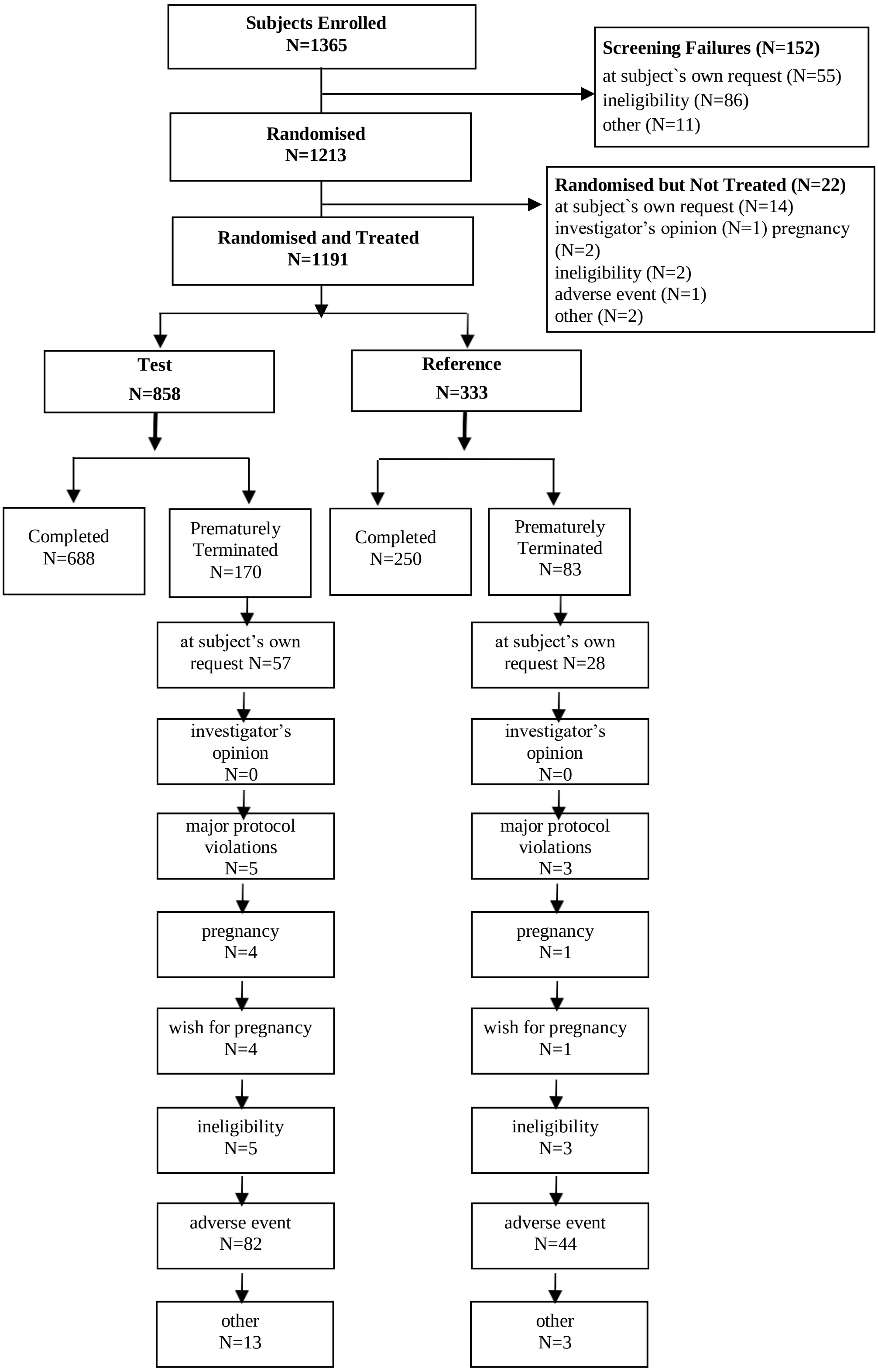

Source: Section 15.1, Figure 15.1.1

The number of subjects and subjects' disposition are presented in Figure 1 and Table 7. A listing of all subjects who prematurely discontinued their participation in the trial prior to randomisation (screening failures) is provided in Appendix 16.2, Listing 16.2.1.1.

Table 7: Subject Disposition (Randomised Set)

	Test		Reference		Total	
	**Treated (N=858) n (%)**	**Not treated (N=14) n (%)**	**Treated (N=333) n (%)**	**Not treated (N=8) n (%)**	**Treated (N=1191) n (%)**	**Not treated (N=22) n (%)**
Subjects who completed the trial	688 (80.2)	-	250 (75.1)	-	938 (78.8)	-
Subjects who prematurely terminated the trial	170 (19.8)	14 (100.0)	83 (24.9)	8 (100.0)	253 (21.2)	22 (100.0)
Primary reasons for discontinuation:						
Withdrawal of consent	57 (6.6)	8 (57.1)	28 (8.4)	6 (75.0)	85 (7.1)	14 (63.6)
Investigator's opinion	-	1 (7.1)	-	-	-	1 (4.5)
Major protocol violations	5 (0.6)	-	3 (0.9)	-	8 (0.7)	-
Pregnancy	4 (0.5)	2 (14.3)	1 (0.3)	-	5 (0.4)	2 (9.1)
Wish for pregnancy	4 (0.5)	-	1 (0.3)	-	5 (0.4)	-
Ineligibility	5 (0.6)	2 (14.3)	3 (0.9)	-	8 (0.7)	2 (9.1)
Adverse event	82 (9.6)	-	44 (13.2)	1 (12.5)-	126 (10.6)	1 (4.5)-
At the sponsor's request	-	-	-	-	-	-
Other	13 (1.5)	1 (7.1)	3 (0.9)	1 (12.5)	16 (1.3)	2 (9.1)

Source: Section 15.1, Table 15.1.1.2
N: Number of subjects in specified group
n: Number of subjects with data available
%: Percentage based on N

Of the 1365 subjects enrolled, 152 subjects prematurely discontinued from the trial before randomisation (screening failures). The primary reasons for discontinuation before randomisation were ineligibility (86 subjects), withdrawal of consent (55 subjects) and other (11 subjects), see Section 15.1, Table 15.1.1.1.

Overall, 1213 subjects were randomised: 327 (27.0%) in Czech Republic, 275 (22.7%) in Poland, 217 (17.9%) in Romania, 172 (14.2%) in Germany, 86 (7.1%) in Hungary, 75 (6.2%) in Spain, 57 (4.7%) in Slovakia and four (0.3%) in Austria (for subject disposition by country and site, see Section 15.1, Table 15.1.1.3).

Of 1213 subjects randomised, 1191 received IMP. 14 subjects randomised to the Test group and eight subjects randomised to the Reference group prematurely terminated the trial without receiving double-blind treatment (Table 7).

Additionally, 253 subjects who had started receiving double-blind treatment prematurely terminated the trial. The most common primary reasons for discontinuation were AEs (126 subjects, 10.6%) and withdrawal of consent (85 subjects, 7.1%). In total, five subjects (0.4%) discontinued due to pregnancy and the same number due to wish of pregnancy. 16 (1.3%) subjects discontinued due to other reasons, i.e. were lost to follow-up (see Appendix 16.2, Listing 16.2.1.3).

Thus, of 1213 subjects randomised, 938 completed the trial.

Number of subjects (FAS, SS) at each visit is presented in Table 8, a listing of subjects with visit dates is provided in Appendix 16.2, Listing 16.2.1.4.

Table 8: Subjects Disposition by Attended Visit (FAS, SS)

	Test (N=858) n(%)	Reference (N=332) n (%)	Total (N=1190) n (%)
Visit 1a	858 (100)	332 (100)	1190 (100)
Visit 1b	858 (100)	332 (100)	1190 (100)
Visit 2	836 (97.4)	325 (97.9)	1161 (97.6)
Visit 3	776 (90.4)	286 (86.1)	1062 (89.2)
Visit 4	713 (83.1)	256 (77.1)	969 (81.4)
Visit 5/EDV	842 (98.1)	323 (97.3)	1165 (97.9)
Visit 6 (FU)	837 (97.6)	323 (97.3)	1160 (97.5)

Source: Section 15.1, Table 15.1.1.4
N: Number of subjects in specified treatment group
n: Number of subjects with data available
%: Percentage based on N

10.2 Protocol Deviations

In this trial Per Protocol Set was not defined since the Pearl Index (PI) was also calculated as method failure PI, taking into account relevant protocol deviations. Therefore protocol deviations were categorised according to their impact on the perfect medication cycle instead of being classified as minor or major.

During the blind data review meeting the following deviations having an impact on the exposure cycle* were defined:

- Inclusion/exclusion criteria related deviations
- Trial conduct criteria deviations
- Deviations related to missing/incomplete assessments
- Use of prohibited medication (including additional contraception)
- Overall treatment compliance deviations

*Exposure cycle was defined as 28-day cycle, where at least one e-diary entry of IMP intake was available.

Table 9: Deviations Leading to Exclusion from the Method Failure Analysis (FAS)

	Test<br>n (%)	Reference<br>n (%)	Total<br>n (%)
Total number of exposure cycles	6691 (100)	2487 (100)	9178 (100)
Total number of cycles excluded	2050 (30.6)	671 (27.0)	2721 (29.6)
Reasons for exclusion:			
No sexual activity	347 (5.2)	140 (5.6)	487 (5.3)
No assessment of sexual activity	4 (0.1)	3 (0.1)	7 (0.1)
Use of additional contraception	363 (5.4)	117 (4.7)	480 (5.2)
Protocol deviation	1 (0.0)	2 (0.1)	3 (0.0)
Inadequate diary compliance:			
2 or more subsequent pills missed/not documented	1288 (19.2)	389 (15.6)	1677 (18.3)
Missing diary entries for 4 or more separated pills	47 (0.7)	20 (0.8)	67 (0.7)

Source: Section 15.1, Table 15.1.1.6
n: Number of cycles
%: Percentage based on total number of exposure cycles

From the 9178 exposure cycles, a total of 2721 cycles were excluded due to protocol deviations. These deviations leading to exclusion from the method failure analysis are summarised in Table 9. The most common reason for cycle exclusion from the method failure analysis was inadequate diary compliance, which led to exclusion of 1335 cycles (19.9%) in the Test and 409 (16.4%) cycles in the Reference group.

By-subject listings of cycles and protocol deviations having an impact on the cycle are presented in the minutes of the data review meeting (see Appendix 16.1.9).

11. EFFICACY EVALUATION

11.1 Data Sets Analysed

Appendix 16.2, Listing 16.2.1.5 presents a by-subject listing of analysis sets. Table 10 shows the numbers of subjects included in each analysis set.

A total of 1213 subjects were randomised to two treatment groups. No subject unblinding cases, except SUSARs (ectopic pregnancy and hepatic adenoma) for regulatory reporting, took place. The unblinded Safety Manager broke the treatment codes for SUSARs reporting, whereas the blind was maintained for other study personnel and the investigators.

Overall 1190 subjects (858 in the Test group and 332 in the Reference group) were exposed to IMP and had at least one post-baseline safety and at least one efficacy assessment, thus were included in the Safety Set (SS) and in the Full Analysis Set (FAS).

Table 10: Analysis Sets (Randomised Set)

	Test (N=872) n (%)	Reference (N=341) n (%)	Total (N=1213) n (%)
Randomised Set [a]	872 (100)	341 (100)	1213 (100)
Safety Set [b]	858 (98.4)	332 (97.4)	1190 (98.1)
Number of subjects excluded from Safety Set [a]	14 (1.6)	9 (2.6)	23 (1.9)
no administration of the IMP	14 (1.6)	8 (2.3)	22 (1.8)
no post baseline safety assessment	-	1 (0.3)	1 (0.1)
Full Analysis Set [c]	858 (98.4)	332 (97.4)	1190 (98.1)
Number of subjects excluded from Full Analysis Set [a]	14 (1.6)	9 (2.6)	23 (1.9)
subject does not belong to Safety Set	14 (1.6)	9 (2.6)	23 (1.9)
no post baseline efficacy assessment	-	-	-

Source: Section 15.1, Table 15.1.1.5
N: Number of subjects in specified treatment group n: Number of subjects with data available
%: Percentage based on N
Notes:
[a] All randomised subjects
[b] Randomised subjects who had at least one dose of IMP and had at least one post-baseline assessment of any safety/tolerability measurement.
[c] Subjects who were included in the Safety Set and had at least one post-baseline assessment of any efficacy measurement.

11.2 Demographic and Other Baseline Characteristics

Appendix 16.2, Listing 16.2.1.9 presents a by-subject listing of demographics and highest level of education completed. A summary of demographics (FAS, SS) is presented in Table 11.

The mean (SD) subjects' age was 28.9 (7.1) years in each treatment group ranging from 18 to 45 years in the Test and to 44 years in the Reference group. The majority of women (682 subjects, 79.5% in the Test and 259, 78.0% in the Reference group) were 35 years of age or younger. In total, 176 (20.5%) Test group and 73 (22.0%) Reference group subjects were older than 35 years. Two Test group subjects were African (black) and one Reference group subject was Asian. All other subjects were Caucasian.

Over 70% of the FAS subjects in each group had completed high school or had a university degree.

No statistically significant differences between the groups with regard to subject's age (p = 0.9687, 2-sample t test), proportion of subjects in age subgroups (p = 0.5747, chi square

test), ethnicity (p = 0.3351, Fishers exact test) or highest education level completed (p = 0.1300, chi square test) were observed.

Table 11: Demographics (SS, FAS)

		Test (N=858)	Reference (N=332)	P value
Age (years)	n	858	332	
	Mean (SD)	28.9 (7.1)	28.9 (7.1)	0.9687 [a]
	Median	28.0	28.0	
	Q1/ Q3	23.0/34.0	23.0/35.0	
	Min/ Max	18/45	18/44	
Age group [n (%)]	<=35 years	682 (79.5)	259 (78.0)	0.5747 [b]
	>35 years	176 (20.5)	73 (22.0)	
Gender [n (%)]	Male	-	-	
	Female	858 (100)	332 (100)	
Ethnic group [n (%)]	Caucasian	856 (99.8)	331 (99.7)	0.3351 [c]
	African (black)	2 (0.2)	-	
	Asian	-	1 (0.3)	
	Other	-	-	
Highest level of education completed [n (%)]	No school diploma	15 (1.7)	13 (3.9)	0.1300 [b]
	Short-course secondary school	30 (3.5)	16 (4.8)	
	Intermediate secondary school	158 (18.4)	61 (18.4)	
	High school	407 (47.4)	142 (42.8)	
	University degree	248 (28.9)	100 (30.1)	
	Other	-	-	

Source: Section 15.1, Table 15.1.2.1
N: Number of subjects in specified treatment group
n: Number of subjects with data available
%: Percentage based on N
SD: Standard deviation
Q1: 1st Quartile
Q3: 3rd Quartile
Notes: [a] 2-sample t test; [b] Chi square test; [c] Fishers exact test

A summary of substance use (SS) is presented in Table 12. Substance use is listed by subject in Section 16.2, Listing 16.2.1.10.

The majority of trial subjects (67.0% Test and 63.9% Reference group) reported being non-smokers. Ex-smokers comprised 5.4% of the Test group and 5.1% of the Reference group subjects with shorter median non-smoking duration in the Test group compared to the Reference group (28.0 months vs. 37.0 months).

Current smokers comprised 27.6% of the Test group and 31.0% of the Reference group subjects, who reported a mean (SD) number of 8.3 (5.58) and 7.9 (5.75) cigarettes smoked per day, respectively. Only one (0.3%) Reference group subject reported using nicotine replacement therapy.

Approximately 60% of the subjects in each treatment group reported being moderate drinkers. The remaining subjects were abstainers. There were no excessive drinkers in the trial.

In conclusion, no major differences between the treatment groups were observed with regard to substance use.

Table 12: Substance Use at Screening (SS, FAS)

		Test (N=858)	Reference (N=332)	Total (N=1190)
Smoking status [n (%)]	Non-smoker	575 (67.0)	212 (63.9)	787 (66.1)
	Current smoker	237 (27.6)	103 (31.0)	340 (28.6)
	Ex-smoker	46 (5.4)	17 (5.1)	63 (5.3)
Non smoking time (months without smoking)	n	46	17	63
	Mean (SD)	45.9 (47.79)	47.9 (60.06)	46.5 (50.88)
	Median	28.0	37.0	29.0
	Min/ Max	0/173	2/260	0/260
Number of cigarettes per day	n	237	102	339
	Mean (SD)	8.3 (5.58)	7.9 (5.75)	8.2 (5.63)
	Median	8.0	6.0	7.0
	Min/ Max	1/25	1/30	1/30
Nicotine replacement therapy [n (%)]	Yes	-	1 (0.3)	1 (0.1)
	No	858 (100)	331 (99.7)	1189 (99.9)
	Missing	-	-	-
Alcohol consumption [n (%)]	Abstainer	344 (40.1)	135 (40.7)	479 (40.3)
	Moderate drinker	514 (59.9)	197 (59.3)	711 (59.7)
	Excessive drinker	-	-	-

Source: Section 15.1, Table 15.1.2.2
N: Number of subjects in specified treatment group
%: Percentage based on N
n: Number of subjects with data available
SD: Standard deviation

No statistically significant differences in weight ($p = 0.846$), height ($p = 0.439$) or BMI ($p = 0.560$) were observed between the treatment groups at screening (Table 13). P values for the comparison of weight, height and BMI were calculated by means of 2-sample t test.

The proportion of obese subjects (BMI $\geq$ 30 kg/m^2) was comparable between the groups: 30 subjects (3.5%) in the Test and 16 subjects (4.8%) in the Reference group, with no statistically significant difference observed ($p = 0.2884$, chi square test).

No major differences between the groups with regard to vital signs: SBP ($p = 0.738$), DBP ($p = 0.607$) or heart rate ($p = 0.238$) were seen at screening, too (p values were calculated by means of 2-sample t test).

In total, 131 subjects (15.3%) of the Test and 42 subjects (12.7%) of the Reference group had mildly elevated blood pressure, i.e. SBP $\geq$ 130 mmHg or DBP $\geq$ 85 mmHg (but SBP<140 mmHg and DBP<90mmHg, as defined per inclusion criterion). No statistically significant differences were observed between the groups with regard to the proportion of subjects in the respective BP group ($p = 0.2506$, chi square test).

Direct switchers (subjects who had no break between administration of another oral contraceptive and IMP) comprised 73.2% of the Test and 78.0% of the Reference group subjects.

Baseline characteristics are listed by subject in Section 16.2, Listing 16.2.1.11.

Table 13: Baseline Characteristics at Screening (FAS, SS)

		Test (N=858)	Reference (N=332)	Total (N=1190)	P value
**Weight (kg)**	Mean (SD)	63.4 (10.54)	63.3 (11.55)	63.4 (10.83)	0.846 [a]
	Median	61.0	62.0	61.0	
	Q1/ Q3	56.0/69.0	54.3/70.0	56.0/69.0	
	Min/ Max	42/114	42/110	42/114	
**Height (cm)**	Mean (SD)	166.1 (6.08)	166.5 (6.12)	166.2 (6.09)	0.439 [a]
	Median	166.0	167.0	166.0	
	Q1/ Q3	162.0/170.0	163.0/170.0	163.0/170.0	
	Min/ Max	146/186	149/186	146/186	
**BMI (kg/m^2)**	Mean (SD)	22.96 (3.537)	22.82 (3.905)	22.92 (3.642)	0.560 [a]
	Median	22.30	22.05	22.30	
	Q1/ Q3	20.40/24.60	20.00/24.55	20.30/24.60	
	Min/ Max	16.6/41.0	15.9/38.0	15.9/41.0	
**BMI group [n (%)]**	<30 kg/m^2	828 (96.5)	316 (95.2)	1144 (96.1)	0.2884 [b]
	≥30 kg/m^2	30 (3.5)	16 (4.8)	46 (3.9)	
**Systolic blood pressure (mmHg)**	Mean (SD)	115.5 (10.38)	115.3 (9.99)	115.4 (10.27)	0.738 [a]
	Median	116.0	115.0	116.0	
	Q1/ Q3	110.0/123.0	110.0/122.0	110.0/123.0	
	Min/ Max	80/142	76/138	76/142	
**Diastolic blood pressure (mmHg)**	Mean (SD)	72.4 (7.99)	72.1 (7.78)	72.3 (7.93)	0.607 [a]
	Median	72.0	71.0	72.0	
	Q1/ Q3	68.0/80.0	68.0/80.0	68.0/80.0	
	Min/ Max	50/95	53/90	50/95	
**BP group [n (%)]**	SBP<130 mmHg and DBP<85 mmHg	727 (84.7)	290 (87.3)	1017 (85.5)	0.2506 [b]
	SBP≥130 mmHg or DBP≥85 mmHg	131 (15.3)	42 (12.7)	173 (14.5)	
**Heart Rate (bpm)**	n	856	332	1188	
	Mean (SD)	73.5 (9.03)	72.8 (8.23)	73.3 (8.82)	0.238 [a]
	Median	73.0	72.0	72.0	
	Q1/ Q3	67.0/80.0	68.0/78.0	68.0/79.0	
	Min/ Max	51/117	54/98	51/117	
**Number of direct switchers**	n (%)	628 (73.2)	259 (78.0)	887 (74.5)	
**Number of indirect switchers**	n (%)	39 (4.5)	14 (4.2)	53 (4.5)	
**Number of starters**	n (%)	191 (22.3)	59 (17.8)	250 (21.0)	

Source: Section 15.1, Table 15.1.2.3
N: Number of subjects in specified treatment group Q1: 1st Quartile Q3: 3rd Quartile
n: Number of subjects with data available %: Percentage based on N
Notes: [a] 2-sample t test; [b] Chi square test
Direct switcher: No break in administration from another hormonal contraceptive to the IMPs.
Indirect switcher: Break between the administration of another oral contraceptive and IMP is > 2 days and ≤4 months.
Starter: first administration of a hormonal contraceptive or > 4 month break between the administration of another hormonal contraceptive and IMP.

11.3 Medical History

Prior medical history findings were defined as those starting and ending prior to screening. Ongoing medical history findings were defined as those which were ongoing at screening. Medical history was coded using MedDRA version 15.1.

A by-subject listing of medical history is provided in Appendix 16.2, Listing 16.2.1.15. Table 14 summarises the most frequent prior medical history findings by PT.

Prior findings occurring in ≥ 5% of subjects in any treatment group were surgical and medical procedures (range 11.4% Test to 14.2% Reference subjects), reproductive system and breast disorders (7.3% Test to 9.0% Reference) and infections and infestations (6.1% Test and 5.7% Reference), see Section 15.2, Table 15.1.2.7.

A total of 201 (23.4%) Test group subjects and 91 (27.4%) Reference group subjects reported at least one prior medical history finding. The most common previous medical or surgical history finding in both groups was Caesarean section (range: 8.0% Test to 11.1% Reference), followed by dysmenorrhoea in the Test group (2.4% Test and 2.1% Reference) and by vaginal infection in the Reference group (2.7% of Reference and 1.6% of Test group subjects).

Table 14: Prior Medical History Findings, Frequency ≥ 2.0% of Subjects in any Treatment Group (FAS, SS)

Preferred Term	Test (N=858) n (%)	Reference (N=332) n (%)	Total (N=1190) n (%)
Number of subjects with at least one prior medical history finding	201 (23.4)	91 (27.4)	292 (24.5)
Caesarean section	69 (8.0)	37 (11.1)	106 (8.9)
Dysmenorrhoea	21 (2.4)	7 (2.1)	28 (2.4)
Vaginal infection	14 (1.6)	9 (2.7)	23 (1.9)
Breast pain	10 (1.2)	7 (2.1)	17 (1.4)
Cervical dysplasia	9 (1.0)	7 (2.1)	16 (1.3)

Source: Section 15.1, Table 15.1.2.7
N: Number of subjects in specified treatment group
n: Number of subjects with data available
%: Percentage based on N

Table 15 summarises the most common ongoing medical history findings at screening (FAS, SS). A total of 314 (36.6%) Test group and 129 (38.9%) Reference group subjects reported at least one ongoing finding. The most frequent findings were dysmenorrhoea (17.6% of the Test and 17.2% of the Reference group subjects), breast pain (10.0% Test and 12.7% Reference) and obesity (2.9% Test and 4.5% Reference).

The proportions of subjects with ongoing medical and surgical findings were comparable between the treatment groups in the FAS and SS populations.

Table 15: Ongoing Medical Findings, Frequency ≥ 2.0% of Subjects in any Treatment Group (FAS, SS)

Preferred Term	Test (N=858) n (%)	Reference (N=332) n (%)	Total (N=1190) n (%)
Number of subjects with at least one prior medical history finding	314 (36.6)	129 (38.9)	443 (37.2)
Dysmenorrhoea	151 (17.6)	57 (17.2)	208 (17.5)
Breast pain	86 (10.0)	42 (12.7)	128 (10.8)
Obesity	25 (2.9)	15 (4.5)	40 (3.4)
Hypothyroidism	30 (3.5)	5 (1.5)	35 (2.9)
Headache	20 (2.3)	5 (1.5)	25 (2.1)

Source: Section 15.1, Table 15.1.2.8
N: Number of subjects in specified treatment group
n: Number of subjects with data available
%: Percentage based on N

At screening the investigators assessed the subjects‘ indivdual VTE risk/benefit ratio. If the ratio was considered unfavourable for the subject, the subject was not to be included in the trial. The summary of subjects with VTE risk factors is provided in Table 16 below.

No major differences between the treatment groups were observed with regard to the VTE factors assessed. The majority of subjects (83.4% in the Test and 82.2% in the Reference group) were assessed by the investigators as having no VTE risk factors. Two risk factors were documented for seven (0.6%) subjects: three (0.3%) subjects in the Test group and four (1.2%) subjects in the Reference group.

Data on VTE risk factors are listed by subject in Appendix 16.2, Listing 16.2.17.

Table 16: VTE Risk Factors (FAS, SS)

		Test (N=858) n (%)	Reference (N=362) n (%)	Total (N=1190) n (%)
Family history of thromboembolic illness, e.g. deep venous or arterial thrombosis in one of the siblings or parents at the age < 55 years	Yes	12 (1.4)	6 (1.8)	18 (1.5)
	No	846 (98.6)	326 (98.2)	1172 (98.5)
Evidence of predisposing conditions for a vascular or metabolic disease	Yes	-	-	-
	None	853 (99.4)	329 (99.1)	1182 (99.3)
	Miss	5 (0.6)	3 (0.9)	8 (0.7)
Current smoker older than 35 years or non-smoker over 40 years old	Yes	103 (12.0)	41 (12.3)	144 (12.1)
	No	755 (88.0)	291 (87.7)	1046 (87.9)
Body weight so that BMI > 30 kg/m^2	Yes	30 (3.5)	16 (4.8)	46 (3.9)
	No	828 (96.5)	316 (95.2)	1144 (96.1)
Number of VTE risk factors	0	716 (83.4)	273 (82.2)	989 (83.1)
	1	139 (16.2)	55 (16.6)	194 (16.3)
	2	3 (0.3)	4 (1.2)	7 (0.6)
	≥3	-	-	-

Source: Section 15.1, Table 15.1.2.10
N: Number of subjects in specified treatment group
n: Number of subjects with data available
%: Percentage based on N

11.4 Gynaecological, Obstetric and Menstrual History

A by-subject listing of gynaecological, obstetric and menstrual history is provided in Appendix 16.2, Listing 16.2.1.16. Table 17 summarises gynaecological, obstetric and menstrual history (FAS, SS).

The mean and median subjects' age at menarche was 13.0 years in both treatment groups. In total 395 women (46.0%) in the Test and 150 women (45.2%) in the Reference group reported having had at least one delivery. Approximately 50% of these reported one delivery, whereas three or more deliveries were reported by 28 (7.1%) Test group and six (4.0%) Reference group subjects.

Miscarriages were reported by 92 subjects (7.7%), and abortions by 189 subjects (15.9%). Three or more miscarriages were reported only in the Test group (four subjects, 5.9%), whereas three or more abortions were reported by nine (6.9%) Test group and five (8.6%) Reference group subjects.

Table 17: Gynaecological, Obstetric and Menstrual History (FAS, SS)

		Test (N=858) n (%)	Reference (N=332) n (%)	Total (N=1190) n (%)
**Subject's age at menarche (years)**	n	858	332	1190
	Mean (SD)	13.0 (1.24)	13.0 (1.37)	13.0 (1.28)
	Median	13.0	13.0	13.0
	Min/ Max	9/17	9/20	9/20
**Any deliveries [n (%)]**	Yes	395 (46.0)	150 (45.2)	545 (45.8)
	No	463 (54.0)	182 (54.8)	645 (54.2)
**Number of deliveries* [n (%)]**	1 delivery	196 (49.6)	78 (52.0)	274 (50.3)
	2 deliveries	171 (43.3)	66 (44.0)	237 (43.5)
	3 and more deliveries	28 (7.1)	6 (4.0)	34 (6.2)
**Any miscarriages [n (%)]**	Yes	68 (7.9)	24 (7.2)	92 (7.7)
	No	790 (92.1)	308 (92.8)	1098 (92.3)
**Number of miscarriages * [n (%)]**	1 miscarriage	53 (77.9)	21 (87.5)	74 (80.4)
	2 miscarriages	11 (16.2)	3 (12.5)	14 (15.2)
	3 and more miscarriages	4 (5.9)	-	4 (4.3)
**Any abortion [n (%)]**	Yes	131 (15.3)	58 (17.5)	189 (15.9)
	No	727 (84.7)	274 (82.5)	1001 (84.1)
**Number of abortions * [n (%)]**	1 abortion	91 (69.5)	41 (70.7)	132 (69.8)
	2 abortions	31 (23.7)	12 (20.7)	43 (22.8)
	3 and more abortions	9 (6.9)	5 (8.6)	14 (7.4)

Source: Section 15.1, Table 15.1.2.9
N: Number of subjects in specified treatment group
n: Number of subjects with data available
%: Percentage based on N
*: Percentage based on total number of subjects with at least one delivery/miscarriage/abortion
SD: Standard deviation

Scheduled/regular bleeding

A summary of scheduled/regular bleeding during the last six cycles prior to screening is provided in Table 18. The by-subject listing of previous bleeding results documented at screening is provided in Appendix 16.2, Listing 16.2.1.12.

No statistically significant differences were observed with regard to relevant bleeding characteristics. Regular/scheduled bleeding during six cycles prior to screening was reported

by the vast majority of subjects (range 91.6% in the Test group to 91.9% in the Reference group, p = 0.7488, chi square test).

Absence of more than one scheduled bleeding during the last six cycles before screening was reported by seven (0.8%) Test group and six (1.8%) Reference group subjects (p = 0.2955, chi square test). The proportion of subjects in each bleeding intensity category (slight, moderate or heavy) were similar in both groups, with moderate intensity of scheduled bleeding prevailing, reported by 68.9% of the Test and 70.2% of the Reference group subjects (p = 0.8015, chi square test).

Table 18: Scheduled/Regular Bleeding Characteristics Before Screening (Randomised Set)

		Test (N=858)	Reference (N=332)	Total (N=1190)	P value
**Time since last scheduled/menstrual bleeding (days)**	n	805	314	1119	
	Mean (SD)	16.0 (7.9)	17.9 (9.4)	16.5 (8.4)	<0.001 [a]
	Median	15.0	17.0	16.0	
	Q1/ Q3	10.0/21.0	11.0/23.0	10.0/22.0	
	Min/ Max	1/69	1/95	1/95	
**Regular scheduled/menstrual bleeding during last 6 cycles [n (%)]**	Yes	786 (91.6)	305 (91.9)	1091 (91.7)	0.7488 [b]
	No	21 (2.4)	10 (3.0)	31 (2.6)	
	Missing	51 (5.9)	17 (5.1)	68 (5.7)	
**Intensity of scheduled/menstrual bleeding during last 6 cycles [n (%)]**	Slight	173 (20.2)	69 (20.8)	242 (20.3)	0.8015 [b]
	Moderate	591 (68.9)	233 (70.2)	824 (69.2)	
	Heavy	43 (5.0)	13 (3.9)	56 (4.7)	
	Missing	51 (5.9)	17 (5.1)	68 (5.7)	
**Spotting in the last 6 cycles [n (%)]**	Yes	20 (2.3)	13 (4.0)	33 (2.8)	0.2227 [b]
	No	832 (97.7)	315 (96.0)	1147 (97.2)	
	Missing	6 (0.7)	4 (1.2)	10 (0.8)	
**Unscheduled bleeding in the last 6 cycles [n (%)]**	Yes	9 (1.0)	7 (2.1)	16 (1.3)	0.2950 [b]
	No	842 (98.1)	321 (96.7)	1163 (97.7)	
	Missing	7 (0.8)	4 (1.2)	11 (0.9)	
***Intensity of unscheduled bleeding [n (%)]**	Slight	7 (77.8)	5 (71.4)	12 (75.0)	0.4870 [b]
	Moderate	2 (22.2)	1 (14.3)	3 (18.8)	
	Heavy	0	1 (14.3)	1 (6.3)	
	Missing	0	0	0	
**Absence of more than one scheduled/menstrual bleeding in the last 6 cycles [n (%)]**	Yes	7 (0.8)	6 (1.8)	13 (1.1)	0.2955 [b]
	No	800 (93.2)	309 (93.1)	1109 (93.2)	
	Missing	51 (5.9)	17 (5.1)	68 (5.7)	

Source: Section 15.1, Table 15.1.2.4

N: Number of subjects in specified treatment group
n: Number of subjects with data available
%: Percentage based on N
SD: Standard deviation
Q1: 1st Quartile
Q3: 3rd Quartile

Notes: * Percentage based on subjects with unscheduled bleeding in the last 6 cycles

[a] 2-sample t test; [b] Chi square test

The incidence of previous spotting was low and was reported by 20 (2.3%) Test group and 13 (4.0%) Reference group subjects (p = 0.2227, chi square test).

Unscheduled bleeding was uncommon and was experienced by nine (1.0%) of the Test and seven (2.1%) of the Reference group subjects (p = 0.2950, chi square test). Unscheduled bleeding of heavy intensity was reported by one Reference group subject.

Dysmenorrhoea

The summary of subjects with dysmenorrhoea during the last six cycles prior to screening and at follow-up is presented in Table 19. A by-subject listing with dysmenorrhoea characteristics is presented in Appendix 16.2, Listing 16.2.1.13.

At screening, 232 (19.6% of the Test and 19.3% of the Reference group subjects) of the FAS subjects reported that they had suffered from dysmenorrhoea within six cycles prior to V1a. At follow-up, only these subjects were asked about the intensity of dysmenorrhoea and pain medications used since Visit 1a. A total of 150, i.e. more than a half of these subjects, reported having no dysmenorrhoea anymore.

Mild and moderate intensity of dysmenorrhoea prevailed before the study, whereas at follow up the majority of subjects had dysmenorrhoea of mild intensity. Severe intensity of dysmenorrhoea within six cycles prior to V1a was documented for higher percentage of the Test group subjects (34 subjects, 20.2%) compared to the Reference group (seven subjects, 10.9%). At follow-up severe dysmenorrhoea was reported only for one Test group and two Reference group subjects.

Table 19: Dysmenorrhoea Characteristics at Screening and at Follow-up (FAS, SS)

Visit			Test (N=858) n (%)	Reference (N=332) n (%)	Total (N=1190) n (%)
Visit 1a	Suffering from dysmenorrhoea within the last 6 cycles	Yes	168 (19.6)	64 (19.3)	232 (19.5)
		No	690 (80.4)	268 (80.7)	958 (80.5)
	Intensity of dysmenorrhoea*	Mild	70 (41.7)	30 (46.9)	100 (43.1)
		Moderate	64 (38.1)	27 (42.2)	91 (39.2)
		Severe	34 (20.2)	7 (10.9)	41 (17.7)
	Use of pain medication*	Yes	49 (29.2)	15 (23.4)	64 (27.6)
		No	119 (70.8)	49 (76.6)	168 (72.4)
Follow-up	Intensity of dysmenorrhoea*	None	104 (61.9)	46 (71.9)	150 (64.7)
		Mild	42 (25.0)	12 (18.8)	54 (23.3)
		Moderate	6 (3.6)	0	6 (2.6)
		Severe	1 (0.6)	2 (3.1)	3 (1.3)
		Missing	15 (8.9)	4 (6.3)	19 (8.2)
	Use of pain medication*	Yes	8 (4.8)	3 (4.7)	11 (4.7)
		No	142 (84.5)	56 (87.5)	198 (85.3)
		Missing	18 (10.7)	5 (7.8)	23 (9.9)

Source: Section 15.1, Table 15.1.2.5
N: Number of subjects in specified treatment group
n: Number of subjects with data available
%: Percentage based on N
*: Percentage is based on the number of subjects suffering from dysmenorrhoea

Medication for pain relief was used by 64 subjects (27.6%) prior to screening (29.2% of the Test and 23.4% of the Reference group subjects). At follow-up only eight (4.8%) Test group and three (4.7%) Reference group subjects took medication for pain relief.

Mastodynia/mastalgia

In total, 145 FAS subjects (range: 11.2% Test to 14.8% Reference) experienced mastodynia/mastalgia within six cycles prior to the screening (see Table 20). Of these, 81 subjects had no mastodynia/mastalgia at follow up. Mastodynia/mastalgia of severe intensity was reported by five subjects (three, 3.1% Test and two, 4.1% Reference group subjects) at screening, whereas none of the subjects reported having severe mastodynia/mastalgia at follow-up. Only two (2.1%) Test group subjects took medication for pain relief at screening and none of the subjects took pain medication at follow up.

Data on mastodynia during six cycles before screening and at follow-up are listed by subject in Appendix 16.2, Listing 6.2.1.14.

Table 20: Mastodynia/Mastalgia Characteristics at Screening and at Follow-up (FAS, SS)

Visit			Test (N=858) n (%)	Reference (N=332) n (%)	Total (N=1190) n (%)
Visit 1a	Suffering from mastodynia/ mastalgia within the last 6 cycles	Yes	96 (11.2)	49 (14.8)	145 (12.2)
		No	762 (88.8)	283 (85.2)	1045 (87.8)
	Intensity of mastodynia/ mastalgia*	Mild	63 (65.6)	24 (49.0)	87 (60.0)
		Moderate	30 (31.3)	23 (46.9)	53 (36.6)
		Severe	3 (3.1)	2 (4.1)	5 (3.4)
	Use of pain medication*	Yes	2 (2.1)	0	2 (1.4)
		No	94 (97.9)	49 (100)	143 (98.6)
Follow-up	Intensity of mastodynia/ mastalgia*	None	51 (53.1)	30 (61.2)	81 (55.9)
		Mild	37 (38.5)	15 (30.6)	52 (35.9)
		Moderate	3 (3.1)	1 (2.0)	4 (2.8)
		Severe	0	0	0
		Missing	5 (5.2)	3 (6.1)	8 (5.5)
	Use of pain medication*	Yes	0	0	0
		No	91 (94.8)	46 (93.9)	137 (94.5)
		Missing	5 (5.2)	3 (6.1)	8 (5.5)

Source: Section 15.1, Table 15.1.2.6
N: Number of subjects in specified treatment group
n: Number of subjects with data available
%: Percentage based on N
* Percentage is based on the number of subjects suffering from mastodynia/mastalgia

11.5 Prior medications, including contraceptive methods

Prior medications include all medications with the stop date before the date of first IMP intake. A by-subject listing of prior medication is provided in Appendix 16.2, Listing 16.2.1.18. The most frequently used prior medications by ATC 2nd level (RS) are summarised in Table 21, by substance name WHO DD in Table 22. Medications were coded by the WHO-DD version March 2013.

In total, 992 (83.4%) of FAS subjects reported at least one prior medication or contraceptive method (range: 82.1% Test to 86.7% Reference group subjects). The most common prior medications were sex hormones and modulators of the genital system, used by 664 (55.8%) of subjects (range: 54.7% Test to 58.7% Reference group subjects).

Table 21: Prior Medications, Therapies, Contraceptives by ATC 2nd Level, Frequency ≥ 2.0% of Subjects in any Treatment Group (FAS, SS)

ATC 2nd level subgroup [a]	Test (N=858) n (%)	Reference (N=332) n (%)	Total (N=1190) n (%)
**Number of subjects with any prior medication or contraceptive**	704 (82.1)	288 (86.7)	992 (83.4)
Sex hormones and modulators of the genital system	469 (54.7)	195 (58.7)	664 (55.8)
Uncoded*	214 (24.9)	90 (27.1)	304 (25.5)
Antiinflammatory and antirheumatic products	36 (4.2)	12 (3.6)	48 (4.0)
Gynecological antiinfectives and antiseptics	30 (3.5)	17 (5.1)	47 (3.9)
Antibacterials for systemic use	29 (3.4)	5 (1.5)	34 (2.9)
Analgesics	28 (3.3)	6 (1.8)	34 (2.9)
Other gynecologicals	19 (2.2)	5 (1.5)	24 (2.0)

Source: Section 15.1, Table 15.1.2.11
N: Number of subjects in specified treatment group
n: Number of subjects with data available
%: Percentage based on N
Notes: [a] A subject may have taken more than one medication in any category.
*„Uncoded“ included condoms and sexual abstinence

Table 22: Prior Medications, Therapies, Contraceptives by Generic Name WHO DD, Frequency ≥ 2.0% of Subjects in any Group (FAS, SS)

Generic Name WHO DD	Test (N=858) n (%)	Reference (N=332) n (%)	Total (N=1190) n (%)
Uncoded*	214 (24.9)	90 (27.1)	304 (25.5)
Drospirenone with ethinylestradiol	93 (10.8)	41 (12.3)	134 (11.3)
Ethinylestradiol with gestodene	62 (7.2)	29 (8.7)	91 (7.6)
Drospirenone with ethinylestradiol betadex clathrate	68 (7.9)	20 (6.0)	88 (7.4)
Desogestrel with ethinylestradiol	56 (6.5)	19 (5.7)	75 (6.3)
Ethinylestradiol with levonorgestrel	50 (5.8)	20 (6.0)	70 (5.9)
Desogestrel	44 (5.1)	17 (5.1)	61 (5.1)
Dienogest with ethinylestradiol	39 (4.5)	15 (4.5)	54 (4.5)
Ibuprofen	25 (2.9)	10 (3.0)	35 (2.9)
Cyproterone acetate with ethinylestradiol	17 (2.0)	15 (4.5)	32 (2.7)
Ethinylestradiol with norgestimate	22 (2.6)	7 (2.1)	29 (2.4)
Chlormadinone acetate with ethinylestradiol	15 (1.7)	10 (3.0)	25 (2.1)
Paracetamol	17 (2.0)	3 (0.9)	20 (1.7)
Clotrimazole	9 (1.0)	7 (2.1)	16 (1.3)

Source: Section 15.1, Table 15.1.2.11
N: Number of subjects in in specified treatment group
n: Number of subjects with data available %: Percentage based on N
*„Uncoded“ included condoms and sexual abstinence

The most common prior medications by substance name were: drospirenone with ethinylestradiol (10.8% Test to 12.3% Reference group subjects), ethinylestradiol with gestodene (7.2% Test to 8.7% Reference group subjects) and drospirenone with ethinylestradiol betadex clathrate (7.9% Test and 6.0% Reference group subjects).

11.6 Concomitant medications

Concomitant medications included all medications taken after the start of IMP and those medications which started prior to the first intake of IMP and were continued after the start of IMP. Medications were coded using WHO and Anatomical Therapeutic Chemical (ATC)

classification system (WHO-DD Version March 2013). A by-subject listing of concomitant medication is provided in Appendix 16.2, Listing 16.2.1.19.

Approximately 30% of subjects in each treatment group reported intake of at least one concomitant medication (Table 23). The most common concomitant medications by ATC 2nd level were analgesics (7.5% Test to 8.4% Reference group subjects), antibacterials for systemic use (6.9% of each group subjects), and antiinflammatory and antirheumatic products (6.8% of Test and 6.9% of Reference group subjects).

Table 23: Concomitant Medications, Therapies, Contraceptives by ATC 2nd Level, Frequency ≥ 2.0% of Subjects in any Treatment Group (FAS, SS)

ATC 2nd level subgroup	Test (N=858) n (%)	Reference (N=332) n (%)	Total (N=1190) n (%)
**Number of subjects with any prior medication [b, c]**	259 (30.2)	116 (34.9)	375 (31.5)
Analgesics	64 (7.5)	28 (8.4)	92 (7.7)
Antibacterials for systemic use	59 (6.9)	23 (6.9)	82 (6.9)
Antiinflammatory and antirheumatic products	58 (6.8)	23 (6.9)	81 (6.8)
Thyroid therapy	35 (4.1)	8 (2.4)	43 (3.6)
Gynecological antiinfectives and antiseptics	29 (3.4)	12 (3.6)	41 (3.4)
Drugs for functional gastrointestinal disorders	13 (1.5)	12 (3.6)	25 (2.1)
Antihistamines for systemic use	18 (2.1)	6 (1.8)	24 (2.0)

Source: Section 15.1, Table 15.1.2.12
N: Number of subjects in specified treatment group
n: Number of subjects with data available
%: Percentage based on N

As shown in Table 24 below, the most frequently used concomitant medications by generic name were ibuprofen (5.0% of the Test and 5.1% of the Reference group subjects) and paracetamol (4.8% of the Test to 5.7% of the Reference group subjects).

Table 24: Concomitant Medications by Substance Name, Frequency ≥ 2.0% of Subjects (FAS, SS)

Generic Name WHO DD	Test (N=858) n (%)	Reference (N=332) n (%)	Total (N=1190) n (%)
Ibuprofen	43 (5.0)	17 (5.1)	60 (5.0)
Paracetamol	41 (4.8)	19 (5.7)	60 (5.0)
Levothyroxine sodium	29 (3.4)	8 (2.4)	37 (3.1)

Source: Section 15.1, Table 15.1.2.12
N: Number of subjects in specified treatment group
n: Number of subjects with data available
%: Percentage based on N

Antibiotics by generic name (antibacterials for systemic use by ATC 2nd level) used by at least 0.5% of subjects in any treatment group are listed in Table 25, whereas treatment duration with each of these antibiotics is provided in Table 26. In total, 82 (6.9%) of FAS subjects took concomitant antibiotics. The most frequently used concomitant antibiotics were amoxicillin (0.9% Test and 0.6% Reference group subjects), azithromycin (0.9% Test and 0.3% Reference group subjects) and amoxicillin trihydrate (0.6% Test and 0.9% Reference group subjects). Treatment duration with the most frequently used antibiotics varied from one to 30 days.

Table 25: Concomitant Antibiotics by Substance Name, Frequency> 0.5% of Subjects in any Treatment Group (FAS, SS)

ATC 2nd level subgroup [a]<br>Generic Name WHO DD	Test (N=858) n (%)	Reference (N=332) n (%)	Total (N=1190) n (%)
**Antibacterials For Systemic Use**	59 (6.9)	23 (6.9)	82 (6.9)
Amoxicillin	8 (0.9)	2 (0.6)	10 (0.8)
Azithromycin	8 (0.9)	1 (0.3)	9 (0.8)
Amoxicillin trihydrate	5 (0.6)	3 (0.9)	8 (0.7)
Amoxicillin sodium w/clavulanate potassium	6 (0.7)	1 (0.3)	7 (0.6)
Cefuroxime	6 (0.7)	0	6 (0.5)
Fosfomycin trometamol	5 (0.6)	1 (0.3)	6 (0.5)

Source: Section 15.1, Table 15.1.2.13
N: Number of subjects in specified treatment group
n: Number of subjects with data available
%: Percentage based on N
Notes: [a] A subject may have taken more than one medication in any category.

Table 26: Concomitant Antibiotics Use Duration in Days (FAS, SS)

Generic Name WHO DD		Test (N=858)	Reference (N=332)	Total (N=1190)
Amoxicillin	Mean (SD)	11.0 (7.52)	7.0 (2.83)	10.3 (6.97)
	Median	8.0	7.0	8.0
	Q1/ Q3	7.0/11.0	5.0/9.0	7.0/11.0
	Min/ Max	7/30	5/9	5/30
Azithromycin	Mean (SD)	4.4 (2.50)	3.0 (.)	4.2 (2.39)
	Median	3.0	3.0	3.0
	Q1/ Q3	3.0/5.0	3.0/3.0	3.0/4.0
	Min/ Max	3/10	3/3	3/10
Amoxicillin trihydrate	Mean (SD)	8.0 (3.00)	7.7 (2.52)	7.9 (2.64)
	Median	8.0	8.0	8.0
	Q1/ Q3	5.0/11.0	5.0/10.0	5.0/10.5
	Min/ Max	5/11	5/10	5/11
Amoxicillin sodium w/clavulanate potassium	Mean (SD)	6.5 (1.22)	8.0 (.)	6.7 (1.25)
	Median	7.0	8.0	7.0
	Q1/ Q3	7.0/7.0	8.0/8.0	7.0/7.0
	Min/ Max	4/7	8/8	4/8
Cefuroxime	Mean (SD)	7.6 (3.42)	0	7.6 (3.42)
	Median	9.0	0	9.0
	Q1/ Q3	5.5/10.0	0	5.5/10.0
	Min/ Max	1/11	0	1/11
Fosfomycin trometamol	Mean (SD)	1.0 (0.00)	1.0 (.)	1.0 (0.00)
	Median	1.0	1.0	1.0
	Q1/ Q3	1.0/1.0	1.0/1.0	1.0/1.0
	Min/ Max	1/1	1/1	1/1

Source: Section 15.1, Table 15.1.2.14
N: Number of subjects in specified treatment group
n: Number of subjects with data available
%: Percentage based on N
SD: Standard deviation
Q1: 1st Quartile; Q3: 3rd Quartile

11.7 Measurements of Treatment Compliance

A by-subject listing of exposure to IMP based on tablet count and individual treatment compliance is provided in Appendix 16.2, Listing 16.2.1.6.

Table 27 summarises overall compliance to trial medication (FAS, SS). The mean (SD) overall compliance was similar in both treatment groups: 101.7 (14.13)% in the Test and

101.9 (5.93)% in the Reference group, the median value was 100.0% in each group. One missed tablet was reported for four (0.5%) Test and one (0.3%) Reference group subjects, four or more missed tablets were reported for slightly higher proportion of the Test group subjects (73 subjects, 8.5%) than the Reference group subjects (18 subjects, 5.4%).

Compliance was calculated based on the numbers of dispensed and returned tablets. Compliance above 100% was also achieved not because the subjects took more tablets than prescribed, but due to unreturned tablets or blisters. E.g. for the Test group Subject #370015 with the maximum compliance of 350%, 112 tablets were dispensed, her treatment period lasted eight days. She lost a blister and returned 56 tablets only.

Table 27: Compliance (%) to Investigational Medicinal Product (FAS, SS)

		Test (N=858)	Reference (N=332)	Total (N=1190)
Overall compliance	n	851	332	1183
	Mean (SD)	101.7 (14.13)	101.9 (5.93)	101.5 (12.39)
	Median	100.0	100.0	100.0
	Q1/ Q3	100.0/100.0	100.0/100.0	100.0/100.0
	Min/ Max	74/350	89/158	74/350
Missing pill category [n (%)]	1 pill missing	4 (0.5)	1 (0.3)	5 (0.4)
	2 pills missing	45 (5.2)	21 (6.3)	66 (5.5)
	3 pills missing	-	1 (0.3)	1 (0.1)
	4 and more pills missing	73 (8.5)	18 (5.4)	91 (7.6)

Source: Section 15.1, Table 15.1.2.16
N: Number of subjects in specified treatment group
n: Number of subjects with data available
SD: Standard deviation

11.8 Efficacy Results and Tabulations of Individual Subject Data

11.8.1 Analysis of efficacy

11.8.1.1 Overall Pearl Index

In total, six in-treatment pregnancies (five in the Test and one in the Reference group) occurred. For more detailed information about these and other reported pregnancies, see Section 12.4. The calculation of the overall PI included pregnancies classified as method failure or user failure. In this trial, all pregnancies were considered a result of method failure,
i.e. the subject was treatment-compliant near the time of conception and the estimated date of conception was during the treatment period, extended by a maximum of two days. A by- subject listing of cycles’ status is provided in Appendix 16.2, Listing 16.3.1.3.

In the Test group, 858 subjects with 6691 drospirenone treatment cycles (Table 28), and in the Reference group, 332 subjects with 2487 desogestrel treatment cycles were analysed.

During these cycles five Test group and one Reference group subjects became pregnant, leading to an overall Pearl Index of 0.9715, 95% CI (0.3154; 2.2671) in the Test group and 0.5227, 95% CI (0.0132; 2.9124) in the Reference group, see Table 29. The PI point estimate for the Test group was higher than for the Reference group. The PI calculation for the Reference group, however, was less precise, as it was based on a considerably lower number of cycles, resulting in a much wider confidence interval. The upper limit of the PI 95% CI was lower for the Test group than for the Reference group.

Table 28 Exposure Cycles (FAS)

Cycle	Test (N=858) n/m (%)	Reference (N=332) n/m (%)	Total (N=1190) n/m (%)
Total number of cycles	6691	2487	9178
Cycle 1	835 / 858 (97.3)	326 / 332 (98.2)	1161 / 1190 (97.6)
Cycle 2	817 / 830 (98.4)	313 / 325 (96.3)	1130 / 1155 (97.8)
Cycle 3	785 / 816 (96.2)	291 / 304 (95.7)	1076 / 1120 (96.1)
Cycle 4	759 / 776 (97.8)	281 / 286 (98.3)	1040 / 1062 (97.9)
Cycle 5	732 / 755 (97.0)	271 / 279 (97.1)	1003 / 1034 (97.0)
Cycle 6	714 / 734 (97.3)	262 / 269 (97.4)	976 / 1003 (97.3)
Cycle 7	695 / 713 (97.5)	252 / 259 (97.3)	947 / 972 (97.4)
Cycle 8	690 / 701 (98.4)	249 / 251 (99.2)	939 / 952 (98.6)
Cycle 9	664 / 697 (95.3)	242 / 250 (96.8)	906 / 947 (95.7)

Source: Section 15.1, Table 15.2.1.1
N: Number of subjects in specified treatment group
n: Number of subjects with data available
m: Number of subjects in the cycle
%: Percentage based on N

Table 29 Overall Pearl Index (FAS)

	Test (N=858)	Reference (N=332)
Pregnancy [n (%)]		
Yes	5 (0.6%)	1 (0.3%)
No	853 (99.4%)	331 (99.7%)
Total number of exposure cycles [n]	6691	2487
Overall Pearl Index	0.9715	0.5227
95% Confidence Interval (Lower limit/ Upper limit)	0.3154/2.2671	0.0132/2.9124

Source: Section 15.1, Table 15.2.1.7
N: Number of subjects in specified treatment group
n: Number of cycles/subjects with data available
%: Percentage based on N

The corresponding overall PI estimate for the age subgroup ≤ 35 years is provided below in Table 30.

All six pregnancies were reported for subjects aged 35 years or younger. The overall PI (95% CI) for women ≤ 35 years was 1.2428 (0.4035; 2.9004) (number of cycles: 5230) in the Test group and 0.6767 (0.0171; 3.7705) (number of cycles 1921) in the Reference group. The PI calculation for the Reference group, however, was less precise, as it was based on a considerably lower number of cycles, resulting in a much wider confidence interval. The upper limit of the PI 95% CI was lower for the Test group than for the Reference group.

Table 30 Overall Pearl Index for Subjects ≤ 35 Years (FAS)

	Test (N=682)	Reference (N=259)
Pregnancy [n (%)]		
Yes	5 (0.7%)	1 (0.4%)
No	677 (99.3%)	258 (99.6%)
Total number of exposure cycles [n]	5230	1921
Overall Pearl Index	1.2428	0.6767
95% Confidence Interval (Lower limit/ Upper limit)	0.4035/2.9004	0.0171/3.7705

Source: Section 15.1, Table 15.2.1.8
N: Number of subjects in specified treatment group and subgroup
n: Number of cycles/subjects with data available
%: Percentage based on N

Overall Pearl Index after correction for additional contraception and sexual activity

The Pearl Index was also calculated after correction for additional contraception and sexual activity status. There were 5977 cycles in the Test group and 2224 cycles in the Reference group analysed, excluding cycles with additional contraception and those without sexual activity (Table 31).

Table 31: Sexual Activity Cycles Without Additional Contraception (FAS)

Cycle	Test (N=858) n/m (%)	Reference (N=332) n/m (%)	Total (N=1190) n/m (%)
Total number of sexual activity cycles without additional contraception	5977	2224	8201
Cycle 1	651 / 858 (75.9)	252 / 332 (75.9)	903 / 1190 (75.9)
Cycle 2	718 / 830 (86.5)	271 / 325 (83.4)	989 / 1155 (85.6)
Cycle 3	704 / 816 (86.3)	258 / 304 (84.9)	962 / 1120 (85.9)
Cycle 4	687 / 776 (88.5)	255 / 286 (89.2)	942 / 1062 (88.7)
Cycle 5	671 / 755 (88.9)	251 / 279 (90.0)	922 / 1034 (89.2)
Cycle 6	652 / 734 (88.8)	242 / 269 (90.0)	894 / 1003 (89.1)
Cycle 7	646 / 713 (90.6)	234 / 259 (90.3)	880 / 972 (90.5)
Cycle 8	635 / 701 (90.6)	230 / 251 (91.6)	865 / 952 (90.9)
Cycle 9	613 / 697 (87.9)	231 / 250 (92.4)	844 / 947 (89.1)

Source: Section 15.1, Table 15.2.1.4
N: Number of subjects in specified treatment group
n: Number of subjects with data available
m: number of subjects in the cycle
%: Percentage based on m

The overall PI (95% CI) after correction for additonal contraception and sexual activity status was 1.0875 (0.3531; 2.5379) for the Test group subjects, and 0.5845 (0.0148; 3.2568) for the Reference group subjects, see Table 32.

Table 32 Overall Pearl Index After Correction for Additional Contraception and Sexual Activity Status (FAS)

	Test (N=858)	Reference (N=332)
Pregnancy [n (%)]		
Yes	5 (0.6%)	1 (0.3%)
No	853 (99.4%)	331 (99.7%)
Total number of sexual activity cycles without backup contaception [n]	5977	2224
Overall Pearl Index	1.0875	0.5845
95% Confidence Interval (Lower limit/Upper limit)	0.3531/2.5379	0.0148/3.2568

Source: Section 15.1, Table 15.2.2.1
N: Number of subjects in specified treatment group
n: Number of cycles/subjects with data available
%: Percentage based on N

The corresponding overall PI (95% CI) after correction for additional contraception and sexual activity status for the subgroup of subjects aged 35 years or younger was 1.4000 (0.4546; 3.2670) in the Test group and 0.7598 (0.0192; 4.2333) in the Reference group, see Table 33.

Table 33 Overall Pearl Index After Correction for Additional Contraception and Sexual Activity Status, Age Group ≤ 35 Years (FAS)

	Test (N=682)	Reference (N=259)
Pregnancy [n (%)]		
Yes	5 (0.7%)	1 (0.4%)
No	677 (99.3%)	258 (99.6%)
Total number of sexual activity cycles without backup contaception [n]	4643	1711
Overall Pearl Index	1.4000	0.7598
95% Confidence Interval (Lower limit/Upper limit)	0.4546/3.2670	0.0192/4.2333

Source: Section 15.1, Table 15.2.2.2
N: Number of subjects in specified treatment group and subgroup
n: Number of cycles/subjects with data available
%: Percentage based on N

Method failure Pearl Index

Perfect medication cycles were defined in the SAP as sexual activity cycles without additional contraception where the e-diary documents regular pill intake during the cycle, excluding the cycles with four or more days with forgotten tablets (i.e. no records in the diary on tablet intake), or two or more consecutive days with forgotten tablets (i.e. no records in the diary on tablet intake) during the cycle and no protocol deviations having effect on this cycle. Method failure Pearl Index is based on pregnancies classified as method failure.

Table 34: Perfect Medication Cycles (FAS)

Cycle	Test (N=858) n/m (%)	Reference (N=332) n/m (%)	Total (N=1190) n/m (%)
**Total number of perfect cycles**	4641	1816	6457
Cycle 1	594 / 858 (69.2)	233 / 332 (70.2)	827 / 1190 (69.5)
Cycle 2	606 / 830 (73.0)	246 / 325 (75.7)	852 / 1155 (73.8)
Cycle 3	573 / 816 (70.2)	221 / 304 (72.7)	794 / 1120 (70.9)
Cycle 4	550 / 776 (70.9)	221 / 286 (77.3)	771 / 1062 (72.6)
Cycle 5	523 / 755 (69.3)	206 / 279 (73.8)	729 / 1034 (70.5)
Cycle 6	487 / 734 (66.3)	189 / 269 (70.3)	676 / 1003 (67.4)
Cycle 7	467 / 713 (65.5)	173 / 259 (66.8)	640 / 972 (65.8)
Cycle 8	430 / 701 (61.3)	166 / 251 (66.1)	596 / 952 (62.6)
Cycle 9	411 / 697 (59.0)	161 / 250 (64.4)	572 / 947 (60.4)

Source: Section 15.2, Table 15.2.1.5
N: Number of subjects in specified treatment group
n: Number of subjects with data available
m: Number of subjects in the cycle
%: Percentage based on m

Total number of perfect medication cycles in the Test group was 4641 (Table 34), leading to method failure PI (95% CI) of 1.4006 (0. 4548; 3.2684), see Table 35. In the Reference group, total number of perfect medication cycles was 1816, leading to method failure PI (95% CI) of 0.7159 (0.0181; 3.9885).

Table 35 Method Failure Pearl Index, (FAS)

	Test (N=858)	Reference (N=332)
Pregnancy [n (%)]		
Yes	5 (0.6%)	1 (0.3%)
No	853 (99.4%)	331 (99.7%)
Total number of perfect medication cycles [n]	4641	1816
Method failure Pearl Index	1.4006	0.7159
95% Confidence Interval (Lower limit/Upper limit)	0.4548/3.2684	0.0181/3.9885

Source: Section 15.2, Table 15.2.2.3
N: Number of subjects in specified treatment group
n: Number of cycles/subjects with data available
%: Percentage based on N

The method failure Pearl Index for a subgroup of women aged 35 years or younger is presented in Table 36. In the Test group, total number of perfect medication cycles was 3542, leading to a method failure PI (95% CI) of 1.8351 (0.5959; 4.2826). In the Reference group, total number of perfect medication cycles was 1395, leading to a method failure PI (95% CI) of 0.9319 (0.0236; 5.1922).

Table 36 Method Failure Pearl Index, Age Group ≤ 35 Years (FAS)

	Test (N=682)	Reference (N=259)
Pregnancy [n (%)]		
Yes	5 (0.7%)	1 (0.4%)
No	677 (99.3%)	258 (99.6%)
Total number of perfect medication cycles [n]	3542	1395
Method failure Pearl Index	1.8351	0.9319
95% Confidence Interval (Lower limit/Upper limit)	0.5959/4.2826	0.0236/5.1922

Source: Section 15.2, Table 15.2.2.4
N: Number of subjects in specified group and subgroup
n: Number of cycles/subjects with data available
%: Percentage based on N

Pregnancy ratio

In this trial overall and method failure pregnancy ratios were the same, as all pregnancies were considered method failure (overall pregnancy ratio is based on both user and method failure pregnancies, whereas method failure pregnancy ratio is based on method failure pregnancies).

Table 37: Overall Pregnancy Ratio (FAS)

Cycle	Test (N=858)			Reference (N=332)		
	Pregnancy Ratio m/n (%)	Cumulative Pregnancy Ratio m/n (%)	95% CI (%)	Pregnancy Ratio m/n (%)	Cumulative Pregnancy Ratio m/n (%)	95% CI (%)
Cycle 1	0/835 (0.00)	0.00	-	0/326 (0.00)	0.00	-
Cycle 2	0/817 (0.00)	0.00	-	0/313 (0.00)	0.00	-
Cycle 3	1/785 (0.13)	0.13	0.00 - 0.38	1/291 (0.34)	0.34	0.00 - 1.01
Cycle 4	0/759 (0.00)	0.13	0.00 - 0.38	0/281 (0.00)	0.34	0.00 - 1.01
Cycle 5	1/732 (0.14)	0.26	0.00 - 0.63	0/271 (0.00)	0.34	0.00 - 1.01
Cycle 6	1/714 (0.14)	0.40	0.00 - 0.86	0/262 (0.00)	0.34	0.00 - 1.01
Cycle 7	0/695 (0.00)	0.40	0.00 - 0.86	0/252 (0.00)	0.34	0.00 - 1.01
Cycle 8	1/690 (0.14)	0.55	0.01 - 1.08	0/249 (0.00)	0.34	0.00 - 1.01
Cycle 9	1/664 (0.15)	0.70	0.09 - 1.31	0/242 (0.00)	0.34	0.00 - 1.01

Source: Section 15.2, Table 15.2.2.5
N: Number of subjects in the treatment group
n: Number of subjects with conception date in the respective cycle
m: Number of subjects in the cycle
%: Percentage based on m
CI: Confidence interval

The cumulative 9-cycle pregnancy ratio (95% CI) in the Test group was 0.70% (0.09; 1.31), and was higher than that of 0.34% (0.00; 1.01) in the Reference group, see Table 37.

For the age group ≤ 35 years, the cumulative 9-cycle pregnancy ratio (95% CI) in the Test group was 0.90% (0.11; 1.68) vs. 0.44% (0.00; 1.31) in the Reference group, see Table 38 below.

Table 38: Overall Pregnancy Ratio, Age Group ≤ 35 Years (FAS)

Cycle	Test (N=858)			Reference (N=332)		
	Pregnancy Ratio m/n (%)	Cumulative Pregnancy Ratio m/n (%)	95% CI (%)	Pregnancy Ratio m/n (%)	Cumulative Pregnancy Ratio m/n (%)	95% CI (%)
Cycle 1	0/661 (0.00)	0.00	-	0/255 (0.00)	0.00	-
Cycle 2	0/646 (0.00)	0.00	-	0/241 (0.00)	0.00	-
Cycle 3	1/617 (0.16)	0.16	0.00 - 0.48	1/225 (0.44)	0.44	0.00 - 1.31
Cycle 4	0/592 (0.00)	0.16	0.00 - 0.48	0/217 (0.00)	0.44	0.00 - 1.31
Cycle 5	1/571 (0.18)	0.34	0.00 - 0.80	0/209 (0.00)	0.44	0.00 - 1.31
Cycle 6	1/555 (0.18)	0.51	0.00 - 1.09	0/201 (0.00)	0.44	0.00 - 1.31
Cycle 7	0/540 (0.00)	0.51	0.00 - 1.09	0/195 (0.00)	0.44	0.00 - 1.31
Cycle 8	1/535 (0.19)	0.70	0.01 - 1.39	0/192 (0.00)	0.44	0.00 - 1.31
Cycle 9	1/513 (0.19)	0.90	0.11 - 1.68	0/186 (0.00)	0.44	0.00 - 1.31

Source: Section 15.2, Table 15.2.2.6
N: Number of subjects in the treatment group and subgroup
n: Number of subjects with conception date in the respective cycle
m: Number of subjects in the cycle %: Percentage based on m
CI: Confidence interval

11.8.1.2 Overall Pearl Index for DRSP users in pooled CF111/301 and CF111/302 trials

During the CF111/301 and CF111/302 trials, eight in-treatment pregnancies in subjects treated with DRSP 4.0 mg were reported and all of them were assessed as being method failure pregnancies. The total number of pooled exposure cycles was 14329. The pooled overall PI (95% CI) for subjects exposed to DRSP was 0.7258 (0.3133/1.4301), see Table 39.

Table 39: Overall Pearl Index for Pooled CF111/301 and CF111/302 Trials (FAS)

	Total (N=1571)	Age group ≤ 35 years (N=1251)
Pregnancy [n (%)]		
Yes	8 (0.5%)	8 (0.6%)
No	1563 (99.5%)	1243 (99.4%)
Total number of exposure cycles [n]	14329	11145
Pooled Overall Pearl Index	0.7258	0.9332
95% Confidence Interval (Lower limit/Upper limit)	0.3133/1.4301	0.4029/1.8387

Source: Section 15.2, Tables 15.2.1.7 and 15.2.1.8
N: Number of subjects in specified group
n: Number of cycles/subjects with data available
%: Percentage based on N

All eight pregnancies which occurred during the treatment period with drospirenone were reported for subjects ≤ 35 years. The total number of exposure cycles analysed was 11145, the pooled overall PI (95% CI) in this age subgroup was 0.9332 (0.4029; 1.8387).

<u>Overall Pearl Index after correction for additional contraception and sexual activity</u>

As shown in Table 40, 13168 sexual activity cycles without backup contraception were analysed. The overall PI (95% CI) after correction for additonal contraception and sexual activity status was 0.7898 (0.3410; 1.5562). The corresponding pooled overall PI (95% CI)

after correction for additonal contraception and sexual activity status for subjects ≤ 35 years exposed to drospirenone was 1.0223 (0.4414; 2.0144), based on 10173 cycles.

Table 40: Overall Pearl Index after Correction and Sexual Activity Status in Pooled CF111/301 and CF111/302 Trials (FAS)

	Total (N=1571)	Age group ≤ 35 years (N=1251)
Pregnancy [n (%)]		
Yes	8 (0.5%)	8 (0.6%)
No	1563 (99.5%)	1243 (99.4%)
Total number of sexual activity cycles without backup contaception [n]	13168	10173
Pooled Overall Pearl Index	0.7898	1.0223
95% Confidence Interval (Lower limit/Upper limit)	0.3410/1.5562	0.4414/2.0144

Source: Section 15.2, Tables 15.2.2.1 and 15.2.2.2
N: Number of subjects in specified treatment group and subgroup
N: Number of cycles/subjects with data available
%: Percentage based on N

Method failure Pearl Index

Total number of perfect medication cycles in DRSP users was 10742, leading to a method failure PI (95% CI) of 0.9682 (0.4180; 1.9077). For subjects aged 35 years or younger, 8188 perfect DRSP cycles were analysed, leading to the method failure PI of 1.2702 (0.5484; 2.5027), see Table 41 below.

Table 41: Method Failure Pearl Index in Pooled CF111/301 and CF111/302 Trials (FAS)

	Total (N=1571)	Age group ≤ 35 years (N=1251)
Pregnancy [n (%)]		
Yes	8 (0.5%)	8 (0.6%)
No	1563 (99.5%)	1243 (99.4%)
Total number of perfect medication cycles [n]	10742	8188
Method failure Pearl Index	0.9682	1.2702
95% Confidence Interval (Lower limit/Upper limit)	0.4180/1.9077	0.5484/2.5027

Source: Section 15.2, Tables 15.2.2.3 and 15.2.2.4
N: Number of subjects in specified treatment group and subgroup
n: Number of cycles/subjects with data available
%: Percentage based on N

The calculation of method failure PI was based on the perfect medication cycles. In CF111/301 trial, approximately 13% and in CF111/302 trial, approximately 20% of the cycles were excluded from the method failure analysis due to inaccurate completion of the diaries, i.e. missing e-diary entries on the tablet intake. Thus the method failure PI was relatively high as was based on the lower number of cycles.

Pregnancy ratio

Table 42: Overall Pregnancy Ratio for Pooled CF111/301 and CF111/302 Trials (FAS)

Cycle	Total (N=1571)			Age Group ≤ 35 years (N=1251)		
	Pregnancy Ratio m/n (%)	Cumulative Pregnancy Ratio m/n (%)	95% CI (%)	Pregnancy Ratio m/n (%)	Cumulative Pregnancy Ratio m/n (%)	95% CI (%)
Cycle 1	0/1539 (0.00)	0.00	-	0/1222 (0.00)	0.00	-
Cycle 2	1/1494 (0.07)	0.07	0.00 - 0.20	1/1181 (0.08)	0.08	0.00 - 0.25
Cycle 3	2/1444 (0.14)	0.20	0.00 - 0.44	2/1135 (0.18)	0.26	0.00 - 0. 55
Cycle 4	0/1393 (0.00)	0.20	0.00 - 0.44	0/1088 (0.00)	0.26	0.00 - 0. 55
Cycle 5	1/1354 (0.07)	0.28	0.01 - 0.55	1/1056 (0.09)	0.35	0.01 - 0.70
Cycle 6	1/1304 (0.08)	0.35	0.04 - 0.66	1/1010 (0.10)	0.45	0.06 - 0.85
Cycle 7	0/1264 (0.00)	0.35	0.04 - 0.66	0/976 (0.00)	0.45	0.06 - 0.85
Cycle 8	1/1248 (0.08)	0.43	0.09 - 0.78	1/961 (0.10)	0.56	0.11 - 1.00
Cycle 9	1/1208 (0.08)	0.52	0.13 - 0.90	1/927 (0.11)	0.66	0.17 - 1.16
Cycle 10	0/536 (0.00)	0.52	0.13 - 0.90	0/409 (0.00)	0.66	0.17 - 1.16
Cycle 11	0/527 (0.00)	0.52	0.13 - 0.90	0/402 (0.00)	0.66	0.17 - 1.16
Cycle 12	0/519 (0.00)	0.52	0.13 - 0.90	0/395 (0.00)	0.66	0.17 - 1.16
Cycle 13	1/499 (0.20)	0.72	0.17 - 1.27	1/383 (0.26)	0.93	0.21 - 1.64

Source: Section 15.2, Tables 15.2.2.5 and 15.2.2.6
N: Number of subjects in the treatment group and subgroup
n: Number of subjects with conception date in the respective cycle
m: Number of subjects in the cycle %: Percentage based on m
CI: Confidence interval

The cumulative 13-cycle pregnancy ratio (95% CI) of DRSP users (FAS) in both trials was 0.72 (0.17; 1.27), and that for the age subgroup ≤ 35 years was 0.93 (0.21; 1.64), see Table 42.

11.8.2 Statistical/analytical issues

Full details of the statistical analyses are presented in the SAP, which is provided in Appendix 16.1.9.

11.8.2.1 Adjustment for covariates

Adjustments for covariates were not performed in the analyses of this trial.

11.8.2.2 Handling of drop-outs or missing data

Handling of drop-outs or missing data is described in SAP, Section 5 and references therein (see Appendix 16.1.9).

11.8.2.3 Interim analyses and data monitoring

No interim analyses were performed during the course of this trial.

11.8.2.4 Multicentre studies

This was a multicentre trial.

11.8.2.5 Multiple comparisons/multiplicity

There were no multiple comparisons or multiplicity issues in this trial.

11.8.2.6 Use of an "efficacy subset" of subjects

There were no efficacy subsets of subjects.

11.8.2.7 Active-control studies intended to show equivalence

Not applicable.

11.8.2.8 Examination of subgroups

Based on the assessments at screening visit, in the SAP the following subgroups were defined for exploratory analyses:

- Age groups, defined as ≤ 35 years (682 subjects in the Test and 259 subjects in the Reference group) and >35 years (176 subjects in the Test and 73 subjects in the Reference group)
- BMI groups, defined as BMI < 30 kg/m^2 (828 subjects in the Test and 316 subjects in the Reference group) and BMI ≥ 30 kg/m^2 (30 subjects in the Test and 16 subjects in the reference group)
- Blood pressure groups, defined as SBP < 130 mmHg and DBP < 85 mmHg (727 subjects in the Test and 290 subjects in the Reference group), and SBP ≥ 130 mmHg or DBP ≥ 85 mmHg (131 subjects in the Test and 42 in the Reference group)

The following data were analysed by subgroups:

- Study status by cycles
- Pearl Indices, including those for pooled CF111/301 and CF111/302 trials for the age subgroup ≤ 35 years.
- The main safety data (adverse events and vital signs) were summarised by all above-mentioned subgroups.

<u>Efficacy calculations for the age subgroup ≤ 35 years</u>

Pearl Indices and pregnancy ratio calculations for this age group are presented in Section 11.8.1.

<u>Main safety data by subgroups</u>

Summary tables of adverse events and vital signs by subgroups are presented in Section 12.2 and Section 12.6, respectively.

11.8.3 Tabulation of individual response data

Not applicable.

11.8.4 Drug dose, drug concentration and relationships to response

Relationship of individual drug dose or concentration to response was not analysed in this trial.

11.8.5 Drug-drug and drug-disease interactions

Drug-drug and drug-disease interactions were not analysed in this trial.

11.8.6 By-subject displays

By-subject displays (FAS) of pill intake, sexual activity and use of additional contraceptives are presented in the Blind Data Review Report (Appendix 16.1.9).

11.8.7 Efficacy conclusions

Summary of subject disposition and baseline characteristics

Of the 1365 subjects screened, 152 subjects were screening failures and 1213 subjects were randomised in a ratio 5:2 to treatment with either Test (872 subjects) or Reference (341 subjects) medication. Of 1213 subjects randomised, 1191 received IMP and 22 subjects prematurely terminated the trial before the start of treatment.

Of 1191 treated subjects, 253 (21.2%) subjects terminated prematurely: 170 subjects (19.8%) in the Test and 83 subjects (24.9%) in the Reference group. The most common reasons for discontinuation in both treatment groups were adverse events and withdrawal of consent. In total, 688 (78.9% of the Randomised Set) Test group and 250 (73.3%) Reference group subjects were completers.

The Safety Set and the Full Analysis Set comprised 1190 subjects each: 858 (98.4% of the randomised subjects) in the Test and 332 (97.4%) in the Reference group.

All but three FAS subjects were of Caucasian ethnicity. The mean (SD) subjects' age was 28.9 (7.1) years in each treatment group ranging from 18 to 45 years. The majority of women, 682 subjects (79. 5%) in the Test and 259 (78.0%) in the Reference group, were 35 years of age or younger. In total, 176 (20.5%) Test group and 73 (22.0%) Reference group subjects were older than 35 years. Over 70% of the FAS subjects in each group had completed high school or had a university degree. No statistically significant differences between the groups with regard to subjects' age, ethnicity or highest education level completed were observed.

Current smokers comprised 27.6% of the Test and 31.0% of Reference group subjects.

No statistically significant differences in weight ($p = 0.846$, 2-sample t test), height ($p = 0.439$, 2-sample t test) or BMI ($p = 0.560$, 2-sample t test) were observed between the treatment groups at screening. The proportion of subjects with BMI ≥ 30 kg/m^2 was comparable between the groups (3.5% of the Test and 4.8% of the Reference group subjects). Subjects with systolic blood pressure (SBP) > 140 mmHg, or diastolic blood pressure (DBP) > 90 mmHg were not eligible. At screening, 15.3% of the Test and 12.7% of the Reference group subjects had SBP ≥ 130 mmHg or DBP ≥ 85 mmHg.

887 (74.5%) FAS subjects switched directly from another oral contraceptive, whereas 250 (21.0%) were starters.

The most common previous medical or surgical history finding in both groups was Caesarean section, followed by dysmenorrhoea in the Test group and by vaginal infection in the Reference group. The most frequent ongoing medical history findings were dysmenorrhoea and breast pain.

No major differencies between the treatment groups were observed with regard to the VTE risk factors assessed.

In total 395 women (46.0%) in the Test and 150 women (45.2%) in the Reference group reported having had at least one delivery. Prior miscarriages were reported by 7.9% of the Test and 7.2% of the Reference group subjects, and prior abortions by 15.3% of the Test and 17.5% of the Reference group subjects.

No statistically significant differences were observed between the groups with regard to prior bleeding characteristics. The vast majority of subjects (91.6% of the Test and 91.9% of the Reference group subjects) reported having had scheduled/regular bleeding during the last 6 cycles prior to screening, unscheduled bleeding was uncommon (1.0% of the Test versus 2.1% of the Reference group subjects), absence of more than one bleeding was reported by 0.8% of the Test and 1.8% of the Reference group subjects. Moderate intensity of scheduled bleeding prevailed (68.9% subjects in the Test group versus 70.2% in the Reference subjects).

The incidence of previous spotting was low (2.3% Test group to 4.0% Reference group subjects).

At screening, 232 FAS subjects (19.6% of the Test and 19.3% of the Reference group subjects) reported that they had suffered from dysmenorrhoea within six cycles prior to screening. At follow-up, 150, i.e. more than a half of these subjects, reported having no dysmenorrhoea. In total, 145 FAS subjects (11.2% of the Test and 14.8% of the Reference group) experienced mastodynia/mastalgia within six cycles prior to the screening and 81 subjects of these had no mastodynia/mastalgia at follow up.

In total, 992 (83.4%) of FAS subjects reported at least one prior medication or contraceptive method (range: 82.1% Test to 86.7% Reference group subjects). The most common of these were sex hormones and modulators of the genital system (range: 54.7% Test to 58.7% Reference subjects). Approximately 30% of the subjects in each treatment group reported intake of at least one concomitant medication. The most common were analgesics (7.5% Test to 8.4% Reference group subjects), antibacterials for systemic use (6.9% in each treatment group), and antiinflammatory and antirheumatic products (6.8% of the Test and 6.9% of the Reference group subjects).

Based on tablet count, the mean (SD) overall compliance to the IMP (FAS) was very high: 101.7 (14.13)% in the Test and 101.9 (5.93)% in the Reference group. In total, 163 subjects (13.6%) missed at least one pill. Compliance above 100% was achieved not because the subjects took more tablets than prescribed, but due to unreturned tablets or blisters.

Summary of efficacy results

The primary efficacy variable was the overall Pearl Index. A total of 858 subjects with 6691 drospirenone and 332 subjects with 2487 desogestrel treatment cycles were analysed. During these cycles five Test group and one Reference group subjects became pregnant, all pregnancies occured in the age group ≤35 years and were considered method failure. Secondary efficacy analyses included overal PI after correction for additional contraception and sexual activity status, method failure PI and pregnancy ratio.

The method failure PI was calculated based on sexual activity cycles without additional contraception where the e-diary documented regular pill intake during the cycle, excluding the cycles with four or more days with forgotten tablets (i.e. no records in the diary on tablet intake), or two or more consecutive days with forgotten tablets (i.e. no records in the diary on tablet intake) during the cycle and no protocol deviations having effect on this cycle.

The PI Indices for the FAS and for the age group ≤35 years subjects are presented in the table below:

	**Test**<br>**PI (95% CI)**	**Reference**<br>**PI (95% CI)**
Overall PI	0.9715 (0.3154; 2.2671)	0.5227 (0.0132; 2.9124)
Overall PI for subjects ≤ 35 years	1.2428 (0.4035/2.9004)	0.6767 (0.0171; 3.7705)
Overall PI after correction for additional contraception and sexual activity status	1.0875 (0.3531/2.5379)	0.5845 (0.0148; 3.2568)
Overall PI after correction for additional contraception and sexual activity status for subjects ≤ 35 years	1.4000 (0.4546/3.2670)	0.7598 (0.0192; 4.2333)
Method failure PI	1.4006 (0.4548/3.2684)	0.7159 (0.0181; 3.9885)
Method failure PI for subjects ≤ 35 years	1.8351 (0.5959/4.2826)	0.9319 (0.0236; 5.1922)

The PI point estimate for the Test group was higher than for the Reference group. The PI calculation for the Reference group, however, was less precise, as it was based on a considerably lower number of cycles, resulting in a much wider confidence interval. The upper limit of the PI 95% CI was lower for the Test group than for the Reference group.

The cumulative 9-cycle pregnancy ratio (95% CI) in the Test group was 0.70% (0.09; 1.31), and in the Reference group it was 0.34% (0.00; 1.01). For the age subgroup ≤ 35 years, it was 0.90% (0.11; 1.68) in the Test vs. 0.44% (0; 1.31) in the Reference group.

Pearl Indices for DRSP users in pooled CF111/301 and CF111/302 trials

A total of eight in-treatment pregnancies, assessed as being method failure, were observed in women who used DRSP 0.4 mg up to 13x28 day cycles in CF111/301 and CF111/302 trials. All eight pregnancies were reported for subjects ≤ 35 years. The total number of analysed exposure cycles for the overall PI was 14329. The PI Indices for the FAS and for age group ≤35 years subjects are presented in the table below:

	**Total (N=1571)**<br>**PI (95% CI)**	**Subjects ≤ 35 years (N=1251)**<br>**PI (95% CI)**
Overall PI	0.7258 (0.3133; 1.4301)	0.9332 (0.4029; 1.8387)
Overall PI after correction for additional contraception and sexual activity status	0.7898 (0.3410; 1.5562)	1.0223 (0.4414; 2.0144)
Method failure Pearl Index	0.9682 (0.4180; 1.9077)	1.2702 (0.5484; 2.5027)

The cumulative 13-cycle pregnancy ratio (95% CI) of DRSP users (FAS) in both trials was 0.72 (0.17-1.27), and that of the age subgroup ≤ 35 years it was 0.93 (0.21-1.64).

12. SAFETY EVALUATION

12.1 Extent of Exposure

In total 1190 subjects received randomised trial medication: 858 of them received drospirenone 4.0 mg and 332 received desogestrel 0.075 mg. Exposure to IMP (days) in the SS and the FAS is provided in Table 43. Individual data on exposure to IMP are provided in Appendix 16.2, Listing 16.2.1.6.

The mean (SD) treatment duration was 222.7 (65.79) days in the Test group and 213.9 (72.14) days in the Reference group. The median duration was 252.0 days in both groups, ranging from three to 276 days in the Test and from one to 280 days in the Reference group.

In total 673 (78.4%) Test group and 244 (73.5%) Reference group subjects were exposed to IMP for 252 days or longer.

Table 43: Exposure to Investigational Medicinal Product [Days] (SS, FAS)

		Test (N=858)	Reference (N=332)	Total (N=1190)
Exposure (days) [1]	n	858	332	1190
	Mean (SD)	222.7 (65.79)	213.9 (72.14)	220.3 (67.70)
	Median	252.0	252.0	252.0
	Q1/ Q3	252.0/252.0	242.5/252.0	252.0/252.0
	Min/ Max	3/276	1/280	1/280
Cumulative exposure [n (%)][1]	any	858 (100)	332 (100)	1190 (100)
	≥ 28 days	835 (97.3)	327 (98.5)	1162 (97.6)
	≥ 84 days	787 (91.7)	292 (88.0)	1079 (90.7)
	≥ 168 days	718 (83.7)	263 (79.2)	981 (82.4)
	≥ 252 days	673 (78.4)	244 (73.5)	917 (77.1)
	Missing	-	-	-

Source: Section 15.1, Table 15.1.2.15
N: Number of subjects in specified treatment group
n: Number of subjects with data available
%: Percentage based on N
SD: Standard deviation
[1] Duration was defined as (the date of last IMP intake) – (the date of first IMP intake) + 1.

12.2 Adverse Events (AEs)

Treatment-emergent adverse events (TEAEs) were defined as AEs which started at or after the first administration of the IMP and included those events started prior to the first administration of the IMP but which worsened after the first intake. AEs starting after the last administration of the IMP but within the follow-up period (14 days) were also regarded as treatment-emergent. TEAEs leading to trial termination were obtained from the AE form, where the field "Action taken on study drug" was indicated as "drug withdrawn".

12.2.1 Brief summary of adverse events

Listings of AEs are provided in Appendix 16.2, Listing 16.2.2.1 (original terms) and Listing 16.2.2.2 (MedDRA coding).

A summary of subjects with AEs, including numbers of events, is presented in Table 44.

Table 44: Summary of Subjects with Treatment Emergent Adverse Events (SS)

	Test (N=858) n (%)	Reference (N=332) n (%)	Total (N=1190) n (%)	Fisher's exact test p value
Subjects with at least one AE [n (%)]	357 (41.6)	158 (47.6)	515 (43.3)	0.068
Number of AEs (#)	779	319	1098	
Subjects with at least one TEAE [n (%)]	332 (38.7)	150 (45.2)	482 (40.5)	0.042
Number of TEAEs (#)	705	290	995	
Subjects with at least one related TEAE [n (%)]	135 (15.7)	62 (18.7)	197 (16.6)	0.224
Number of related TEAEs (#) [a]	242	103	345	
Subjects with at least one severe TEAE [n (%)]	24 (2.8)	11 (3.3)	35 (2.9)	0.702
Number of severe TEAEs (#)	28	11	39	
Subjects with at least one SAE [n (%)]	20 (2.3)	7 (2.1)	27 (2.3)	1.000
Number of SAEs (#)	21	9	30	
Subjects with at least one TESAE [n (%)]	15 (1.7)	6 (1.8)	21 (1.8)	1.000
Number of TESAEs (#)	16	8	24	
Subjects with at least one related TESAE [n (%)]	1 (0.1)	1 (0.3)	2 (0.2)	0.480
Number of related TESAEs (#)	1	1	2	
Subjects with at least one TEAE leading to discontinuation [n (%)]	82 (9.6)	44 (13.3)	126 (10.6)	0.074
Number of TEAEs leading to discontinuation (#)	82	44	126	
Subjects who died [n (%)]	0	0	0	

Source: Section 15.3, Table 15.3.1.1.1

N: Number of subjects in specified treatment group n: Number of subjects with data available

#: Number of events %: Percentage based on N

Note: [a] Related TEAEs include TEAEs with the assessments "possibly related" or "related" and events with missing relationship

In total, 332 (38.7%) Test group and 150 (45.2%) Reference group subjects experienced TEAEs. The proportion of subjects with TEAEs was lower in the Test than in the Reference group, and the difference between the groups was statistically significant (p = 0.042, Fisher's exact test).

The proportions of subjects with related TEAEs were comparable between the treatment groups (15.7% of the Test and 18.7% of the Reference group subjects).

The vast majority of TEAEs were assessed as mild or moderate. Severe TEAEs were reported for 24 (2.8%) Test group and 11 (3.3%) Reference group subjects.

No deaths were reported. In total, 21 subjects (1.8%) experienced 24 serious TEAEs with comparable proportions of subjects in the treatment groups (1.7% Test and 1.8% Reference). In total, one subject per each treatment group experienced an SAE, assessed as possibly related to study treatment.

The frequency of TEAEs leading to withdrawal was lower in the Test group (9.6%) than in the Reference group (13.3%), but the difference was not statistically significant (p = 0.074, Fisher's exact test).

12.2.1.1 Summary of adverse events by subgroups

Subjects with TEAEs were analysed by age, BMI and BP subgroups.

Age subgroups

The summary of subjects who experienced TEAEs by age groups is provided in Table 45. For the ≤ 35 years subgroup, both treatment groups were comparable with regard to the frequency of TEAEs in each category. For the subgroup of > 35 years, the frequency of TEAEs, related

TEAEs and TEAEs leading to discontinuation was lower in the Test than in the Reference group.

Table 45: Summary of Subjects with TEAEs by Age Group (SS)

Subjects with:	≤ 35 years<br>Test<br>(N = 682)<br>n (%)	 Reference<br>(N = 259)<br>n (%)	> 35 years<br>Test<br>(N = 176)<br>n (%)	 Reference<br>(N = 73)<br>n (%)
Any TEAE	277 (40.6)	119 (45.9)	55 (31.3)	31 (42.5)
Any related TEAE	116 (17.0)	49 (18.9)	19 (10.8)	13 (17.8)
Any severe TEAE	21 (3.1)	8 (3.1)	3 (1.7)	3 (4.1)
Any serious TEAE	11 (1.6)	5 (1.9)	4 (2.3)	1 (1.4)
Any TEAE leading to discontinuation	69 (10.1)	33 (12.7)	13 (7.4)	11 (15.1)

Source: Section 15.3, Table 15.3.1.1.2
N: Number of subjects in specified treatment group and subgroup
n: Number of subjects with AEs
%: Percentage based on N

BMI subgroups

Table 46: Summary of Subjects with TEAEs by BMI Group (SS)

Subjects with:	< 30 kg/m^2<br>Test<br>(N = 828)<br>n (%)	 Reference<br>(N = 316)<br>n (%)	≥30 kg/m^2<br>Test<br>(N = 30)<br>n (%)	 Reference<br>(N = 16)<br>n (%)
Any TEAE	320 (38.6)	140 (44.3)	12 (40.0)	10 (62.5)
Any related TEAE	128 (15.5)	61 (19.3)	7 (23.3)	1 (6.3)
Any severe TEAE	22 (2.7)	11 (3.5)	2 (6.7)	0
Any serious TEAE	15 (1.8)	6 (1.9)	0	0
Any TEAE leading to discontinuation	79 (9.5)	43 (13.6)	3 (10.0)	1 (6.3)

Source: Section 15.3, Table 15.3.1.1.3
N: Number of subjects in specified treatment group and subgroup
n: Number of subjects with AEs
%: Percentage based on N

The summary of subjects who experienced TEAEs by BMI groups is provided in Table 46. The number of subjects in the high-BMI groups was too small to draw any conclusions from this analysis.

Blood pressure subgroups

The summary of subjects who experienced TEAEs by blood pressure groups is provided in Table 47. No major differences in frequency of any TEAE category were observed between the treatment groups of the SBP<130 mmHg and DBP<85 mmHg subgroup. The number of subjects in the Reference group of the high-BP subgroup was too small to draw any conclusions from this analysis.

Table 47: Summary of Subjects with TEAEs by Blood Pressure Group (SS)

	SBP<130 mmHg and DBP<85 mmHg		SBP≥130 mmHg or DBP ≥ 85 mmHg	
**Subjects with:**	**Test (N = 727) n (%)**	**Reference (N = 290) n (%)**	**Test (N = 131) n (%)**	**Reference (N = 42) n (%)**
Any TEAE	290 (39.9)	130 (44.8)	42 (32.1)	20 (47.6)
Any related TEAE	116 (16.0)	52 (17.9)	19 (14.5)	10 (23.8)
Any severe TEAE	19 (2.6)	9 (3.1)	5 (3.8)	2 (4.8)
Any serious TEAE	13 (1.8)	6 (2.1)	2 (1.5)	0
Any TEAE leading to discontinuation	71 (9.8)	37 (12.8)	11 (8.4)	7 (16.7)

Source: Section 15.3, Table 15.3.1.1.4
N: Number of subjects in specified treatment group and subgroup
n: Number of subjects with AEs
%: Percentage based on N

12.2.2 Display of adverse events

The incidence of the most frequent TEAEs by primary System Organ Class (SOC), reported for at least 2% of subjects in any treatment group is presented in Table 48. The most common primary SOCs were infections and infestations (197 subjects, 16.6%) and reproductive system and breast disorders (143 subjects, 12.0%). The proportions of subjects with AEs classified to the above listed SOCs, were comparable between the groups.

Table 48: Incidence of TEAEs by Primary System Organ Class, Frequency of Subjects with TEAEs ≥ 2.0% in any Treatment Group (SS)

System Organ Class	Test (N=858) n (%)	Reference (N=332) n (%)	Total (N=1190) n (%)
Subjects with at least one TEAE	332 (38.7)	150 (45.2)	482 (40.5)
Infections and infestations	136 (15.9)	61 (18.4)	197 (16.6)
Reproductive system and breast disorders	98 (11.4)	45 (13.6)	143 (12.0)
Skin and subcutaneous tissue disorders	43 (5.0)	26 (7.8)	69 (5.8)
Nervous system disorders	44 (5.1)	22 (6.6)	66 (5.5)
Gastrointestinal disorders	48 (5.6)	13 (3.9)	61 (5.1)
Investigations	46 (5.4)	14 (4.2)	60 (5.0)
Psychiatric disorders	25 (2.9)	11 (3.3)	36 (3.0)
General disorders and administration site conditions	19 (2.2)	4 (1.2)	23 (1.9)

Source: Section 15.3, Table 15.3.1.2
N: Number of subjects in specified treatment group
n: Number of subjects with AEs
%: Percentage based on N

The incidence of the most frequent TEAEs reported for ≥ 2% of subjects in any treatment group by preferred term is presented in Table 49.

The most common individual TEAEs in both treatment groups were vaginal haemorrhage (3.7% of the Test and 7.2% of the Reference group subjects), headache (4.4% of the Test and 5.1% of the Reference group subjects), acne (3.1% Test and 5.7% Reference) and nasopharyngitis (3.4% Test and 3.9% Reference). The treatment groups were comparable with regard to the incidence of the most frequent TEAEs.

Table 49: Incidence of TEAEs by Preferred Term, Frequency of Subjects with TEAEs ≥ 2.0% in any Treatment Group (SS)

Preferred Term	Test (N=858) n(%)	Test (N=858) n*	Reference (N=332) n (%)	Reference (N=332) n*	Total (N=1190) n (%)	Total (N=1190) n*
Vaginal haemorrhage	32 (3.7)	39	24 (7.2)	26	56 (4.7)	65
Headache	38 (4.4)	75	17 (5.1)	37	55 (4.6)	112
Acne	27 (3.1)	32	19 (5.7)	20	46 (3.9)	52
Nasopharyngitis	29 (3.4)	32	13 (3.9)	15	42 (3.5)	47
Cervical dysplasia	26 (3.0)	26	11 (3.3)	11	37 (3.1)	37
Weight increased	21 (2.4)	21	6 (1.8)	6	27 (2.3)	27
Influenza	6 (0.7)	6	7 (2.1)	8	13 (1.1)	14

Source: Section 15.3, Table 15.3.1.2 and Table 15.3.1.3
N: Number of subjects in specified treatment group and subgroup
n: Number of subjects with AEs
%: Percentage based on N

The incidence of TEAEs by age, BMI and blood pressure subgroups is provided in Section 15.3, Table 15.3.1.10, Table 15.3.1.11 and Table 15.3.1.12, respectively.

In Table 50, Table 51 and Table 52 below the incidence of TEAEs which were most common in the Safety Set and listed in Table 49, by age, BMI and blood pressure groups is presented.

Due to the small numbers of subjects in the Reference groups of the high-age, high-BMI and high-BP groups, it was not possible to draw any conclusions with regard to between-group differences in TEAE rates.

Table 50: Incidence of Most Frequent TEAEs by Age Subgroup (SS)

	≤ 35 years		> 35 years	
	Test (N=682) n (%)	Reference (N=259) n (%)	Test (N=176) n (%)	Reference (N=73) n (%)
Vaginal haemorrhage	26 (3.8)	18 (6.9)	6 (3.4)	6 (8.2)
Headache	32 (4.7)	11 (4.2)	6 (3.4)	6 (8.2)
Acne	26 (3.8)	15 (5.8)	1 (0.6)	4 (5.5)
Nasopharyngitis	28 (4.1)	11 (4.2)	1 (0.6)	2 (2.7)
Cervical dysplasia	21 (3.1)	9 (3.5)	5 (2.8)	2 (2.7)
Weight increased	17 (2.5)	6 (2.3)	4 (2.3)	0
Influenza	4 (0.6)	5 (1.9)	2 (1.1)	2 (2.7)

Source: Section 15.3, Table 15.3.1.10
N: Number of subjects in specified treatment group and subgroup
n: Number of subjects with AEs
%: Percentage based on N

Table 51: Incidence of Most Frequent TEAEs by BMI Subgroup (SS)

	<30 kg/m^2		≥30 kg/m^2	
	Test (N=828) n (%)	Reference (N=316) n (%)	Test (N=30) n(%)	Reference (N=16) n (%)
Vaginal haemorrhage	30 (3.6)	24 (7.6)	2 (6.7)	0
Headache	35 (4.2)	15 (4.7)	3 (10.0)	2 (12.5)
Acne	27 (3.3)	18 (5.7)	0	2 (12.5)
Nasopharyngitis	28 (3.4)	12 (3.8)	1 (3.3)	1 (6.3)
Cervical dysplasia	26 (3.1)	9 (2.8)	0	2 (12.5)
Weight increased	17 (2.1)	6 (1.9)	4 (13.3)	0
Influenza	6 (0.7)	7 (2.2)	0	0

Source: Section 15.3, Table 15.3.1.11
N: Number of subjects in specified treatment group and subgroup
n: Number of subjects with AEs
%: Percentage based on N

Table 52: Incidence of Most Frequent TEAEs by Blood Pressure Subgroup (SS)

	SBP < 130 mmHg and DBP<85 mmHg		SBP >= 130 mmHg or DBP >= 85 mmHg	
	Test (N=727) n (%)	Reference (N=290) n (%)	Test (N=131) n(%)	Reference (N=42) n (%)
Vaginal haemorrhage	29 (4.0)	21 (7.2)	3 (2.3)	3 (7.1)
Headache	32 (4.4)	13 (4.5)	6 (4.6)	4 (9.5)
Acne	24 (3.3)	14 (4.8)	3 (2.3)	5 (11.9)
Nasopharyngitis	27 (3.7)	11 (3.8)	2 (1.5)	2 (4.8)
Cervical dysplasia	25 (3.4)	10 (3.4)	1 (0.8)	1 (2.4)
Weight increased	15 (2.1)	6 (2.1)	6 (4.6)	0
Influenza	4 (0.6)	6 (2.1)	2 (1.5)	1 (2.4)

Source: Section 15.3, Table 15.3.1.12
N: Number of subjects in specified treatment group and subgroup
n: Number of subjects with AEs
%: Percentage based on N

12.2.3 Analysis of adverse events

TEAEs classified as at least possibly related to study treatment were reported for 15.7% of the Test and 18.7% of the Reference group subjects, see Table 53.

The most frequently reported TEAEs assessed as at least possibly related to trial treatment were vaginal haemorrhage (3.1% of Test group and 6.0% of Reference group subjects), acne (3.0% Test and 5.1% Reference) and weight increased (2.2% Test and 1.8% Reference).

Table 53: Incidence of TEAEs Assessed as Related, Which Occurred in > 1 Subject of any Treatment Group (SS)

Preferred Term	Test (N=858) n (%)	Reference (N=332) n (%)	Total (N=1190) n (%)
Subjects with at least one related TEAE	135 (15.7)	62 (18.7)	197 (16.6)
Vaginal haemorrhage	27 (3.1)	20 (6.0)	47 (3.9)
Acne	26 (3.0)	17 (5.1)	43 (3.6)
Weight increased	19 (2.2)	6 (1.8)	25 (2.1)
Headache	13 (1.5)	5 (1.5)	18 (1.5)
Libido decreased	10 (1.2)	5 (1.5)	15 (1.3)
Breast pain	8 (0.9)	5 (1.5)	13 (1.1)
Uterine haemorrhage	5 (0.6)	5 (1.5)	10 (0.8)
Alopecia	6 (0.7)	2 (0.6)	8 (0.7)
Dysmenorrhoea	5 (0.6)	1 (0.3)	6 (0.5)
Abdominal pain	5 (0.6)	0	5 (0.4)
Increased appetite	3 (0.3)	1 (0.3)	4 (0.3)
Metrorrhagia	3 (0.3)	1 (0.3)	4 (0.3)
Mood altered	3 (0.3)	1 (0.3)	4 (0.3)
Mood swings	4 (0.5)	0	4 (0.3)
Ovarian cyst	2 (0.2)	2 (0.6)	4 (0.3)
Fatigue	3 (0.3)	0	3 (0.3)
Hot flush	2 (0.2)	1 (0.3)	3 (0.3)
Vertigo	3 (0.3)	0	3 (0.3)
Abdominal pain upper	2 (0.2)	0	2 (0.2)
Affect lability	2 (0.2)	0	2 (0.2)
Alanine aminotransferase increased	2 (0.2)	0	2 (0.2)
Anxiety	2 (0.2)	0	2 (0.2)
Aspartate aminotransferase increased	2 (0.2)	0	2 (0.2)
Blood thyroid stimulating hormone increased	2 (0.2)	0	2 (0.2)
Depressed mood	2 (0.2)	0	2 (0.2)
Gamma-glutamyltransferase increased	2 (0.2)	0	2 (0.2)
Nausea	2 (0.2)	0	2 (0.2)
Obesity	2 (0.2)	0	2 (0.2)
Oedema Peripheral	2 (0.2)	0	2 (0.2)
Seborrhoea	2 (0.2)	0	2 (0.2)
Rash	2 (0.2)	0	2 (0.2)
Vulvovaginal Dryness	2 (0.2)	0	2 (0.2)

Source: Section 15.3, Table 15.3.1.4
N: Number of subjects in specified treatment group n: Number of subjects with data available
%: Percentage based on N

The vast majority of TEAEs were classified as mild or moderate (Table 54). Overall, 35 subjects (2.9%) reported TEAEs, which were assessed by the investigators as being of severe intensity. The proportions of subjects with severe TEAEs were comparable between the treatment groups.

Table 54: TEAEs by Severity (SS)

Severity category [a]	Test (N=858) n (%)	Reference (N=332) n (%)	Total (N=1190) n (%)
Subjects with at least one TEAE	332 (38.7)	150 (45.2)	482 (40.5)
Mild	230 (26.8)	91 (27.4)	321 (27.0)
Moderate	167 (19.5)	81 (24.4)	248 (20.8)
Severe	24 (2.8)	11 (3.3)	35 (2.9)

Source: Section 15.3, Table 15.3.1.5
N: Number of subjects in specified treatment group
n: Number of subjects with data available
%: Percentage based on N
Note: [a] A subject may have more than one AE in any category.

The frequency of individual TEAEs assessed as severe was low, only four of them: vaginal haemorrhage (two subjects per each treatment group, 0.2% Test group and 0.6% Reference group), acne and libido decreased (two subjects, 0.2% in the Test and one, 0.3% in the Reference group, each) and headache was reported for two subjects, 0.2% in the Test group. All other TEAEs each occurred only in one subject (Section 15.3, Table 15.3.1.5).

12.2.4 Listing of adverse events by subject

Full details of all AEs are presented in Appendix 16.2, Listing 16.2.2.1 (general) and Listing 16.2.2.2 (MedDRA coding).

12.3 **Deaths, Other Serious Adverse Events and Other Significant Adverse Events**

The CRFs for all subjects with SAEs, AEs leading to withdrawal and pregnancies are provided in Appendix 16.3.1.

In this trial there were no deaths reported.

Other serious adverse events

Subjects with SAEs are listed in Appendix 16.2, Listing 16.2.2.4. Narratives for subjects with SAEs are provided in Section 15.3.3.

Overall, 21 subjects (1.8%) experienced treatment emergent SAEs, see Table 55. Two of the reported TESAEs, hepatic adenoma (Test group Subject #351032) and ectopic pregnancy (Reference group Subject #460008) were assessed as possibly related to study treatment and were reported as SUSARs. After the database lock, some doubts have arisen regarding the diagnosis of hepatic adenoma in favour of focal nodular hyperplasia. The diagnosis will be clarified in July 2014, when the results of MRI and ultrasound examination are available.

All other TESAEs were assessed as not or unlikely to be related to IMP.

The majority of the reported TESAEs were of moderate intensity. According to the investigators‘ judgment, the following TESAEs were of severe intensity:

- Test group: hepatic adenoma (Subject #351032), tension headache (Subject #362001), nephrolithiasis (Subject #371009), appendicitis (#759001) and cervical dysplasia (Subject #859012)
- Reference group: cervix neoplasm (Subject#252010) and ectopic pregnancy (Subject #460008).

Reference group Subject #460008 who experienced an TESAE of ectopic pregnancy prematurely discontinued the trial. The primary reason for discontinuation in the CRF was documented as “pregnancy” (following the requirement of the protocol), therefore this subject is not present in the Listing 16.2.2.5, TEAEs leading to premature discontinuation,

AE summary Tables 15.3.1.1.1, 15.3.1.1.2, 15.3.1.1.3, 15.3.1.1.4 and Table 15.3.1.8, as well as in the following in-text tables: Table 44, Table 45, Table 46, Table 47 and Table 56.

The majority of the TESAEs resolved, except two fibroadenoma of breast events (Subjects #759001 and #759002), hepatic adenoma (Subject #35103) and cervical dysplasia (Subject #859012) in the Test group, and cervix neoplasm (Subject#252010) in the Reference group with documented "not resolved" or "unknown" outcome.

Table 55: Incidence of Serious Adverse Events (SS)

Preferred Term	Test (N=858) n (%)	Reference (N=332) n (%)	Total (N=1190) n (%)
Subjects with at least one SAE	20 (2.3)	7 (2.1)	27 (2.3)
Subjects with at least one TESAE	15 (1.7)	6 (1.8)	21 (1.8)
Appendicitis	3 (0.3)	0	3 (0.3)
Selective abortion*	3 (0.3)	0	3 (0.3)
Cervical dysplasia	2 (0.2)	0	2 (0.2)
Concussion	1 (0.1)	1 (0.3)	2 (0.2)
Fibroadenoma of breast	2 (0.2)	0	2 (0.2)
Abortion spontaneous*	1 (0.1)	0	1 (0.1)
Blood potassium increased	1 (0.1)	0	1 (0.1)
Cervix neoplasm	0	1 (0.3)	1 (0.1)
Colitis	0	1 (0.3)	1 (0.1)
Ectopic Pregnancy	0	1 (0.3)	1 (0.1)
Hepatic adenoma	1 (0.1)	0	1 (0.1)
Hyperkalaemia	1 (0.1)	0	1 (0.1)
Hypersensitivity	1 (0.1)	0	1 (0.1)
Premature baby*	1 (0.1)	0	1 (0.1)
Nasal Polyps	0	1 (0.3)	1 (0.1)
Nephrolithiasis	1 (0.1)	0	1 (0.1)
Neurological infection	0	1 (0.3)	1 (0.1)
Orthostatic hypotension	0	1 (0.3)	1 (0.1)
Procedural pain	1 (0.1)	0	1 (0.1)
Tension headache	1 (0.1)	0	1 (0.1)
Thyroid cancer*	-	1 (0.3)	1 (0.1)
Vertigo	0	1 (0.3)	1 (0.1)
Wrist fracture	1 (0.1)	0	1 (0.1)

Source: Section 15.3, Table 15.3.1.6 and Table 15.3.1.7
N: Number of subjects in specified treatment group
n: Number of subjects with data available
%: Percentage based on N
*Non-treatment emergent SAEs

Additionally, Subject #353001 who withdrew prior to randomisation experienced an SAE of pyelonephritis. An SAE of ankle fracture was reported for Subject #463002, allocated to the Reference group. The subject did not start the intake of IMP and withdrew consent.

The following SAEs with a start date of more than 14 days after the last IMP intake and thus not considered to be treatment-emergent, were reported in the Test group:

- Three cases of selective abortion (reported term: elective abortion) for Subjects: #256038, #365018 and #751010. The events were erroneously coded as "selective abortions", but they should be considered as "induced abortions".
- Spontaneous abortion for Subject #256012, and
- Premature baby for Subject #360029.

In the Reference group a case of thyroid cancer was reported for Subject #256022.

AEs of special interest

In this trial, two TEAEs of special interest, hyperkalaemia (Subject #252013) of moderate and blood potassium increased (Subject #454004) of mild intensity were reported in the Test group. The cases were judged by the investigators as unlikely to be related to study treatment. Based on the requirements defined in the protocol, both events were documented as SAEs.

Subject #252013 developed an elevated potassium level of 5.7 mmol/L (reference range: 3.5-5.3 mmol/L) after completion of the study treatment (Day 256).

Subject #454004 had received study medication for 164 days prior to the onset of the event (potassium level of 5.9 mmol/L). Both subjects did not use any concomitant medication that could cause elevations of potassium levels. The subjects did not present any clinical signs of hyperkalaemia, the ECGs did not reveal pathological signs. IMP was not stopped for Subject #454004, as the repeated test did not reveal an increase of potassium and due to the normal results of other important parameters defined in the protocol (blood urea nitrogen, creatinine, creatine kinase). The events resolved without additional treatment within one week (Subject #252013) and three weeks (Subject #454004), respectively.

Subjects with AEs of special interest are listed in Appendix 16.2, Listing 16.2.2.6; incidence of TEAEs of special interest can be found in Section 15.3, Table 15.3.1.9.

Adverse events leading to withdrawal from the trial

The subjects withdrawn from the trial due to TEAEs are listed in Appendix 16.2, Listing 16.2.2.5. Narratives for subjects withdrawn from the trial due to TEAEs are provided in Section 15.3.3.

Overall 82 (9.6%) Test group and 44 (13.3%) Reference group subjects experienced TEAEs, leading to premature termination of the trial (Table 56). The most frequent SOCs of TEAEs leading to withdrawal were reproductive system and breast disorders (51 subjects, 4.3%), skin and subcutaneous tissue disorders (26 subjects, 2.2%) as well as investigations and psychiatric disorders (15 subjects, 1.3% each), see Section 15.3, Table 15.3.1.8.

The most common individual TEAEs leading to withdrawal were vaginal haemorrhage (2.6% of the Test group vs. 5.4% of the Reference group subjects) and acne (1.0% Test group vs. 2.7% Reference group).

Table 56: Incidence of TEAEs Leading to Premature Discontinuation (SS)

Preferred Term	Test (N=858) n (%)	Reference (N=332) n (%)	Total (N=1190) n (%)
Subjects with at least one TEAE leading to withdrawal	82 (9.6)	44 (13.3)	126 (10.6)
Vaginal haemorrhage	22 (2.6)	18 (5.4)	40 (3.4)
Acne	9 (1.0)	9 (2.7)	18 (1.5)
Weight increased	8 (0.9)	3 (0.9)	11 (0.9)
Uterine haemorrhage	5 (0.6)	3 (0.9)	8 (0.7)
Libido decreased	5 (0.6)	2 (0.6)	7 (0.6)
Headache	2 (0.2)	2 (0.6)	4 (0.3)
Alopecia	2 (0.2)	1 (0.3)	3 (0.3)
Mood swings	3 (0.3)	0	3 (0.3)
Abdominal pain	2 (0.2)	0	2 (0.2)
Depressed mood	2 (0.2)	0	2 (0.2)
Depression	1 (0.1)	1 (0.3)	2 (0.2)
Gamma-glutamyltransferase increased	2 (0.2)	0	2 (0.2)
Nausea	2 (0.2)	0	2 (0.2)
Rash	2 (0.2)	0	2 (0.2)
Abdominal pain lower	1 (0.1)	0	1 (0.1)
Abdominal pain upper	1 (0.1)	0	1 (0.1)
Affective disorder	1 (0.1)	0	1 (0.1)
Alanine aminotransferase increased	1 (0.1)	0	1 (0.1)
Blood thyroid stimulating hormone increased	1 (0.1)	0	1 (0.1)
Constipation	1 (0.1)	0	1 (0.1)
Contact lens intolerance	1 (0.1)	0	1 (0.1)
Dysmenorrhoea	1 (0.1)	0	1 (0.1)
Feeling abnormal	0	1 (0.3)	1 (0.1)
Generalised oedema	0	1 (0.3)	1 (0.1)
Hot flush	1 (0.1)	0	1 (0.1)
Hyperhidrosis	1 (0.1)	0	1 (0.1)
Hyperthyroidism	1 (0.1)	0	1 (0.1)
Hypertrichosis	0	1 (0.3)	1 (0.1)
Malaise	1 (0.1)	0	1 (0.1)
Metrorrhagia	0	1 (0.3)	1 (0.1)
Premenstrual syndrome	1 (0.1)	0	1 (0.1)
Respiratory tract infection	0	1 (0.3)	1 (0.1)
Skin disorder	1 (0.1)	0	1 (0.1)
Vertigo	1 (0.1)	0	1 (0.1)

Source: Section 15.3, Table 15.3.1.8
N: Number of subjects in specified treatment group
n: Number of subjects with data available
%: Percentage based on N

12.4 Pregnancies

Overall, 12 pregnancies (six in-treatment and six post-treatment), occurring after the start of IMP intake were reported in this trial, see Table 57. All pregnancies occurred in the Test group, except one extrauterine pregnancy in the Reference group (Subject #46008). Narratives of subjects who became pregnant after the start of IMP intake are provided in Section 15.3.3. The CRFs for these subjects are provided in Appendix 16.3.1.

Table 57: Pregnancies (SS)

Subject No	Treatment group	Age (years)	Outcome	Comments
256004	Test	25	Healthy male twins via C-section	On-treatment pregnancy The subject used amoxicillin for 7 days.
256026	Test	20	Normal male baby	On-treatment pregnancy
360029	Test	23	Premature male baby	On-treatment pregnancy, IMP compliance issues reported. SAE: premature baby
365018	Test	22	Elective abortion	On-treatment pregnancy, IMP compliance issues reported. SAE: selective abortion.
751010	Test	26	Elective abortion	On-treatment pregnancy, IMP compliance issues reported. SAE: selective abortion.
46008	Reference	21	Salpingectomy due to ectopic pregnancy	On-treatment pregnancy, IMP compliance issues reported. SAE: ectopic pregnancy.
252019	Test	26	Expected delivery: 21-JUL-2014	Post-study pregnancy
253001	Test	33	Normal baby	Post-study pregnancy. Lost contact with the subject. According to unofficial information, healthy baby was born on 28-MAR-2014 (gender and other details are unknown).
256012	Test	27	Spontaneous abortion	Post-study pregnancy. SAE: abortion spontaneous.
256038	Test	21	Elective abortion	Post-study pregnancy. SAE: selective abortion.
370015	Test	20	Normal male baby	Post-study pregnancy
859003	Test	19	Expected delivery: 24-AUG-2014	Post-study pregnancy

Source: Section 15.2, Table 15.2.1.6 and Section 15.3.3.3, Pregnancy CIOMS

12.5 Clinical Laboratory Evaluation

The reference ranges of laboratory parameters as well as methods used for the tests are provided in Appendix 16.1.10.

12.5.1 Listing of individual laboratory measurements by subject and each abnormal laboratory value

By-subject listings of laboratory assessments can be found in Appendix 16.2, Listing 16.2.3.1 (haematology), Listing 16.2.3.2 (biochemistry), Listing 16.2.3.3 (haemostatic parameters), Listing 16.2.3.4 (carbohydrate metabolism parameters), Listing 16.2.3.5 (bone metabolism parameters) and Listing 16.2.3.6 (urinalysis).

12.5.2 Evaluation of each laboratory parameter

For all laboratory tests baseline was defined as the last non-missing value collected before the first IMP intake, and endpoint was defined as the last non-missing value.

Haematology and biochemistry laboratory assessments were performed for all subjects at screening, visits V3, V4 (electrolytes only) and V5 (or EDV). Haemostatic, carbohydrate metabolism and bone metabolism parameters were analysed in a subset of 39 Test group and

29 Reference group subjects at visits V1b and V5 (or EDV). Urinalysis by dipstick was performed for all subjects at screening, V3 and V5 (or EDV).

12.5.2.1 Laboratory values over time and individual subject changes

Haematology

The mean and median values of haematology parameters were within reference ranges at baseline and at endpoint and the changes over time were not clinically significant (Section 15.3, Table 15.3.2.12). Though statistically significant differences between the groups were observed for mean values of erythrocytes, haemoglobin and haematocrit, the differences between the groups were small, see Table 58.

Table 58: Summary of Haematology Variables with Statistically Significant Differences Between the Treatment Groups (SS)

Parameter	Visit		Test (N=858)	Reference (N=332)	2-sample t test p-value
**Erythrocytes (10^{12}/L)**	Baseline	n	858	332	
[Ref. range: 3.6 - 5]		Mean (SD)	4.446 (0.3107)	4.456 (0.3308)	0.6239
		Median	4.430	4.440	
		Min/ Max	3.54/6.29	3.38/6.16	
	Endpoint	n	838	320	
		Mean (SD)	4.421 (0.3177)	4.497 (0.3382)	0.0004
		Median	4.410	4.470	
		Min/ Max	3.57/5.97	3.55/5.84	
	Change	Mean (SD)	-0.022 (0.2810)	0.046 (0.2773)	0.0002
		Median	-0.020	0.065	
		Min/ Max	-2.01/1.28	-0.86/1.44	
**Haematocrit (%)**	Baseline	n	858	332	
[Ref. range: 0.37–0.47%]		Mean (SD)	0.396 (0.0289)	0.395 (0.0266)	0.9008
		Median	0.395	0.390	
		Min/ Max	0.30/0.52	0.31/0.48	
	Endpoint	n	838	320	
		Mean (SD)	0.405 (0.0303)	0.410 (0.0312)	0.0137
		Median	0.400	0.410	
		Min/ Max	0.32/0.53	0.31/0.49	
	Change	Mean (SD)	0.010 (0.0298)	0.015 (0.0303)	0.0043
		Median	0.010	0.020	
		Min/ Max	-0.09/0.14	-0.12/0.12	
**Haemoglobin (mmol/L)**	Baseline	n	858	332	
[Ref. range: 7.5 – 9.9]		Mean (SD)	8.14 (0.612)	8.13 (0.568)	0.8920
		Median	8.10	8.10	
		Min/ Max	5.2/10.2	6.0/9.8	
	Endpoint	n	838	320	
		Mean (SD)	8.11 (0.574)	8.21 (0.596)	0.0068
		Median	8.10	8.30	
		Min/ Max	5.0/10.1	5.8/9.7	
	Change	Mean (SD)	-0.03 (0.555)	0.09 (0.515)	0.0016
		Median	0.00	0.10	
		Min/ Max	-2.4/2.7	-2.2/2.1	

Source: Section 15.3, Table 15.3.2.1
N: Number of subjects in specified treatment group
n: Number of subjects with data available
SD: Standard deviation

The most frequent changes of haematology parameters, reported for more than 5.0% of subjects in any treatment group are provided in Table 59 below. The proportions of subjects were comparable between the treatment groups.

Table 59: Shifts of Haematology Parameters from Baseline to Endpoint in >5.0% of Subjects of Any Group (SS)

Variable	Shift	Test (N=858) n (%)	Reference (N = 332) n (%)
Haematocrit	Normal to Low	47 (5.5)	18 (5.4)
	Low to normal	93 (10.8)	39 (11.7)
Haemoglobin	Normal to Low	43 (5.0)	19 (5.7)
	Low to normal	53 (6.2)	20 (6.0)
MCV	Normal to high	111 (12.9)	44 (13.3)
Neutrophils	Normal to high	33 (3.8)	18 (5.4)
	High to normal	53 (6.2)	15 (4.5)
Neutrophils abs	Normal to high	36 (4.2)	20 (6.0)
	High to normal	54 (6.3)	17 (5.1)
Eosinophils abs.	Low to normal	44 (5.1)	15 (4.5)

Source: Section 15.3, Table 15.3.2.12
N: Number of subjects in specified treatment group
n: Number of subjects with data available
%: Percentage based on N

Numbers of subjects with normal and abnormal haematology values are presented in Section 15.3, Table 15.3.2.6.

Biochemistry and TSH

The changes of biochemistry variables over time were not clinically significant in each group, too. Though statistically significant differences between the groups were observed for mean values of albumin, bilirubin direct and bilirubin total, cholesterol total (all SS subjects and fasting subjects separately) and triglycerides (all SS subjects and fasting subjects), these differences were small (Section 15.3, Table 15.3.2.2).

The mean and median values of fasting cholesterols and triglycerides decreased over time in both treatment groups, see Table 60 below.

Table 60: Summary of Cholesterol and Triglycerides under Fasting Conditions (SS)

Parameter	Visit		Test (N=858)	Reference (N=332)	2-sample t test p-value
**Cholesterol (mmol/L)**	Baseline	n	514	195	
[Ref. range: 4.14 - 6.73]		Mean (SD)	4.752 (0.7936)	4.666 (0.8663)	0.2080
		Median	4.690	4.530	
		Min/ Max	2.59/7.41	2.67/8.00	
	Endpoint	n	522	199	
		Mean (SD)	4.473 (0.7863)	4.289 (0.8050)	0.0052
		Median	4.430	4.250	
		Min/ Max	2.28/7.20	2.31/9.01	
	Change	n	420	162	
		Mean (SD)	-0.247 (0.8108)	-0.373 (0.8018)	0.0906
		Median	-0.290	-0.345	
		Min/ Max	-2.82/3.60	-2.54/2.15	
**Cholesterol-HDL**	Baseline	n	514	195	
**(mmol/L)**		Mean (SD)	1.712 (0.3909)	1.690 (0.4185)	0.5180
[Ref. range: 1.09 – 2.28]		Median	1.680	1.680	
		Min/ Max	0.78/3.26	0.47/3.00	
	Endpoint	n	522	199	
		Mean (SD)	1.578 (0.3443)	1.522 (0.3528)	0.0520
		Median	1.550	1.500	
		Min/ Max	0.75/2.77	0.70/3.26	
	Change	n	420	162	
		Mean (SD)	-0.140 (0.3414)	-0.141 (0.3516)	0.9746
		Median	-0.130	-0.130	
		Min/ Max	-1.29/1.24	-0.99/1.14	
**Cholesterol-LDL**	Baseline	n	514	195	
**(mmol/L)**		Mean (SD)	2.680 (0.7299)	2.588 (0.8104)	0.1462
[Ref. range: 1.97 – 5.65]		Median	2.620	2.490	
		Min/ Max	0.70/5.18	1.01/5.36	
	Endpoint	n	522	199	
		Mean (SD)	2.566 (0.7183)	2.457 (0.7357)	0.0697
		Median	2.540	2.430	
		Min/ Max	0.80/5.18	0.88/6.45	
	Change	n	420	162	
		Mean (SD)	-0.091 (0.7059)	-0.147 (0.7373)	0.3919
		Median	-0.100	-0.050	
		Min/ Max	-2.28/3.11	-2.74/1.97	
**Triglycerides (mmol/L)**	Baseline	n	514	195	
[Ref. range: 0.8 – 1.94]		Mean (SD)	1.064 (0.5281)	1.124 (0.6561)	0.2131
		Median	0.950	0.980	
		Min/ Max	0.30/4.55	0.27/5.94	
		n	522	199	
	Endpoint	Mean (SD)	0.927 (0.4662)	0.906 (0.4821)	0.5910
		Median	0.820	0.790	
		Min/ Max	0.33/4.71	0.32/3.84	
		n	420	162	
	Change	Mean (SD)	-0.111 (0.5629)	-0.226 (0.6520)	0.0351
		Median	-0.095	-0.165	
		Min/ Max	-2.64/3.59	-2.43/2.00	

Source: Section 15.3, Table 15.3.2.2
N: Number of subjects in specified treatment group
n: Number of subjects with data available
SD: Standard deviation

Most frequent changes of biochemistry parameters and TSH, reported for >5.0% of subjects in any treatment group are provided in Table 61. The proportions of subjects were comparable between the treatment groups.

Numbers of subjects with normal and abnormal biochemistry parameters and TSH are presented in Section 15.3, Table 15.3.2.7.

Table 61: Biochemistry Variables with Individual Subject Changes in More than 5.0% of Subjects (SS)

Variable	Shift	Test (N=858) n (%)	Reference (N = 332) n (%)
Bilirubin direct	Normal to high	58 (6.8)	27 (8.1)
Bilirubin total	Normal to high	31 (3.6)	17 (5.1)
Cholesterol total	Normal to low	163 (19.0)	74 (22.3)
	Low to normal	57 (6.6)	19 (5.7)
Cholesterol total (fasting)	Normal to low	90 (10.5)	38 (11.4)
	Low to normal	33 (3.8)	13 (3.9)
Cholesterol-HDL	Normal to low	33 (3.8)	18 (5.4)
	High to normal	46 (5.4)	22 (6.6)
Cholesterol-LDL	Normal to low	84 (9.8)	32 (9.6)
	Low to normal	56 (6.5)	20 (6.0)
Cholesterol-LDL (fasting)	Normal to low	44 (5.1)	17 (5.1)
CK NAC-activated	Normal to high	59 (6.9)	24 (7.2)
	High to normal	46 (5.4)	13 (3.9)
Creatinine	Normal to high	110 (12.8)	39 (11.7)
	High to normal	57 (6.6)	29 (8.7)
LDH	Normal to low	54 (6.3)	18 (5.4)
	Low to normal	157 (18.3)	60 (18.1)
Triglycerides	Normal to low	181 (21.1)	76 (22.9)
	Low to normal	94 (11.0)	32 (9.6)
	High to normal	43 (5.0)	18 (5.4)
Triglycerides (fasting)	Normal to low	93 (10.8)	34 (10.2)
	Low to normal	52 (6.1)	14 (4.2)
TSH	High to normal	34 (4.0)	17 (5.1)

Source: Section 15.3, Table 15.3.2.13
N: Number of subjects in specified treatment group
n: Number of subjects with data available
%: Percentage based on N

Haemostatic parameters

Summary statistics for haemostatic parameters are presented in Section 15.3, Table 15.3.2.3. Statistically significant differences between the treatment groups were identified for the clotting factor VII and protein C activity, see Table 62. No relevant changes were observed in mean and median values for other haemostatic variables over time, and there were no relevant differences between the treatment groups.

Table 62: Summary of Haemostatic Parameters with Statistically Significant Differences Between the Groups (SS)

Parameter	Visit		Test (N=858)	Reference (N=332)	2-sample t test p-value
**Clotting factor VII**	Baseline	n	39	29	
[Ref. range: 0.57 - 1.47%]		Mean (SD)	1.123 (0.2486)	1.241 (0.2607)	0.0613
		Median	1.120	1.330	
		Min/ Max	0.67/1.50	0.79/1.50	
	Endpoint	n	36	28	
		Mean (SD)	1.066 (0.2351)	1.034 (0.1964)	0.5611
		Median	0.985	0.980	
		Min/ Max	0.67/1.50	0.53/1.50	
	Change	n	33	28	
		Mean (SD)	-0.033 (0.2713)	-0.218 (0.2594)	0.0088
		Median	-0.040	-0.255	
		Min/ Max	-0.52/0.50	-0.61/0.21	
**Protein C activity**	Baseline	n	39	29	
[Ref. range: 0.7 - 1.5%]		Mean (SD)	1.140 (0.2052)	1.293 (0.2447)	0.0069
		Median	1.150	1.210	
		Min/ Max	0.73/1.59	0.91/1.78	
	Endpoint	n	36	28	
		Mean (SD)	1.108 (0.1688)	1.136 (0.2230)	0.5779
		Median	1.120	1.135	
		Min/ Max	0.76/1.45	0.81/1.53	
	Change	n	33	28	
		Mean (SD)	-0.033 (0.2302)	-0.161 (0.1989)	0.0249
		Median	-0.070	-0.165	
		Min/ Max	-0.43/0.56	-0.71/0.25	

Source: Section 15.3, Table 15.3.2.3
N: Number of subjects in specified treatment group
n: Number of subjects with data available
SD: Standard deviation

At baseline mean (SD) values of clotting factor VII were lower in the Test (1.123 [0.2486]%) than in the Reference group (1.241 [0.2607]%). At endpoint, the mean values of factor VII were comparable between the groups, but the change from baseline to endpoint was more pronounced in the Reference group leading to the statistically significant difference (p = 0.0088, 2-sample t test) between the groups.

Mean [SD] protein C activity in the Test group at baseline was also lower than in the Reference group (1.140 [0.2052]% vs. 1.293 [0.2447]%; p = 0.0069, 2-sample t test). The same was also true for the endpoint values (1.108 [0.1688]% vs. 1.136 [0.2230]%). The difference in change of mean (SD) protein C activity from baseline to endpoint was seen: -0.033 (0.2302)% in the Test vs. -0.161 (0.1989)% in the Reference group; p = 0.0249, 2-sample t test.

The difference in change of clotting factor VII and Protein C activity during the trial may be attributed to the baseline levels' differences.

Changes from normal to abnormal values of haemostatic variables were not frequent (Table 63). The majority of abnormal values were documented at baseline, at endpoint low or high haemostatic parameters' values were reported for a few subjects only. None of the abnormal values was classified by the investigators as being clinically significant (Section 15.3, Table 15.3.2.8).

Table 63: Changes of Haemostatic Parameters from Baseline to Endpoint (SS)

Variable	Shift	Test (n=33) n (%)	Reference (n = 28) n (%)
Apc resistance	Normal to low	1 (3.0)	0
Antithrombin III	Normal to low	0	1 (3.6)
	Low to normal	3 (9.1)	2 (7.1)
D-dimer	Normal to high	1 (3.0)	1 (3.6)
	High to normal	2 (6.1)	0
Clotting factor VII	Normal to high	2 (6.1)	0
	High to normal	4 (12.1)	10 (35.7)
Clotting factor VIII	Normal to low	1 (3.0)	0
	Low to normal	2 (6.1)	0
Protein C activity	Normal to high	0	1 (3.6)
	High to normal	2 (6.1)	6 (21.4)

Source: Section 15.3, Table 15.3.2.14
n: Number of subjects with data available
%: Percentage based on n

Numbers of subjects with normal and abnormal haemostatic values are presented in Section 15.3, Table 15.3.2.8.

Carbohydrate metabolism

Table 64: Shifts of Carbohydrate Metabolism Parameters from Baseline to Endpoint (SS)

Variable	Shift	Test n (%)	Reference n (%)
	n	33	28
C-Peptide	Normal to high	4 (12.1)	2 (7.1)
	High to normal	2 (6.1)	3 (10.7)
Insulin	Normal to high	4 (12.1)	4 (14.3)
	High to normal	2 (6.1)	2 (7.1)
Plasma fasting glucose	n	448	179
	Normal to high	29 (6.5)	8 (4.5)
	Normal to low	8 (1.8)	1 (0.6)
	High to normal	16 (3.6)	13 (7.3)
	Low to normal	2 (0.4)	2 (1.1)

Source: Section 15.3, Table 15.3.2.15
n: Number of subjects with data available
%: Percentage based on n

A summary of carbohydrate metabolism parameters (C-peptide, insulin and plasma fasting glucose) is provided in Section 15.3, Table 15.3.2.4. No clinically relevant changes were observed in mean or median carbohydrate metabolism values over time.

The proportions of subjects with changes of carbohydrate metabolism variables from baseline to endpoint were comparable between the treatment groups, see Table 64. Numbers of subjects with normal and abnormal individual C-peptide, insulin and plasma fasting glucose levels are provided in Section 15.3, Table 15.3.2.9.

Bone metabolism parameters

A summary of bone metabolism parameters (bone alkaline phosphatase and cross-linked c-terminal telopeptides) is provided in Section 15.3, Table 15.3.2.5.

Bone alkaline phosphatase level decreased over time in both groups. In the Test group, mean (SD) value decreased from 415.7 (153.51) nkat/L at baseline to 325.4 (117.74) nkat/L at

endpoint (reference range: 193-493 nkat/L). In the Reference group it decreased from 393.6 (153.06) nkat/L to 358.0 (142.65) nkat/L. The between-group differences at baseline and at endpoint were not statistically significant (p = 0.5596 and p = 0.3250, respectively; 2-sample t test). The mean [SD] change from baseline at endpoint was more pronounced in the Test group than in the Reference group (-79.9 [126.11] nkat/L vs. -23.9 [108.65] nkat/L). The median change was -102.0 nkat/L vs. -30.0 nkat/L, respectively. The between-group difference in mean change from baseline to endpoint was not statistically significant (p = 0.0742, 2-sample t test).

The mean (SD) levels of Beta-CTX increased from baseline to endpoint in both groups: from 354.5 (207.51) ng/L to 366.9 (161.50) ng/L in the Test, and from 312.9 (215.71) ng/L to 363.0 (208.11) ng/L in the Reference group (reference range: 25-573 ng/L). The mean [SD] change was 39.9 [171.08] ng/L in the Test and 47.4 [217.92] ng/L in the Reference group). The median change was 34.0 or 55.5 ng/L, respectively. The between-group difference in mean change from baseline to endpoint was not statistically significant (p = 0.8810, 2-sample t test)

The levels of bone remodelling markers were within the range for premenopausal women, not treated with contraceptives and no statistically significant differences between the treatment groups were found. The detailed report on Recommendations and Proposals on Drospirenone, Bone and the CF111/302 Clinical Trial by José Luis Pérez-Castrillón MD, PhD and Antonio Dueñas-Laita MD, PhD, FACCP can be found in Appendix 16.1.13.

All individual values of bone metabolism parameters were assessed as normal in both treatment groups (Section 15.3, Tables 15.3.2.10 and 15.3.2.16).

Urinalysis

A summary of abnormal values based on urinalysis by dipstick is presented in Table 65 below.

The proportions of subjects with abnormal values were comparable between the treatment groups.

Table 65: Summary of Abnormal Urinalysis Values (SS)

Parameter	Visit	Test (N=858) n (%)	Reference (N=332) n (%)	Total (N=1190) n (%)
**Leucocytes**	Baseline	27 (3.1)	16 (4.8)	43 (3.6)
	Visit 3	25 (2.9)	9 (2.7)	34 (2.9)
	Visit 5/EDV	22 (2.6)	8 (2.4)	30 (2.5)
	Endpoint	24 (2.8)	9 (2.7)	33 (2.8)
**Protein**	Baseline	18 (2.1)	11 (3.3)	29 (2.4)
	Visit 3	9 (1.0)	1 (0.3)	10 (0.8)
	Visit 5/EDV	13 (1.5)	6 (1.8)	19 (1.6)
	Endpoint	13 (1.5)	7 (2.1)	20 (1.7)
**Blood**	Baseline	18 (2.1)	7 (2.1)	25 (2.1)
	Visit 3	14 (1.6)	6 (1.8)	20 (1.7)
	Visit 5/EDV	16 (1.9)	5 (1.5)	21 (1.8)
	Endpoint	17 (2.0)	6 (1.8)	23 (1.9)
**Haemoglobin**	Baseline	11 (1.3)	9 (2.7)	20 (1.7)
	Visit 3	12 (1.4)	6 (1.8)	18 (1.5)
	Visit 5/EDV	7 (0.8)	6 (1.8)	13 (1.1)
	Endpoint	8 (0.9)	7 (2.1)	15 (1.3)
**Nitrites**	Baseline	8 (0.9)	2 (0.6)	10 (0.8)
	Visit 3	3 (0.3)	0 (0.0)	3 (0.3)
	Visit 5 (EDV) /Endpoint	2 (0.2)	1 (0.3)	3 (0.3)
**pH 8**	Baseline	5 (0.6)	4 (1.2)	9 (0.8)
	Visit 3	1 (0.1)	1 (0.3)	2 (0.2)
	Visit 5 (EDV) /Endpoint	2 (0.2)	1 (0.3)	3 (0.3)
**pH 9**	Visit 5 (EDV) /Endpoint	1 (0.1)	0 (0.0)	1 (0.1)
**Ketones**	Baseline	4 (0.5)	4 (1.2)	8 (0.7)
	Visit 3	1 (0.1)	1 (0.3)	2 (0.2)
	Visit 5 (EDV) /Endpoint	1 (0.1)	0 (0.0)	1 (0.1)
**Glucose**	Baseline	3 (0.3)	1 (0.3)	4 (0.3)
**Bilirubin**	Baseline	0 (0.0)	2 (0.6)	2 (0.2)
	Visit 3	1 (0.1)	0 (0.0)	1 (0.1)
**Urobilinogen**	Baseline	0 (0.0)	2 (0.6)	2 (0.2)

Source: Section 15.3, Table 15.3.2.11
N: Number of subjects in specified treatment group
n: Number of subjects with data available
%: Percentage based on N

12.5.2.2 Individual clinically significant abnormalities

No clinically significant values were reported for special laboratory parameters: haemostatic, carbohydrate metabolism or bone metabolism variables. As concerns haematology, biochemistry variables and TSH, the frequency of abnormal CS values at any post-baseline assessments was low.

Clinically significant (CS) abnormalities in laboratory parameters were documented as TEAEs, classified mainly in the "Investigations" SOC. A summary of subjects with abnormal laboratory findings that led to the reporting of TEAEs is provided in Table 66 below.

Table 66: TEAEs Based on Individual Clinically Significant Laboratory Abnormalities / MedDRA Coding (SS)

System Organ Class [a]<br>Preferred Term	Test<br>(N=858)<br>n (%)	Reference<br>(N=332)<br>n (%)	Total<br>(N=1190)<br>n (%)
**Investigations**			
Blood thyroid stimulating hormone increased	8 (0.9)	2 (0.6)	10 (0.8)
Gamma-glutamyltransferase increased	5 (0.6)	0	5 (0.4)
Alanine aminotransferase increased	4 (0.5)	0	4 (0.3)
Aspartate aminotransferase increased	3 (0.3)	0	3 (0.3)
White blood cells urine	3 (0.3)	0	3 (0.3)
Blood thyroid stimulating hormone decreased	2 (0.2)	0	2 (0.2)
Blood bilirubin increased	0	1 (0.3)	1 (0.1)
Blood creatine phosphokinase increased	1 (0.1)	0	1 (0.1)
Blood creatinine increased	1 (0.1)	0	1 (0.1)
Blood lactate dehydrogenase increased	1 (0.1)	0	1 (0.1)
Blood potassium increased	1 (0.1)	0	1 (0.1)
Hepatic enzyme increased	0	1 (0.3)	1 (0.1)
Transaminases increased	0	1 (0.3)	1 (0.1)
White blood cell count decreased	0	1 (0.3)	1 (0.1)
**Blood and lymphatic system disorders**			
Iron deficiency anaemia	1 (0.1)	-	1 (0.1)
Anaemia	0	1 (0.3)	1 (0.1)
**Metabolism and nutrition disorders**			
Hyperkalaemia	1 (0.1)	0	1 (0.1)

Source: Section 15.3, Table 15.3.1.2
N: Number of subjects in specified treatment group.
n: Number of subjects with adverse events.
%: Percentage based on N

The most frequent laboratory TEAEs were related to elevated blood thyroid stimulating hormone levels. TEAEs of blood TSH increased were reported for 10 subjects (0.8%), all of them were mild to moderate in intensity. The majority of these TEAEs were assessed as not or unlikely to be related to IMP, except two possibly related cases in the Test group (Subjects #568018 and #658054).

Blood potassium increased (Subject #454004) and hyperkalaemia (Subject #252013) were classified as SAEs due to meeting the criterion of special interest AEs though the subjects did not present clinical signs of hyperkalaemia, see Section 12.3. Narratives of these SAEs are provided in Section 15.3.3.

12.6 Vital Signs, Physical Findings and Other Observations Related to Safety

12.6.1 Vital signs

Body weight, blood pressure and heart rate were measured at screening, V4 and V6 (EDV). Subjects' height was measured at screening. Body mass index (BMI) was calculated as weight / height^2. For individual data on vital signs, please refer to Appendix 16.2, Listing 16.2.4.1. For all vital signs data, presented in this section, baseline value was defined as the last non-missing value collected before the first IMP administration. Endpoint value was defined as the last non-missing value.

12.6.1.1 Body weight and body mass index

As shown in Table 67 below, the mean (SD) weight change from baseline to endpoint was slightly lower in the Test group than in the Reference group (0.1 [3.2] kg vs. 0.5 [3.2] kg), the difference between the groups was statistically significant (p=0.0296, ANCOVA).

Respectively, mean (SD) BMI in the Test group increased by 0.04 (1.17) kg/m^2, and that of the Reference group by 0.20 (1.11) kg/m^2, with a statistically significant difference (p = 0.0331, ANCOVA). The median weight and BMI changes were 0.0 in both groups.

Table 67: Summary of Body Weight and BMI (SS)

Visit		Body weight (kg)		BMI (kg/m^2)	
		Test (N=858)	Reference (N=332)	Test (N=858)	Reference (N=332)
Baseline	n	858	332	858	332
	Mean (SD)	63.4 (10.5)	63.3 (11.5)	22.96 (3.54)	22.82 (3.90)
	Median	61.0	62.0	22.30	22.05
	Min/Max	42/114	42/110	16.6/41.0	15.9/38.0
Visit 2	n	835	325	835	325
	Mean (SD)	63.4 (10.4)	63.7 (11.7)	22.94 (3.53)	22.96 (3.94)
	Median	61.0	62.0	22.40	22.20
	Min/Max	42/112	42/110	16.6/41.9	16.3/38.0
Visit 3	n	775	286	775	286
	Mean (SD)	63.0 (10.1)	63.8 (11.9)	22.81 (3.41)	22.99 (4.02)
	Median	61.0	62.0	22.30	22.10
	Min/Max	41/112	40/110	16.6/39.1	15.9/36.9
Visit 4	n	713	256	713	256
	Mean (SD)	63.2 (10.2)	64.0 (12.0)	22.87 (3.44)	23.05 (4.02)
	Median	61.0	61.5	22.30	22.15
	Min/Max	42/113	40/110	16.4/39.8	16.0/36.0
Visit 5/EDV	n	820	315	820	315
	Mean (SD)	63.6 (10.7)	64.0 (11.6)	23.02 (3.67)	23.04 (3.94)
	Median	62.0	62.0	22.40	22.30
	Min/Max	43/114	43/112	15.9/41.9	16.3/38.9
Endpoint	n	853	328	853	328
	Mean (SD)	63.6 (10.7)	63.9 (11.8)	23.01 (3.66)	23.05 (4.01)
	Median	62.0	62.0	22.30	22.30
	Min/Max	43/114	43/112	15.9/41.9	16.3/38.9
Change from baseline at endpoint	n	853	328	853	328
	Mean (SD)	0.1 (3.2)	0.5 (3.1)	0.04 (1.17)	0.20 (1.11)
	Median	0.0	0.0	0.00	0.00
	Min/Max	-24/14	-9/22	-8.9/5.2	-3.3/6.8
	p-value [a]		p=0.0296		p = 0.0331
Relative change from baseline at endpoint	n	853	328	853	328
	Mean (SD)	0.2 (4.9)	0.9 (4.9)	0.20 (4.90)	0.95 (4.91)
	Median	0.0	0.0	0.00	0.00
	Min/Max	-25/24	-12/25	-25.2/24.1	-11.9/25.0

Source: Section 15.3, Table 15.3.3.1
N: Number of subjects in specified treatment group
n: Number of subjects with data available
SD: Standard deviation
[a]: ANCOVA with age and baseline value as covariates and treatment group as a fixed factor.

TEAEs related to weight gain were reported for 24 (2.7%) Test group and six (1.8%) Reference group subjects (Table 68). All these TEAEs were assessed by the investigators as being mild or moderate in intensity.

The majority of these TEAEs were assesed as at least possibly related to study treatment.

None of the subjects discontinued prematurely due to weight gain TEAE.

Four Test group subjects reported weight decreased TEAEs, which were assessed as being not related to IMP.

Table 68: Incidence of TEAEs Based on Weight Changes (SS)

Preferred Term	Test (N=858)			Reference (N=332)		
	Total n (%)	Related* n (%)	Leading to early discontinuation n (%)	Total n (%)	Related n (%)	Leading to early discontinuation n (%)
Weight increased	21 (2.4)	19 (2.2)	8 (0.9)	6 (1.8)	6 (1.8)	3 (0.9)
Obesity	3 (0.3)	2 (0.2)	0	0	0	0
Weight decreased	4 (0.5)	0	0	0	0	0
Total	28 (3.2)	21 (2.4)	8 (0.9)	6 (1.8)	6 (1.8)	3 (0.9)

Source: Section 15.3, Tables 15.3.1.2, 15.3.1.4 and 15.3.1.8
N: Number of subjects in specified treatment group
n: Number of subjects with TEAEs
%: Percentage based on N
* Related was defined as at least possibly related.

12.6.1.2 Body weight and body mass index by subgroups

Age subgroups

Table 69: Body Weight and BMI Changes from Baseline to Endpoint by Age Subgroups (SS)

		≤ 35 years		> 35 years	
		Test	Reference	Test	Reference
**Weight (kg)**	n	677	255	176	73
	Mean (SD)	0.0 (3.3)	0.7 (3.3)	0.3 (2.6)	0.1 (2.7)
	Median	0.0	0.0	0.0	1.0
	Min/Max	-24/14	-9/22	-10/12	-8/7
**BMI (kg/m^2)**	n	677	255	176	73
	Mean (SD)	0.02 (1.23)	0.24 (1.14)	0.11 (0.95)	0.04 (0.96)
	Median	0.00	0.00	0.00	0.30
	Min/Max	-8.9/5.2	-3.3/6.8	-3.7/4.3	-2.8/2.5

Source: Section 15.3, Table 15.3.3.2
N: Number of subjects in specified treatment group
n: Number of subjects with data available
SD: standard deviation

Body weight and BMI at baseline were comparable between both age groups. No relevant changes were reported in the age ≤ 35 years groups over time. The median values of body weight and BMI absolute change from baseline to endpoint were 0.00 in the Test and in the Reference group.

In the > 35 years subgroup, some changes were seen. In the Test group median weight and BMI change was 0.0, whereas in the Reference group a small median increase in weight (change: 1.0 kg) and BMI (change: 0.30 kg/m^2) was observed.

BMI subgroups

Table 70: Body Weight and BMI Changes from Baseline to Endpoint by BMI Subgroups (SS)

		BMI<30 kg/m^2		BMI≥30 kg/m^2	
		**Test**	**Reference**	**Test**	**Reference**
**Weight (kg)**	n	823	312	30	16
	Mean (SD)	0.1 (3.0)	0.5 (3.1)	-0.2 (6.5)	0.9 (3.2)
	Median	0.0	0.0	0.0	0.5
	Min/Max	-13/14	-9/22	-24/13	-6/6
**BMI (kg/m^2)**	n	823	312	30	16
	Mean (SD)	0.04 (1.11)	0.19 (1.10)	-0.07 (2.41)	0.33 (1.20)
	Median	0.00	0.00	0.00	0.20
	Min/Max	-5.0/5.2	-3.3/6.8	-8.9/4.8	-2.1/2.4

Source: Section 15.3, Table 15.3.3.3
N: Number of subjects in specified treatment group
n: Number of subjects with data available
SD: standard deviation

No body weight or BMI changes over time were observed for the BMI<30kg/m^2 subgroup: The absolute median changes of body weight and BMI from baseline to endpoint were 0.0 in both treatment groups (Table 70). Some differences for the BMI ≥ 30 kg/m^2 subgroup were noted: In the Test group median changes of body weight and BMI were 0.0, whereas in the Reference group median weight change was 0.5 kg, and that of BMI was 0.20 kg/m^2. However, due to the small number of subjects in the BMI ≥ 30 kg/m^2 subgroup, the relevance of these differences is limited.

Blood pressure subgroups

The mean and median body weight and BMI values were comparable in the group of subjects with SBP≥130 mmHg or DBP≥85 mmHg at baseline. No relevant changes over time were reported in the lower blood pressure subgroup: The median changes of body weight and BMI from baseline to endpoint were 0.0 for both treatment groups (Table 71).

For the subgroup of SBP≥130 mmHg or DBP≥85 mmHg, a small mean and median increase of body mass and BMI was seen in both groups. Median weight changes of 0.8 kg in the Test and of 1.0 kg in the Reference group were observed. The median change of BMI was 0.25 kg/m^2 vs. 0.30 kg/m^2, respectively.

Table 71: Body Weight and BMI Changes from Baseline to Endpoint by Blood Pressure Subgroups (SS)

		SBP<130 mmHg and DBP<85 mmHg		SBP≥130 mmHg or DBP≥85 mmHg	
		**Test**	**Reference**	**Test**	**Reference**
**Weight (kg)**	n	723	287	130	41
	Mean (SD)	-0.0 (3.1)	0.5 (3.2)	0.5 (3.8)	0.7 (2.8)
	Median	0.0	0.0	0.8	1.0
	Min/Max	-13/14	-9/22	-24/13	-6/7
**BMI (kg/m^2)**	n	723	287	130	41
	Mean (SD)	0.01 (1.13)	0.19 (1.12)	0.20 (1.40)	0.27 (1.05)
	Median	0.00	0.00	0.25	0.30
	Min/Max	-5.0/5.2	-3.3/6.8	-8.9/5.2	-2.1/2.7

Source: Section 15.3, Table 15.3.3.4
N: Number of subjects in specified treatment group
n: Number of subjects with data available
SD: Standard deviation

12.6.1.3 Blood pressure and heart rate

Blood pressure and heart rate

Three measurements of systolic and diastolic blood pressure and heart rate were performed at screening, V2, V3, V4 and V5 (EDV) with 1 min pause between them after 5 minutes of rest in a sitting position. The summary of blood pressure is provided in Table 72.

The treatment groups were comparable with respect to the mean and median systolic and diastolic blood pressure values.

No relevant changes in systolic and diastolic blood pressure from baseline at endpoint were observed in each treatment group during the trial (median change: 0.0 mmHg).

Table 72: Blood Pressure (SS)

Visit		SBP (mmHg)		DBP (mmHg)	
		Test (N=858)	Reference (N=332)	Test (N=858)	Reference (N=332)
Baseline	n	858	332	858	332
	Mean (SD)	115.5 (10.4)	115.3 (10.0)	72.4 (8.0)	72.1 (7.8)
	Median	116.0	115.0	72.0	71.0
	Min/Max	80/142	76/138	50/95	53/90
Visit 2	n	836	325	836	325
	Mean (SD)	114.9 (9.7)	114.7 (9.2)	71.2 (7.8)	71.0 (7.9)
	Median	115.0	115.0	70.0	70.0
	Min/Max	87/142	90/136	46/96	49/95
Visit 3	n	776	286	776	286
	Mean (SD)	113.3 (10.0)	112.6 (9.9)	71.2 (7.4)	70.0 (7.5)
	Median	115.0	113.0	70.0	70.0
	Min/Max	75/140	80/140	49/91	50/97
Visit 4	n	713	256	713	256
	Mean (SD)	113.3 (10.0)	113.5 (10.5)	70.6 (7.9)	70.3 (7.9)
	Median	113.0	114.0	70.0	70.0
	Min/Max	80/140	80/140	40/92	50/90
Visit 5/EDV	n	818	315	818	315
	Mean (SD)	114.2 (9.7)	114.0 (10.2)	70.6 (7.6)	70.7 (7.8)
	Median	115.0	115.0	70.0	70.0
	Min/Max	82/140	90/142	50/97	55/94
Endpoint	n	853	328	853	328
	Mean (SD)	114.0 (9.7)	114.0 (10.2)	70.6 (7.6)	70.7 (7.8)
	Median	115.0	115.0	70.0	70.0
	Min/Max	82/140	90/142	50/97	55/94
Change from Baseline at Endpoint	n	853	328	853	328
	Mean (SD)	-1.5 (10.2)	-1.3 (10.0)	-1.8 (8.1)	-1.4 (8.1)
	Median	0.0	0.0	0.0	0.0
	Min/Max	-35/33	-30/40	-35/26	-22/30
	p-value [a]		p=0.8048		p = 0.6666
Relative change from Baseline at Endpoint	n	853	328	853	328
	Mean (SD)	-0.9 (9.0)	-0.7 (8.9)	-1.8 (11.2)	-1.3 (11.4)
	Median	0.0	0.0	0.0	0.0
	Min/Max	-28/40	-23/44	-39/41	-26/50
	p-value [a]		p=0.9490		p =0.7341

Source: Section 15.3, Table 15.3.3.1
N: Number of subjects in specified treatment group
n: Number of subjects with data available
SD: Standard deviation
Note [a] ANCOVA with age and baseline value as covariates and treatment group as a fixed factor

Heart rate

Table 73: Summary of Heart Rate (bpm) (SS)

		Test (N=858)	Reference (N=332)	Total (N=1190)
Baseline	n	856	332	1188
	Mean (SD)	73.5 (9.0)	72.8 (8.2)	73.3 (8.8)
	Median	73.0	72.0	72.0
	Min/ Max	51/117	54/98	51/117
Visit 2	n	835	325	1160
	Mean (SD)	75.3 (9.6)	74.2 (9.2)	75.0 (9.5)
	Median	75.0	74.0	75.0
	Min/ Max	51/111	51/110	51/111
Visit 3	n	776	286	1062
	Mean (SD)	74.8 (9.1)	73.8 (8.5)	74.6 (9.0)
	Median	74.0	74.0	74.0
	Min/ Max	52/123	52/97	52/123
Visit 4	n	713	256	969
	Mean (SD)	74.8 (8.5)	74.2 (9.2)	74.6 (8.7)
	Median	75.0	73.0	74.0
	Min/ Max	55/103	54/106	54/106
Visit 5/EDV	n	818	314	1132
	Mean (SD)	74.9 (9.1)	74.6 (9.3)	74.8 (9.2)
	Median	74.0	74.0	74.0
	Min/ Max	50/109	55/114	50/114
Endpoint	n	853	328	1181
	Mean (SD)	74.8 (9.1)	74.7 (9.2)	74.7 (9.1)
	Median	74.0	74.0	74.0
	Min/ Max	50/109	55/114	50/114
Change from Baseline at Endpoint	n	851	328	1179
	Mean (SD)	1.2 (8.9)	1.8 (8.9)	1.4 (8.9)
	Median	1.0	2.0	1.0
	Min/ Max	-35/33	-26/45	-35/45
Relative change from Baseline at Endpoint	n	851	328	1179
	Mean (SD)	2.4 (12.1)	3.1 (12.3)	2.6 (12.2)
	Median	1.3	2.6	1.4
	Min/ Max	-35/46	-29/69	-35/69

Source: Section 15.3, Table 15.3.3.1
N: Number of subjects in specified treatment group
n: Number of subjects with data available
SD: Standard deviation
Notes: [a] 2-sample t test

The treatment groups were comparable with respect to the mean and median heart rate values both at baseline and at endpoint. A slight median increase of 1.0 bpm in the Test group and 2.0 bpm in the Reference group was observed at final examination as compared to baseline.

Based on individual blood pressure changes, five TEAEs were reported. In the Test group, blood pressure fluctuation (Subject #371025; AE of moderate intensity, possibly related to IMP), blood pressure systolic increased (Subject #371005; AE of mild intensity, not related to IMP) and hypertension (Subject #551006; AE of mild intensity, unlikely to be related to IMP, concomitant medication not prescribed) were reported. In the Reference group hypertension (Subject #551004; AE of mild intensity, possibly related to IMP) and orthostatic

hypotension SAE (Subject # 263022; SAE of moderate intensity, not related to IMP) were reported. A narrative of this SAE is provided in Section 15.3.3.

All subjects recovered.

Based on individual heart rate changes, two TEAEs were reported: Two Test group subjects had tachychardia: Subject #463003 (AE of mild intensity, unlikely to be related to IMP, no action taken, AE resolved) and Subject #366005 (AE of mild intensity, possibly related to IMP, no action taken, outcome unknown).

12.6.1.4 Blood pressure and heart rate by subgroups

Age subgroups

Table 74: Blood Pressure and Heart Rate Changes from Baseline to Endpoint by Age Subgroups (SS)

		≤ 35 years		> 35 years	
		**Test**	**Reference**	**Test**	**Reference**
**SBP (mmHg)**	n	677	255	176	73
	Mean (SD)	-1.1 (10.3)	-1.3 (10.0)	-2.9 (9.7)	-1.0 (9.9)
	Median	0.0	-1.0	-0.5	0.0
	Min/Max	-30/33	-30/32	-35/20	-30/40
**DBP (mmHg)**	n	677	255	176	73
	Mean (SD)	-1.5 (8.2)	-1.6 (8.4)	-2.8 (7.8)	-0.7 (6.9)
	Median	0.0	0.0	-2.0	0.0
	Min/Max	-35/26	-22/30	-30/14	-17/20
**Pulse (bpm)**	n	675	255	176	73
	Mean (SD)	1.3 (9.4)	1.7 (9.5)	1.0 (6.7)	2.4 (6.2)
	Median	1.0	2.0	1.0	2.0
	Min/Max	-35/33	-26/45	-23/18	-17/20

Source: Section 15.3, Table 15.3.3.2
N: Number of subjects in specified treatment group
n: Number of subjects with data available
SD: standard deviation

Changes of systolic and diastolic blood pressure, and heart rate from baseline at endpoint by age subgroups are provided in Table 74. Both age groups were comparable with respect to mean and median blood pressure and heart rate at baseline. No relevant changes over time in mean and median blood pressure values were observed in the ≤35 years subgroup. In the subgroup >35 years, a median change of -2.0 mmHg was observed in the Test group for diastolic blood pressure, as compared with 0.0 in the Reference group.

BMI subgroups

Table 75: Blood Pressure and Heart Rate Changes from Baseline to Endpoint by BMI Subgroups (SS)

		BMI<30 kg/m^2		BMI≥30 kg/m^2	
		**Test**	**Reference**	**Test**	**Reference**
**SBP (mmHg)**	n	823	312	30	16
	Mean (SD)	-1.5 (10.2)	-1.5 (10.0)	-2.7 (9.7)	2.8 (8.9)
	Median	0.0	0.0	0.0	4.5
	Min/Max	-35/33	-30/40	-27/10	-13/21
**DBP (mmHg)**	n	823	312	30	16
	Mean (SD)	-1.8 (8.0)	-1.5 (8.2)	-2.2 (10.8)	0.1 (6.4)
	Median	0.0	0.0	-0.5	0.0
	Min/Max	-33/26	-22/30	-35/22	-12/14
**Pulse (bpm)**	n	822	312	29	16
	Mean (SD)	1.2 (9.0)	1.8 (9.0)	0.2 (7.0)	1.9 (7.3)
	Median	1.0	2.0	0.0	1.0
	Min/Max	-35/33	-26/45	-13/16	-12/20

Source: Section 15.3, Table 15.3.3.3
N: Number of subjects in specified treatment group
n: Number of subjects with data available
SD: standard deviation

At baseline, mean and median values of blood pressure and heart rate were comparable between the treatment groups (Section 15.3, Table 15.3.3.3). For both subgroups, no relevant changes were observed over time in mean and median diastolic blood pressure values (Table 75). For the high-BMI subgroup a median increase of 4.5 mmHg in systolic blood pressure was reported at endpoint in the Reference group, whereas the median change in the Test group was 0.0. However, due to the small number of subjects in the BMI ≥ 30 kg/m^2 subgroup, the relevance of this difference is limited.

No relevant differences in heart rate changes between the treatment groups were seen in any BMI subgroup.

Blood pressure subgroups

Table 76: Blood Pressure and Heart Rate Changes from Baseline to Endpoint by Blood Pressure Subgroups (SS)

		SBP<130 mmHg and DBP<85 mmHg		SBP≥130 mmHg or DBP≥85 mmHg	
		**Test**	**Reference**	**Test**	**Reference**
**SBP (mmHg)**	n	723	287	130	41
	Mean (SD)	-0.3 (10.0)	-0.2 (9.6)	-8.3 (8.6)	-8.8 (9.3)
	Median	0.0	0.0	-7.0	-8.0
	Min/Max	-35/33	-24/40	-30/12	-30/9
**DBP (mmHg)**	n	723	287	130	41
	Mean (SD)	-0.8 (7.7)	-0.9 (7.9)	-7.2 (8.4)	-5.2 (8.5)
	Median	0.0	0.0	-5.5	-5.0
	Min/Max	-25/26	-22/30	-35/10	-20/20
**Pulse (bpm)**	n	721	287	130	41
	Mean (SD)	1.5 (8.6)	1.9 (8.5)	-0.5 (10.1)	1.1 (11.4)
	Median	1.0	2.0	0.0	1.0
	Min/Max	-32/33	-24/45	-35/30	-26/26

Source: Section 15.3, Table 15.3.3.4
N: Number of subjects in specified treatment group
n: Number of subjects with data available
SD: standard deviation

In the SBP < 130 mmHg and DBP <85 mmHg subgroup, no relevant changes over time were seen with regard to systolic or diastolic blood pressure (median change: 0.0 for SBP and DBP in both groups) or heart rate (median increase of 1.0 bpm in the Test and of 2.0 bpm in the Reference group).

In the subgroup of subjects with SBP≥130 mmHg or DBP≥85 mmHg at screening, blood pressure decreased over time, which may be (partially) attributed to the statistical phenomenon “regression toward the mean”: The median change in SBP from baseline at endpoint was -7.0 mmHg (mean change: -8.3 [8.6] mmHg) in the Test and -8.0 mmHg (mean change: -8.8 [9.3] mmHg) in the Reference group. The median change of DBP was -5.5 mmHg in the Test and -5.0 mmHg in the Reference group. Mean changes were: -7.2 (8.4) mmHg vs. -5.2 (8.5) mmHg, respectively.

12.6.2 Gynaecological examination

At screening and at Visit 5/EDV, the subjects underwent gynaecological examination. Any CS findings were to be documented either as a medical history finding or as an AE. A by- subject listing of all subjects with gynaecological examination results is provided in Appendix 16.2, Listing 16.2.5.1. Shifts of gynaecological examination findings from screening to Visit 5 or EDV are summarised in Section 15.3, Table 15.3.4.2.

No CS abnormal findings were documented for external genitalia and breasts at any scheduled visit.

The incidence of abnormal findings assessed as CS by the investigators was low. The most frequent CS findings were related to speculum examination and were documented for five Test group and four Reference group subjects, see Section 15.3, Table 15.3.4.1.

12.6.3 Cervical smear examination

The by-subject listing of the cervical cytology results at screening and Visit V5 or EDV for all subjects is provided in Appendix 16.2, Listing 16.2.5.2. Subjects with positive test results (ASC-US or worse) at screening were not to be included in the trial. Cervical cytology at screening revealed normal or inflammatory results for 99.7% of the Test group and 99.4% of the Reference group subjects (Table 77). No major differences were seen between the treatment groups with regard to the frequency of abnormal cervical smear examination findings.

Table 77: Summary of Cervical Smear Examination (SS)

Cervical smear result	Test (N=858)		Reference (N=332)	
	Screening Visit n (%)	Visit 5/EDV n (%)	Screening Visit n (%)	Visit 5/EDV n (%)
Normal / inflammatory	855 (99.7)	773 (90.1)	330 (99.4)	293 (88.3)
ASC-US	0	10 (1.2)	1 (0.3)	4 (1.2)
ASC-H and AGUS - cannot exclude high-grade disease or cancer	0	1 (0.1)	0	1 (0.3)
CIN 1	0	16 (1.9)	0	5 (1.5)
CIN 2	0	0	0	1 (0.3)
CIN 3 – carcinoma in situ	0	1 (0.1)	0	0
Carcinoma in situ – microinvasive carcinoma	0	0	0	0
Microinvasive carcinoma – invasive carcinoma	0	0	0	0
Assessment not performed	0	0	0	0

Source: Section 15.3, Table 15.3.4.3
N: Number of subjects in specified treatment group
n: Number of subjects with data available
%: Percentage based on N

12.6.4 Intravaginal ultrasound examination

Intravaginal ultrasound examination was performed at screening and V5 (EDV). Any CS findings were documented either as medical history findings or as AEs. Shifts of ultrasound examination findings from screening to final examination are provided in Section 15.3, Table 15.3.4.5. The individual ultrasound examination results are listed in Appendix 16.2, Listing 16.2.5.3.

The frequency of abnormal findings based on intravaginal ultrasound examination was low. The most frequently reported were abnormal CS findings of the ovaries (eight subjects, 0.9% in the Test and one subject, 0.3% in the Reference group) at V5/EDV, see Section 15.3, Table 15.3.4.4.

12.6.5 Physical examination

Physical examination was performed at screening and V5 (or EDV). By-subject physical examination findings are listed in Appendix 16.2, Listing 16.2.6.1. The incidence of physical examination findings assessed as CS was low, see Table 78. The most frequent findings in both groups were skin abnormalities, mainly acne, which was documented for eight persons in each treatment group (Appendix 16.2, Listing 16.2.6.1).

Table 78: Frequency of Abnormal Physical Examination Findings Assessed as Clinically Significant (SS)

Assessed parameters	Test (N=858) n (%)	Reference (N=332) n (%)	Total n (%)
Skin	11 (1.3)	8 (2.4)	19 (1.6)
Thyroid	1 (0.1)	0	1 (0.1)
General appearance	1 (0.1)	1 (0.3)	2 (0.2)

Source: Section 15.3, Table 15.3.5.1
N: Number of subjects in specified treatment group
n: Number of subjects with data available
%: Percentage based on N

Shifts of physical examination findings from screening to Visit 5/EDV are provided in Section 15.3, Table 15.3.5.2.

12.6.6 12-lead ECG

12-lead ECG was performed for a subset of subjects at Visit 1b and Visit 5/EDV. ECG parameters assessed for each subject are presented in Appendix 16.2, Listing 16.2.6.3.

The following variables related to ECG were collected for a subset of 151 Test group and 56 Reference group subjects: summary (mean) heart rate, RR, PR and QRS duration, QT duration, QTcB – Bazett's correction formula and QTcF-Fridiricia's correction formula. These ECGs were centrally evaluated by ERT (for contact details, see Section 6). The project requirement specification from ERT is provided in Appendix 16.1.3.

In the Test group a numerical (but not statistically significant) decrease in heart rate and an increase in RR duration were observed.

At Visit 1b, summary mean (SD) QRS duration was comparable between the treatment grpups: 90.9 (8.1) ms in the Test and 89.6 (8.3) ms in the Reference group. Statistically significant differences in mean (SD) QRS duration between the treatment groups were seen at Visit 5/EDV: 92.0 (8.4) ms in the Test vs. 88.4 (8.6) ms in the Reference group (LS mean difference of 3.58 ms, 90% CI: 1.40; 5.77) and with regard to the mean (SD) change from Visit 1b to Visit 5/EDV: 1.5 (5.4) ms in the Test vs. -1.1 (5.0) ms in the Reference group (LS mean difference of 2.55 ms, 90% CI: 1.13; 3.97). Nevertheless, these differences were not clinically significant.

In the Test group, the mean (SD) QT interval changed from 383 (22.1) ms at V1b to 390.8 (23.0) ms at V5/EDV. In the Reference group it changed from 385.3 (19.9) to 384.3 (27.1). No statistically significant differences at both visits were observed between the treatment groups. The mean (SD) QT duration changed by 8.0 (22.0) ms in the Test group and by -0.9 (20.5) ms in the Reference group. The LS mean difference of 8.90 ms between the groups was statistically significant (90% 2-sided CI: 3.13; 14.68). However, as there was also observed some difference between groups in the changes of the heart rate and RR duration, the corrected QT intervals (QTcB and QTcF) should be considered for interpretation.

The mean (SD) QTcB interval increased by 0.7 (15.8) ms in the Test group and decreased by -1.5 (20.7) ms in the Reference group, and the difference of 2.19 ms between the groups' changes was not statistically signifcant (90% 2-sided CI: -2.44; 6.82).

The mean QTcF interval increased by 3.2 (12.3) ms in the Test group and decreased by -1.3 (14.5) ms in the Reference group. The difference of 4.55 ms between the groups' changes was statistically signifcant (90% 2-sided CI: 1.09; 8.02). However, such change (4.55 ms) is below the threshold of regulatory concern (around 5 ms) given that it is not associated with an increased risk of torsade de pointes according to the Note for Guidance on

the Clinical Evaluation of QT/QTc Interval Prolongation and Proarrhythmic Potential for Non-Antiarrhythmic Drugs) [10].

Table 79: Summary of ECG Parameters (SS)

Parameter	Visit		Test (N=858)	Reference (N=332)	LS Mean Difference 90% CI
**Summary (mean) Heart rate (bpm)**	Visit 1b	n	151	56	
		Mean (SD)	72.9 (9.4)	72.6 (10.1)	0.31 [-2.17; 2.80]
		Median	73.0	71.5	
		Min/ Max	51/106	49/102	
	Visit 5/EDV	n	138	58	
		Mean (SD)	70.7 (10.7)	72.7 (12.3)	-1.97 [-4.86; 0.92]
		Median	71.0	70.5	
		Min/ Max	50/102	49/104	
	Change from V1b	n	132	54	
		Mean (SD)	-2.5 (10.3)	0.3 (11.7)	-2.81 [-5.67; 0.05]
		Median	-3.0	0.0	
		Min/ Max	-34/18	-28/32	
**Summary (mean) RR Duration (ms)**	Visit 1b	n	151	56	
		Mean (SD)	830.8 (109.1)	836.5 (117.2)	-5.66 [-34.43; 23.11]
		Median	821.0	832.5	
		Min/ Max	564/1162	586/1223	
	Visit 5/EDV	n	138	58	
		Mean (SD)	861.7 (133.4)	842.0 (139.3)	19.68 [-15.27; 54.64
		Median	835.0	843.0	
		Min/ Max	586/1200	572/1218	
	Change from V1b	n	132	54	
		Mean (SD)	35.5 (121.8)	2.3 (125.9)	33.19 [0.33; 66.04]
		Median	29.5	0.5	
		Min/ Max	-198/391	-306/260	
**Summary (mean) PR duration (ms)**	Visit 1b	n	151	56	
		Mean (SD)	150.0 (26.0)	150.2 (18.7)	-0.22 [-6.50; 6.06]
		Median	147.0	150.0	
		Min/ Max	91/305	115/207	
	Visit 5/EDV	n	138	58	
		Mean (SD)	151.1 (21.4)	150.8 (17.8)	0.35 [-4.93; 5.63]
		Median	149.5	152.5	
		Min/ Max	105/242	111/198	
	Change from V1b	n	132	54	
		Mean (SD)	-0.6 (17.6)	-0.2 (11.6)	
		Median	0.0	0.0	-0.41 [-4.72; 3.90]
		Min/ Max	-117/73	-41/35	
**Summary (mean) QRS duration (ms)**	Visit 1b	n	151	56	
		Mean (SD)	90.9 (8.1)	89.6 (8.3)	1.31 [-0.79; 3.42]
		Median	90.0	89.0	
		Min/ Max	74/118	70/107	
	Visit 5/EDV	n	138	58	
		Mean (SD)	92.0 (8.4)	88.4 (8.6)	3.58 [1.40; 5.77]
		Median	92.0	87.5	
		Min/ Max	71/115	68/106	
	Change from V1b	n	132	54	
		Mean (SD)	1.5 (5.4)	-1.1 (5.0)	2.55 [1.13; 3.97]
		Median	1.0	0.0	
		Min/ Max	-13/20	-12/8	

Table 79: Summary of ECG Parameters (SS) (continued)

Parameter	Visit		Test (N=858)	Reference (N=332)	LS Mean Difference 90% CI
**Summary (mean) QT duration (ms)**	Visit 1b	n	151	56	
		Mean (SD)	383.3 (22.1)	385.3 (19.9)	-1.96 [-7.52; 3.60]
		Median	382.0	384.5	
		Min/ Max	332/437	335/438	
	Visit 5/EDV	n	138	58	
		Mean (SD)	390.8 (23.0)	384.3 (27.1)	6.50 [0.22; 12.78]
		Median	389.0	387.0	
		Min/ Max	330/454	317/442	
	Change from V1b	n	132	54	
		Mean (SD)	8.0 (22.0)	-0.9 (20.5)	8.90 [3.13; 14.68]
		Median	6.0	3.5	
		Min/ Max	-43/70	-49/51	
**Summary (mean) QTcB - Bazett's Correction Formula (ms)**	Visit 1b	n	151	56	
		Mean (SD)	421.5 (17.3)	422.8 (20.5)	-1.33 [-6.05; 3.38]
		Median	420.0	420.5	
		Min/ Max	377/473	382/466	
	Visit 5/EDV	n	138	58	
		Mean (SD)	422.7 (19.2)	420.6 (17.5)	2.14 [-2.70; 6.98]
		Median	423.0	418.5	
		Min/ Max	373/483	392/463	
	Change from V1b	n	132	54	
		Mean (SD)	0.7 (15.8)	-1.5 (20.7)	2.19 [-2.44; 6.82]
		Median	-1.0	-5.0	
		Min/ Max	-44/40	-41/61	
**Summary (mean) QTcF - Fridericia's Correction Formula (ms)**	Visit 1b	n	151	56	
		Mean (SD)	408.0 (14.7)	409.5 (15.1)	-1.56 [-5.40; 2.27]
		Median	407.0	410.0	
		Min/ Max	374/461	370/436	
	Visit 5/EDV	n	138	58	
		Mean (SD)	411.4 (14.5)	407.7 (14.8)	3.69 [-0.09; 7.47]
		Median	410.5	407.0	
		Min/ Max	371/458	381/450	
	Change from V1b	n	132	54	
		Mean (SD)	3.2 (12.3)	-1.3 (14.5)	4.55 [1.09; 8.02]
		Median	4.0	-2.0	
		Min/ Max	-28/48	-30/43	

Source: Section 15.3, Table 15.3.6.2
N: Number of subjects in specified treatment group
n: Number of subjects with data available
SD: Standard deviation

The ECGs were assessed as being normal or abnormal based on the following criteria: artefact present; abnormal wave from analysis; heart rate < 50 or > 100 bpm; PR > 200 ms: first degree AV block; QRS > 109 ms; QTcB or QTcF > 499 ms.

A summary of ECG parameters' interpretation is presented in Table 80 below. In the Test group, abnormal ECG findings at Visit 1b and Visit 5 or EDV were reported for eight subjects (5.3% and 5.8%, respectively).

In the Reference group, abnormal ECG findings were reported for two subjects (3.6%) at Visit 1b, whereas at Visit 5 (or EDV) ECGs of all subjects were assessed as normal. The difference between the groups with regard to distribution of subjects with normal and

abnormal ECG assessments was not statistically significant (V1b: p = 1.0000; Visit 5/EDV: p = 0.1079, Fisher’s exact test).

Table 80: Interpretation of ECG Parameters (SS)

Interpretation	Test (N=858)		Reference (N=332)		p-value [a]	
	Visit 1b n (%)	Visit 5/EDV n (%)	Visit 1b n (%)	Visit 5/EDV n (%)	Visit 1b	Visit 5/EDV
n	152 (100)	138 (100)	56 (100)	58 (100)		
Normal	144 (94.7)	130 (94.2)	54 (96.4)	58 (100)	1.0000	0.1079
Abnormal	8 (5.3)	8 (5.8)	2 (3.6)	0 (0.0)		

Source: Section 15.3, Table 15.3.6.2
N: Number of subjects in specified treatment group n: Number of subjects with data available
%: Percentage based on N
Note: [a] Fisher’s exact testThe analysis of categorical QTcF indices demonstrated that in the Test group one subject at V1b and at V5/EDV had QTcF >450 ms and one subject had a QTcF increase of 30 ms-60 ms from baseline at V5/EDV, whereas none of the subjects met these criteria in the Reference group. There were no subjects in either treatment group with QTcF >470 ms or an increase from baseline of the QTcF > 60 ms. None of the Test group subjects at V5 had a heart rate <50 bpm while one subject met this criterion in the Reference group, see Section 15.3, Table 15.3.6.2.

The finding of more “abnormal” ECGs at Visit 5 in the Test group is likely due to chance and the ~2.4 fold larger size of the Test group. Only three Test group subjects at Visit 5 had new isolated abnormal ECG determinations.

None of the abnormal ECG findings was assessed as being clinically significant (Section 15.3, Table 15.3.6.1).

As concerns TEAEs related to ECG, tachycardia was reported for two Test group subjects (see Section 12.6.1.3).

12.7 Tolerability

The tolerability assessments were based on the vaginal bleeding data, as reported in subjects’ e-diaries on the daily basis. All diary records with less than 84 days were excluded from the analysis by the reference period. Cycles without consecutively missing entries and with less than five non-consecutive missing entries only were used for the analysis.

Imputation was applied for single missing entries only. The maximum of the bleeding intensity recorded on the day before or the day after the missing entries were imputed.

All diary records with less than 84 days were excluded from the analysis by reference period.

12.7.1 Number of subjects with bleeding or spotting

Individual data on bleeding pattern by cycle are provided in Appendix 16.2, Listing 16.3.1.1.

Numbers of subjects with bleeding or spotting by treatment day are presented in Figure 2 below. The differences between the bleeding patterns can be explained by the different regimen of the two tested contraceptives: Desogestrel was used in a regimen of 28 active pills, whereas drospirenone was used in a regimen of 24 verum tablets followed by four placebo tablets. Therefore scheduled bleedings were present only in the Test group using drospirenone.

Figure 2: Number of Subjects with Bleeding orSpotting by Treatment Day (FAS)

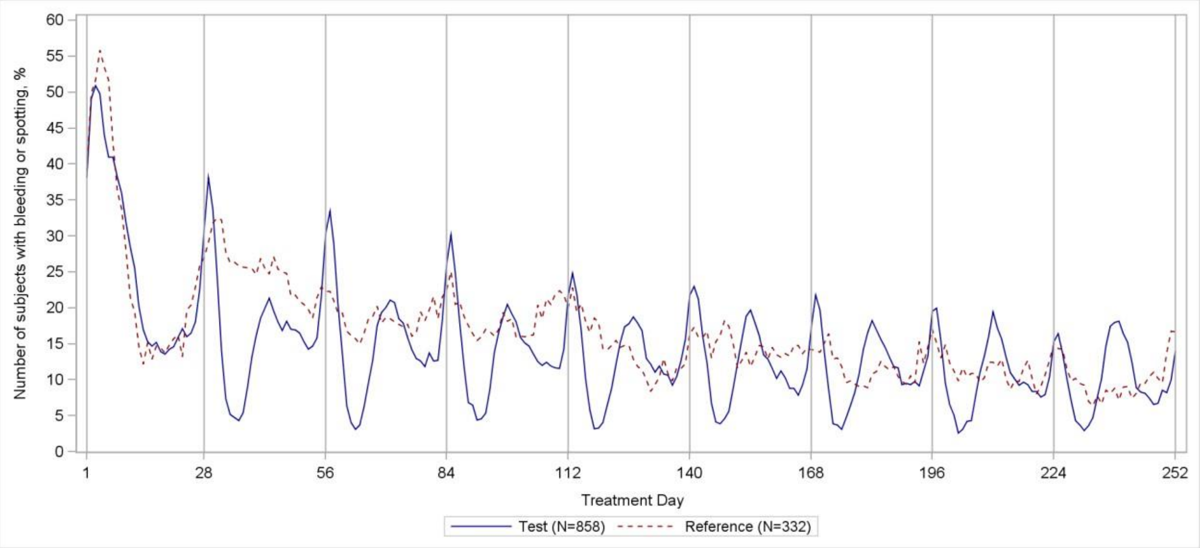

Source: Section 15.4, Figure 15.4.1

Numbers of subjects with bleeding/spotting in each treatment cycle and period are presented in Table 81, and those by the reference period are depicted in Figure 3. During the entire treatment period, excluding Cycle 1, the proportions of subjects with bleeding/spotting were comparable between the groups (83.9% of the Test and 87.9% of the Reference group subjects). During the first reference period (Cycles 2-4) the proportion of subjects with bleeding/spotting was lower in the Test than in the Reference group (79.9% vs. 86.5% of subjects), and the difference was statistically significant (p = 0.0324, chi square test). During the second (Cycles 5-7) and the third reference periods (Cycles 7-9) the proportions of subjects in the Test group were higher than in the Reference group (74.0% vs. 67.5% and 73.3% vs. 67.9%, respectively), with no statistically significant differences between the groups.

Figure 3: Number of Subjects with Bleeding/Spotting by Reference Period (FAS)

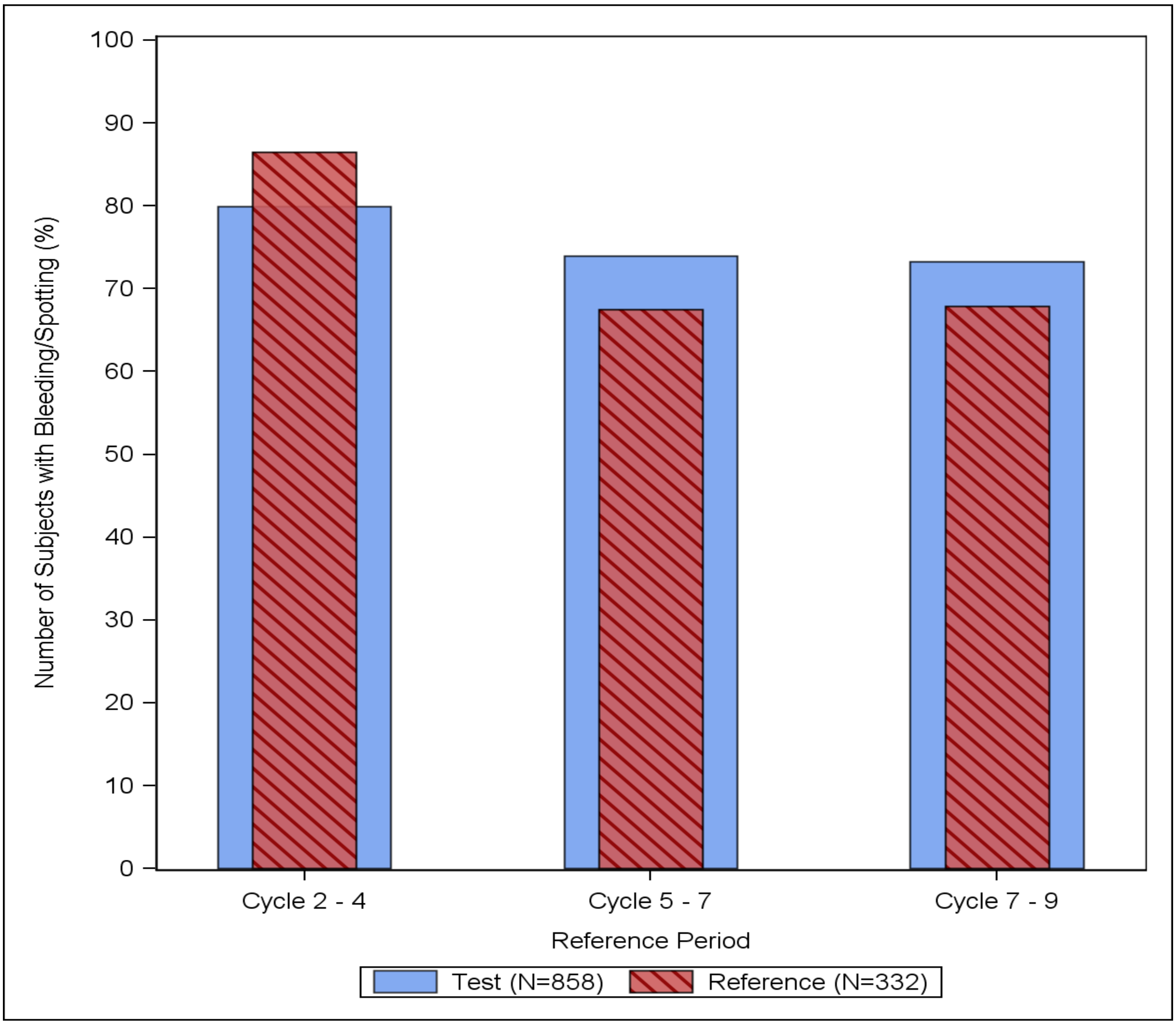

Source: Section 15.4, Figure 15.4.2

Table 81: Number of Subjects with Bleeding or Spotting by Treatment Cycle and Period (FAS)

Cycle	Test n/m (%)	Reference n/m (%)	Difference (95% CI)	Chi square test p value
Cycle 1	692/765 (90.5)	284/305 (93.1)	-2.66 (-6.18 ; 0.87)	0.1657
Cycle 2	482/692 (69.7)	211/285 (74.0)	-4.38 (-10.5 ; 1.75)	0.1704
Cycle 3	429/637 (67.3)	160/251 (63.7)	3.60 (-3.37 ; 10.58)	0.3064
Cycle 4	390/606 (64.4)	161/244 (66.0)	-1.63 (-8.69 ; 5.44)	0.6531
Cycle 5	351/566 (62.0)	118/219 (53.9)	8.13 (0.41 ; 15.85)	0.0372
Cycle 6	305/530 (57.5)	110/199 (55.3)	2.27 (-5.82 ; 10.36)	0.5812
Cycle 7	292/503 (58.1)	91/185 (49.2)	8.86 (0.47 ; 17.26)	0.0380
Cycle 8	264/468 (56.4)	87/178 (48.9)	7.53 (-1.07 ; 16.14)	0.0859
Cycle 9	249/442 (56.3)	73/161 (45.3)	10.99 (2.02 ; 19.97)	0.0167
Cycles 2 - 4	421/527 (79.9)	192/222 (86.5)	-6.60 (-12.3 ; -0.95)	0.0324
Cycles 5 - 7	313/423 (74.0)	106/157 (67.5)	6.48 (-1.95 ; 14.91)	0.1216
Cycles 7 - 9	274/374 (73.3)	93/137 (67.9)	5.38 (-3.64 ; 14.39)	0.2312
Cycles 2 - 6	346/422 (82.0)	152/172 (88.4)	-6.38 (-12.4 ; -0.35)	0.0553
Cycles 2 - 9	256/305 (83.9)	102/116 (87.9)	-4.00 (-11.2 ; 3.22)	0.3044

Source: Section 15.4, Table 15.4.1.1

n: Number of subjects with data available
m: Number of subjects in respective cycle
%: Percentage based on m
CI: Confidence interval

Bleeding

The incidence of bleeding by treatment cycle and period is presented in Table 82.

Table 82: Number of Subjects with Bleeding by Treatment Cycle and Period (FAS)

Cycle	Test n/m (%)	Reference n/m (%)	Difference (95% CI)	Chi square test p value
Cycle 1	571/765 (74.6)	243/305 (79.7)	-5.03 (-10.5 ; 0.44)	0.0816
Cycle 2	297/692 (42.9)	137/285 (48.1)	-5.15 (-12.0 ; 1.72)	0.1408
Cycle 3	237/634 (37.4)	86/251 (34.3)	3.10 (-3.87 ; 10.07)	0.3876
Cycle 4	213/606 (35.1)	86/244 (35.2)	-0.10 (-7.20 ; 7.00)	0.9785
Cycle 5	199/566 (35.2)	59/219 (26.9)	8.22 (1.15 ; 15.29)	0.0279
Cycle 6	176/530 (33.2)	70/199 (35.2)	-1.97 (-9.72 ; 5.78)	0.6166
Cycle 7	155/503 (30.8)	50/185 (27.0)	3.79 (-3.78 ; 11.35)	0.3354
Cycle 8	128/468 (27.4)	44/178 (24.7)	2.69 (-4.88 ; 10.15)	0.4990
Cycle 9	139/442 (31.4)	30/161 (18.6)	12.81 (5.40 ; 20.22)	0.0019
Cycles 2 - 4	300/527 (56.9)	128/222 (57.7)	-0.73 (-8.49 ; 7.02)	0.8534
Cycles 5 - 7	200/423 (47.3)	69/157 (43.9)	3.33 (-5.77 ; 12.44)	0.4746
Cycles 7 - 9	171/374 (45.7)	61/137 (44.5)	1.20 (-8.54 ; 10.93)	0.8099
Cycles 2 - 6	263/422 (62.3)	105/172 (61.0)	1.28 (-7.35 ; 9.91)	0.7715
Cycles 2 - 9	198/305 (64.9)	76/116 (65.5)	-0.60 (-10.8 ; 9.57)	0.9083

Source: Section 15.4, Table 15.4.1.2
n: Number of subjects with data available m: Number of subjects in respective cycle
%: Percentage based on m CI: Confidence interval

During Cycles 2-9, a comparable proportion of subjects: 64.9% of the Test and 65.5% of the Reference group experienced bleeding.

Spotting

The numbers and proportions of subjects with spotting by treatment cycle and period are provided in Table 83 below. During Cycles 2-9, 83.3% of the Test and 87.1% of the Reference group subjects experienced spotting, with no statistically significant difference between the groups. The proportions of subjects who spotted tended to decrease over time in both groups. From Cycle 2 to Cycle 9 the proportion of subjects in the Test group decreased from 66.3% to 52.3%, and in the Reference group from 71.9% to 43.5%.

During the first reference period, the proportion of subjects who spotted in the Test group was statistically significantly lower than in the Reference group: 78.2% vs. 86.5% (p = 0.0086, chi square test). No significant differences between the groups were observed during the two subsequent reference periods.

Table 83: Number of Subjects with Spotting by Treatment Cycle and Period (FAS)

Cycle	Test n/m (%)	Reference n/m (%)	Difference (95% CI)	Chi square test p value
Cycle 1	659/765 (86.1)	269/305 (88.2)	-2.05 (-6.42 ; 2.32)	0.3716
Cycle 2	459/692 (66.3)	205/285 (71.9)	-5.60 (-11.9 ; 0.69)	0.0882
Cycle 3	411/637 (64.5)	154/251 (61.4)	3.17 (-3.91 ; 10.24)	0.3771
Cycle 4	377/606 (62.2)	157/244 (64.3)	-2.13 (-9.28 ; 5.01)	0.5605
Cycle 5	336/566 (59.4)	109/219 (49.8)	9.59 (1.83 ; 17.35)	0.0150
Cycle 6	293/530 (55.3)	102/199 (51.3)	4.03 (-4.11 ; 12.16)	0.3310
Cycle 7	275/503 (54.7)	90/185 (48.6)	6.02 (-2.39 ; 14.44)	0.1604
Cycle 8	245/468 (52.4)	86/178 (48.3)	4.04 (-4.59 ; 12.66)	0.3592
Cycle 9	231/442 (52.3)	70/161 (43.5)	8.78 (-0.18 ; 17.75)	0.0563
Cycles 2 - 4	412/527 (78.2)	192/222 (86.5)	-8.31 (-14.0 ; -2.59)	0.0086
Cycles 5 - 7	304/423 (71.9)	104/157 (66.2)	5.63 (-2.92 ; 14.17)	0.1875
Cycles 7 - 9	268/374 (71.7)	92/137 (67.2)	4.50 (-4.59 ; 13.60)	0.3229
Cycles 2 - 6	342/422 (81.0)	151/172 (87.8)	-6.75 (-12.9 ; -0.59)	0.0471
Cycles 2 - 9	254/305 (83.3)	101/116 (87.1)	-3.79 (-11.2 ; 3.61)	0.3392

Source: Section 15.4, Table 15.4.1.3
n: Number of subjects with data available
m: Number of subjects in respective cycle
%: Percentage based on m
CI: Confidence interval

12.7.2 Number of subjects with unscheduled bleeding or spotting

Unscheduled bleeding or spotting day was defined as any bleeding/spotting that occurred while taking active hormones (Days 2 – 23), except days which were classified as scheduled bleeding days. Due to the different treatment regimen, this definition is applicable only to the Test group subjects. All bleeding which occurred in the Reference group was classified as unscheduled. The numbers of subjects with unscheduled bleeding and spotting during each cycle and defined treatment periods are provided in Table 84 below.

Table 84: Number of Subjects with Unscheduled Bleeding or Spotting by Treatment Cycle and Period (FAS)

Cycle	Test n/m (%)	Reference n/m (%)	Difference (%) (95% CI)	Chi square test p value
Cycle 1	375/765 (49.0)	177/305 (58.0)	-9.01 (-15.59 ; -2.44)	0.0077
Cycle 2	356/692 (51.4)	211/285 (74.0)	-22.59 (-28.90 ; -16.28)	<0.0001
Cycle 3	319/637 (50.1)	160/251 (63.7)	-13.67 (-20.77 ; -6.56)	0.0002
Cycle 4	291/606 (48.0)	161/244 (66.0)	-17.96 (-25.12 ; -10.81)	<0.0001
Cycle 5	252/566 (44.5)	118/219 (53.9)	-9.36 (-17.13 ; -1.59)	0.0185
Cycle 6	240/530 (45.3)	110/199 (55.3)	-9.99 (-18.10 ; -1.89)	0.0161
Cycle 7	221/503 (43.9)	91/185 (49.2)	-5.25 (-13.66 ; 3.16)	0.2198
Cycle 8	202/468 (43.2)	87/178 (48.9)	-5.71 (-14.32 ; 2.89)	0.1919
Cycle 9	194/442 (43.9)	73/161 (45.3)	-1.45 (-10.42 ; 7.52)	0.7511
Cycles 2 - 4	358/527 (67.9)	192/222 (86.5)	-18.55 (-24.56 ; -12.55)	<0.0001
Cycles 5 - 7	269/423 (63.6)	106/157 (67.5)	-3.92 (-12.56 ; 4.72)	0.3799
Cycles 7 - 9	243/374 (65.0)	93/137 (67.9)	-2.91 (-12.10 ; 6.28)	0.5392
**Cycles 2 - 6**	**308/422 (73.0)**	**152/172 (88.4)**	**-15.39 (-21.78 ; -8.99)**	**<0.0001**
Cycles 2 - 9	243/305 (79.7)	102/116 (87.9)	-8.26 (-15.71 ; -0.81)	0.0490

Source: Section 15.4, Table 15.4.1.7
n: Number of subjects with data available
m: Number of subjects in respective cycle
%: Percentage based on m
CI: Confidence interval

The proportion of subjects with unscheduled bleeding/spotting during Cycles 2-6 was lower in the Test group (308 subjects, 73.0%) than in the Reference group (152 subjects, 88.4%), with a difference (95% CI) of -15.39% (-21.78; -8.99) between the groups. Since the two- sided 95% CI lies entirely to the left of the defined non-inferiority margin of 9%, the Test group is non inferior to the Reference group. Moreover, since the 95% CI not only lies entirely below 9% but also below zero, superiority in terms of statistical significance at the 5% level ($p < 0.05$) was concluded.

During Cycles 2-9, the incidence of unscheduled bleeding/spotting was lower in the Test group compared to the Reference group: 79.7% vs. 87.9%. The difference between the groups was statistically significant ($p = 0.0490$, chi square test).

The highest proportion of subjects with unscheduled bleeding or spotting was observed in Cycle 2: 51.4% of the Test and 74.0% of the Reference group subjects. The incidence of unscheduled bleeding decreased over time in both groups, to 43.9% of the Test and 45.3% of the Reference group subjects in Cycle 9.

The proportions of subjects with unscheduled bleeding and spotting in the Test group were lower than in the Reference group in each cycle and defined period. The tendency to larger, statistically significant differences between the groups was observed in each cycle up to Cycle 7, but the difference was not significant in Cycles 7, 8 and 9. The difference between the groups was also significant during the first reference period and Cycles 2-6 ($p < 0.0001$, both; chi square test). The differences between the groups during the second and the third reference periods were not statistically significant.

Unscheduled bleeding

During Cycles 2-9, 54.8% of the Test group and 65.5% of the Reference group subjects experienced unscheduled bleeding (Table 85). For bleeding taken alone, the same trends were observed as for bleeding and spotting taken together.

Table 85: Number of Subjects with Unscheduled Bleeding by Treatment Cycle and Period (FAS)

Cycle	Test n/m (%)	Reference n/m (%)	Difference (95% CI)	Chi square test p value
Cycle 1	158/765 (20.7)	81/305 (26.6)	-5.90 (-11.6 ; -0.18)	0.0363
Cycle 2	192/692 (27.7)	137/285 (48.1)	-20.3 (-27.0 ; -13.6)	<0.0001
Cycle 3	170/637 (26.7)	86/251 (34.3)	-7.58 (-14.4 ; -0.77)	0.0248
Cycle 4	148/606 (24.4)	86/244 (35.2)	-10.8 (-17.7 ; -3.92)	0.0014
Cycle 5	136/566 (24.0)	59/219 (26.9)	-2.91 (-9.76 ; 3.94)	0.3970
Cycle 6	128/530 (24.2)	70/199 (35.2)	-11.0 (-18.6 ; -3.46)	0.0029
Cycle 7	109/503 (21.7)	50/185 (27.0)	-5.36 (-12.7 ; 1.99)	0.1394
Cycle 8	92/468 (19.7)	44/178 (24.7)	-5.06 (-12.3 ; 2.23)	0.1586
Cycle 9	103/442 (23.3)	30/161 (18.6)	4.67 (-2.52 ; 11.86)	0.2212
Cycles 2 - 4	229/527 (43.5)	128/222 (57.7)	-14.2 (-22.0 ; -6.45)	0.0004
Cycles 5 - 7	153/423 (36.2)	69/157 (43.9)	-7.78 (-16.8 ; 1.23)	0.0868
Cycles 7 - 9	137/374 (36.6)	61/137 (44.5)	-7.89 (-17.5 ; 1.75)	0.1047
Cycles 2 - 6	212/422 (50.2)	105/172 (61.0)	-10.8 (-19.5 ; -2.10)	0.0166
Cycles 2 - 9	167/305 (54.8)	76/116 (65.5)	-10.8 (-21.1 ; -0.47)	0.0458

Source: Section 15.4, Table 15.4.1.8

n: Number of subjects with data available
m: Number of subjects in respective cycle
%: Percentage based on m
CI: Confidence interval

Unscheduled spotting

During Cycles 2-9 unscheduled spotting was more common than unscheduled bleeding and was documented for 78.4% of the Test and 87.1% of the Reference group subjects, see Table 86. The difference between the groups was statistically significant (p = 0.0428, chi square test). The proportions of subjects with spotting were lower in the Test than in the Reference group in each cycle and treatment period. From Cycle 2 to Cycle 9 the proportion of subjects with unscheduled spotting decreased in both treatment groups (Test: 50.0% to 41.2%; Reference: 71.9% to 43.5%)

With respect to the reference periods, the difference between the treatment groups was statistically significant during Cycles 2-4 (66.6% Test vs. 86.5% Reference, p < 0.0001, chi square test) only. From the first to the third reference period the proportion of subjects with unscheduled spotting decreased from 66.6% to 63.1% in the Test and from 86.5% to 67.2% in the Reference group.

Table 86: Number of Subjects with Unscheduled Spotting by Treatment Cycle and Period (FAS)

Cycle	Test n/m (%)	Reference n/m (%)	Difference (95% CI)	Chi square test p value
Cycle 1	363/765 (47.5)	168/305 (55.1)	-7.63 (-14.2 ; -1.02)	0.0242
Cycle 2	346/692 (50.0)	205/285 (71.9)	-21.9 (-28.3 ; -15.5)	<0.0001
Cycle 3	306/637 (48.0)	154/251 (61.4)	-13.3 (-20.5 ; -6.15)	0.0003
Cycle 4	280/606 (46.2)	157/244 (64.3)	-18.1 (-25.3 ; -10.9)	<0.0001
Cycle 5	240/566 (42.4)	109/219 (49.8)	-7.37 (-15.1 ; 0.40)	0.0624
Cycle 6	231/530 (43.6)	102/199 (51.3)	-7.67 (-15.8 ; 0.46)	0.0640
Cycle 7	211/503 (41.9)	90/185 (48.6)	-6.70 (-15.1 ; 1.69)	0.1162
Cycle 8	193/468 (41.2)	86/178 (48.3)	-7.08 (-15.7 ; 1.51)	0.1048
Cycle 9	182/442 (41.2)	70/161 (43.5)	-2.30 (-11.2 ; 6.62)	0.6122
Cycles 2 - 4	351/527 (66.6)	192/222 (86.5)	-19.9 (-25.9 ; -13.8)	<0.0001
Cycles 5 - 7	262/423 (61.9)	104/157 (66.2)	-4.30 (-13.0 ; 4.42)	0.3399
Cycles 7 - 9	236/374 (63.1)	92/137 (67.2)	-4.05 (-13.3 ; 5.21)	0.3974
Cycles 2 - 6	302/422 (71.6)	151/172 (87.8)	-16.2 (-22.7 ; -9.71)	<0.0001
Cycles 2 - 9	239/305 (78.4)	101/116 (87.1)	-8.71 (-16.4 ; -1.05)	0.0428

Source: Section 15.4, Table 15.4.1.9

n: Number of subjects with data available

m: Number of subjects in respective cycle

%: Percentage based on m

CI: Confidence interval

12.7.3 Number of subjects who had no bleeding or spotting

During Cycles 2-9, the proportion of subjects with no bleeding/spotting was comparable between the groups: 16.1% of subjects in the Test and 12.1% of subjects in the Reference group, Table 87.

Table 87: Number of Subjects with no Bleeding or Spotting by Treatment Cycle and Period (FAS)

Cycle	Test n/m (%)	Reference n/m (%)	Difference (95% CI)	Chi square test p value
Cycle 1	73/765 (9.5)	21/305 (6.9)	2.66 (-0.87 ; 6.18)	0.1657
Cycle 2	210/692 (30.3)	74/285 (26.0)	4.38 (-1.75 ; 10.52)	0.1704
Cycle 3	208/637 (32.7)	91/251 (36.3)	-3.60 (-10.6 ; 3.37)	0.3064
Cycle 4	216/606 (35.6)	83/244 (34.0)	1.63 (-5.44 ; 8.69)	0.6531
Cycle 5	215/566 (38.0)	101/219 (46.1)	-8.13 (-15.9 ; -0.41)	0.0372
Cycle 6	225/530 (42.5)	89/199 (44.7)	-2.27 (-10.4 ; 5.82)	0.5812
Cycle 7	211/503 (41.9)	94/185 (50.8)	-8.86 (-17.3 ; -0.47)	0.0380
Cycle 8	204/468 (43.6)	91/178 (51.1)	-7.53 (-16.1 ; 1.07)	0.0859
Cycle 9	193/442 (43.7)	88/161 (54.7)	-11.0 (-20.0 ; -2.02)	0.0167
Cycles 2 - 4	106/527 (20.1)	30/222 (13.5)	6.60 (0.95 ; 12.25)	0.0324
Cycles 5 - 7	110/423 (26.0)	51/157 (32.5)	-6.48 (-14.9 ; 1.95)	0.1216
Cycles 7 - 9	100/374 (26.7)	44/137 (32.1)	-5.38 (-14.4 ; 3.64)	0.2312
Cycles 2 - 6	76/422 (18.0)	20/172 (11.6)	6.38 (0.35 ; 12.41)	0.0553
Cycles 2 - 9	49/305 (16.1)	14/116 (12.1)	4.00 (-3.22 ; 11.22)	0.3044

Source: Section 15.4, Table 15.4.1.10
n: Number of subjects with data available
m: Number of subjects in respective cycle
%: Percentage based on m
CI: Confidence interval

The lowest proportion of subjects who had no bleeding or spotting in both treatment groups was observed in Cycle 1: 9.5% of the Test group and 6.9% of the Reference group subjects. In Cycle 2 the proportion of subjects with no bleeding/spotting increased approximately threefold in both groups. From Cycle 2 to Cycle 9 the proportion of subjects who had no bleeding or spotting increased from 30.3% to 43.7% subjects in the Test and from 26.0% to 54.7% subjects in the Reference group.

As concerns the reference periods, the proportion of subjects who had no bleeding or spotting increased during Cycles 5-7 and remained almost the same during the last 3-cycle period in both groups.

12.7.4 Numbers of subjects by bleeding intensity

The proportions of subjects with slight, moderate and heavy bleeding (Table 88, Table 89 and Table 90) decreased over time in both treatment groups, with no major differences between the groups. Slight and moderate bleeding intensities prevailed during all defined periods. During Cycles 2-9, 54 (17.7%) of the Test and 25 (21.6%) of the Reference group subjects had bleeding of heavy intensity.

Table 88: Number of Subjects with Slight Bleeding by Treatment Period (FAS)

Cycle	Test n/m (%)	Reference n/m (%)	Difference (95% CI)	Chi square test p value
Cycles 2 - 4	239/527 (45.4)	96/222 (43.2)	2.11 (-5.67 ; 9.89)	0.5962
Cycles 5 - 7	167/423 (39.5)	56/157 (35.7)	3.81 (-5.01 ; 12.63)	0.4019
Cycles 7 - 9	147/374 (39.3)	41/137 (29.9)	9.38 (0.25 ; 18.50)	0.0515
Cycles 2 - 6	215/422 (50.9)	86/172 (50.0)	0.95 (-7.92 ; 9.81)	0.8340
Cycles 2 - 9	173/305 (56.7)	61/116 (52.6)	4.14 (-6.52 ; 14.79)	0.4455

Source: Section 15.4, Table 15.4.1.4
n: Number of subjects with data available %: Percentage based on m
m: Number of subjects in respective cycle CI: Confidence interval

Table 89: Number of Subjects with Moderate Bleeding by Treatment Period (FAS)

Cycle	Test n/m (%)	Reference n/m (%)	Difference (95% CI)	Chi square test p value
Cycles 2 - 4	223/527 (42.3)	95/222 (42.8)	-0.48 (-8.23 ; 7.28)	0.9038
Cycles 5 - 7	147/423 (34.8)	49/157 (31.2)	3.54 (-5.01 ; 12.09)	0.4230
Cycles 7 - 9	119/374 (31.8)	49/137 (35.8)	-3.95 (-13.3 ; 5.36)	0.4000
Cycles 2 - 6	205/422 (48.6)	81/172 (47.1)	1.49 (-7.37 ; 10.34)	0.7425
Cycles 2 - 9	153/305 (50.2)	62/116 (53.4)	-3.28 (-14.0 ; 7.39)	0.5470

Source: Section 15.4, Table 15.4.1.5
n: Number of subjects with data available %: Percentage based on m
m: Number of subjects in respective cycle CI: Confidence interval

Table 90: Number of Subjects with Heavy Bleeding by Treatment Period (FAS)

Cycle	Test n/m (%)	Reference n/m (%)	Difference (95% CI)	Chi square test p value
Cycles 2 - 4	62/527 (11.8)	27/222 (12.2)	-0.40 (-5.50 ; 4.71)	0.8780
Cycles 5 - 7	38/423 (9.0)	12/157 (7.6)	1.34 (-3.63 ; 6.31)	0.6094
Cycles 7 - 9	34/374 (9.1)	15/137 (10.9)	-1.86 (-7.84 ; 4.13)	0.5275
Cycles 2 - 6	65/422 (15.4)	22/172 (12.8)	2.61 (-3.45 ; 8.68)	0.4141
Cycles 2 - 9	54/305 (17.7)	25/116 (21.6)	-3.85 (-12.5 ; 4.78)	0.3664

Source: Section 15.4, Table 15.4.1.6
n: Number of subjects with data available %: Percentage based on m
m: Number of subjects in respective cycle CI: Confidence interval

The numbers of subjects by bleeding intensity in each treatment cycle are provided in Section 15.4, Tables 15.4.1.4, 15.4.1.5 and 15.4.1.6.

12.7.5 Subjects with infrequent, frequent and prolonged bleeding

The infrequent, frequent and prolonged bleedings were defined according to the WHO Belsey system of bleeding.[11] The system establishes criteria for defining clinically important bleeding patterns during a 90-day reference period. Infrequent bleeding was defined in the SAP as 1 – 2 bleeding/spotting episodes during the reference period. During each reference period the proportions of subjects with infrequent bleeding were lower in the Test group compared to the Reference group, see Table 91. A statistically significant difference between the groups was observed only during Cycles 7-9: 57.0% of the Test vs. 73.0% of the Reference group subjects (p = 0.0010, chi square test).

Table 91: Number of Subjects with Infrequent Bleeding per Reference Period (FAS)

Cycle	Test n/m (%)	Reference n/m (%)	Difference (95% CI)	Chi square test p-value
Cycles 2 - 4	246/527 (46.7)	105/222 (47.3)	-0.62 (-8.45 ; 7.21)	0.8770
Cycles 5 - 7	243/423 (57.4)	102/157 (65.0)	-7.52 (-16.3 ; 1.30)	0.1011
Cycles 7 - 9	213/374 (57.0)	100/137 (73.0)	-16.0 (-25.0 ; -7.07)	0.0010

Source: Section 15.4, Table 15.4.1.11
n: Number of subjects with data available %: Percentage based on m
m: Number of subjects in respective cycle CI: Confidence interval

Frequent bleeding was defined as 6 or more bleeding/spotting episodes during the reference period. The percentage of subjects with frequent bleeding gradually decreased over time from 9.1% to 5.3% in the Test and from 7.2% to 4.4% in the Reference group and was comparable between the treatment groups in each reference period (Table 92).

Table 92: Number of Subjects with Frequent Bleeding per Reference Period (FAS)

Cycle	Test n/m (%)	Reference n/m (%)	Difference (95% CI)	Chi square test p value
Cycles 2 - 4	48/527 (9.1)	16/222 (7.2)	1.90 (-2.30 ; 6.10)	0.3954
Cycles 5 - 7	28/423 (6.6)	8/157 (5.1)	1.52 (-2.65 ; 5.70)	0.4992
Cycles 7 - 9	20/374 (5.3)	6/137 (4.4)	0.97 (-3.15 ; 5.08)	0.6591

Source: Section 15.4, Table 15.4.1.12
n: Number of subjects with data available %: Percentage based on m
m: Number of subjects in respective cycle CI: Confidence interval

Prolonged bleeding was defined as a bleeding/spotting episode with a length of more than 14 days. The proportions of subjects with prolonged bleeding decreased over time from 12.1% to 2.9% of the Test and from 16.7% to 10.9% of the Reference group subjects, see Table 93. The percentage of subjects who experienced prolonged bleeding in each reference period was lower in the Test than in the Reference group, with statistically significant differences between the groups in the second ($p = 0.0172$, chi square test) and in the third ($p = 0.0003$, chi square test) reference period.

Table 93: Number of Subjects with Prolonged Bleeding per Reference Period (FAS)

Cycle	Test n/m (%)	Reference n/m (%)	Difference (95% CI)	Chi square test p-value
Cycles 2 - 4	64/527 (12.1)	37/222 (16.7)	-4.52 (-10.2 ; 1.12)	0.0980
Cycles 5 - 7	26/423 (6.1)	19/157 (12.1)	-5.96 (-11.5 ; -0.36)	0.0172
Cycles 7 - 9	11/374 (2.9)	15/137 (10.9)	-8.01 (-13.5 ; -2.51)	0.0003

Source: Section 15.4, Table 15.4.1.13
n: Number of subjects with data available %: Percentage based on m
m: Number of subjects in respective cycle CI: Confidence interval

12.7.6 Number of bleeding or spotting days

The mean number of bleeding or spotting days by cycle is depicted in Figure 4 and presented in Section 15.4, Table 15.4.1.14.

Figure 4: Mean Number of Bleeding or Spotting Days per Cycle

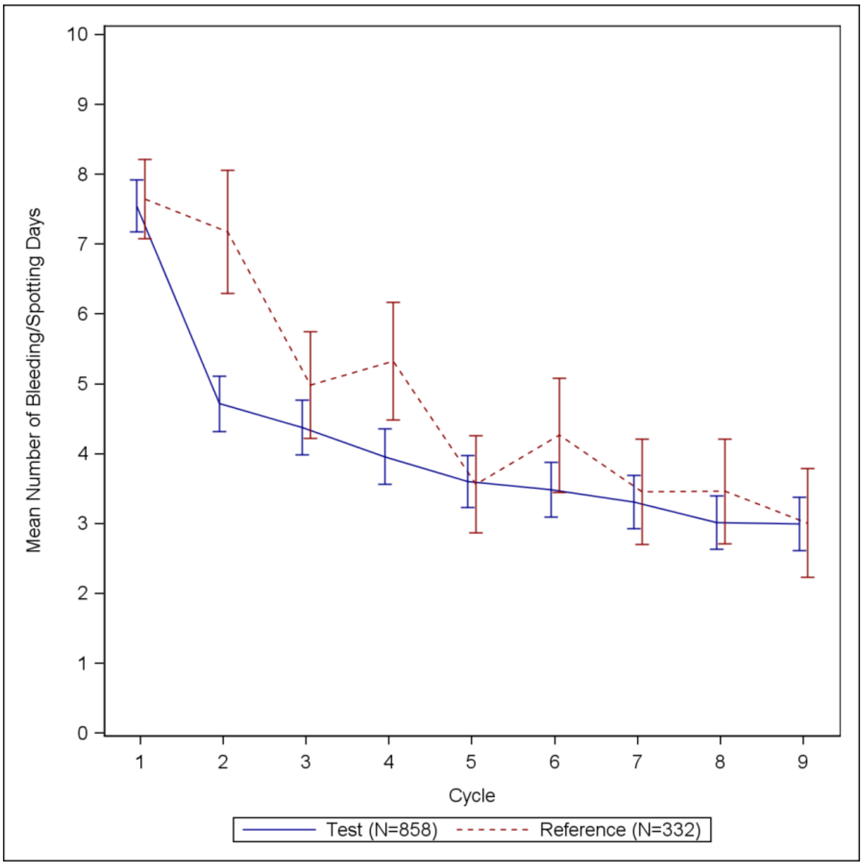

Source: Section 15.4, Table 15.4.5.1

The summary of bleeding or spotting days by reference period is provided in Table 94.

A trend towards less bleeding/spotting days was observed over time. The mean (SD) number of bleeding or spotting days decreased from 13.1 (13.05) days during Cycles 2-4 to 9.7 (10.39) days during Cycles 7-9 in the Test and from 16.9 (16.93) to 10.8 (13.34) days in the Reference group.The median number of bleeding or spotting days decreased from 10.0 to 6.0 days in the Test and from 12.0 to 7.0 days in the Reference group, respectively.

The number of bleeding/spotting days was lower in the Test than in the Reference group at all defined treatment periods. However, the difference between the mean (SD) number of bleeding or spotting days was statistically significant only during the first reference period:
13.1 (13.05) days in the Test vs. 16.9 (16.93) days in the Reference group (p=0.0149, Wilcoxon-rank-sum-test).

Table 94: Number of Days with Bleeding and/or Spotting by Treatment Period (FAS)

Cycle		Test (N=858)	Reference (N=332)	Total (N=1190)	Wilcoxon-rank-sum-test p value
Cycles 2 - 4	n	527	222	749	
	Mean (SD)	13.1 (13.05)	16.9 (16.93)	14.2 (14.40)	0.0149
	Median	10.0	12.0	10.0	
	Min/ Max	0/66	0/79	0/79	
Cycles 5 - 7	n	423	157	580	
	Mean (SD)	10.2 (11.13)	10.6 (12.69)	10.3 (11.56)	0.6868
	Median	6.0	7.0	6.0	
	Min/ Max	0/67	0/61	0/67	
Cycles 7 - 9	n	374	137	511	
	Mean (SD)	9.7 (10.39)	10.8 (13.34)	10.0 (11.26)	0.9659
	Median	6.0	7.0	6.0	
	Min/ Max	0/60	0/83	0/83	
Cycles 2 - 6	n	422	172	594	
	Mean (SD)	19.1 (18.77)	23.7 (24.69)	20.5 (20.74)	0.0894
	Median	14.0	17.0	15.5	
	Min/ Max	0/100	0/134	0/134	
Cycles 2 - 9	n	305	116	421	
	Mean (SD)	29.4 (27.84)	34.7 (33.73)	30.9 (29.63)	0.2557
	Median	21.0	26.0	22.0	
	Min/ Max	0/109	0/156	0/156	

Source: Section 15.4, Table 15.4.1.14
N: Number of subjects in specified treatment group
n: Number of subjects with data available
SD: Number of subjects in respective cycle

Number of bleeding days

During Cycles 2-9 the mean (SD) number of bleeding days was 9.1 (13.35) days in the Test and 12.3 (20.49) days in the Reference group, with no statistically significant difference observed between the groups (Table 95). The median number of bleeding days was 3.0 days in both groups, ranging from 0 to 75 days in the Test and from 0 to 128 days in the Reference group.

The mean number of bleeding days during all defined periods was lower in the Test group as compared to the Reference group with no statistically significant difference between the groups.

Table 95: Number of Days with Bleeding by Treatment Period (FAS)

Cycle		Test (N=858)	Reference (N=332)	Total (N=1190)	Wilcoxon-rank-sum-test p-value
Cycles 2 - 4	n	527	222	749	
	Mean (SD)	4.2 (6.14)	5.5 (9.13)	4.6 (7.18)	0.4251
	Median	1.0	1.0	1.0	
	Min/ Max	0/37	0/75	0/75	
Cycles 5 - 7	n	423	157	580	
	Mean (SD)	3.1 (5.18)	3.9 (8.18)	3.3 (6.14)	0.7517
	Median	0.0	0.0	0.0	
	Min/ Max	0/34	0/53	0/53	
Cycles 7 - 9	n	374	137	511	
	Mean (SD)	3.0 (5.11)	3.9 (7.43)	3.3 (5.83)	0.9593
	Median	0.0	0.0	0.0	
	Min/ Max	0/39	0/49	0/49	
Cycles 2 - 6	n	422	172	594	
	Mean (SD)	6.3 (9.02)	8.2 (14.29)	6.8 (10.83)	0.6361
	Median	2.0	2.0	2.0	
	Min/ Max	0/54	0/127	0/127	
Cycles 2 - 9	n	305	116	421	
	Mean (SD)	9.1 (13.35)	12.3 (20.49)	10.0 (15.68)	0.5149
	Median	3.0	3.0	3.0	
	Min/ Max	0/75	0/128	0/128	

Source: Section 15.4, Table 15.4.1.16
N: Number of subjects in specified treatment group
n: Number of subjects with data available
SD: Number of subjects in respective cycle

Number of spotting days

During Cycles 2-9 the mean (SD) number of spotting days was slightly lower in the Test than in the Reference group: 20.3 (20.20) days vs. 22.4 (21.54) days, see Table 96. The median number of spotting days was 14.0 days in the Test and 15.0 days in the Reference group. The mean (SD) number of spotting days in the Test group decreased from 8.9 (9.82) days during Cycles 2-4 to 6.6 (7.67) days during Cycles 7-9. In the Reference group it decreased from 11.4 (11.88) to 7.0 (8.61) days, respectively. The statistically significant difference between the groups was observed only during the first reference period (p = 0.0065, Wilcoxon-rank-sum-test).

Table 96: Number of Days with Spotting by Treatment Period (FAS)

Cycle		Test (N=858)	Reference (N=332)	Total (N=1190)	Wilcoxon-rank-sum-test p-value
Cycles 2 - 4	n	527	222	749	
	Mean (SD)	8.9 (9.82)	11.4 (11.88)	9.7 (10.53)	0.0065
	Median	6.0	6.0	6.0	
	Min/ Max	0/54	0/58	0/58	
Cycles 5 - 7	n	423	157	580	
	Mean (SD)	7.1 (8.17)	6.7 (8.38)	7.0 (8.22)	0.3073
	Median	5.0	3.0	4.0	
	Min/ Max	0/67	0/41	0/67	
Cycles 7 - 9	n	374	137	511	
	Mean (SD)	6.6 (7.67)	7.0 (8.61)	6.7 (7.92)	0.7585
	Median	4.5	4.0	4.0	
	Min/ Max	0/59	0/40	0/59	
Cycles 2 - 6	n	422	172	594	
	Mean (SD)	12.9 (13.74)	15.6 (16.49)	13.7 (14.62)	0.0847
	Median	8.0	8.0	8.0	
	Min/ Max	0/89	0/85	0/89	
Cycles 2 - 9	n	305	116	421	
	Mean (SD)	20.3 (20.20)	22.4 (21.54)	20.9 (20.57)	0.3585
	Median	14.0	15.0	14.0	
	Min/ Max	0/108	0/86	0/108	

Source: Section 15.4, Table 15.4.1.15
N: Number of subjects in specified treatment group
n: Number of subjects with data available
SD: Number of subjects in respective cycle

12.7.7 Number of unscheduled bleeding or spotting days

The mean number of days with unscheduled bleeding and spotting in each cycle was lower in the Test group, compared to the Reference group and the differences in the mean number of days in each cycle, except Cycle 7 and Cycle 9, were statistically significant with p values < 0.05 (Wilcoxon-rank-sum-test), see Table 97. The number of unscheduled bleeding and spotting days decreased over time in both treatment groups.

Table 97: Number of Days with Unscheduled Bleeding and/or Spotting by Treatment Cycle (FAS)

Cycle		Test (N=858)	Reference (N=332)	Total (N=1190)	Wilcoxon-rank-sum-test p value
Cycle 1	n	765	305	1070	
	Mean (SD)	2.9 (4.33)	3.1 (4.05)	2.9 (4.25)	0.0417
	Median	0.0	1.0	1.0	
	Min/ Max	0/19	0/20	0/20	
Cycle 2	n	692	285	977	
	Mean (SD)	3.4 (4.75)	7.2 (7.54)	4.5 (5.96)	<0.0001
	Median	1.0	5.0	2.0	
	Min/ Max	0/27	0/28	0/28	
Cycle 3	n	637	251	888	
	Mean (SD)	3.2 (4.58)	5.0 (6.13)	3.7 (5.13)	<0.0001
	Median	1.0	3.0	1.0	
	Min/ Max	0/28	0/28	0/28	
Cycle 4	n	606	244	850	
	Mean (SD)	2.9 (4.47)	5.3 (6.68)	3.6 (5.31)	<0.0001
	Median	0.0	3.0	1.0	
	Min/ Max	0/28	0/28	0/28	
Cycle 5	n	566	219	785	
	Mean (SD)	2.6 (4.06)	3.6 (5.22)	2.9 (4.43)	0.0194
	Median	0.0	1.0	0.0	
	Min/ Max	0/24	0/28	0/28	
Cycle 6	n	530	199	729	
	Mean (SD)	2.6 (3.98)	4.3 (5.88)	3.0 (4.63)	0.0011
	Median	0.0	1.0	0.0	
	Min/ Max	0/28	0/27	0/28	
Cycle 7	n	503	185	688	
	Mean (SD)	2.5 (3.91)	3.5 (5.21)	2.7 (4.31)	0.0629
	Median	0.0	0.0	0.0	
	Min/ Max	0/28	0/28	0/28	
Cycle 8	n	468	178	646	
	Mean (SD)	2.3 (3.65)	3.5 (5.07)	2.6 (4.12)	0.0350
	Median	0.0	0.0	0.0	
	Min/ Max	0/28	0/27	0/28	
Cycle 9	n	442	161	603	
	Mean (SD)	2.3 (3.62)	3.0 (5.01)	2.5 (4.05)	0.4236
	Median	0.0	0.0	0.0	
	Min/ Max	0/26	0/28	0/28	

Source: Section 15.4, Table 15.4.1.20
N: Number of subjects in specified treatment group
n: Number of subjects in with data available
SD: Standard deviation

The same tendencies were also observed for each reference period and for Cycles 2 to 6 and Cycles 2 to 9, see Figure 5. The differences between the groups in each defined period were statistically significant (Table 98). During Cycles 2 to 9, the mean (SD) and median number of unscheduled bleeding and spotting days in the Test group was 21.5 (22.86) and 14.0 days, respectively compared to 34.7 (33.73) and 26.0 days in the Reference group.

The mean (SD) number of unscheduled bleeding or spotting days decreased from 9.6 (11.58) days during Cycles 2-4 to 7.2 (8.85) days during Cycles 7-9 in the Test group and from 16.9 (16.93) to 10.8 (13.34) days in the Reference group.

Figure 5: Mean Number of Unscheduled Bleeding or Spotting Days per Reference Period (FAS)

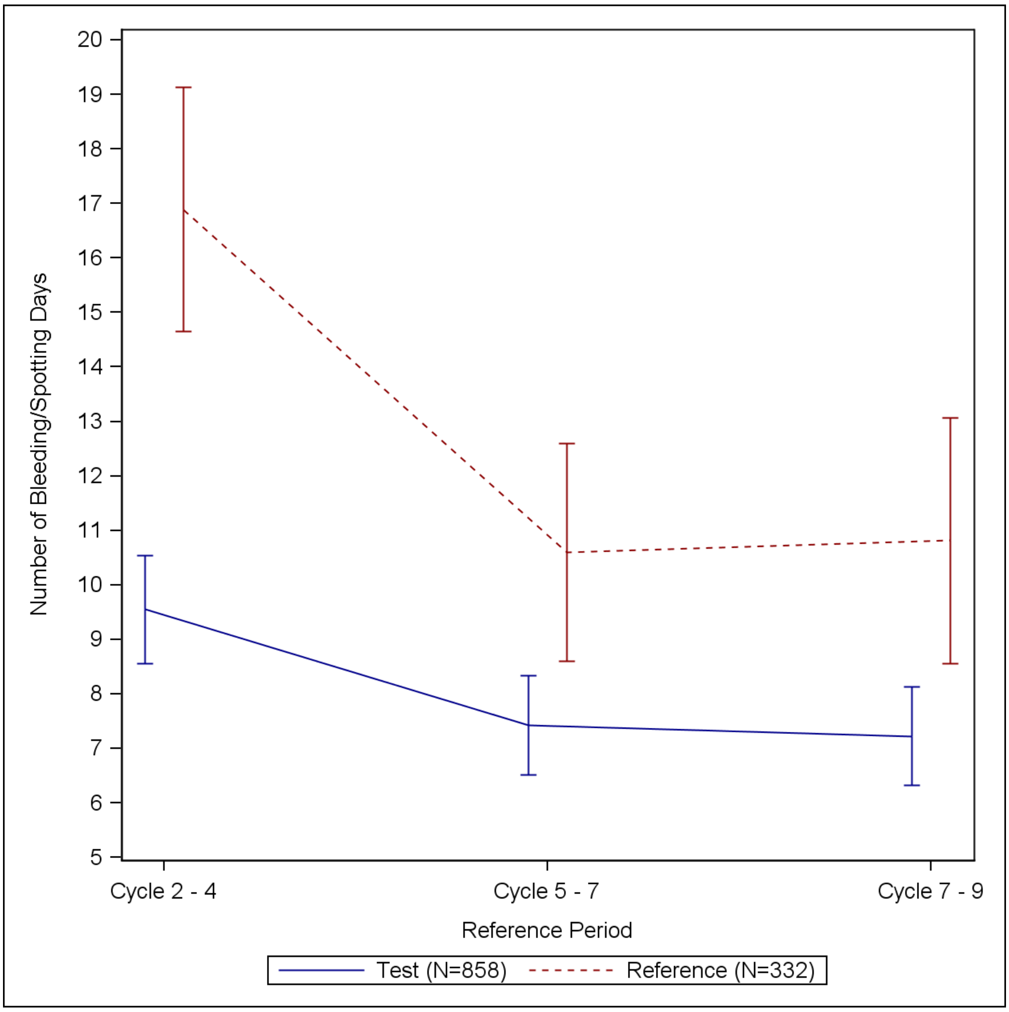

Source: Section 15.4, Figure 15.4.6.2

Table 98: Number of Days with Unscheduled Bleeding and/or Spotting by Treatment Period (FAS)

Cycle		Test (N=858)	Reference (N=332)	Total (N=1190)	Wilcoxon-rank-sum-test p value
Cycles 2 - 4	n	527	222	749	
	Mean (SD)	9.6 (11.58)	16.9 (16.93)	11.7 (13.80)	<0.0001
	Median	5.0	12.0	7.0	
	Min/ Max	0/66	0/79	0/79	
Cycles 5 - 7	n	423	157	580	
	Mean (SD)	7.4 (9.53)	10.6 (12.69)	8.3 (10.56)	0.0232
	Median	4.0	7.0	4.0	
	Min/ Max	0/67	0/61	0/67	
Cycles 7 - 9	n	374	137	511	
	Mean (SD)	7.2 (8.85)	10.8 (13.34)	8.2 (10.35)	0.0277
	Median	4.0	7.0	4.0	
	Min/ Max	0/51	0/83	0/83	
Cycles 2 - 6	n	422	172	594	
	Mean (SD)	13.7 (15.98)	23.7 (24.69)	16.6 (19.44)	<0.0001
	Median	7.0	17.0	9.5	
	Min/ Max	0/89	0/134	0/134	
Cycles 2 - 9	n	305	116	421	
	Mean (SD)	21.5 (22.86)	34.7 (33.73)	25.1 (26.92)	0.0003
	Median	14.0	26.0	16.0	
	Min/ Max	0/95	0/156	0/156	

Source: Section 15.4, Table 15.4.1.20
N: Number of subjects in specified treatment group
n: Number of subjects in with data available
SD: Standard deviation

Number of days with unscheduled bleeding

The numbers of unscheduled bleeding days by treatment period are provided in Table 99. Numbers of unscheduled bleeding days by cycle are presented in Section 15.4, Table 15.4.1.22.

The number of unscheduled bleeding days was low in both groups over the entire treatment period. The number of unscheduled bleeding days was lower in the Test group than in the Reference group at each defined treatment period, with statistically significant differences during Cycles 2-9, Cycles 2-4 and Cycles 2-6. During Cycles 2-9 the Test group subjects had a mean (SD) number of 6.5 (10.51) unscheduled bleeding days versus 12.3 (20.49) days in the Reference group, and the difference between the groups was statistically significant (p = 0.0089, Wilcoxon-rank-sum test). The medians of the groups were 1.0 day in the Test and 3.0 days in the Reference group.

Table 99: Number of Days with Unscheduled Bleeding by Treatment Period (FAS)

Cycle		Test (N=858)	Reference (N=332)	Total (N=1190)	Wilcoxon-rank-sum-test p-value
Cycle 2 - 4	n	527	222	749	
	Mean (SD)	2.9 (5.02)	5.5 (9.13)	3.6 (6.62)	<0.0001
	Median	0.0	1.0	0.0	
	Min/ Max	0/33	0/75	0/75	
Cycle 5 - 7	n	423	157	580	
	Mean (SD)	2.2 (4.27)	3.9 (8.18)	2.7 (5.64)	0.0506
	Median	0.0	0.0	0.0	
	Min/ Max	0/26	0/53	0/53	
Cycle 7 - 9	n	374	137	511	
	Mean (SD)	2.2 (4.10)	3.9 (7.43)	2.7 (5.25)	0.0528
	Median	0.0	0.0	0.0	
	Min/ Max	0/24	0/49	0/49	
Cycles 2 - 6	n	422	172	594	
	Mean (SD)	4.3 (7.37)	8.2 (14.29)	5.4 (10.03)	0.0010
	Median	1.0	2.0	1.0	
	Min/ Max	0/45	0/127	0/127	
Cycles 2 - 9	n	305	116	421	
	Mean (SD)	6.5 (10.51)	12.3 (20.49)	8.1 (14.20)	0.0089
	Median	1.0	3.0	2.0	
	Min/ Max	0/56	0/128	0/128	

Source: Section 15.4, Table 15.4.1.22
N: Number of subjects in specified treatment group
n: Number of subjects in with data available
SD: Standard deviation

Number of unscheduled spotting days

A summary of unscheduled spotting days by treatment cycle is presented in Table 100 below. The number of unscheduled spotting days was higher than that of unscheduled bleeding days in both groups. In each reference period and during the overall treatment period (excluding Cycle 1) the Test group subjects tended to record less unscheduled spotting days than the Reference group subjects. The differences between the groups in mean number of unscheduled spotting days were statistically significant, except Cycles 5-7 and Cycles 7-9. During the entire treatment period, excluding Cycle 1, the mean (SD) number of unscheduled days in the Test group was 15.1 (16.39) compared to 22.4 (21.54) days in the Reference group, and the difference was statistically significant (p = 0.0011, Wilcoxon-rank-sum-test). The median number of days was 9.0 vs. 15.0 days.

Table 100: Number of Days with Unscheduled Spotting by Treatment Period (FAS)

Cycle		Test (N=858)	Reference (N=332)	Total (N=1190)	Wilcoxon-rank-sum-test p-value
Cycle 2 - 4	n	527	222	749	
	Mean (SD)	6.7 (8.71)	11.4 (11.88)	8.1 (9.98)	<0.0001
	Median	3.0	6.0	4.0	
	Min/ Max	0/54	0/58	0/58	
Cycle 5 - 7	n	423	157	580	
	Mean (SD)	5.2 (6.98)	6.7 (8.38)	5.6 (7.41)	0.1049
	Median	2.0	3.0	3.0	
	Min/ Max	0/67	0/41	0/67	
Cycle 7 - 9	n	374	137	511	
	Mean (SD)	5.0 (6.56)	7.0 (8.61)	5.5 (7.21)	0.0535
	Median	2.0	4.0	3.0	
	Min/ Max	0/46	0/40	0/46	
Cycles 2 - 6	n	422	172	594	
	Mean (SD)	9.4 (11.53)	15.6 (16.49)	11.2 (13.44)	<0.0001
	Median	5.0	8.0	6.0	
	Min/ Max	0/81	0/85	0/85	
Cycles 2 - 9	n	305	116	421	
	Mean (SD)	15.1 (16.39)	22.4 (21.54)	17.1 (18.23)	0.0011
	Median	9.0	15.0	11.0	
	Min/ Max	0/79	0/86	0/86	

Source: Section 15.4, Table 15.4.1.21
N: Number of subjects in specified treatment group
n: Number of subjects in with data available
SD: Standard deviation

12.7.8 Number of bleeding days by intensity

Numbers of days with slight, moderate and heavy bleeding by treatment period are presented in Table 101. No relevant differences between the groups were observed with regard to the mean number of days in each category of bleeding intensity (Section 15.4, Tables 15.4.1.17, 15.4.1.18 and 15.4.1.19). Heavy bleeding was uncommon, median number of days in both groups was 0.0 at all treatment cycles.

Table 101: Number of Days with Slight, Moderate and Heavy Bleeding by Treatment Period (FAS)

Cycle		Slight bleeding		Moderate bleeding		Heavy bleeding	
		Test (N=858)	Reference (N=332)	Test (N=858)	Reference (N=332)	Test (N=858)	Reference (N=332)
Cycles 2 - 4	n	527	222	527	222	527	222
	Mean (SD)	2.0 (3.77)	2.3 (4.34)	1.9 (3.25)	2.9 (6.63)	0.3 (0.93)	0.2 (0.78)
	Median	0.0	0.0	0.0	0.0	0.0	0.0
	Min/ Max	0/34	0/26	0/17	0/65	0/8	0/7
Cycles 5 - 7	n	423	157	423	157	423	157
	Mean (SD)	1.5 (3.05)	1.9 (5.40)	1.4 (2.71)	1.8 (4.51)	0.2 (0.67)	0.2 (0.79)
	Median	0.0	0.0	0.0	0.0	0.0	0.0
	Min/ Max	0/20	0/51	0/19	0/42	0/6	0/6
Cycles 7 - 9	n	374	137	374	137	374	137
	Mean (SD)	1.6 (3.06)	1.7 (5.05)	1.3 (2.50)	1.8 (3.64)	0.2 (0.72)	0.3 (1.32)
	Median	0.0	0.0	0.0	0.0	0.0	0.0
	Min/ Max	0/23	0/41	0/16	0/24	0/8	0/12
Cycles 2 - 6	n	422	172	422	172	422	172
	Mean (SD)	3.1 (5.45)	3.8 (7.20)	2.8 (4.58)	4.0 (9.90)	0.4 (1.33)	0.3 (1.07)
	Median	1.0	0.5	0.0	0.0	0.0	0.0
	Min/ Max	0/43	0/61	0/24	0/107	0/12	0/8
Cycles 2 - 9	n	305	116	305	116	305	116
	Mean (SD)	4.8 (8.14)	5.2 (11.60)	3.8 (6.64)	6.3 (13.09)	0.5 (1.44)	0.8 (2.06)
	Median	1.0	1.0	1.0	1.0	0.0	0.0
	Min/ Max	0/55	0/87	0/37	0/107	0/10	0/12

Source: Section 15.4, Table 15.4.1.17, Table 15.4.1.18 and Table 15.4.1.19
N: Number of subjects in specified treatment group
n: Number of subjects with data available
SD: Standard deviation

12.7.9 Number of bleeding or spotting episodes

An episode of bleeding/spotting was defined in the SAP as bleeding/spotting days bounded on either end by two days of no bleeding or spotting.

The groups were similar with regard to the mean (2.6 episodes) and median numbers (3.0 episodes) of bleeding/spotting episodes during the first reference period, Cycles 2-4, see Table 102 below.

During the second and the third reference periods the mean (SD) numbers of bleeding or spotting episodes were statistically significantly higher in the Test than in the Reference group: 2.3 (2.03) vs. 1.9 (1.90) episodes (p = 0.0418, Wilcoxon-rank-sum-test) in Cycles 5-7, and 2.3 (2.01) vs. 1.7 (1.84) episodes (p = 0.0054, Wilcoxon-rank-sum-test) in Cycles 7-9. The medians were 2.0 episodes in the Test vs. 1.0 episode in the Reference group in the second and third reference period, ranging from 0 to 14 episodes in the Test and from 0 to 9 episodes in the Reference group.

Table 102: Number of Bleeding and/or Spotting Episodes by Reference Period (FAS)

Cycles		Test (N=858)	Reference (N=332)	Total (N=1190)	Wilcoxon-rank-sum-test p-value
Cycles 2 - 4	n	527	222	749	
	Mean (SD)	2.6 (2.02)	2.6 (1.92)	2.6 (1.99)	0.9168
	Median	3.0	3.0	3.0	
	Min/ Max	0/9	0/9	0/9	
Cycles 5 - 7	n	423	157	580	
	Mean (SD)	2.3 (2.03)	1.9 (1.90)	2.2 (2.00)	0.0418
	Median	2.0	1.0	2.0	
	Min/ Max	0/11	0/7	0/11	
Cycles 7 - 9	n	374	137	511	
	Mean (SD)	2.3 (2.01)	1.7 (1.84)	2.1 (1.98)	0.0054
	Median	2.0	1.0	2.0	
	Min/ Max	0/14	0/9	0/14	

Source: Section 15.4, Table 15.4.1.23
N: Number of subjects in specified treatment group
n: Number of subjects with data available
SD: Standard deviation

Unscheduled bleeding/spotting episodes

The mean and median numbers of unscheduled bleeding/spotting episodes during each reference period are presented in Table 103.

Table 103: Number of Unscheduled Bleeding and/or Spotting Episodes by Reference Period (FAS)

Cycles		Test (N=858)	Reference (N=332)	Total (N=1190)	Wilcoxon-rank-sum-test p value
Cycles 2 - 4	n	527	222	749	
	Mean (SD)	1.7 (1.55)	2.6 (1.92)	2.0 (1.72)	<0.0001
	Median	1.0	3.0	2.0	
	Min/ Max	0/7	0/9	0/9	
Cycles 5 - 7	n	423	157	580	
	Mean (SD)	1.5 (1.56)	1.9 (1.90)	1.6 (1.67)	0.0535
	Median	1.0	1.0	1.0	
	Min/ Max	0/9	0/7	0/9	
Cycles 7 - 9	n	374	137	511	
	Mean (SD)	1.5 (1.54)	1.7 (1.84)	1.6 (1.63)	0.5238
	Median	1.0	1.0	1.0	
	Min/ Max	0/9	0/9	0/9	

Source: Section 15.4, Table 15.4.1.24
N: Number of subjects in specified treatment group
n: Number of subjects with data available
SD: Standard deviation

The mean numbers of unscheduled bleeding/spotting episodes at each reference period were lower in the Test group compared to the Reference group. The difference between the mean [SD] numbers of unscheduled episodes was statistically significant only in Cycles 2-4 (1.7 [1.55] episodes in the Test vs. 2.6 [1.92] episodes in the Reference group; $p < 0.0001$, Wilcoxon-rank-sum-test) and not significant in Cycles 5-7 and Cycles 7-9. The median number of unscheduled bleeding/spotting episodes in the Test group was 1.0 episode in each reference period, and that in the Reference group was 3.0 episodes in the first and 1.0 episode in the second and the third reference periods.

12.7.10 Incidence of TEAEs based on abnormal vaginal (or uterine) bleeding

In total, 46 (5.4%) Test group and 31 (9.3%) Reference group subjects experienced bleeding-related TEAEs, the majority of which were considered at least possibly related to IMP, see Table 104.

The vast majority of bleeding TEAEs were of mild or moderate severity, whereas TEAEs of severe intensity were reported for four Test and three Reference group subjects.

A total of 28 (3.3%) Test group and 22 (6.6%) Reference group subjects discontinued early due to bleeding TEAEs.

Table 104: Summary of Subjects with TEAEs Based on Abnormal Bleeding (SS)

Preferred Term	Test (N=858)				Reference (N=332)			
	Total n (%)	Related n (%)	Severe n (%)	Withdrew n (%)	Total n (%)	Related n (%)	Severe n (%)	Withdrew n (%)
Vaginal haemorrhage	32 (3.7)	27 (3.1)	2 (0.2)	22 (2.6)	24 (7.2)	20 (6.0)	2 (0.6)	18 (5.4)
Dysmenorrhoea	8 (0.9)	5 (0.6)	1 (0.1)	1 (0.1%)	2 (0.6)	1 (0.3)	1 (0.3)	0
Uterine haemorrhage	5 (0.6)	5 (0.6)	1 (0.1)	5 (0.6)	5 (1.5)	5 (1.5)	0	3 (0.9)
Metrorrhagia	3 (0.3)	3 (0.3)	0	0	1 (0.3)	1 (0.3)	0	1 (0.3%)
Menorrhagia	0	0	0	0	1 (0.3)	1 (0.3)	0	0
Total	46 (5.4)	39 (4.5)	4 (0.5)	28 (3.3)	31 (9.3)	26 (7.8)	3 (0.9)	22 (6.6)

Source: Section 15.3, Tables 15.3.1.2, 15.3.1.4, 15.3.1.5 and 15.3.1.8
N: Number of subjects in specified treatment group
n: Number of subjects with TEAEs
%: Percentage based on N

12.7.11 Return of fertility

In total four Test group (Subjects: #260004, #351030, #454002 and #653035) subjects and one Reference group subject (#559003) prematurely terminated the trial due to wish of pregnancy (Appendix 16.2, Listing 16.2.1.2). These subjects were followed up to one year after discontinuation of the trial, the results were as follows:

- Subject #351030 received drospirenone for 57 days and Subject #454002 received drospirenone for 57 days. Both of them had not got pregnant within one year after discontinuation of the trial.
- Subjects #260004 and #559003 changed their plans regarding pregnancy.
- Subject #653035 was lost to follow-up.

The relevant correspondence regarding these subjects is filed in the pharmacovigilance part of the TMF.

12.8 Safety and Tolerability Conclusions

The mean (SD) treatment duration was 222.7 (65.79) days in the Test group and 213.9 (72.14) days in the Reference group. The median duration was 252.0 days in both groups, ranging from 3 to 276 days in the Test and from 1 to 280 days in the Reference group.

The proportion of subjects with TEAEs was lower in the Test than in the Reference group (38.7% vs. 45.2%), and this difference was statistically significant (p = 0.042, Fisher's exact test). The most frequently affected SOCs were infections and infestations, and reproductive system and breast disorders.

The most common individual TEAEs in both treatment groups were vaginal haemorrhage (3.7% of the Test and 7.2% of the Reference group subjects), headache (4.4% Test and 5.1%

Reference group subjects), acne (3.1% Test and 5.7% Reference) and nasopharyngitis (3.4% Test and 3.9% Reference). Vaginal haemorrhage and acne were more frequent in the Reference group than in the Test group. The treatment groups were comparable with regard to the frequencies of the other TEAEs. The treatment groups were comparable with regard to the frequencies of other most frequent TEAEs.

The most common TEAEs considered by the investigators as at least possibly related were vaginal haemorrhage, acne and weight increased.

The vast majority of TEAEs were classified as mild or moderate, severe TEAEs were reported for 2.8% of the Test and 3.3% of the Reference group subjects.

There were no deaths reported. In total 15 (1.7%) Test group and six (1.8%) Reference group subjects experienced treatment emergent SAEs. Of these, two TESAEs, hepatic adenoma reported in the Test group and ectopic pregnancy reported in the Reference group were assessed as possibly related to study treatment and were reported as SUSARs.After the database lock, some doubts have arisen regarding the diagnosis of hepatic adenoma in favour of focal nodular hyperplasia. The diagnosis will be clarified in July 2014, when the results of MRI and ultrasound examination are available.

TEAEs of special interest (hyperkalaemia and blood potassium increased) were reported for two Test group subjects. The subjects did not present clinical signs related to hyperkalaemia. In the Reference group no TEAEs based on increased blood potassium levels were reported. No VTE cases were reported during the trial.

Overall 82 (9.6%) Test group and 44 (13.3%) Reference group subjects experienced TEAEs, leading to premature termination of the trial. The most frequent TEAEs leading to withdrawal were vaginal haemorrhage (2.6% of the Test and 5.4% of the Reference group subjects) and acne (1.0% of the Test and 2.7% of the Reference group subjects).

Overall 12 pregnancies occurring after the start of IMP intake were reported in this trial (six on-treatment and six post-treatment). All pregnancies occurred in the Test group, except one extrauterine pregnancy in the Reference group. Five (including twins) normal male babies were born.

Haematology, biochemistry, TSH and urinalysis laboratory assessments were performed in all trial subjects, while haemostatic, carbohydrate metabolism and bone metabolism assessments were performed in a subset of 68 subjects.

The changes of haematology, biochemistry, TSH, haemostatic and carbohydrate metabolism parameters over time were not clinically relevant and the between-group differences were small.

The levels of bone remodelling markers (bone alkaline phosphatase and Beta-CTX) were within the range for premenopausal women, not treated with contraceptives and no statistically significant differences between the treatment groups were found. The changes over time in each treatment group were assessed as not clinically significant.

The mean [SD] weight increase from baseline to endpoint was less pronounced in the Test group than in the Reference group (0.1 [3.2] kg vs. 0.5 [3.1] kg), the difference between the groups was statistically significant (p=0.0296, ANCOVA with age and baseline value as covariates and treatment group as a fixed factor). The mean (SD) BMI in the Test group increased by 0.04 (1.17) kg/m^2, and that of the Reference group by 0.20 (1.11) kg/m^2, with a statistically significant difference (p = 0.0331, ANCOVA).

No relevant changes in blood pressure or heart rate over time or differences between the groups were observed for the Safety Set, as well as for the age and BMI subgroups. In the

subgroup of subjects with SBP≥130 mmHg or DBP≥85 mmHg, blood pressure decreased over time: The median change in SBP from baseline at endpoint was -7.0 mmHg in the Test and -8.0 mmHg in the Reference group. The median change of DBP was -5.5 mmHg in the Test and -5.0 mmHg in the Reference group.

The incidence of abnormal gynaecological, cervical cytology, TVUS examination and physical examination findings assessed as clinically significant was low.

The data of this trial did not show a clinically meaningful effect in the Test group on the QTcF interval as well as the other ECG intervals (heart rate, QRS and PR intervals) alone or compared to the Reference group.

Tolerability

The tolerability analyses were focused on bleeding pattern changes. The proportion of subjects with bleeding and spotting decreased from 69.7 % in Cycle 2 to 56.3 % in Cycle 9 in the Test and from 74.0% to 45.3% in the Reference group; the overall median number of bleeding and spotting days decreased from 10 days (first reference period: Cycles 2 to 4) to 6 days (last reference period: Cycles 7 to 9) in the Test and from 12 to 7 days in the Reference group. Among these spotting days prevailed.

The proportion of subjects with unscheduled bleeding/spotting during Cycles 2-6 was lower in the Test than in the Reference group (73.0% vs. 88.4%), with the difference (95% CI) of -15.39% (-21.78%; -8.99%) between the groups. During Cycles 2-6, the Test treatment was superior to the Reference treatment with regard to the proportion of subjects with unscheduled bleeding.

The highest proportion of subjects with unscheduled bleeding or spotting was observed in Cycle 2: 51.4% of the Test and 74.0% of the Reference group subjects. The incidence of unscheduled bleeding decreased over time in both groups, to 43.9% of the Test and 45.3% of the Reference group subjects in Cycle 9. In each cycle up to Cycle 7, the proportion of subjects with unscheduled bleeding was statistically significantly lower in the Test group than in the Reference group.

The mean [SD] number of unscheduled bleeding and spotting days during Cycles 2-9 was statistically significantly lower in the Test than in the Reference group (21.5 [22.86] days vs. 34.7 [33.73] days; p = 0.0003, Wilcoxon-rank-sum-test). The mean number of days with unscheduled bleeding and spotting decreased over time and was lower in the Test than in the Reference group in each reference period and the difference was statistically significant.

From Cycle 2 to Cycle 9 the proportion of subjects who had no bleeding or spotting increased from 30.3% to 43.7% in the Test and from 26.0% to 54.7% in the Reference group.

The percentage of subjects with frequent bleeding gradually decreased over time from 9.1% during Cycles 2-4 to 5.3% during Cycles 7-9 in the Test group and from 7.2% to 4.4% in the Reference group and was comparable between the treatment groups in each reference period. The percentage of subjects who experienced prolonged bleeding decreased from 12.1% during Cycles 2-4 to 2.9% during Cycles 7-9 in the Test group and from 16.7% to 10.9% in the Reference group. The incidence of prolonged bleeding in each reference period was lower in the Test than in the Reference group, with statistically significant differences between the groups in the second and in the third reference period.

The median number of unscheduled bleeding/spotting episodes in the Test group was 1.0 episode in each reference period, and that in the Reference group was 3.0 episodes in the first and 1.0 episode in the second and the third reference periods. The difference between the mean [SD] numbers of unscheduled episodes was statistically significant only in Cycles 2-4

(1.7 [1.55] episodes in the Test vs. 2.6 [1.92] episodes in the Reference group; $p < 0.0001$, Wilcoxon-rank-sum-test) and not significant in Cycles 5-7 and Cycles 7-9.

With regard to bleeding, 46 (5.4%) Test group and 31 (9.3%) Reference subjects experienced vaginal (or uterine) bleeding-related TEAEs, 28 (3.3%) Test group subjects and 22 (6.6%) Reference group subjects discontinued prematurely due to TEAEs, and four (0.5 %) Test group and three (0.9%) Reference group subjects had severe TEAEs.

1.0 TITLE PAGE

INTEGRATED SUMMARY OF SAFETY
Drospirenone 4.0 mg
Indication: Oral Contraception
STATISTICAL ANALYSIS PLAN
Sponsor: Exeltis USA, Inc.
180 Park Ave, Suite 101
Florham Park, NJ 07932
Author: Xiuchun Ge, PhD
Statistician
BDM Consulting, Inc.
100 Franklin Square, Suite 305
Somerset, NJ 08873
Version: Final Version 1.0
Date: 15-JUN-2018
Confidentiality Statement
This document is the property of Exeltis USA, Inc. and may not—in full or part—be passed on, reproduced, published, distributed to any person, or submitted to any regulatory authority without the express written permission of Exeltis USA, Inc.
Sponsor: Exe/tis USA, Inc,
Product: LFl 11 (drospire11011e 4 mg tablets) NDA 211367
A11alysis: J11tegrated Summmy of Safety (ISS)
Pagel
Document Title:
Prnposed Imlication:
Document Date:
Document Version:
SAP APPROVAL FORM
Statistical Analysis }lan - Integrated Summary of Safety
Drospirenone for Oral Contraception
15 June **2018**
Final Version 1.0
This Statistical Analysis Plan has been reviewed and approved by:
Apprnved by:
Xiuchun Ge, PhD
Statistician
BDM Consulting, Inc.
Belen Liebana
Clinical Operations Manager
Chemo Research S.L.

i /\A

Enric¥1!7.MD
Chief Scientific Officer
Chemo Research S.L.
StaNstical Analysis Plan
Date
Date
Date
J1111e 15, 2018
Sponsor: Exeltis USA, Inc. Analysis: Integrated Summary of Safety (ISS)
Product: LF111 (drospirenone 4 mg tablets) NDA 211367 Page 3
Statistical Analysis Plan June 15, 2018

2.0 TABLE OF CONTENTS

1.0 TITLE PAGE.......... 1
2.0 TABLE OF CONTENTS.......... 2
3.0 LIST OF ABBREVIATIONS.......... 5
4.0 INTRODUCTION 7
5.0 OVERVIEW OF THE CLINICAL STUDIES.......... 8
5.1 Group 1: Phase III Studies in healthy WOMEN with risk of pregancy.......... 9
5.2 Group 2: Phase II Studies in healthy WOMEN with risk of pregancy 10
5.3 Group 3: Phase I Pharmacology Studies..........11
5.4 General Specifications 12
5.4.1 Pooled ISS Database..........12
5.4.2 Analysis Strategy..........12
5.4.3 Baseline and End of Treatment 13
5.4.4 General Handling of TEAEs and SAEs..........13
6.0 ANALYSIS POPULATION..........14
7.0 SUBJECT DISPOSITION..........15
8.0 OVERALL EXTENT OF EXPOSURE TO INVESTIGATIONAL MEDICINAL PRODUCT....16
9.0 DEMOGRAPHIC AND OTHER BASELINE CHARACTERISTICS 17
10.0 PRIOR AND CONCOMITANT MEDICATIONS 19
11.0 ADVERSE EXPERIENCES IN CLINICAL STUDIES.......... 20
11.1 Treatment-Emergent Adverse Events by System Organ Class..........20
11.2 Deaths 20
11.3 Other Serious Adverse Events 21
11.4 Bleeding Data Analysis 21
11.5 Other Significant Adverse Events..........21
12.0 CLINICAL LABORATORY EVALUATIONS..........23
13.0 VITAL SIGNS..........26
14.0 SAFETY IN SPECIAL GROUPS AND SITUATIONS.......... 27
15.0 COMPUTER METHODS..........28
16.0 DATA HANDLING CONVENTIONS 29
16.1 Visit Time Windows..........29
16.2 Missing Data Handling 29
16.2.1 Missing Date of IMP 29
16.2.2 Missing Severity Assessment for Adverse Events 29
16.2.3 Missing Relationship to IMP for Adverse Events 29

Sponsor: Exeltis USA, Inc. Analysis: Integrated Summary of Safety (ISS)
Product: LF111 (drospirenone 4 mg tablets) NDA 211367 Page 4
Statistical Analysis Plan June 15, 2018

16.2.4 Missing Date Information for Adverse Events 29
16.2.5 Missing Date Information for Prior or Concomitant Medications..........30
17.0 REFERENCES 33

Sponsor: Exeltis USA, Inc. Analysis: Integrated Summary of Safety (ISS)
Product: LF111 (drospirenone 4 mg tablets) NDA 211367 Page 5
Statistical Analysis Plan June 15, 2018

3.0 LIST OF ABBREVIATIONS

ADO Adverse event leading to dropout
AE(s) Adverse Event(s)
ALP Alkaline Phosphatase
ALT Alanine Aminotransferase
AST Aspartate Aminotransferase
ATC2 ATC2 Drug Class Terms
BA Bioavailability
BMI Body Mass Index
bpm Beats per Minute
BUN Blood Urea Nitrogen
CK Creatine Kinase
cm Centimeter
CRF Case Report Form
DBP Diastolic Blood Pressure

eCRF Electronic Case Report Form
FAS Full Analysis Set
GGT Gamma Glutamyl Transferase
HDL High-density Lipoprotein
IMP Investigational Medicinal Product
ISS Integrated Summary of Safety
kg Kilogram
LDH Lactate Dehydrogenase
LDL Low Density Lipoprotein
m^2
MCH Mean Corpuscular/cellular Haemoglobin
Square meter
MCV Mean Corpuscular Volume
MedDRA Medical Dictionary for Regulatory Activities
MFAS Modified Full Analysis Set
NDA New Drug Application
PCS Potentially Clinically Significant

PK Pharmacokinetics
RBC Red Blood Cell
SAE(s) Serious Adverse Event(s)
SBP Systolic Blood Pressure
SI *Le Système International d'Unités* (International System of Units)
SOC System Organ Class
TEAE(s) Treatment-emergent Adverse Event(s)
ULN Upper Limit of Normal
WBC White Blood Cell
WHO World Health Organization

4.0 INTRODUCTION

The objective of this Statistical Analysis Plan is to describe the detailed statistical methods used in the Integrated Summary of Safety (ISS) for the New Drug Application (NDA) of Drospirenone 4.0 mg for oral contraceptive. Specifications of the ISS tables, figures, and data listings are contained in a separate document.

5.0 OVERVIEW OF THE CLINICAL STUDIES

The drospirenone clinical development program includes 19 clinical studies (9 Phase 1 studies and 10 Phase 2/3 studies). For the purpose of the ISS, the 19 clinical studies have been organized into 3 distinct groups, based on the study design such as subject population and treatment duration. Refer to Table 5.1.1-1 to Table 5.3-1.

5.1 GROUP 1: PHASE III STUDIES IN HEALTHY WOMEN WITH RISK OF PREGANCY

Table 5.1-1 describes the 5 Phase III studies, with 1 randomized, double-blind, activecontrolled study CF111/302. The 5 studies are organized into 2 groups: 1A and 1B.
Group 1A contains studies CF111/301, CF111/302, CF111/303, and CF111/205; Group

1B contains the adolescent study CF111/304. The primary safety analyses will be based on pooled Group 1A studies. The safety data from comparative treatment, as randomized in study CF111/302, will not be pooled in the integrated analysis for Group 1A. Group 1B (i.e., CF111/304) will not be pooled with any other studies and its safety results will be presented and discussed in the ISS report along with Group 1A results.

Table 5.1–1 Overview of Phase III Studies

Study No.
(Country) Study Objectives Design
Treatment
Duration
(Cycles*)
Treatment
(Sample Size
a
b)

Group 1A: Adult Subjects Studies

CF111/301
(Czech Republic,
Germany, Hungary,
Poland,
Romania)
Contraceptive
efficacy, safety, and
tolerability of
drospirenone 4.0 mg
Phase III, multinational,
multicenter, open-label,
non-controlled,
fixed-dose study
13*
Drospirenone
4.0 mg
(FAS and Safety:
713)
CF111/302
(Austria, Czech
Republic, Germany,
Hungary, Poland,
Romania, Slovakia,
Spain)
Contraceptive
efficacy, safety, and
tolerability of
drospirenone 4.0 mg
vs desogestrel 0.075
mg
Phase III, multinational,
multicenter,
randomised, active
control, double-blind,
double-dummy
9
Drospirenone
4.0 mg
(Randomized: 872;
FAS and Safety:
858)
CF111/303
(United States)
Contraceptive
efficacy, safety,
tolerability and

pharmacokinetics of
drospirenone 4.0 mg
Phase III, multicenter,
open-label, noncontrolled,
including
adolescents between the
ages of 15-17.
13
Drospirenone
4.0 mg
(FAS: 1004;
MFAS: 993;
Safety:1006)
CF111/205
(Bulgaria) Endometrial safety
Phase 3*, monocentric,
open, multiple
dose trial in healthy
female subjects at risk
of pregnancy
13
Drospirenone
4.0 mg 24/4
(Safety: 21)
Group 1B: Adolescents Only Study
CF111/304
(Finland, Germany,
Sweden, Ukraine)
Safety, and
tolerability of
drospirenone 4.0 mg
Phase III, Multicentre,
Open-Label Trial to
Assess the Safety and
Tolerability of LF111
(Drospirenone 4.0 mg)
Over 6 Cycles in
Female Adolescents,
6+7
Drospirenone
4.0 mg
(Safety: 102)
Sponsor: Exeltis USA, Inc. Analysis: Integrated Summary of Safety (ISS)
Product: LF111 (drospirenone 4 mg tablets) NDA 211367 Page 10
Statistical Analysis Plan June 15, 2018
With a 7-Cycle
Extension Phase
Note: * As indicated in the study protocol.
a Drospirenone 4.0 mg 24/4: Drospirenone 4 mg for 24 days and placebo for 4 days for a cycle
of 28 days.
b The Safety Set.
FAS = Full Analysis Set; MFAS = Modified Full Analysis Set.

5.2 GROUP 2: PHASE II STUDIES IN HEALTHY WOMEN WITH RISK OF PREGANCY

Table 5.2-1 describes the 5 Phase II studies, 3 of which were randomized. Safety data from these studies will not be pooled for analysis due to different study designs. Safety results for each individual study will be presented in the ISS report.

Table 5.2–1 Overview of Phase II Studies

Study No. (Country) Study Objectives Design
Treatment
Duration

(Cycles)
Treatment
(Sample Size
a
b)
CF111/201A
(Tunis)
Efficacy and
safety
Phase 2, single-center,
open label multiple
dose trial in healthy
female subjects
female volunteers of
childbearing potential
2
Drospirenone
4.0 mg 24/4
(20)
CF111/201B
(France)
Tolerability and
safety
Phase 2, single-center,
open label, randomized
trial in healthy female
subjects at risk of
pregnancy
3
Drospirenone
4.0 mg 24/4
(10)
CF111/202
(Germany)
Efficacy and
safety
Phase 2, single-center,
open label, randomized
multiple
dose trial in healthy
female subjects at risk
of pregnancy
1+2+1
Drospirenone
4.0 mg 24/4
(32)
CF111/203
(Germany)
Efficacy and
safety
Phase 2, single-center,
open label, randomized
multiple
dose trial in healthy
female subjects at risk
of pregnancy
2
Drospirenone
4.0 mg 24/4 or
2.8 mg 28 days
(27, 25)
CF111/204 Maintenance of Phase 2, single-center,
open, multiple 2 Drospirenone

Sponsor: Exeltis USA, Inc. Analysis: Integrated Summary of Safety (ISS)
Product: LF111 (drospirenone 4 mg tablets) NDA 211367 Page 11
Statistical Analysis Plan June 15, 2018
(Germany) ovulation
inhibition and
safety
dose trial in healthy
female subjects at risk
of pregnancy
4.0 mg 24/4
(127)
Note: a Drospirenone 4.0 mg 24/4: Drospirenone 4 mg for 24 days and placebo for 4 days for a
cycle of 28 days.
b The Safety Set.

5.3 GROUP 3: PHASE I PHARMACOLOGY STUDIES

Phase I pharmacology studies are presented in Table 5.3-1. Data from these studies will not be pooled in this ISS and will be summarized separately for each study in the ISS report.

Table 5.3–1 Overview of Pharmacology Studies (Phase I)

Study No. (Country) Study
Objectives Design Treatment
Duration
Treatment
(Sample Size
a
b)
CF111/101A
(Turkey) BA
Phase 1, single-center, open
label, randomized 2×2
crossover Single dose Drospirenone
3.0 mg (14)
CF111/101B
(Turkey) BA
Phase 1, single-center, open
label, randomized 2×3
crossover Single dose
Drospirenone
3.0 mg
(14)
CF111/102
(Turkey) BA
Phase 1, single-center, open
label, randomized 2×2
crossover Single dose
Drospirenone
4.0 mg
(10)
CF111/103A
(Bulgaria) BA Phase 1, open label,
crossover
Single and
repeated doses
(1+12 days)
Drospirenone
4.0 mg
(24)
CF111/103C
(Canada)
BA
(Under fed
conditions)

Phase 1, single-center, open
label, randomized 2×2
crossover Single dose
Drospirenone
4.0 mg
(32)
CF111/104
(France) BA
Phase 1, single-center, open
label, randomized 2×2
crossover Single dose
Drospirenone
4.0 mg 2 batches
(8)
CF111/105
(Bulgaria) BA
Phase 1, single-center, open
label, randomized threetreatment,
three-period, sixsequence
crossover
Single dose
Drospirenone
4.0 mg 3 batches
(14)
CF111/106
(Bulgaria) Food effect Phase 1, single-center, open
label, randomized 2×2 Single dose Drospirenone
4.0 mg with and
Sponsor: Exeltis USA, Inc. Analysis: Integrated Summary of Safety (ISS)
Product: LF111 (drospirenone 4 mg tablets) NDA 211367 Page 12
Statistical Analysis Plan June 15, 2018
crossover w/o food
(24)
CF111/107
(Latvia)
Transfer in
milk
Phase 1, single-center, open
label, noncomparative in
healthy lactating female
volunteers
Multiple dose
Drospirenone
4.0 mg
(12)
Note: a Drospirenone 4.0 mg 24/4: Drospirenone 4 mg for 24 days and placebo for 4 days for a
cycle of 28 days.
b The Safety Set.
BA = bioavailability.

5.4 GENERAL SPECIFICATIONS

5.4.1 Pooled ISS Database

The integrated safety analyses for the oral contraceptive indication will be primarily based on the Phase III studies (Group 1A). The pooled database of Group 1A studies will include the following information for each subject: demographics and other baseline characteristics, medical history, physical examinations, treatment exposure and duration, adverse events (AEs), clinical laboratory parameters, vital signs, and concomitant medications.

For Group 1B (Study CF111/304), analyses of common treatment-emergent adverse events (TEAEs) and AEs of special interests as specified in Section 11 will be performed. No other analysis is planned.

5.4.2 Analysis Strategy

Table 5.4.2-1 provides a brief summary of planned analysis.

Sponsor: Exeltis USA, Inc. Analysis: Integrated Summary of Safety (ISS)
Product: LF111 (drospirenone 4 mg tablets) NDA 211367 Page 13
Statistical Analysis Plan June 15, 2018

Table 5.4.2–1 Overview of Safety Analysis

Group 1A Subgroups Group 1B Subgroups

Subject disposition X X
Demographics X
IMP exposure X X
Medical History X
Concomitant medications X
AE (Overall summary and
TEAE by SOC and preferred
term; Common TEAE)
X X X (common
TEAE)
Special Interest AEs X X X
Death/SAE/ADO X
AE (severity and relationship
to IMP) X X
AE by exposure X
Clinical laboratory
(descriptive statistics, PCS,
shift tables)
X
Vital signs (descriptive
statistics, PCS, etc) X
Bleeding Pattern X X

Note: AE = adverse event; TEAE = Treatment-emergent adverse event; SAE = serious adverse event;
SOC = system organ class; ADO = Dropout due to AE; PCS = potentially clinically significant;
IMP = investigational medicinal product.

5.4.3 Baseline and End of Treatment

The *Baseline* for the safety parameters (i.e., clinical laboratory parameters, vital signs etc.) for Group 1 is defined as the last non-missing value before the first dose of investigational medicinal product (IMP). End of treatment is defined as the last nonmissing value while subject was on IMP.

5.4.4 General Handling of TEAEs and SAEs

The Medical Dictionary for Regulatory Activities (MedDRA) Version 17.0 will be used for all the coding. Terms that use other versions will be recoded using MedDRA 17.0.

Sponsor: Exeltis USA, Inc. Analysis: Integrated Summary of Safety (ISS)
Product: LF111 (drospirenone 4 mg tablets) NDA 211367 Page 14
Statistical Analysis Plan June 15, 2018

6.0 ANALYSIS POPULATION

The Safety Set consists of all subjects who have taken at least one dose of IMP.
All safety data will be summarized based on the Safety Set primarily using descriptive statistics, unless specified otherwise.

Sponsor: Exeltis USA, Inc. Analysis: Integrated Summary of Safety (ISS)
Product: LF111 (drospirenone 4 mg tablets) NDA 211367 Page 15
Statistical Analysis Plan June 15, 2018

7.0 SUBJECT DISPOSITION

The number and percentage of subjects who completed and prematurely discontinued during the treatment period will be summarized for the Safety Set for Group 1A pooled. Reasons for premature discontinuation, as recorded on the termination page of the case report form, will be summarized (number and percentage) for the Safety Set.
Disposition and withdrawal data will include the following:

- Subjects enrolled
- Safety Set

• Subjects completing the study
• Premature discontinuations, reasons including:
- At subject's own request (withdrawal of consent)
- Investigator's opinion
- Major protocol violations
- Pregnancy
- Wish for pregnancy
- Ineligibility
- Adverse event
- Lost to follow-up
- Other
Sponsor: Exeltis USA, Inc. Analysis: Integrated Summary of Safety (ISS)
Product: LF111 (drospirenone 4 mg tablets) NDA 211367 Page 16
Statistical Analysis Plan June 15, 2018
8.0 OVERALL EXTENT OF EXPOSURE TO INVESTIGATIONAL MEDICINAL PRODUCT
Exposure to IMP during the treatment period will be summarized in terms of treatment duration. The treatment duration of a subject will be calculated as the number of days from the date of first dose of IMP taken to the date of last dose of IMP taken, inclusive. Descriptive statistics (n, mean, standard deviation, minimum, median, and maximum) for treatment duration will be presented for Group 1A. Frequency tabulations (numbers and percentages of subjects) will also be provided for the following categories of cumulative treatment exposure:
• ≥ 28 days
• ≥ 84 days
• ≥ 168 days
• ≥ 252 days
The extent of exposure summaries will also be presented for the relevant subgroups (see Section 14.0).
Sponsor: Exeltis USA, Inc. Analysis: Integrated Summary of Safety (ISS)
Product: LF111 (drospirenone 4 mg tablets) NDA 211367 Page 17
Statistical Analysis Plan June 15, 2018
9.0 DEMOGRAPHIC AND OTHER BASELINE CHARACTERISTICS
The demographic profile, which consists of key demographics and baseline characteristics, will be summarized for Group 1A. The following demographic parameters will be included in the analysis: age, age group, race, weight, height, body mass index (BMI), smoking status, alcohol use, and education.
Regardless of whether the subject's age is collected on the case report form (CRF) or the electronic case report form (eCRF), age will be derived programmatically based on the date of birth and the informed consent date.
Demographic data analyzed include the following:
• Age categories (≤35 years, >35 years)
• Race (Caucasian, Black or African American, Asian, Other)
• Highest level of education completed (not completed high school, high school or equivalent, college/university degree or higher, other)
Baseline characteristics of the subjects include:
• Body height (cm)
• Body weight (kg)
• BMI (kg/m2
• BMI (kg/m

)
2
- <30
) categories
- ≥ 30
- • Systolic blood pressure (SBP, mmHg)
- < 130
- ≥ 130
- • Diastolic blood pressure (DBP, mmHg)
- < 85
- ≥ 85
- • Blood pressure (mmHg) categories
- SBP<130 and DBP < 85
- SBP≥130 or DBP ≥ 85
- • Smoking Status (current smoker, ex-smoker, and never)
- • Alcohol Use (drinker and never)

Sponsor: Exeltis USA, Inc. Analysis: Integrated Summary of Safety (ISS)
Product: LF111 (drospirenone 4 mg tablets) NDA 211367 Page 18
Statistical Analysis Plan June 15, 2018

Medical history including prior medical history and current medical history will be summarized for Group 1A. Prior medical history findings are defined as those starting and ending prior to screening visit; current medical history findings are defined as those ongoing at screening visit.

Descriptive statistics (n, mean, standard deviation, minimum, median, and maximum) will be presented for continuous variables. Frequency summaries (numbers and percentages) will be presented for categorical variables.

Sponsor: Exeltis USA, Inc. Analysis: Integrated Summary of Safety (ISS)
Product: LF111 (drospirenone 4 mg tablets) NDA 211367 Page 19
Statistical Analysis Plan June 15, 2018

10.0 PRIOR AND CONCOMITANT MEDICATIONS

The *World Health Organization (WHO) Drug Dictionary Version 01Mar2018C3* will be used to classify prior and concomitant medications by therapeutic class. For any studies coded with a different version of WHO Drug Dictionary or a different medical dictionary, recoding will be carried out using the current version (i.e., *Version 01Mar2018C3*) of WHO Drug Dictionary.

Prior medication will not be summarized for the ISS.

The *concomitant medication* is defined as any medication taken on or after the date of the first dose of the IMP and on or before the last dose of the IMP.

The number and percentage of subjects will be tabulated by ATC2 class and preferred drug name for the Safety Set. Multiple drug use by a subject in the same ATC2 class or preferred drug name will be counted only once in the summary tables.

Sponsor: Exeltis USA, Inc. Analysis: Integrated Summary of Safety (ISS)
Product: LF111 (drospirenone 4 mg tablets) NDA 211367 Page 20
Statistical Analysis Plan June 15, 2018

11.0 ADVERSE EXPERIENCES IN CLINICAL STUDIES

The Version 17.0 of the MedDRA will be used for coding AEs across all individual studies in Group 1. For any studies in the group in which AEs were coded with a different version of MedDRA or a different medical dictionary, AEs will be recoded using MedDRA Version 17.0.

An AE (classified by preferred term) will be considered a TEAE if it was not present before the date of the first dose of IMP or was present before the date of the first dose of IMP and increased in severity following the date of the first dose of IMP. If more than

one AE was reported before the date of the first dose of IMP and coded to the same preferred term, the AE with the greatest severity will be used as the benchmark for comparison with the AEs occurring following the date of the first dose of IMP. A TEAE that occurred more than 10 days after the date of the last dose of IMP will not be summarized.

11.1 TREATMENT-EMERGENT ADVERSE EVENTS BY SYSTEM ORGAN CLASS

The number and percentage of subjects with TEAEs will be summarized by system organ class and preferred term for Group 1A and for relevant subgroups (i.e., age group, etc.) in Group 1A.

For Group 1A, the number and percentage of subjects with TEAEs will also be tabulated by system organ class, preferred term, and severity; and by system organ class, preferred term, and relationship to IMP. If more than one event occurs during the study with the same preferred term for the same subject, the subject will be counted only once for that preferred term using the most severe or most related occurrence for the summarization by severity and by relationship to the IMP.

The number and percentage of common TEAEs, defined as TEAEs occurring in ≥ 2% of subjects, will be summarized by system organ class and preferred term for Group 1A and Group 1B.

TEAEs and common TEAEs will also be summarized for group 1A by IMP exposure periods that the TEAEs occurred: ≤28 days, >28-84 days, >84-168 days, >168-252 days and >252 days.

11.2 DEATHS

Deaths, if any, as captured on the CRFs during the treatment period or within 30 days following the date of the last dose of IMP will be summarized for Group 1A. A listing of all subjects who died, including deaths reported after 30 days following the date of the last dose of IMP, will be provided.

Sponsor: Exeltis USA, Inc. Analysis: Integrated Summary of Safety (ISS)
Product: LF111 (drospirenone 4 mg tablets) NDA 211367 Page 21
Statistical Analysis Plan June 15, 2018

11.3 OTHER SERIOUS ADVERSE EVENTS

The serious adverse events (SAEs) other than death (but including the SAEs temporally associated with or preceding the deaths) are defined as those stated in individual study protocols and captured on the CRFs. All SAEs that occurred during the treatment period or within 30 days following the date of the last dose of IMP will be summarized (number and percentage) for Group 1A and, as appropriate, for the relevant subgroups. A listing of all subjects with SAEs, including SAEs reported after 30 days following the date of the last dose of IMP, will be provided. All SAEs by exposure to IMP will also be summarized for Group 1A.

11.4 BLEEDING DATA ANALYSIS

Scheduled bleeding or spotting is defined as any bleeding or spotting that occurs during hormone-free intervals (defined as Days 25-28 ± 1 day). Bleeding or spotting that starts during this period and continues for up to eight consecutive days is considered as scheduled bleeding/spotting. Unscheduled bleeding or spotting is defined as any bleeding or spotting that occurs outside the time window defined for scheduled bleedings. An episode of bleeding or spotting is defined as bleeding or spotting days bounded on either end by two days of no bleeding or spotting.

Number and percentage of subjects with scheduled bleeding or spotting, and unscheduled bleeding or spotting will be presented for each cycle and reference periods (i.e., Cycles 2-4, 5-7, 8-10, and 11-13). The Clopper-Pearson 95% confidence interval for the rate of subjects will be calculated.

Number of days per cycle and reference period with scheduled bleeding, and unscheduled

bleeding or spotting will be analyzed descriptively for Group 1A and for relevant subgroups.
Number and duration of bleeding or spotting episodes will be summarized by treatment cycle and reference periods for Group 1A.
If scheduled bleeding starts in Cycle X but ends in Cycle (X+1) then bleeding episode will be assigned to Cycle X. Any bleeding or spotting that occurs during cycle Days (1-8) of the first treatment cycle and lasts up to 8 consecutive bleeding/spotting days will also be considered as scheduled bleeding days. Cycles without consecutively missing diary entries and with less than five non-consecutive missing diary entries will be used in the bleeding pattern analysis.

11.5 OTHER SIGNIFICANT ADVERSE EVENTS

AEs leading to dropout are those AEs associated with treatment discontinuation as captured on the CRFs. Incidence of AEs leading to dropouts will be tabulated, and subjects who discontinued because of AEs will be listed for Group 1A.

Sponsor: Exeltis USA, Inc. Analysis: Integrated Summary of Safety (ISS)
Product: LF111 (drospirenone 4 mg tablets) NDA 211367 Page 22
Statistical Analysis Plan June 15, 2018

The following categories of AEs of special interest are identified:
1. Thromboembolic events
2. Hyperkalemia or elevated blood potassium

The number and percentage of subjects with AEs of special interest will be summarized by preferred term for each category for Group 1 (1A and 1B) and, as appropriate, for the relevant subgroups. Subjects with these AEs will be listed by study for Group 1. AEs of special interest by exposure to IMP will be summarized for Group 1A.
The preferred terms included in each category are listed in Table 11.5–1.

Table 11.5–1. Preferred Terms for AE Categories of Special Interest

Category	Preferred Terms
Thromboembolic events (Venous thromboembolism and arterial thromboembolism)	Axillary vein thrombosis, Deep vein thrombosis, FemoraI artery embolism, Hypothenar hammer syndrome, Iliac artery embolism, Jugular vein thrombosis, Pelvic venous thrombosis, Peripheral artery thrombosis, Peripheral embolism, Subclavian artery embolism, Subclavian artery thrombosis, Suhclavian vein thrombosis, Thrombophlebitis, Thrombophlebitis setic, Thrombophlebitis superficial, Thrombosis corpora cavernosa, Venous thrombosis limb, Basilar artery thrombosis, Carotid arterial embolus, Carotid artery thrombosis, Cavernous sinus thrombosis, Cerebellar artery thrombosis, Cerebellar embolism, Cerebral artery embolism, Cerebral artery thrombosis, Cerebral microembolism, Cerebral thrombosis, Cerebral venous thrombosis, Cerebrospinal thrombotic tamponade, Embolic cerebral infarction, Embolic stroke, lntracranial venous sinus thrombosis, Superior sagittal sinus thrombosis, Thrombotic cerebral infarction, Thrombotic stroke, Transverse sinus thrombosis, Vertebral artery thrombosis, Pulmonary artery thrombosis, Pulmonary embolism, Pulmonary microemboli, Pulmonary thrombosis, Pulmonary venous thrombosis, Renal artery thrombosis, Renal embolism, Renal vascular thrombosis, Renal vein embolism, Renal vein thrombosis, Retinal artery embolism, Retinal artery thrombosis, Retinal vascular thrombosis, Retinal vein thrombosis.
Hyperkalemia/Elevated blood potassium	Blood potassium abnormal, Blood potassium increased, Hyperkalemia/Hyperkalaemia.

Sponsor: Exeltis USA, Inc. Analysis: Integrated Summary of Safety (ISS)
Product: LF111 (drospirenone 4 mg tablets) NDA 211367 Page 23

12.0 CLINICAL LABORATORY EVALUATIONS

Descriptive statistics (n, mean, standard deviation, minimum, median, and maximum) for quantitative clinical laboratory values at baseline, end of treatment period, and change from baseline will be summarized for Group 1A. Refer to Section 5.4.3 for the definition of *Baseline* and *End of treatment.* All clinical laboratory results will be presented in SI units.

Clinical laboratory parameters in the analysis will be as follows:

- **Hematology:** Haemoglobin, red blood cell (RBC) count, mean corpuscular volume (MCV), haematocrit, mean corpuscular/cellular haemoglobin (MCH), white blood cell (WBC) count, differential white blood cell count including neutrophils, lymphocytes, eosinophils, basophils and monocytes, and platelet count.
- **Chemistry:** Sodium, potassium, chloride, creatinine, blood urea nitrogen (BUN), calcium, glucose, total proteins, albumin, total cholesterol (high-density lipoprotein [HDL], lowdensity lipoprotein [LDL] cholesterol), triglycerides, gamma glutamyl transferase (GGT), total and direct bilirubin, alkaline phosphatase (ALP), alanine aminotransferase (ALT), aspartate aminotransferase (AST), creatine kinase (CK) and lactate dehydrogenase (LDH).
- **Urinalysis:** Leukocytes, nitrites, protein, glucose, ketones, blood, pH, urobilinogen, bilirubin, haemoglobin.

For some parameters in the list above, Study 205 did not assess or collect them. Relevant analysis presentations (i.e., tables/listings/figures) will show what studies are included.

Clinical laboratory values are potentially clinically significant (PCS) if they meet either the lower or upper PCS criteria listed in **Table 12–1**. For the parameters listed in **Table 12–1**, the corresponding PCS tables will present the number and percentage of subjects with PCS high values and with PCS low values separately for Group 1A. The number and percentage of subjects with PCS postbaseline values in the treatment period will be summarized. The percentages will be calculated relative to the number of subjects with available non-PCS baseline values and at least 1 postbaseline assessment in the treatment period. The numerator will be the total number of subjects with available non-PCS baseline values and at least 1 PCS postbaseline value in the treatment period.

Supportive listings of subjects with PCS values during the treatment period will be provided for Group 1A. The listings will include the study number, subject number, study center, and baseline and postbaseline values. The listings of all AEs for subjects with PCS values during the treatment period will also be provided for Group 1A.

A shift table will be presented for each parameter in Table 12-1 to identify any postbaseline changes between categories “Normal” and “Abnormal” values.

The number and percentage of subjects with hepatic laboratory parameter values of clinical interest (Hy’s law analysis) will also be tabulated:

- ALT or AST ≥ 3× ULN with total bilirubin ≥ 2× ULN and alkaline phosphatase < 2× ULN

Table 12–1. Criteria for Potentially Clinically Significant Laboratory Tests for Selected parameters

SI Units Traditional Units PCS Criteria *a* ***PCS Criteria***
Low Values

a ***High***
Values
ALT U/L U/L — ≥ 3 × UNL
AST U/L U/L — ≥ 3 × UNL
GGT U/L U/L — ≥ 2 × UNL
Creatinine μmol/L mg/dL — > 1.3 × UNL
Potassium mmol/L mEq/L < 0.9 × LNL > 1.1 × UNL
Total bilirubin μmol/L mg/dL — > 1.5 × UNL
Note: a Criteria refer to SI units.
LNL = lower normal limit of laboratory reference range; PCS = potentially clinically significant; SI = *Le Système International d'Unités* (International System of Units); UNL = upper normal limit of laboratory reference range.
Sponsor: Exeltis USA, Inc. Analysis: Integrated Summary of Safety (ISS)
Product: LF111 (drospirenone 4 mg tablets) NDA 211367 Page 26
Statistical Analysis Plan June 15, 2018

13.0 VITAL SIGNS

Descriptive statistics (n, mean, standard deviation, minimum, median, and maximum) for vital signs (e.g., systolic and diastolic blood pressure, pulse rate, and weight) at the baseline and at the end of treatment period will be presented for Group 1A. Refer to **Section 5.4.3** for the definition of *Baseline* and *End of treatment period.*
Vital sign values will be defined as PCS if they meet both the observed-value criteria and the change-from-baseline criteria listed in **Table 13–1**. The number and percentage of subjects with PCS postbaseline values will be summarized for Group 1A. The percentages will be calculated relative to the number of subjects with nonmissing baseline and at least one postbaseline assessment in the specific treatment period. The numerator will be the total number of subjects with nonmissing baseline and at least one PCS postbaseline value in the specific treatment period. Supportive listings of subjects with PCS values during the treatment period will be provided for Group 1A. The listings will include the study number, subject number, study center, and baseline and postbaseline values. The listings of all AEs for subjects with PCS values will also be provided.

Table 13–1. Criteria for Potentially Clinically Significant Vital Signs
Vital Sign Parameter Flag
Criteria*a*
Observed Value Change From Baseline
Systolic blood
pressure, mm Hg (Supine)
High ≥ 180 Increase of ≥ 20
Low ≤ 90 Decrease of ≥ 20
Diastolic blood
pressure, mm Hg (Supine)
High ≥ 105 Increase of ≥ 15
Low ≤ 50 Decrease of ≥ 15
Pulse rate, bpm (Supine)
High ≥ 120 Increase of ≥ 15
Low ≤ 50 Decrease of ≥ 15
Weight, kg
High — Increase of ≥ 7%
Low — Decrease of ≥ 7%
Note: a A postbaseline value will be considered a PCS value if it meets criteria for both observed value and change from baseline.
Sponsor: Exeltis USA, Inc. Analysis: Integrated Summary of Safety (ISS)
Product: LF111 (drospirenone 4 mg tablets) NDA 211367 Page 27

14.0 SAFETY IN SPECIAL GROUPS AND SITUATIONS

The drug-demographic interactions for Group 1A will be explored by subgroup safety analyses based on the following demographic and baseline factors:

• Age group (≤ 35, > 35 years)

• BMI (< 30, ≥ 30 kg/m2

• Blood Pressure (SBP < 130 mmHg and DBP < 85 mmHg, SBP ≥ 130 mmHg or DBP ≥ 85 mmHg)

)

• Smoking (Current smoker, Ex-smoker, Never)

• Alcohol Use (Drinker and Never)

For the corresponding subgroup analyses of exposure to IMP, AEs, refer to **Section 8.0** (Overall Extent of Exposure to IMP) and **Section 11.0** (Adverse Experience in Clinical Studies). If the incidence of SAEs or AEs of special interest is low (<1%), its subgroup analysis will not be performed.

Due to the small number of subjects in Group 1B (study CF111/304), the above subgroup analyses will not be performed.

15.0 COMPUTER METHODS

Statistical analyses will be performed using version 9.3 (or newer) of SAS on a server system.

16.0 DATA HANDLING CONVENTIONS

16.1 VISIT TIME WINDOWS

No visit window will be assigned in the pooled analysis.

16.2 MISSING DATA HANDLING

16.2.1 Missing Date of IMP

Unless otherwise specified, if the date of the last dose of IMP is missing for a subject in the Safety Set, then last date of IMP administration recorded in subject’s diary will be used to impute the date of last dose for the purpose of computing the treatment duration for the summary tables of treatment duration. If last date of IMP administration recorded in subject’s diary is not available and subject did not return all IMP pills, the subject's last visit date will be taken into account. In addition, a footnote will be added to indicate such an imputation in the summary tables of treatment duration, but the observed date will be presented in data listings.

16.2.2 Missing Severity Assessment for Adverse Events

AEs severity will be defined as “Unknown” if severity assessment is missing, whereas the actual values will be presented in data listings.

16.2.3 Missing Relationship to IMP for Adverse Events

The relationship of an AE to the IMP will be defined as “Unknown” if relationship grade assessment is missing, whereas the actual values will be presented in data listings.

16.2.4 Missing Date Information for Adverse Events

The following imputation algorithm for incomplete start dates for AEs will be applied to the randomized, double-blind controlled studies and their extension studies if any. The imputed value will be used for summaries, whereas the actual observed value will be presented in data listings.

Missing day and month

• If the year of the incomplete start date is the same as the year of the date of the first dose of IMP, then the day and month of the date of the first dose of IMP will be assigned to the missing fields
• If the year of the incomplete start date is before the year of the date of the first dose of IMP, then December 31 will be assigned to the missing fields
• If the year of the incomplete start date is after the year of the date of the first dose of IMP, then January 1 will be assigned to the missing fields

Missing month only
• The day will also be treated as missing, and both month and day will be replaced according to the above procedure
Missing day only
• If the month and year of the incomplete start date are the same as the month and year of the date of the first dose of IMP, then the day of the date of the first dose of IMP will be assigned to the missing field
• If the year of the incomplete start date is before the year of the date of the first dose of IMP or if both years are the same but the month of the incomplete start date is before the month of the date of the first dose of IMP, then the last day of the month will be assigned to the missing field
• If the year of the incomplete start date is after the year of the date of the first dose of IMP or if both years are the same but the month of the incomplete start date is after the month of the date of the first dose of IMP, then the first day of the month will be assigned to the missing field
If the stop date is complete and the imputed start date, when imputed as instructed above, is after the stop date, the start date will be imputed to equal the stop date.
16.2.5 Missing Date Information for Prior or Concomitant Medications
For prior or concomitant medications, incomplete (ie, partially missing) start dates and/or stop dates will be imputed. When the start date and the stop date are both incomplete for a subject, the start date will be imputed first. The imputed value will be used for summaries, whereas the actual observed value will be presented in data listings.
Incomplete Start Date
The following rules will be applied to impute the missing numeric fields. If the stop date is complete and the imputed start date is after the stop date, then the start date will be imputed to equal the stop date.
Missing day and month
• If the year of the incomplete start date is the same as the year of the date of the first dose of IMP, then the day and month of the date of the first dose of IMP will be assigned to the missing fields
• If the year of the incomplete start date is before the year of the date of the first dose of IMP, then December 31 will be assigned to the missing fields
• If the year of the incomplete start date is after the year of the date of the first dose of IMP, then January 1 will be assigned to the missing fields

Missing month only
• The day will also be treated as missing, and both month and day will be replaced according to the above procedure
Missing day only
• If the month and year of the incomplete start date are the same as the month and year

of the date of the first dose of IMP, then the day of the date of the first dose of IMP will be assigned to the missing field

• If the year of the incomplete start date is before the year of the date of the first dose of IMP or if both years are the same but the month of the incomplete start date is before the month of the date of the first dose of IMP, then the last day of the month will be assigned to the missing field

• If the year of the incomplete start date is after the year of the date of the first dose of IMP or if both years are the same but the month of the incomplete start date is after the month of the date of the first dose of IMP, then the first day of the month will be assigned to the missing field

Incomplete Stop Date

For the purpose of deriving concomitant medication flag, the following rules will be applied to impute the missing numeric fields. If the date of the last dose of the IMP is missing, then it will be replaced with the last visit date. If the imputed stop date is before the start date (imputed or nonimputed start date), then the imputed stop date will be imputed to equal the start date.

Missing day and month

• If the year of the incomplete stop date is the same as the year of the date of the last dose of IMP, then the day and month of the date of the last dose of IMP will be assigned to the missing fields

• If the year of the incomplete stop date is before the year of the date of the last dose of IMP, then December 31 will be assigned to the missing fields

• If the year of the incomplete stop date is after the year of the date of the last dose of IMP, then January 1 will be assigned to the missing fields

Missing month only

• The day will also be treated as missing, and both month and day will be replaced according to the above procedure

Sponsor: Exeltis USA, Inc. Analysis: Integrated Summary of Safety (ISS)
Product: LF111 (drospirenone 4 mg tablets) NDA 211367 Page 32
Statistical Analysis Plan June 15, 2018

Missing day only

• If the month and year of the incomplete stop date are the same as the month and year of the date of the last dose of IMP, then the day of the date of the last dose of IMP will be assigned to the missing field

• If the year of the incomplete stop date is before the year of the date of the last dose of IMP or if both years are the same but the month of the incomplete stop date is before the month of the date of the last dose of IMP, then the last day of the month will be assigned to the missing field

• If the year of the incomplete stop date is after the year of the date of the last dose of IMP or if both years are the same but the month of the incomplete stop date is after the month of the date of the last dose of IMP, then the first day of the month will be assigned to the missing field

Sponsor: Exeltis USA, Inc. Analysis: Integrated Summary of Safety (ISS)
Product: LF111 (drospirenone 4 mg tablets) NDA 211367 Page 33
Statistical Analysis Plan June 15, 2018

17.0 REFERENCES

US Food and Drug Administration. Center for Drug Evaluation and Research. Guideline for the format and content of the clinical and statistical sections of an application. 1988 Jul. Available from: http://www.fda.gov/downloads/Drugs/GuidanceComplianceRegulatoryInformation/Guidances/UCM071665.pdf. Accessed 2018 March 8.

US Food and Drug Administration. Center for Drug Evaluation and Research. Guidance for industry M4E: the CTD—efficacy. 2001 Aug. Available from:

http://www.fda.gov/downloads/RegulatoryInformation/Guidances/UCM129865.pdf. Accessed 2018 March 8.
US Food and Drug Administration. Center for Drug Evaluation and Research. Conducting a clinical safety review of a new product application and preparing a report on the review. 2005 Feb. Available from: http://www.fda.gov/downloads/Drugs/GuidanceComplianceRegulatoryInformation/Guidances/UCM072974.pdf. 2018 March 8.

13. DISCUSSION AND OVERALL CONCLUSION

13.1 Discussion

This Phase III multicentre, double-blind, double-dummy trial investigated the contraceptive efficacy, tolerability and safety of drospirenone 4.0 mg as LF111 over 9 treatment cycles in comparison with desogestrel 0.075 mg. The trial was conducted at 73 trial centres in Europe.

A total of 1365 subjects were screened, 1213 subjects were randomised. Of these, 1190 subjects received randomised trial medication: 858 received drospirenone 4.0 mg in a regimen of 24 verum/4 placebo and 332 subjects received desogestrel 0.075 mg in a 28/0 regimen. All 1190 treated subjects were included in the Safety Set and the Full Analysis Set.

The primary efficacy variable was the overall Pearl Index. A total of 6691 drospirenone and 2487 desogestrel treatment cycles were analysed. During these cycles five Test group and one Reference group subjects became pregnant, all pregnancies were classified as method failure. The contraceptive efficacy of drospirenone in a regimen 24/4 was reflected in an overall Pearl Index of 0.9715, 95% CI of 0.3154-2.2671.

All six on-treatment pregnancies were reported for subjects aged 35 years or younger. The overall PI (95% CI) for women ≤ 35 years in the Test group was 1.2428 (0.4035; 2.9004).

Secondary efficacy analyses included overal PI after correction for additional contraception and sexual activity status, method failure PI and pregnancy ratio. The respective PIs varied from 1.0875 to 1.4006 (age group ≤ 35 years: 1.4000 to 1.8351) in the Test group.

The cumulative 9-cycle pregnancy ratio (95% CI) in the Test group was 0.70% (0.09; 1.31). For the age subgroup ≤ 35 years, it was 0.90% (0.11; 1.68).

Pooled efficacy analysis of CF111/301 and CF111/302 trials

Following the CHMP Guideline on Clinical Investigation of Steroid Contraceptives in Women [5] the number of cycles collected should be at least large enough to give the overall Pearl Index (PI) with a 95% confidence interval such that the difference between the upper limit of the confidence interval and the point estimate does not exceed 1. The data from two studies (CF111/301 and this study) were pooled for the calculation of the overall PI.

For an assumed PI < 1.0 the number of cycles needed to fulfil this precision requirement with a 90% power was 12337. The analysed number of evaluable cycles in both trials was 14329. Eight pregnancies occured during the treatment and all of them were assessed as being method failure. The overall PI (95% CI) was: 0.7258 (0.3133; 1.4301) calculated for all women and of 0.9332 (0.4029; 1.8387) for women aged 35 years or younger (number of cycles: 11145).

The PI (95% CI) after correction for additonal contraception and sexual activity status as well as method failure PI, calculated for FAS, were below „1.0“, too: 0.7898 (0.3410; 1.5562) and 0.9682 (0.4180; 1.9077). The respective PIs calculated for subjects ≤ 35 years were slightly higher: 1.0223 (0.4414; 2.0144) and PI of 1.0785 (0.4656; 2.1251).

The cumulative 13-cycle pregnancy ratio (95% CI) of DRSP users (FAS) in both trials was 0.72 (0.17-1.27), and that for the age subgroup ≤ 35 years was 0.93 (0.21-1.64).

Tolerability and safety

The tolerability assessments in this trial were based on the vaginal bleeding pattern.

First of all it should be noted that the regimen of both contraceptives used in this trial was different: drospirenone was administered for 24 days followed by a 4-day hormone-free interval, whereas desogestrel was administered for 28 days without any interval. Therefore

subjects who received drospirenone experienced both scheduled and unscheduled bleeding, whereas the users of desogestrel experienced unscheduled bleeding only.

The proportion of subjects with unscheduled bleeding and spotting during Cycles 2-6 was lower in the Test group (73.0%) than in the Reference group (88.4%), with the difference (95% CI) of -15.39% (-21.78%; -8.99%) between the groups. Since the two-sided 95% CI lies entirely to the left of the defined non-inferiority margin of 9%, the Test group is non inferior to the Reference group. Moreover, since 95% CI not only lies entirely below 9% but also below zero, superiority in terms of statistical significance at the 5% level ($p < 0.05$) was concluded.

During Cycles 2-9 bleeding or spotting was reported by 83.9% of the Test and 87.9% of the Reference group subjects, unscheduled bleeding by 79.7% vs. 87.9%. Spotting was more common than bleeding. The following trends were observed over time: The incidence of both overall and unscheduled bleeding and spotting decreased in both treatment groups, as well as the incidence of prolonged bleeding. The rate of prolonged bleeding was significantly lower in the test group as compared with the desogestrel group for cycles 5-7 and 7-9. In addition, during the first period the number of days of bleeding was lower in the test group.

The number of bleeding/spotting days decreased, as well as the number of bleeding/spotting episodes. At the same time the proportion of subjects who had no bleeding or spotting increased from 30.3% to 43.7% subjects in the Test and from 26.0% to 54.7% subjects in the Reference group. Taken together, the bleeding became lighter and shorter in both groups, with an increasing number of subjects reporting absence of bleeding.

Discontinuation rates due to abnormal bleeding were low: 3.3% for drospirenone and 6.6% for desogestrel users.

The AE profiles of drospirenone and desogestrel were comparable with no statistically significant differences between the groups with regard to the incidence of related, severe, serious TEAEs or TEAEs leading to premature discontinuation.

The frequency of treatment-emergent SAEs was low (2.3% of the Test and 2.1% of the Reference group subjects) and the vast majority of them were considered to be not related to IMP. In total two SAEs, hepatic adenoma in the Test and ectopic pregnancy in the Reference group, were assessed as being possibly related to trial treatment. The vast majority of TEAEs was of mild-to-moderate intensity. Overall, 9.6% of the Test and 13.3% of the Reference group subjects prematurely discontinued treatment due to TEAEs.

Though some changes over time and differences between the treatment groups were observed for several ECG parameters, all these changes were very mild and considered not clinically significant.

No relevant differences between the treatment groups or within each treatment group were observed in laboratory tests, including haemostatic parameters, carbohydrate metabolism and bone metabolism parameters and other safety analyses.

13.2 Overall Conclusion

The results of this trial show that the use of drospirenone 4.0 mg in a regimen 24 verum / 4 placebo over 9 treatment cycles provided effective contraceptive protection with an acceptable bleeding pattern. In particular, the treatment with drospirenone 4.0 mg in a regimen 24 verum / 4 placebo resulted in a lower frequency of unscheduled bleeding (i.e. provided better cycle control) and less prolonged bleeding than the treatment with desogestrel 0.075 mg in a 28/0 regimen. During Cycles 2-6, the treatment with drospirenone 4.0 mg in a

regimen 24 verum / 4 placebo was superior to the treatment with desogestrel 0.075 mg in a 28/0 regimen with regard to the proportion of subjects with unscheduled bleeding.

No major differences between the treatment groups were observed for safety analyses.

The data from two 9-cycle to 13-cycle trials showed that LF111 is an effective oral contraceptive with an overall Pearl Index (95% CI) of 0.7258 (0.3133; 1.4301).

14. REFERENCE LIST

1. Angeliq® product monograph.
2. NDA 21098 submitted to the Food and Drug Administration by Berlex Laboratories Inc., Medical Review completed 13 April 2001. http://www.accessdata.fda.gov/drugsatfda_docs/nda/2001/21-098_Yasmin_medr_P1.pdf (accessed May 2010)
3. Rosenbaum P, Schmidt W, Helmerhorst FM, Wuttke W, Rossmanith W, Freundl F, Thomas K, Grillo M, Wolf A, Heithecker R. Inhibition of ovulation by a novel progestogen (drospirenone) alone or in combination with ethinyl estradiol. Eur J Contracep Reprod Health Care 2000; 5: 16-24.
4. Investigator's Brochure, Drospirenone POP-LF111, León Farma, Version 5.0, 24 July *2012*.
5. Guideline on clinical investigation of steroid contraceptives in women, committee for medicinal products for human use (CHMP), 27 July 2005. EMEA/CPMP/EWP/519/98 Rev 1.
6. Milsom J, Korver T. Ovulation incidence with oral contraceptives: a literature review. J Fam Plann Reprod Health Care. 2008, 34(4): 237-246.
7. Collaborative group on the Desogestrel containing progestogen-only pill. A double-blind study comparing the contraceptive efficacy, acceptability and safety of two progestogen-only pills containing desogestrel 75 micrograms/day or levonorgestrel 30 micrograms/day. Eur J Contracept Health Care. 1998; 3:169-178.
8. Gerlinger C et al. Recommendation for confidence interval and sample size calculation for the Pearl Index. Eur J Contracept Reprod Health Care. 2003; 8:87-92.
9. Huber J, Foidart JM, Wuttke W, Merki-Feld GS, The HS, Gerlinger C, Schellschmidt I, Heithecker R: Efficacy and tolerability of a monophasic oral contraceptive containing ethinylestradiol and drospirenone. Eur J Contracep Repr 2000; 5:25-34.
10. Note for guidance on the clinical evaluation of QT/QTc interval prolongation and proarrhythmic potential for non-antiarrhythmic drugs, November 2005.
11. Belsey EM, Machin D, d'Arcangues C. The analysis of vaginal bleeding patterns induced by fertility regulating methods. World Health Organization special programme of Research, Development and Research Training in Human Reproduction. 1986; 34:253-260.
