# Supplementary material for: Bleeding profile of women using a drospirenone-only pill 4 mg over nine cycles in comparison with desogestrel 0.075 mg
Source: PLoS One. 2020 Jun 29;15(6):e0231856. doi: 10.1371/journal.pone.0231856 (PMC7323950; doi:10.1371/journal.pone.0231856)
Supplement: S2 File — (PDF) [file pone.0231856.s003.pdf]

PROTOCOL CF111/302: STATUS REPORT

DATE: 2014-06-27 FINAL

Site No.	City	Investigator	Participation		Central/Leading EC		Local EC		Authorities		Agreement	SIV	First patient in	Planned No of Pat	Site closed	Comments
			Yes	No	submission	approval	submission	approval	submission	approval	signed	Date	Date		Date	
Austria (151-153)																
151	Vienna	Egarter	X (F)		10.02.2012; 10.07.2012 (AT amend)	27.03.2012	N/A (Leading EC)	N/A	13.02.2012; 10.07.2012 (AT amend)	19.03.2012	yes	16.10.2012	23.01.2013	30	03.06.2014	Amend AT non substantial - no approval required
152	Graz	Lang	(X (F))				13.02.2012	N/A			yes	12.09.2012	N/A	17	07.05.2013	
153	Linz	Oppelt	(X)				13.02.2012	N/A			yes	05.09.2012	N/A	17	22.04.2013	
Czech (251-266)																
251	Hradec Kralove	Kalousek	X		23.01.2012; 23.03.2012 (CZ Amend)	04.04.2012 (incl. CZ amend)	23.02.2012, 23.03.2012 (CZ	22.03.2012; 19.04.2012 (CZ	19.01.2012; 28.03.2012 (CZ amend)	04.04.2012 (incl. CZ Amend)	yes	13.08.2012	04.09.2012	22	30.05.2014	
252	Plzen	Svec	X				N/A (same as MEC)	N/A (same as MEC)			yes	08.08.2012	27.08.2012	30	20.05.2014	
253	Brno	Huvar	X				23.02.2012, 23.03.2012 (CZ Amend)	04.04.2012 (incl. CZ amend)			yes	01.10.2012	07.11.2012	20	21.05.2014	
254	Prague	Horcicka	X				N/A (same as MEC)	N/A (same as MEC)			yes	15.08.2012	30.10.2012	17	26.05.2014	
255	Prague	Tesar	X				N/A (same as MEC)	N/A (same as MEC)			yes	25.07.2012	11.09.2012	20	10.06.2014	
256	Ostrava	Rozbroj	X				23.02.2012, 23.03.2012 (CZ Amend)	29.03.2012; 03.05.2012 (CZ amend)			yes	03.08.2012	28.08.2012	20	23.05.2014	
257	Prague	Hynkova	X				N/A (same as MEC)	N/A (same as MEC)			yes	16.07.2012	21.08.2012	20	28.05.2014	
258	Nachod	Kestranek		X			same as 251	same as 251			N/A	N/A	N/A	18	N/A	
259	Olomouc	Skrivanek	X				23.02.2012, 23.03.2012 (CZ Amend)	16.04.2012 (incl. CZ amend)			yes	24.07.2012	13.08.2012	20	12.06.2014	
260	Pisek	Hlavackova	X				N/A (same as MEC)	N/A (same as MEC)			yes	31.07.2012	27.08.2012	20	04.06.2014	
261	Brno	Dvorak	X		05.10.2012	17.10.2012	05.10.2012	14.11.2012	08.10.2012	notification only (silent approval after 30 days = 08.11.2012)	yes	12.11.2012	12.12.2012	20	16.06.2014	
262	Ceske Budejovice	Horejsi	X				N/A (same as MEC)	N/A (same as MEC)			yes	07.11.2012	29.11.2012	20	05.06.2014	
263	Prague	Jenicek	X				N/A (same as MEC)	N/A (same as MEC)			yes	31.10.2012	14.11.2012	20	09.06.2014	
264	Prague	Stara	X				N/A (same as MEC)	N/A (same as MEC)			yes	08.11.2012	19.11.2012	10	04.06.2014	
265	Pardubice	Sykorova	X				N/A (same as MEC)	N/A (same as MEC)			yes	14.11.2012	13.12.2012	15	02.06.2014	
266	Prague	Vetesnikova-Koubova	X				N/A (same as MEC)	N/A (same as MEC)			yes	19.11.2012	29.11.2012	20	30.05.2014	
Germany (351-372)																
351	Berlin	Maar	X (F)		22.12.2011 (except sites 355, 366, 369), 03.01.2012 for sites 355, 366; 24.02.2012 (DE 1 Amend); 11.07.2012 (DE 2 Amend)	30.03.2012 (incl. DE 1 Amend)	N/A (Leading EC)	N/A	22.12.2011 (except sites 355, 366, 369), 05.01.2012 (site 355, 366); 24.02.2012 (DE 1 Amend); 11.07.2012 (DE 2 Amend)	09.02.2012; 16.03.2012 (DE 1 Amend)	yes	13.07.2012	01.08.2012	25	20.05.2014	Amend 2 DE non substantial - no approval required
352	Munich	Alberti	(X)				22.12.2011	N/A			yes	07.08.2012	N/A	17	20.02.2013	
353	Hamburg	Bühling	X (F)				22.12.2011	N/A			yes	01.08.2012	01.11.2012	20	26.05.2014	
354	Leipzig	Burkghardt	X (F)				22.12.2011	N/A			yes	29.08.2012	10.09.2012	17	24.06.2014	
355	Berlin	Dodt		X (F)			03.01.2012	N/A			N/A	N/A	N/A	N/A	N/A	
356	Essen	Gerlach	(X(F))				22.12.2011	N/A			yes	09.08.2012	N/A	30	25.01.2013	
357	Gesecke	Göckler-Leopold	(X)				22.12.2011	N/A			yes	08.08.2012	N/A	20	05.03.2013	
358	Hannover	Greven	X (F)				22.12.2011	N/A			yes	15.08.2012	28.08.2012	17	19.05.2014	
359	Mannheim	Knauss	(X (F))				22.12.2011	N/A			yes	22.08.2012	N/A	14	18.12.2012	
360	Munich	Kränzlin	X (F)				22.12.2011	N/A			yes	27.03.2012	06.08.2012	20	21.05.2014	
361	Hamburg	Kressin	X (F)				22.12.2011	N/A			yes	21.08.2012	16.10.2012	30	20.05.2014	
362	Hamburg	Peters	X (F)				22.12.2011	N/A			yes	14.08.2012	03.09.2012	17	27.05.2014	
363	Eppelheim	Royar	(X)				22.12.2011	N/A			yes	03.08.2012	N/A	10	01.02.2013	
364	Berlin	Ruhmland	(X(F))				22.12.2011	N/A			yes	11.09.2012	N/A	30	06.03.2013	
365	Magdeburg	Ahrendt	X (F)				22.12.2011	N/A			yes	30.10.2012	13.11.2012	25	27.05.2014	
366	Berlin	Büchau	X				03.01.2012	N/A			yes	07.11.2012	14.11.2012	17	03.06.2014	
367	Giessen	Cassel		X			22.12.2011	N/A			yes	N/A	N/A	N/A	N/A	
368	Freising	Dilenge		X			22.12.2011	N/A			yes	N/A	N/A	N/A	N/A	
369	Weinstadt	Schröter		X			N/A	N/A			N/A	N/A	N/A	N/A	N/A	
370	Frankfurt	Göttker-Schnetmann	X (F)				22.12.2011	N/A			yes	19.09.2012	04.10.2012	17	13.05.2014	
371	Schriesheim	Hoppe	X (F)				22.12.2011	N/A			yes	12.09.2012	15.10.2012	17	08.05.2014	
372	Berlin	Schlothauer		X			22.12.2011	N/A			yes	N/A	N/A	N/A	N/A	
Hungary (451-464)																
451	Budapest	Spanik	X		13.01.2012	29.02.2012 (forwarded by EC to the CA)			13.01.2012	26.03.2012 (includes EC vote)	yes	07.08.2012	06.09.2012	20	20.05.2014	
452	Hatvan	Zambo	X								yes	16.08.2012	07.09.2012	19	14.05.2014	
453	Budapest	Langmar	X								yes	09.08.2012	23.08.2012	20	13.05.2014	
454	Eger	Hernadi	X								yes	15.08.2012	20.09.2012	21	21.05.2014	
455	Budapest	Szönyi	X								yes	24.07.2012	27.08.2012	20	12.05.2014	
456	Budapest	Urban		X							yes	N/A	N/A	N/A	N/A	
457	Tatabanya	Pödör	X								yes	14.08.2012	18.09.2012	23	28.05.2014	
458	Budapest	Gyarmati	(X)								yes	06.08.2012	N/A	23	30.11.2012	
459	Budapest	Szekely/Horvath	X								yes	25.08.2012	16.09.2012	19	02.06.2014	
460	Szikszó	Szentpeteri	X		30.10.2012	15.12.2012 (forwarded by EC to the CA)			30.10.2012	04.01.2013	yes	04.01.2013	18.01.2013	13	07.05.2014	
461	Budapest	Molnar	X								yes	20.12.2012	15.01.2013	18	26.05.2014	
462	Budapest	Novak	X								yes	21.12.2012	21.01.2013	15	14.05.2014	
463	Budapest	Szekely	X								yes	21.12.2012	15.01.2013	15	02.06.2014	
464	Budapest	Palmai	X								yes	18.12.2012	14.01.2013	13	15.05.2014	

Site No.	City	Investigator	Participation		Central/Leading EC		Local EC		Authorities		Agreement	SIV	First patient in	Planned No of Pat	Site closed	Comments
		Poland (551-568)														
551	Krakow	Jaszczynski	X								yes	31.08.2012	13.09.2012	18	17.06.2014	
552	Katowice	Faska	(X)								yes	24.08.2012	N/A	17	05.02.2013	
553	Warsaw	Wisniewska-Sawicki	X								yes	20.07.2012	06.08.2012	17	23.05.2014	
554	Warsaw	Bojar	(X)								yes	21.08.2012	N/A	17	04.03.2013	
555	Katowice	Chelmickci	X								yes	02.08.2012	27.11.2012	18	12.06.2014	
556	Warsaw	Jakimiuk	X								yes	26.07.2012	20.08.2012	17	05.06.2014	
557	Lodz	Pakalski	X		30.01.2012; 29.05.2012 (site 561, PI change site 559)	08.02.2012, 04.06.2012 (site 561, PI change site 559)			23.01.2011, 25.05.2012 (site 561, PI change site 559)	25.03.2012, 04.07.2012 (site 561, PI change site 559)	yes	04.09.2012	08.01.2013	17	07.05.2014	
558	Lodz	Jedrzejczyk	X								yes	07.08.2012	08.08.2012	16	20.05.2014	
559	Lodz	Kowalska (formerly Pieta-Dolinskaa)	X								yes	25.07.2012	16.08.2012	17	11.06.2014	
560	Kielce	Adamczyk-Gruszka	X								yes	01.08.2012	08.08.2012	30	06.05.2014	
561	Lodz	Malinowski	(X)								yes	25.10.2012	N/A	17	14.03.2013	
562	Mikolow	Skrzypulek-Plinta	X								yes	26.07.2012	25.09.2012	30	04.06.2014	
563	Krakow	Nawara-Braun	X								yes	29.03.2012	01.08.2012	17	28.05.2014	
564	Warsaw	Brankowska	(X)						30.10.2012	05.12.2012	yes	27.11.2012	N/A	20	05.03.2013	
565	Lodz	Jedrzejczyk	X								yes	29.11.2012	05.12.2012	13	21.05.2014	
566	Lodz	Polac	X		07.11.2012	14.11.2012			08.10.2012	13.11.2012	yes	14.11.2012	28.11.2012	13	07.05.2014	
567	Krakow	Radecka	X								yes	14.11.2012	23.11.2012	13	08.05.2014	
568	Bialystok	Tomaszewski	X								yes	15.11.2012	16.11.2012	23	20.05.2014	
		Romania (651-662)														
651	Bucharest	Manescu		X		N/A					yes	N/A	N/A	28	N/A	
652	Bucharest	Cornea		X		03.10.2012					yes	N/A	N/A	N/A	N/A	
653	Bucharest	Dumitrascu	X								yes	23.11.2012	13.12.2012	30	03.06.2014	
654	Craiova	Gheorman		X		N/A					yes	N/A	N/A	30	N/A	
655	Bucharest	Tutunaru	X		25.01.2012	03.10.2012			18.01.2012	25.06.2012	yes	13.11.2012	14.12.2012	30	26.05.2014	
656	Ploiesti	Munteanu		X		not given					yes	N/A	N/A	N/A	N/A	
657	Brasov	Gogonea	X			03.10.2012					yes	15.11.2012	26.11.2012	30	30.05.2014	
658	Bucharest	Rotaru	X								yes	14.11.2012	27.11.2012	30	22.05.2014	
659	Constanta	Costea	back-up			not given				not given	yes	back-up	N/A	N/A	N/A	
660	Popesti-Leordeni	Gheorghiu	back-up		N/A	N/A			N/A	N/A	yes	back-up	N/A	N/A	N/A	
661	Bucharest	Moraru	back-up		25.01.2012	03.10.2012			18.01.2012	25.06.2012	yes	back-up	N/A	N/A	N/A	
662	Bucharest	Edu	back-up		N/A	N/A			N/A	N/A	yes	back-up	N/A	N/A	N/A	
		Spain (751-762)														
751	Madrid	Palacios	X				N/A (Reference EC)	N/A			yes	23.07.2012	26.09.2012	23	09.06.2014	
752	Gava	Sanchez-Borrego	X				01.02.2012	N/A			yes	25.09.2012	07.11.2012	20	12.06.2014	
753	Cerdanyola	Sanchez-Borrego	X				see 752	N/A			yes	25.09.2012	28.11.2012	20	12.06.2014	
754	Palma de Mallorca	Jimenez		X			01.02.2012	N/A			yes	N/A	N/A	N/A	N/A	
755	Madrid	Gonzalez	X				N/A (Reference EC)	N/A			yes	22.08.2012	30.10.2012	18	10.06.2014	
756	Guadalajara	De La Viuda	(X)		01.02.2012	12.04.2012	01.02.2012	N/A	21.02.2012	25.04.2012	yes	15.11.2012	N/A	20	11.02.2013	
757	Girona	Alvarez	(X)				see 752	N/A			yes	15.10.2012	N/A	20	20.02.2013	
758	Barcelona	Cereto	(X)				01.02.2012	N/A			yes	02.08.2012	N/A	25	31.01.2013	
759	Vic	Grau	X				01.02.2012	N/A			yes	21.09.2012	08.10.2012	25	29.05.2014	
760	Terrassa	Pessarrodona	X				01.02.2012	N/A			yes	02.08.2012	29.11.2012	20	26.05.2014	
761	St Cugat	Balaguer	X				01.02.2012	N/A			yes	24.07.2012	27.11.2012	20	14.05.2014	
762	Manresa	Bergos	X				see 752	N/A			yes	17.09.2012	28.12.2012	20	19.06.2014	
		Slovakia (851-860)														
851	Martin	Danko	X				N/A (MEC)	N/A			yes	06.08.2012	16.10.2012	20	23.05.2014	
852	Bratislava	Cupanik	X				27.01.2012	24.02.2012			yes	07.08.2012	10.10.2012	23	14.05.2014	
853	Banska Bystrica	Bielik	(X)				27.01.2012	12.03.2012			yes	15.08.2012	N/A	17	21.02.2013	
854	Banska Bystrica	Petrovicova	X				see 853	see 853			yes	07.08.2012	24.09.2012	20	02.06.2014	
855	Bratislava	Sladicekova	X		27.01.2012	11.04.2012	27.01.2012	07.03.2012	25.01.2012	05.03.2012	yes	18.09.2012	08.11.2012	17	14.05.2014	
856	Rinavska Sobota	Bachnakova		X			see 853	see 853			N/A	N/A	N/A	N/A	N/A	
857	Martin	Biringer	X				N/A (MEC)	N/A			yes	06.08.2012	16.10.2012	20	23.05.2014	
858	Poprad	Fathlova	X				27.01.2012	07.02.2012			yes	10.08.2012	22.08.2012	15	20.06.2014	
859	Martin	Gatova	X		09.10.2012	07.12.2012	N/A (MEC)	N/A	09.10.2012	18.12.2012	yes	15.11.2012	08.01.2013	10	24.06.2014	
860	Banska Bystrica	Sutekova	X				09.10.2012	12.11.2012			yes	13.11.2012	14.01.2013	8	11.06.2014	

National Coordinator (only AT, DE, PL, ES)
X (F) Freezer -20°C (only appl. DE, AT)
