# Supplementary material for: Bleeding profile of women using a drospirenone-only pill 4 mg over nine cycles in comparison with desogestrel 0.075 mg
Source: PLoS One. 2020 Jun 29;15(6):e0231856. doi: 10.1371/journal.pone.0231856 (PMC7323950; doi:10.1371/journal.pone.0231856)
Supplement: S3 File — (PDF) [file pone.0231856.s004.pdf]

| ETHICS COMMITTEES CF111/302 | | | | | | | | | | |
|---|---|---|---|---|---|---|---|---|---|---|
| NO. | INVESTIGATOR | EC | Reference no. | CHAIRMAN | POST CODE | CITY | STREET / NUMBER | TEL NUMBER | FAX NUMBER | EMAIL |
| | Austria | | | | | | | | | |
| 151 | Egarter | see leading ethics committee | | | | | | | | |
| 152 | Lang | Ethikkommission der Medizinischen Universität Graz | 24-239 ex11/12 | Univ.Prof. DI Dr. Peter H. Rehak | 8036 | Graz | Auenbruggerplatz 2 | +43 (0316) 385-13928, -17407, -17408 | +43 (0316) 385-14348 | ethikkommission@medunigraz.at |
| 153 | Oppelt | Ethikkommission des Landes Oberösterreich | G-106-12 | Univ.Prof. Prim. Dr. Johannes Fischer | 4020 | Linz | Wagner-Jauregg-Weg 15 | +43 (05) 055462-26091 | +43 (05) 055462-26094 | tina.gattinger@gespag.at |
| lead EC | | Ethikkommission der Medizinischen Universität Wien | EK 1066/2012 | Dr. Ernst Singer | 1090 | Wien | Borschkegasse 8b/6 | +43 (01) 40400-2147, -2248, -2241 | +43 (01) 40400-1690 | ethik-kom@meduniwien.ac.at |
| | Czech Republic | | | | | | | | | |
| 251 | Kalousek | Etická komise, Centrum neurologické péče, s.r.o. | EK 3/12 | MUDr. Radomír Štrupl | 516 01 | Rychnov nad Kněžnou | Jiráskova 1389 | +420 491 112 724 | +420 491 112 725 | helena@naurol.cz |
| 252 | Švec | see MEC | | | | | | | | |
| 253 | Huvar | Etická komise Fakultní nemocnice Brno | 65M,L-2011 | PharmDr. Šárka Kozáková | 625 00 | Brno | Jihlavská 20 | +420 532 232 798 | +420 547 211 961 | etickakomise@fnbrno.cz |
| 254 | Horčička | see MEC | | | | | | | | |
| 255 | Tesař | see MEC | | | | | | | | |
| 256 | Rozbroj | Etická komise Fakultní nemocnice Ostrava | 151/2012 | MUDr. Luděk Rožnovský, CSc. | 708 52 | Ostrava-Poruba | 17. listopadu 1790 | +420 597 372 542 | +420 597 374 801 | eticka.komise@fno.cz |
| 257 | Hynková | see MEC | | | | | | | | |
| 258 | Kestřánek | see site 251 | | | | | | | | |
| 259 | Skřivánek | Etická komise Fakultní nemocnice Olomouc | 42/12 | Doc. MUDr. Vladko Horčička, CSc. | 775 20 | Olomouc | I. P. Pavlova 6 | +420 588 442 477 | +420 588 442 477 | iveta.sudolska@fnol.cz |
| 260 | Hlaváčková | see MEC | | | | | | | | |
| 261 | Dvořák | Etická komise Fakultní nemocnice Brno | 65M,L-2011 | PharmDr. Šárka Kozáková | 625 00 | Brno | Jihlavská 20 | +420 532 232 798 | +420 547 211 961 | etickakomise@fnbrno.cz |
| 262 | Hořejší | see MEC | | | | | | | | |
| 263 | Jeníček | see MEC | | | | | | | | |
| 264 | Stará | see MEC | | | | | | | | |
| 265 | Sýkorová | see MEC | | | | | | | | |
| 266 | Vetešníková - Koubová | see MEC | | | | | | | | |
| leading EC (MEC) | | Multicentrická etická komise Fakultní nemocnice v Motole | EK-110/12 | MUDr. Vratislav Šmelhaus | 150 06 | Praha 5 – Motol | V Úvalu 84 | +420 224 431 195, +420 224 431 197 | +420 224 431 196 | etickakomise@fnmotol.cz |

| ETHICS COMMITTEES CF111/302 | | | | | | | | | | |
|---|---|---|---|---|---|---|---|---|---|---|
| NO. | INVESTIGATOR | EC | Reference no. | CHAIRMAN | POST CODE | CITY | STREET / NUMBER | TEL NUMBER | FAX NUMBER | EMAIL |
| Germany | | | | | | | | | | |
| 351 | Maar | see leading ethics committee | | | | | | | | |
| 352 | Alberti | Ethik-Kommission der Bayerischen Landesärztekammer | 7/11365 | NA | 81677 | München | Mühlbaurstraße 16 | +49 89 4147 165 | +49 89 4147 280 | ethikkommission@blaek.de |
| 353 | Bühling | Ethik-Kommission der Ärztekammer Hamburg | MC-418/11 | NA | 22083 | Hamburg | Humboldtstr. 67 a | +49 40 20 22 99 240 | +49 40 20 22 99 410 | ethik@aekhh.de |
| 354 | Burgkhardt | Ethikkommisson bei der Sächsischen Landesärztekammer | EK-AMG-MCB-189 / 11-1 | NA | 01099 | Dresden | Schützenhöhe 16 | +49 351 8267 333 | +49 351 8267 412 | ethik@slaek.de |
| 365 | Dodt | see leading ethics committee | | | | | | | | |
| 356 | Gerlach | Ethikkommission der Ärztekammer Nordrhein | 2011466 | NA | 40474 | Düsseldorf | Tersteegenerstr. 9 | + 49 211 4302 0 | + 49 211 4302 1200 | ethik@aekno.de |
| 357 | Göckeler-Leopold | Ethik-Kommission der Ärztekammer Westfalen-Lippe | 2011-675-b-A | NA | 48147 | Münster | Gartenstr. 210 - 214 | +49 251 929 2460 | +49 251 929 2478 | ethik-kommission@aekwl.de |
| 358 | Groven | Ethikkommission zur Beurteilung medizinischer Forschung am Menschen | NA | NA | 30175 | Hannover | Berliner Allee 20 | +49 511 380 2208 | +49 511 380 2119 | ethikkommission@aekn.de |
| 359 | Knauss | Ethikkommission der Landesärztekammer Baden-Württemberg | B-AM-2011-204 | NA | 70597 | Stuttgart | Jahnstr. 40 | +49 711 769 89 0 | +49 711 769 89 50 | info@laek-bw.de |
| 360 | Kränzlin | see site 352 | | | | | | | | |
| 361 | Krossin | see site 353 | | | | | | | | |
| 362 | Peters | see site 353 | | | | | | | | |
| 363 | Royar | see site 359 | | | | | | | | |
| 364 | Ruhmland | see leading ethics committee | | | | | | | | |
| 365 | Ahrendt | Ethikkommission des Landes Sachsen-Anhalt | EK-12/008 | NA | 06846 | Dessau -Roßlau | Kühnauer Straße 70 | +49 340 65 01 291 | +49 340 65 01 199 | ek@lav.ms.sachsen-anhalt.de |
| 366 | Büchau | see leading ethics committee | | | | | | | | |
| 367 | Cassel | Ethikkommission der Landesärztekammer Hessen | MC 364/2011 | NA | 60488 | Frankfurt/Main | Im Vogelsgesang 3 | +49 69 97672 0 | + 49 69 97672 128 | info@laekh.de |
| 368 | Dilenge | see site 352 | | | | | | | | |
| 370 | Göttker-Schnetmann | see site 367 | | | | | | | | |
| 371 | Hoppe | see site 359 | | | | | | | | |
| 372 | Schlothauer | see leading ethics committee | | | | | | | | |
| lead EC | | Landesamt für Gesundheit und Soziales Berlin, Geschäftsstelle der Ethik-Kommission des Landes Berlin | 11/0606 - EK | Prof. Dr. Hildebrandt | 10707 | Berlin | Fehrbellinger Platz 1 | +49 30 90229 1220 | +49 30 9028 3383 | ethik-kommission@lageso.berlin.de |

| ETHICS COMMITTEES CF111/302 | | | | | | | | | | |
|---|---|---|---|---|---|---|---|---|---|---|
| NO. | INVESTIGATOR | EC | Reference no. | CHAIRMAN | POST CODE | CITY | STREET / NUMBER | TEL NUMBER | FAX NUMBER | EMAIL |
| | Hungary | | | | | | | | | |
| 451 | Spánik | Metropolitan Municipality St. John's Hospital and Corporated Hospitals of North Buda, Tudományos Bizottság/Research Committee | NA | Prof. Dr. András Jánosi (info dated: 24.08.2012) | 1125 | Budapest | Diós árok 1-3. | NA | NA | NA |
| 452 | Zámbó | Intézményi Kutatásetikai Bizottság/Institutional Research Ethics Committee | NA | Dr. Ildikó Rosta (info dated 27.05.2011) | 3000 | Hatvan | Balassi Bálint str. 16 | NA | NA | NA |
| 453 | Langmár | Ferencvárosi Egészségügyi Szolgáltató Kiemelkedően Közhasznú Nonprofit Kft. Intézményi Kutatásetikai Bizottság/Institutional Research Ethics Committee | NA | Dr. Imre Guller (last info dated: 03.04.2012) | 1096 | Budapest | Mester u. 45. | NA | NA | NA |
| 454 | Hornádi | Markhot Ferenc Hospital Health Provider Nonprofit Advanced Utility Kft., Intézményi Kutatásetikai Bizottság/Institutional Research Ethics Committee | NA | Dr. László Hornádi (info dated: 23.05.2011) | 3300 | Eger | Széchenyi u. 27-29. | NA | NA | NA |
| 455 | Szónyi | Ferencvárosi Egészségügyi Szolgáltató Kiemelkedően Közhasznú Nonprofit Kft. Intézményi Kutatásetikai Bizottság/Institutional Research Ethics Committee | NA | Dr. Imre Guller (last info dated: 03.04.2012) | 1095 | Budapest | Mester u. 45. | NA | NA | NA |
| 456 | Urbán | Metropolitan Municipality St. Imre Hospital , Intézményi Kutatásetikai Bizottság/Institutional Research Ethics Committee | NA | No details were that time available.(info dated: 26.05.2011) | 1115 | Budapest | Tétényi str. 12-16. | NA | NA | NA |
| 457 | Pódör | Komárom -Esztergom County St. Borbala Hospital, Intézményi Kutatásetikai Bizottság/Institutional Research Ethics Committee | NA | Dr. Gábor Nagy (info dated: 06.06.2011) | 2800 | Tatabánya | Dózsa György str. 77. | NA | NA | NA |
| 458 | Gyarmati | Metropolitan Municipality Uzsoki Hospital, Intézményi Kutatásetikai Bizottság/Institutional Research Ethics Committee | NA | Dr. Tibor Milibák (info dated: 07.06.2011) | 1145 | Budapest | Uzsoki str. 29-41. | NA | NA | NA |
| 459 | Horváth | Ferencvárosi Egészségügyi Szolgáltató Kiemelkedően Közhasznú Nonprofit Kft. Intézményi Kutatásetikai Bizottság/Institutional Research Ethics Committee | NA | Dr. Imre Guller (last info dated: 03.04.2012) | 1095 | Budapest | Mester u. 45. | NA | NA | NA |
| 460 | Szentpéteri | Institutional Ethics Commitee of II. Rákóczi Ferenc Hospital | NA | Dr. István Tóth (last info dated 17.05.2014) | 3800 | Szikszó | Kassai út 45-49 | NA | NA | NA |
| 461 | Molnár | Institutional Ethics Commitee of St. John Hospital and North-Buda United Hospitals, | NA | Prof. Dr. András Jánosi (info dated: 24.08.2012) | 1125 | Budapest | Diós árok 1-3 | NA | NA | NA |
| 462 | NOVÁK | Institutional Ethics Commitee of Ferencvárosi Health Provider Nonprofit Advanced Public Utility Kft. | NA | Dr. Imre Guller (last info dated: 03.04.2012) | 1096 | Budapest | Mester u. 45 | NA | NA | NA |
| 463 | SZÉKELY | see 462 | | | | | | | | |
| 464 | Pálmai | Institutional Ethics Commitee of Józsefvárosi Health Provider Kft. | NA | Dr. Judit Faragó (last info dated 11.02.2014), secretary: Julianna Juhász Szabóné | 1084 | Budapest | Auróra u. 22-28 | NA | NA | NA |
| lead EC | | Medical Research Council Ethics Committee For Clinical Pharmacology | 19021-0/2013-EKL | Dr. FÜRST, Zsuzsanna contact person: Istvánné Magyari | 1051 | Budapest | Arany János u. 6-8. | +36 1 795 1195 | +36 1 795 0168 | istvanne.magyari@nefmi.gov.hu |
| | Poland | No local ECcs in Poland | | | | | | | | |
| 551 | Jaszczyński | Ethics Committee at Local Medical Chamber ul. Krupnicza 11A 31-123 Kraków | Opinion No.: 6 /KBL/OIL/2012 | Mariusz Janikowski, MD | 31-123 | Kraków | Krupnicza 11 A | +48 12 619 17 12 | + 48 12 422 57 55 | a.krawczyk@hipokrates.org |
| 552 | Faska | | | | | | | | | |
| 553 | Wiśniewska-Sawicka | | | | | | | | | |
| 554 | Bojar | | | | | | | | | |
| 555 | Chełmicki | | | | | | | | | |
| 556 | Jakimiuk | | | | | | | | | |
| 557 | Pakalski | | | | | | | | | |
| 558 | Jędrzejczyk | | | | | | | | | |
| 559 | Pięta-Dolińska | | | | | | | | | |
| 560 | Adamczyk-Gruszka | | | | | | | | | |
| 561 | Malinowski | | | | | | | | | |
| 562 | Skrzypulec-Plinta | | | | | | | | | |
| 563 | Nawara-Baran | | | | | | | | | |
| 564 | Brankowska-Kozerawska | | | | | | | | | |
| 565 | Jędrzejczyk | | | | | | | | | |
| 566 | Połac | | | | | | | | | |
| 567 | Radecka | | | | | | | | | |
| 568 | Tomaszewski | | | | | | | | | |

| NO. | INVESTIGATOR | EC | Reference no. | CHAIRMAN | POST CODE | CITY | STREET / NUMBER | TEL NUMBER | FAX NUMBER | EMAIL |
|---|---|---|---|---|---|---|---|---|---|---|
| ETHICS COMMITTEES CF111/302 | | | | | | | | | | |
| **Romania** | | **No local ECcs in Romania** | | | | | | | | |
| 651 | Manescu /not approved | NATIONAL BIOETHICS COMMITTEE FOR MEDICINES AND MEDICAL DEVICES | 275; 2358; 3302 / 03.10.2012 | Prof. Dr. Dinu Antonescu | 020125 | Bucuresti | Sos. STEFAN CEL MARE NR. 19-21, SECTOR 2 (pavilion K, Colentina Hospital) | +4021.210.28.80 | +4021.210.28.81 | comisia.bioetica@adsm.ro |
| 652 | Cornea | | | | | | | | | |
| 653 | Dumitrascu | | | | | | | | | |
| 654 | Gheorman/not approved | | | | | | | | | |
| 655 | Tuttunaru | | | | | | | | | |
| 656 | Munteanu/not approved | | | | | | | | | |
| 657 | Gogonea | | | | | | | | | |
| 658 | Rotaru | | | | | | | | | |
| 659/back-up | Costea/not approved | | | | | | | | | |
| 660/back-up | Gheorghiu / not submitted yet | | | | | | | | | |
| 661/back-up | Moraru/not approved | | | | | | | | | |
| 662/back-up | Edu / not submitted yet | | | | | | | | | |
| **Spain** | | | | | | | | | | |
| 751 | Santiago Palacios | | | see Reference EC | | | | | | |
| 752 | Rafael Sánchez | IDIAP Jordi Gol i Gurina | NA | Contact Person: Mari Pau Moreno | 08007 | Barcelona | Av. Gran Via de les Corts Catalanes, 591 Àtic | 0034 93 482 45 72 | 0034 93 482 41 74 | pmoreno@idiapjgol.org<br>AGE.SIC@idiapjgol.org |
| 753 | Rafael Sánchez | | | See Site 752 | | | | | | |
| 754 | Elena Jiménez | Comitè Ètic d'Investigació Clínica de les Illes Balears | NA | Contact Person: Lourdes de la Vega | 07010 | Palma de Mallorca | C/ Camí de Jesús, 38 A | 0034 971 177 378 | 0034 971 177 307 | ldelavega@dgaval.caib.es |
| 755 | Silvia González | | | See Site 751 | | | | | | |
| 756 | Esther de la Viuda | CEIC Hospital General Universitario de Guadalajara | NA | Contact Person: Pilar Martinez | 19002 | Guadalajara | C/ Donantes de Sangre, s/n | 0034 949 209 216 | 0034 949 209 216 | pmartinezm@jccm.es |
| 757 | Elena Álvarez | | | See Site 752 | | | | | | |
| 758 | Fernando Cereto | Fundació Unió Catalana d'Hospitals | NA | Contact Person: Vanessa Massó | 08009 | Barcelona | C/ Bruc, 72-74 1º | 0034 552 92 08 | 0034 93 414 71 22 | ceic@uch.cat |
| 759 | Josep Grau | CEIC Hospital General de Vic - Fundació FORES | NA | Contact Person: Lidia Solerdelcoll | 08500 | Vic | C/ Francesc Pla "El Vigatà", 1 | 0034 93 702 77 13 | 0034 93 885 03 08 | lsolerdelcoll@chv.cat |
| 760 | Antoni Pessarrodona | CEIC Hospital Mútua Terrassa | NA | Contact Person: Susana Redondo | 08221 | Terrassa | Plaza Dr. Robert, 5 | 0034 93 736 50 50/ Ext. 1032 | 0034 93 736 50 59 | ceichmt@mutuaterrassa.es |
| 761 | Ernest Balaguer | CEIC Capio Hospital General de Catalunya | NA | Contact Person: Montse Granados | 08190 | Sant Cugat del Vallès | C/ Pere i Pons, 1 | 0034 93 565 60 00/ Ext. 5077 | 0034 93 589 24 98 | mgranado@hgc.es<br>ceic.idcsa.cat@idcsalud.es |
| 762 | Carme Bergós | | | See Site 752 | | | | | | |
| **Reference EC (lead)** | | **CEIC Área 2 - Hospital Universitario de La Princesa** | **NA** | NA | **28006** | **Madrid** | **C/ Diego de León, 62** | **0034 91 520 24 76** | **0034 91 520 25 60** | **secreceic.hlpr@salud.madrid.org<br>ceic.hlpr@salud.madrid.org** |
| **Slovakia** | | | | | | | | | | |
| 851 | Danko | Etická komisia JLF UK | EK 1151/2012 | Prof. MUDr. Gabriela Nosáľová, DrSc. | 037 53 | Martin | Sklabinská 26 | +421 434 132 535 | +421 434 134 807 | nosalova@jfmed.uniba.sk |
| 852 | Cupaník | Etická komisia GPN | NA | MUDr. Viera Lesná | 811 03 | Bratislava | Partizánska 27 | +421 254 640 091 | +421 254 640 092 | lesna@gpn.sk |
| 853 | Bielik | Etická komisia Banskobystrického samosprávneho kraja | NA | Mgr. Katarína Čupková | 974 01 | Banská Bystrica | Námestie SNP 23 | +421 484 325 570 | +421 484 325 515 | zdenka.kadasiova@vucbb.sk |
| 854 | Petrovičová | see site 853 | see site 853 | see site 853 | see site 853 | | | | | |
| 855 | Sládičeková | Etická komisia Bratislavského samosprávneho kraja | NA | MUDr. Valerián Potičný | 820 05 | Bratislava 25 | Sabinovská 16 | +421 248 264 823 | +421 248 264 386 | katarina.molnarova@region-bsk.sk |
| 856 | Bachňáková | | | see site 853 | | | | | | |
| 857 | Biringer | | | see site 851 | | | | | | |
| 858 | Fathiová | Etická komisia Prešovského samosprávneho kraja | NA | MUDr. Július Zbyňovský, MPH | 080 01 | Prešov | Námestie mieru 2 | +421 517 081 635 | +421 517 481 638 | lubica.cuperova@vucpo.sk |
| 859 | Gáťová | | | see site 851 | | | | | | |
| 860 | Šuteková | | | see site 853 | | | | | | |
| **lead EC** | | **Etická komisia JLF UK** | **EK 1072/2012** | **Prof. MUDr. Gabriela Nosáľová, DrSc.** | **037 53** | **Martin** | **Sklabinská 26** | **+421 434 132 535** | **+421 434 134 807** | **nosalova@jfmed.uniba.sk** |
