# Supplementary material for: Bleeding profile of women using a drospirenone-only pill 4 mg over nine cycles in comparison with desogestrel 0.075 mg
Source: PLoS One. 2020 Jun 29;15(6):e0231856. doi: 10.1371/journal.pone.0231856 (PMC7323950; doi:10.1371/journal.pone.0231856)
Supplement: S4 File — (PDF) [file pone.0231856.s005.pdf]

Appendix 1

List of IECs or IRBs -Study

Czech Republic (Central IEC)
Etická komise pro multicentrické klinické hodnocení
Chair: Vratislav Šmelhaus, MD
Contact person: Mgr. Vlastimil Holíček
V úvalu 84
150 06 Praha 5 - Motol
Phone: + 420 224 431 195, + 420 224 431 197, mobile: 00 420 721 309 300
Fax: + 420 224 431 196
E-mail: etickakomise@fnmotol.cz

Germany
Leading IEC
Ethikkommission des Landes Sachsen-Anhalt, Geschäftsstelle
Contact person: Henning Richter
Kühnauer Straße 70
06846 Dessau -Roßlau
Phone: +49 340 65 01 291
Fax: +49 340 65 01 199
E-mail: ek@lav.ms.sachsen-anhalt.de

Ethik-Kommission der Bayerischen Landesärztekammer
Contact person: Ms. Henne
Mühlbaurstraße 16
81677 München
Phone: +49 89 4147 165, +49 89 4147 335 (Ms. Henne)
Fax: +49 89 4147 280, +49 89 4147 334 (Ms. Henne)[1]
E-mail: ethikkommission@blaek.de

Ethikkommission der Landesärztekammer Hessen
Contact person: Gisela Gerke
Im Vogelsgesang 3
60488 Frankfurt/Main
Phone: +49 69 97672 0, +49 69 97672 119 (G. Gerke)
Fax: + 49 69 97672 128, +49 69 97672 377 (G. Gerke)
E-mail: info@laekh.de, ethikkommission@laekh.de, gisela.gerke@laekh.de
Ethikkommission der Ärztekammer Nordrhein
Contact persons: Andrea Nassiri, Claire Rivoire
Tersteegenstr. 9
40474 Düsseldorf
Phone: + 49 211 4302 1581, +49 211 4302 2272 (A. Nassiri),
+49 211 4302 2273 (C. Rivoire)
Fax: +49 211 4302 1585, +49 211 4302 2279
E-mail: ethik@aekno.de

Ethik-Kommission der Ärztekammer Hamburg
Chair: Karin Küchenmeister
Humboldtstr. 67 a
22083 Hamburg
Phone: +49 40 20 22 99 0, +49 40 20 22 99 240
Fax: +49 40 20 22 99 400, +49 40 20 22 99 410
E-mail: post@aekhh.de, ethik@aekhh.de

Landesamt für Gesundheit und Soziales Berlin, Geschäftsstelle der Ethik-Kommission des Landes Berlin
Contact persons: Sabine Pieprzyk, Susann-Isabelle Grambow
Fehrbelliner Platz 1
10707 Berlin
Phone: +49 30 90229 1220, +49 30 90229 1227 (S. Pieprzyk),
+49 30 90229 1228 (I. Grambow)
Fax: +49 30 9028 3383
E-mail: poststelle@lageso.berlin.de, sabine.pieprzyk@lageso.berlin.de ,
isabelle.grambow@lageso.berlin.de

Ethikkommission der Landesärztekammer Baden-Württemberg
Contact person: Anna Gola
Jahnstr. 40
70597 Stuttgart
Phone: +49 711 769 89 0, +49 711 769 89 60 or 55,
Fax: +49 711 769 89 50, +49 711 769 89 856
E-mail: info@laek-bw.de, anna.gola@laek-bw.de

Ethikkommission zur Beurteilung medizinischer Forschung am Menschen
Contact person: Kai Bogs
Berliner Allee 20
30175 Hannover
Phone: +49 511 380 2208
Fax: +49 511 380 2119
E-mail: ethikkommission@aekn.de

Hungary

Institutional Ethics Committee
Szegedi úti Szakrendelő
3
XIII. kerületi Egészségügyi Szolgálat Közhasznú Nonprofit Kft.
Chair: Dr. Klára ESZTÓ
Szegedi út 17
1139 Budapest
Phone: +36 1 4524 200

Institutional Research Ethics Committee
Ferencvárosi Egészségügyi Szolgáltató Kiemelten Közhasznú Nonprofit Kft.

Chair: Dr. Imre Guller
Mester u. 45
1095 Budapest
Phone: +36 1 4554 570
Fax: +36 1 4554 504
E-mail: gullerimre@gmail.com

Clinical Research Ethics Committee
Egyesített Szent István és Szent László Kórház - Rendelőintézet
Chair: Dr. Dénes Bánhegyi
Nagyvárad tér 1
1096 Budapest
Phone: +36 1 4558 102
Fax: +36 1 2156 501
E-mail: orv_ig@laszlokorhaz.hu
Lead EC
Medical Research Council
Ethics Committee for Clinical Pharmacology
Chair: Prof. Dr. Zsuzsanna Fürst
Phone contact: Istvánné Magyari
Arany J. u. 6-8
4
1051 Budapest
Phone: +36 1 7951-195, +36 30 7951-195
Fax: +36 1 7950-168
E-mail: istvanne.magyari@emmi.gov.hu

Poland (Central IEC)
Ethics Committee at Local Medical Chamber
Chair: Mariusz Janikowski MD
ul. Krupnicza 11A
31-123 Kraków
Phone: +48 12 619 17 12
Fax: +48 12 422 57 55
E-mail: aneta@oil.org.pl.

Romania (Central IEC)
Comisia Nationala de Etica pentru Studiul Clinic al Medicamentului
Chair: Prof. Dr. Sava Dumitrescu
011478 Bucharest
str. Aviator Sanatescu, nr.48, sector 1
Phone: +4031 4051076
Fax: +4031 4051075

List of IECs or IRBs – Study 2
Austria (Central IEC)

Ethikkommission der Medizinischen Universität Wien
Borschkegasse 8b/E06

1090 Wien
Phone: +43 (01) 40400-2147, -2248, -2241
Fax: +43 (01) 40400-1690
E-mail: ethik-kom@meduniwien.ac.at

Czech Republic (Central IEC)
Multicentrická etická komise Fakultní nemocnice v Motole
Chair: Vratislav Šmelhaus, MD
V úvalu 84
150 06 Praha 5 - Motol
Phone: + 420 224 431 195, + 420 224 431 197,
Fax: + 420 224 431 196
E-mail: etickakomise@fnmotol.cz

Etická komise, Centrum neurologické péče, s.r.o.
Chair: MUDr. Radomír Štrupl
Jiráskova 1389
51601 Rychnov nad Kněžnou
Phone: +420 491 112 724
Fax: +420 491 112 725
E-mail: helena@neurol.cz
1

Etická komise Fakultní nemocnice Brno
Chair: PharmDr. Šárka Kozáková
Jihlavská 20
62500 Brno
Phone: +420 532 232 798
Fax: +420 547 211 961
E-mail: etickakomise@fnbrno.cz

Etická komise Fakultní nemocnice Ostrava
Chair: MUDr. Luděk Rožnovský, CSc.
17. listopadu 1790
70852 Ostrava-Poruba
Phone: +420 597 372 542
Fax: +420 597 374 801
E-mail: eticka.komise@fno.cz

Etická komise Fakultní nemocnice Olomouc
Chair: Doc. MUDr. Vladko Horčička, CSc.
I. P. Pavlova 6
77520 Olomouc
Phone: +420 588 442 477
Fax: +420 588 442 477
E-mail: iveta.sudolska@fnol.cz

Germany

Leading IEC
Landesamt für Gesundheit und Soziales Berlin, Geschäftsstelle der Ethik-Kommission desLandes Berlin
Contact person: Dr. Christian von Dewitz[2]
Fehrbelliner Platz 1
10707 Berlin
Phone: +49 30 90229 1220
Fax: +49 30 9028 3383
E-mail: ethik-kommission@lageso.berlin.de

Ethik-Kommission der Ärztekammer Hamburg
Chair: Karin Küchenmeister
Humboldtstr. 67 a
22083 Hamburg
Phone: +49 40 20 22 99 240
Fax: +49 40 20 22 99 410
E-mail: ethik@aekhh.de

Ethikkommisson bei der Sächsischen Landesärztekammer
Schützenhöhe 16
01099 Dresden
Phone: +49 351 8267 333
Fax: +49 351 8267 412
E-mail: ethik@slaek.de

Ethikkommission zur Beurteilung medizinischer Forschung am Menschen
Contact person: Kai Bogs
Berliner Allee 20
30175 Hannover
Phone: +49 511 380 2208
Fax: +49 511 380 2119
E-mail: ethikkommission@aekn.de[3]

Ethik-Kommission der Bayerischen Landesärztekammer
Contact person: Mrs. Schulz Kuhn
Mühlbaurstraße 16
81677 München
Phone: +49 89 4147 165
Fax: +49 89 4147 280
E-mail: ethikkommission@blaek.de

Ethikkommission des Landes Sachsen-Anhalt, Geschäftsstelle
Contact person: Henning Richter
Kühnauer Straße 70
06846 Dessau -Roßlau
Phone: +49 340 65 01 291
Fax: +49 340 65 01 199

E-mail: ek@lav.ms.sachsen-anhalt.de

Ethikkommission der Landesärztekammer Hessen
Contact person: Gisela Gerke
Im Vogelsgesang 3
60488 Frankfurt/Main
Phone: +49 69 97672 0,
Fax: + 49 69 97672 128,
E-mail: info@laekh.de, info@laekh.de

Ethikkommission der Landesärztekammer Baden-Württemberg
Contact person: Anna Gola
Jahnstr. 40
70597 Stuttgart[4]
Phone: +49 711 769 89 0
Fax: +49 711 769 89 50
E-mail: info@laek-bw.de

Hungary

Metropolitan Municipality St. John's Hospital and Corporated Hospitals of North Buda,Tudományos Bizottság/Research Committee
Chair: Prof. Dr. András Jánosi
Diós árok 1-3.
1125 Budapest

Intézményi Kutatásetikai Bizottság/Institutional Research Ethics Committee
Chair: Dr. Ildikó Rosta
Balassi Bálint str. 16
3000 Hatvan

Ferencvárosi Egészségügyi Szolgáltató Kiemelkedően Közhasznú Nonprofit Kft. Intézményi
Kutatásetikai Bizottság/Institutional Research Ethics Committee
Chair: Dr. Imre Guller
Mester u. 45.
1095 Budapest

Markhot Ferenc Hospital Health Provider Nonprofit Advanced Utility Kft., Intézményi
Kutatásetikai Bizottság/Institutional Research Ethics Committee
Chair: László Hernádi
Széchenyi u. 27-29.
3300 Eger[5]

Komárom -Esztergom County St. Borbala Hospital, Intézményi Kutatásetikai Bizottság/Institutional Research Ethics Committee
Chair: Dr. Gábor Nagy

Dózsa György str. 77.
2800 Tatabánya

Institutional Ethics Commitee of II. Rákóczi Ferenc Hospital
Chair:
Kassai út 45-49
3800 Szikszó

Institutional Ethics Commitee of St. John Hospital and North-Buda United Hospitals,
Chair: Prof. Dr. András Jánosi
Diós árok 1-3.
1125 Budapest

Institutional Ethics Commitee of Ferencvárosi Health Provider Nonprofit Advanced Public
Utility Kft.
Chair: Dr. Imre Guller
Mester u. 45.
1095 Budapest

Institutional Ethics Commitee of Józsefvárosi Health Provider Kft.
Auróra u. 22-28
1084 Budapest
Lead EC
Medical Research Council[6]
Ethics Committee for Clinical Pharmacology
Chair: Prof. Dr. Zsuzsanna Fürst
Phone contact: Istvánné Magyari
Arany János u. 6-8.
1051 Budapest
Phone: +36 1 7951195
Fax: +36 1 7950-168
E-mail: istvanne.magyari@emmi.gov.hu

Poland
Central IEC
Ethics Committee at Local Medical Chamber
Chair: Mariusz Janikowski MD
ul. Krupnicza 11A
31-123 Kraków
Phone: +48 12 619 17 12
Fax: +48 12 422 57 55
E-mail: akrawczyk@hipokrates.org

Romania
Central IEC
Comisia Nationala de Etica pentru Studiul Clinic al Medicamentului

National Etichs Committee for the Drug Clinical Study
Chair: Prof. Dr. Sava Dumitrescu
011478 Bucharest
str. Aviator Sanatescu, nr.48, sector 1
Phone: +403 1405 1076
Fax: +403 1405 1075

Spain
Lead EC
CEIC Área 2 - Hospital Universitario de La Princesa
Contact persons: Cecília López / Julio González
C/ Diego de León, 62[7]
28006 Madrid
Phone: +34 91 520 2476
Fax: +34 91 520 2560
E-mail: ceic.hlpr@salud.madrid.org

IDIAP Jordi Gol i Gurina
Contact person: Mari Pau Moreno
Av. Gran Via de les Corts Catalanes, 591 Àtic
08007 Barcelona
Phone: +34 93 482 4572
Fax: ++34 93 482 4174
E-mail: pmoreno@idiapjgol.org

CEIC Hospital General de Vic - Fundació FORES
Contact person: Lidia Soler
C/ Francesc Pla "El Vigatà", 1
08500 Vic
Phone: +34 93 702 7713
Fax: +34 93 885 0308
E-mail: lsolerdelcoll@chv.cat

CEIC Hospital Mútua Terrassa
Contact person: Susana Redondo
Plaza Dr. Robert, 5
08221 Terrassa
Phone: +34 93 736 5050/Exit. 1032
Fax: +34 93736 5059
E-mail: ceichmt@mutuaterrassa.es[8]

CEIC Capio Hospital General de Catalunya
Contact person: Montse Granados
C/ Pere i Pons, 1
08195 Sant Cugat del Vallès
Phone: +34 93565 6000/Exit.5077
Fax: +34 93589 2498
E-mail: mgranado@hgc.es

Slovakia
Lead EC
Etická komisia JLF UK
Chair: Prof. MUDr. Gabriela Nosáľová, DrSc.
Sklabinská 26
03753 Martin
Phone: +421 434 132 535
Fax: +421 434 134 807
E-mail: nasalova@jfmed.uniba.sk

Etická komisia GPN
Chair: MUDr. Viera Lesná.
Partizánska 27
81103 Bratislava
Phone: +421 254 640 091
Fax: +421 254 640 092
E-mail: lesna@gpn.sk
Etická komisia Banskobystrického samosprávneho kraja
Chair: Mgr. Katarína Čupková
Námestie SNP 23[9]
97401 Banska Bystrica
Phone: +421 484 325 570
Fax: +421 484 325 515
E-mail: zdenka.kadasiova@vucbb.sk

Etická komisia Bratislavského samosprávneho kraja
Chair: MUDr. Valerián Potičný
Sabinovská 16
82005 Bratislava 25
Phone: +421 248 264 823
Fax: +421 248 264 386
E-mail: katarina.molnarova@region-bsk.sk

Etická komisia Prešovského samosprávneho kraja
Chair: MUDr. Július Zbyňovský, MPH
Námestie mieru 2
08001 Prešov
Phone: +421 517 081 635
Fax: +421 517 481 638
E-mail: lubica.cuperova@vucpo.sk
